# Azooxanthellate Scleractinia (Cnidaria, Anthozoa) from South Africa

**DOI:** 10.3897/zookeys.1066.69697

**Published:** 2021-10-28

**Authors:** Zoleka N. Filander, Marcelo V. Kitahara, Stephen D. Cairns, Kerry J. Sink, Amanda T. Lombard

**Affiliations:** 1 Biodiversity and Coastal Research, Oceans and Coasts, Department of Environment, Forestry, and Fisheries, Cape Town, South Africa; 2 Zoology Department, Nelson Mandela University, Port Elizabeth, South Africa; 3 Universidade Federal de São Paulo, Departamento de Ciências do Mar, Santos, Brazil; 4 Centro de Biologia Marinha, Universidade de São Paulo, São Sebastião, Brazil; 5 Department of Invertebrate Zoology, Smithsonian Institution, Washington DC, USA; 6 South African National Biodiversity Institute, Cape Town, South Africa; 7 Institute for Coastal and Marine Research, Nelson Mandela University, Port Elizabeth, South Africa

**Keywords:** Ahermatypic, corals, hermatypic, revision, taxonomy

## Abstract

Globally, South Africa ranks in the top five countries regarding marine species richness per unit area. Given the high diversity, it is not surprising that many invertebrate taxa in the region are poorly characterised. The South African azooxanthellate Scleractinia (Anthozoa) is one such taxonomic group, and was last reviewed by Boshoff in 1980. Although more recent regional publications have reported on some species, there has not been a faunistic review that accounts for the country’s species diversity since then. Moreover, numerous unidentified specimens representing more than three decades of sampling effort have accumulated. In this study the authors update the state of knowledge of South African azooxanthellate coral species. Specimens, particularly those within the extensive collections of the Iziko South African and Smithsonian museums, were morphologically examined and identified. Other data considered included historic data represented as imagery data, associated species data from recent research surveys, and the scientific literature. To date, the study has increased the total number of known species from 77 to 108 across eleven families, 28 new South African records, and three are new species with one new genus.

## Introduction

The South African marine environments host a variety of fauna that encompasses at least 12,000 species (Griffiths et al. 2010), although many benthic taxa remain poorly understood (Gibbons 1999; Griffiths et al. 2010). This diversity, and a high level of endemism of species, is influenced by the dynamic nature of the oceanographic regimes that surround the country (Brown and Jarman 1978; Thandar 1989; Awad et al. 2002; Griffiths et al. 2010). These regimes are primarily distinguished by water temperature. The cold, upwelling nutrient-rich Benguela Current flowing northwards along the Atlantic margin results in high biological productivity, which in turn supports high species abundance (Shannon and Nelson 1996), but low species diversity (Griffiths et al. 2010). Along the eastern margin the warm fast southward-flowing Agulhas Current brings nutrient-poor waters, which drive high species diversity in this region (Heydorn et al. 1978). Both these currents interact in the Southern margin region resulting in a unique environment which promotes high endemism patterns for most benthic invertebrate marine fauna (Lutjeharms et al. 2000; Awad et al. 2002).

This heterogenous physical environment underpins South Africa’s high marine species per unit area, which the country is reported to rank amongst the top five globally (Costello et al. 2010). However, it is estimated that more than a third of the country’s fauna remains to be characterised (Costello et al. 2010). This paucity of taxonomic knowledge is particularly acute for the deeper waters (> 200 m), which represent 90% of the marine territory (Lombard et al. 2004; Costello et al. 2010; Griffiths et al. 2010; Sink et al. 2012, 2019). In addition to the existing challenges in sampling efforts across the shallow and offshore regions (Griffiths et al. 2010), the lack of regional taxonomists (Gibbons et al. 1999) has also contributed to the high estimates of undescribed species (Costello et al. 2010; Griffiths et al. 2010). The importance of regional guides has been emphasised by Costello et al. (2010), who demonstrated how published monographs and guides within a region correlate with the state of knowledge of known taxa. These findings are in keeping with the observations of Gibbons et al. (1999) and Griffiths et al. (2010) that South Africa has low taxonomic efforts despite its high biodiversity. Although regional expertise has recently expanded within invertebrate groups such as Echinodermata (Olbers 2016; Filander and Griffiths 2017), Crustacea (Biccard 2012; Landschoff 2011), and one other Cnidaria class, the Actiniaria (Laird 2013), there is still a notable gap in taxonomy of several other cnidarian orders.

One specific group with a paucity in regional taxonomic understanding is the azooxanthellate scleractinian corals and this is the focus of the work presented here. Our knowledge on the South African azooxanthellate scleractinian corals is based on ten publications (Duncan 1876; Gardiner’s 1902a, b, 1904; van der Horst 1927, 1938; Boshoff 1981; Zibrowius and Gili 1990; Cairns and Keller 1993; Cairns and Zibrowius 2016) none of which holistically details the South African fauna across the oceanographic regimes (Agulhas and Benguela regions). A significant contribution to the South African azooxanthellate fauna is documented in the 20^th^ century (Table 1).

**Table 1.** Significant papers contributing to the South African azooxanthellate coral fauna to date. References are presented chronologically (denoted by capital letters: **A** = Duncan 1876, **B** = Gardiner 1902a, **C** = Gardiner 1902b, **D** = Gardiner 1904, **E** = von Marenzeller 1904, **F** = van der Horst 1927, **G** = van der Horst 1933, **H** = van der Horst 1938, **I** = Boshoff 1981, **J** = Zibrowius and Gili 1990, **K** = Cairns and Keller 1993, **L** = Cairns and Zibrowius 2016, and **M** = present study) and then following family and species alphabetically; for which the first column lists the accepted species name at time of publication followed by an authority column. New species records are identified by a numeric superscript.

Species	Authority	References												
		A	B	C	D	E	F	G	H	I	J	K	L	M
*^1.^ Anomocorafecunda*	(Pourtalès, 1871)													X
*Anomocoramarchadi*	(Chevalier, 1966)				X					X		X		
*^2.^Aulocyathus* sp. cf. matricidus	(Kent, 1871)													X
*^3.^ Aulocyathusjuvenescens*	von Marenzeller, 1904a													X
*^4.^ Caryophyllia(A.)dentata*	(Moseley, 1881)													X
*^5.^ Caryophyllia(A.)grayi*	(Moseley, 1881)													X
Caryophyllia (C.) cinticulata	(Alcock, 1898)				X									
*^6.^ Caryophyllia(C.)diomedeae*	von Marenzeller, 1904b													X
Caryophyllia (C.) ephyala	Alcock, 1891				X									
Caryophyllia (C.) grandis	Gardiner & Waugh, 1938											X		
Caryophyllia (C.) lamellifera	Moseley, 1881													X
Caryophyllia (C.) profunda	Moseley, 1881					x						X		
Caryophyllia (C.) quadragenaria	Alcock, 1902a													X
Caryophyllia (C.) rugosa	Moseley, 1881											X		
*^7.^ Caryophyllia(C.)sarsiae*	Zibrowius, 1974b													X
*^8.^ Caryophyllia(C.)scobinosa*	Alcock, 1902a													X
Caryophyllia (C.) stellula	Cairns, 1998				X									
Caryophyllia (C.) valdiviae	Zibrowius & Gili, 1990									X	X			
*^9.^ Crispatotrochuscornu*	(Moseley, 1881)													X
*Desmophyllumdianthus*	(Esper, 1794)				X						X	X		
*Desmophyllumpertusum*	(Linnaeus, 1758)										X			X
*Goniocorelladumosa*	(Alcock, 1902c)											X		
*Heterocyathusaequicostatus*	Milne–Edwards & Haime, 1848a				X									
*Heterocyathusalternatus*	Verrill, 1865									X				
*Heterocyathusmonileseptatum* sp. nov.														X
*Heterocyathussulcatus*	(Verrill, 1866)									X				
*Labyrinthocyathusdelicates*	(von Marenzeller, 1904)				X	X				X		X		
*Monohedotrochuscapensis* comb. nov.	(Gardiner, 1904)				X					X				X
*^10.^Polycyathus* sp														X
*Rhizosmiliarobusta*	Cairns in Cairns & Keller, 1993											X		
*Solenosmiliavariabilis*	Duncan, 1873					X						X		
Stephanocyathus (Acinocyathus) explanans	(von Marenzeller, 1904a)									X		X		
*^11.^Stephanocyathus (Odontocyathus) campaniformis*	(von Marenzeller, 1904a)													X
*^12.^Stephanocyathus (Odontocyathus) nobilis*	(Moseley, 1873)													X
*^13.^ Tethocyathusvirgatus*	(Alcock, 1902a)													X
Trochocyathus (T.) sp. 1	Gardiner,1904				X					X				X
Trochocyathus (T.) sp. 2														X
Trochocyathus (T.) sp. 3					X									X
Trochocyathus (T.) sp. cf. rawsonii sensu Cairns in Cairns and Keller 1993												X		X
*^14.^ Vaughanellaconcinna*	Gravier, 1915													X
*Deltocyathusitalicus*	(Michelotti, 1838)										X			
*Deltocyathusrotulus*	(Alcock, 1898)											X		
*Atlantiadenticulata* sp. nov.														X
Balanophyllia (B.) bonaespei	van der Horst, 1938								X					
Balanophyllia (B.) capensis	Verrill, 1865								X	X				
Balanophyllia (B.) diademata	van der Horst, 1927						X							
Balanophyllia (B.) diffusa	Harrison & Poole, 1909											X		
^15.^Balanophyllia (B.) sp. cf. malouinensis	Squires, 1961													X
Balanophyllia (B.) vanderhorsti	Cairns, 2001											X		
Balanophyllia (E.) stimpsonii	(Verrill, 1865)											X		
*Dendrophylliaarbuscula*	van der Horst, 1922											X		
*Dendrophylliacladonia*	van der Horst, 1927						X					X		
*Dendrophylliacornigera*	(Lamarck, 1816)					x	X							
*Dendrophylliadilatata*	van der Horst, 1927						X					X		
*Dendrophylliaijimai*	Yabe & Eguchi, 1934									X		X		
*Dendrophyllia* sp. 1														X
*Ednapsammiacolumnapriva* sp. nov.														X
*^16.^ Enallopsammiapusilla*	(Alcock, 1902a)													X
*^17.^ Enallopsammiarostrata*	(Pourtalès, 1878)													X
*Endopachysgrayi*	Milne–Edwards & Hamie, 1848b						X			X		X		
*^18.^ Endopsammiaphilippensis*	Milne–Edwards & Haime, 1848b									X				X
*Heteropsammiacochlea*	(Spengler, 1781)									X				
*^19.^ Heteropsammiaeupsammides*	(Gray, 1849)													X
*Pourtalopsammiatogata*	(van der Horst, 1927)						X					X		
*Rhizopsammiaannae*	(van der Horst, 1933)							X		X		X		
*Rhizopsammiacompacta*	Sheppard & Sheppard, 1991									X		X		
*^20.^ Rhizopsammiaverilli*	van der Horst, 1922													X
Tubastraea sp. cf. diaphana	(Dana, 1846)											X		X
*Tubastraeacoccinea*	Lesson, 1829									X				
*Tubastraeamicranthus*	(Ehrenberg, 1834)									X				
Flabellum (F.) leptoconus	Cairns & Zibrowius, 2016				X					X			X	
Flabellum (F.) pavoninum	Lesson, 1831		X		X					X		X		
*^21.^ Flabellum(F.)politum*	Cairns, 1989a													X
Flabellum (U.) alabastrum	Moseley, 1873										X			
^22.^Flabellum(U.)lowekeyesi*	Squires & Ralph, 1965													X
*^23.^ Javaniaantarctica*	(Gravier, 1914)													X
*Javaniainsignis*	Duncan, 1876											X		
*Placotrochidesscaphula*	Alcock, 1902c											X		
*Rhizotrochustypus*	Milne–Edwards & Haime, 1848a		X	X						X				
*Truncatoflabellumformosum*	Cairns, 1989a											X		
*Truncatoflabellumgardineri*	Cairns in Cairns & Keller, 1993											X		
*Truncatoflabelluminconstans*	(von Marenzeller, 1904a)					x				X				
*^24.^ Truncatoflabellummultispinosum*	Cairns in Cairns & Keller, 1993													X
*Truncatoflabellumpusillum*	Cairns, 1989a											X		
*Truncatoflabellum* sp.														X
*Truncatoflabellumzuluense*	Cairns in Cairns and Keller, 1993									X		X		
Fungiacyathus (B.) hydra	Zibrowius & Gili, 1990										X			
Fungiacyathus (B.) sibogae	(Alcock, 1902a)											X		
Fungiacyathus (F.) stephanus	(Alcock, 1893)											X		
Fungiacyathus (F.) sp												X		
*Guyniaannulata*	Duncan, 1872											X		
*Letepsammiaformosissima*	(Moseley, 1876)											X		
*Letepsammiafranki*	Owens, 1994						X			X				
*^25.^ Rhombopsammianiphada*	Owens, 1986a													X
*Stephanophylliafungulus*	Alcock, 1902b											X		
*Madreporaoculata*	Linnaeus, 1758													
*Culiciaexcavata*	(Milne–Edwards & Haime, 1849)									X				
*^26.^Culicia* sp. cf. australiensis	Hoffmeister, 1933													
*Culicia* sp. cf. *tenellanatalensis*	Dana, 1846	X										X		
*^27.^ Stenocyathusvermiformis*	(Pourtalès, 1868)													X
*^28.^ Cyathotrochuspileus*	(Alcock, 1902a)													X
*Deltocyathoidesorientalis*	(Duncan, 1876)									X		X		
*Deltocyathoidessentus*	Kitahara & Cairns, 2021											X		
Sphenotrochus (E.) gilchristi	Gardiner, 1904				X					X		X		
Sphenotrochus (S.) aurantiacus	von Marenzeller, 1904a					X				X		X		
Sphenotrochus (S.) evexicostatus	Cairns in Cairns & Keller, 1993									X		X		
Sphenotrochus (S.) imbricaticostatus	Cairns in Cairns & Keller, 1993									X		X		
*Tropidocyathuslessonii*	(Michelin, 1842)											X		

Prior to this, just one rare solitary form (*Culicianatalensis* Dana, 1846) was reported by Duncan in 1876 (Table 1). Subsequently, Gardiner’s (1902a, b, 1904) research significantly improved the knowledge base of the South African fauna and he reported 16 species (four representing new species). Gardiner’s (1902a, b, 1904) flabellids and caryophylliid contribution was then later complemented by von Marenzeller 1904a reported six species (two of which were new to science). Following this, van der Horst (1927, 1933, 1938) documented ten dendrophylliid species. Both the Gardiner and van der Horst accounts are mainly based on specimens collected through the University of Cape Town Ecological Surveys (UCTES) and remain the foundation of azooxanthellate coral research in South Africa. Five decades later, Boshoff (1981) published a checklist on 54 azooxanthellate scleractinian corals from the south-west Indian Ocean. Subsequent authors (Zibrowius and Gili 1990; Cairns and Keller 1993) highlighted the need for the re-analysis of Boshoff’s account. Other papers which include South African records are biogeographic reviews such as Zibrowius and Gili’s (1990) south-east Atlantic paper documenting five species, Cairns and Keller’s (1993) south-west Indian Ocean review that accounts for 45 species, and Cairns and Zibrowius’s (2016) new flabelliid species (Flabellum (Flabellum) leptoconus Cairns & Zibrowius’s, 2016). Since then, numerous specimens have remained unidentified in the Iziko Museum of South Africa collection. Moreover, additional specimens are being collected by offshore research collaborative programs involving the Department of Environment, Fisheries and Forestry (**DEFF**), the African Coelacanth and Ecosystem Program (**ACEP**), the South African Environmental Observation Network (**SAEON**), and the Ecosystem Approach to Fisheries (**EAF**)-Nansen Programme. Here we present the first faunistic review of South African azooxanthellate scleractinian corals, increasing the number of known species from 77 to 108 (Table 1).

## Materials and methods

This study is primarily based on the azooxanthellate Scleractinia deposited at the Iziko South African Museum (Cape Town) and at the National Museum of Natural History (Smithsonian, Washington DC), comprising more than 600 samples collected through six historical expeditions (RV ‘Anton Bruun’, Benguela IV, RV ‘Meiring Naude’, RV ‘Pieter Faure’, ‘Sardinops’, and University of Cape Town Ecological Surveys). Other collections considered include 38 samples from the Boshoff Collection housed at the Oceanographic Research Institute (Durban), 71 samples from the African Coelacanth Ecosystem Programme (Deep-Secrets and Imida surveys), 31 samples from the Department of Environment, Forestry and Fisheries/South African Environmental Observation Network demersal surveys, ten samples collected on the 2018 Nansen survey, and five from the Department of Environment, Forestry and Fisheries offshore benthic surveys. Overall, these samples comprise ca. 3100 specimens, all of which originated within the South African territorial sea and Economic Exclusive Zone (Suppl. material 1), and covering a depth range from the intertidal zone to 1,420 m depth. Although the Prince Edwards Islands constitute the South African territory, samples from this locality were not considered in the current study owing to the region exhibiting distinctive oceanographic patterns as compared with the surroundings of mainland South African surroundings. Therefore, the Prince Edwards Islands fauna will be reviewed subsequently.

Morphological descriptions follow the terminology used by Cairns (1989a, b, 1997, 2001), and Cairns and Kitahara (2012). Diagnoses for genera were amended from existing literature (Cairns 2001; Cairns and Kitahara 2012). Species are presented in alphabetical order according to family, genus, and species. Each species entry is followed by a synonym list, type locality, and type material (if known). The section on material examined provides the catalogue number or a sample identifier term (arranged in chronological order), followed by the number of specimens (in brackets), sampling location and depth. Both catalogue and sample identifier numbers are presented as written on the labels (e.g., BMNH #). Previously reported South African records are highlighted in bold. Furthermore, refined regional localities are given in which records with coordinate information (Suppl. material 2: Fig. S1) follow a hierarchical approach that gives distance of coral records to the closest gazetted coastal town (in uppercase) and estuary system (Suppl. material 2: Figs S2–5). The methodology undertaken to standardize place names caters for international and national/indigenous communities, whilst locality given on the catalogue label is presented for records without coordinate information. Species descriptions are based mostly, but not solely, on the South African material examined. Imagery data of some South African specimens, shared by Dr Helmut Zibrowius, were also considered and presented. Although these specimens may represent misplaced records as the specimens with Iziko accession numbers were not traceable in the museum’s collection (Suppl. material 1). For all species, following their morphological descriptions, the local and global distribution is provided with a depth range. South African (local) distribution are presented, in lower cases, within the following regions: western margin – from the Namibian border to Cape Point; the southern margin – eastwards from Cape Point to the Mbashe River; and the eastern margin – the region extending from the Mbashe River to the Mozambique border. A locality range is also presented in this section and limited to the closest coastal town. Remarks containing morphological comparisons and, when pertinent, a brief history of the taxonomy of the species in South Africa, is provided. Furthermore, new knowledge on the regional distribution is also added in the remarks section, where applicable. Finally, images from most species identified are presented.

### List of abbreviations

#### Museums and collection institutes

**BMNH** British Museum of Natural History London (now NHMUK);

**IM**Indian Museum, Calcutta;

**IO** Institute of Oceanology, Moscow;

**MCZ** Museum of Comparative Zoology, Harvard University, Cambridge;

**MNHN**Muséum national d’Histoire naturelle, Paris;

**MOM**Muséum Océanographique de Monaco, Monaco;

**MoNZ**Museum of New Zealand Te Papa Tongarewa, Wellington;

**NHMUK**Museum of Natural History London;

**NMNH**National Museum of Natural History, Smithsonian, Washington DC;

**ORI** Oceanographic Research Institute, Durban;

**SAMC**South African Museum, Cape Town;

**SAMH** South African Museum Hydroids;

**WAM**Western Australian Museum, Perth;

**YPM** Yale Peabody Museum, New Haven;

**ZMA**Zöologisch Museum, Amsterdam;

**ZMB**Zoologisches Museum, Berlin.

#### Expeditions and institutions

**AB** RV ‘Anton Bruun’;

**ACEP** African Coelacanth Ecosystem Programme;

**CCS** Cape Canyon Survey (Department of Environment, Forestry and Fisheries: DEFF);

**DSC** Deep Secrets Cruise (African Coelacanth Ecosystem Programme: ACEP);

**DTE** Deutschen Tiefsee-Expedition;

**MN** RV ‘Meiring Naude’;

**PF** RV ‘Pieter Faure’;

**SAEON** South African Environmental Observation Network;

**SVMEC** Southern margin Vulnerable Marine Ecosystem Cruise (Department of Environment, Forestry and Fisheries: DEFF);

**UCTES** University of Cape Town Ecological Survey.

#### Morphological terms

**BD** Basal diameter;

**GCD:H** Ratio of greater calicular diameter to height of a solitary corallum;

**GCD:LCD** Ratio of greater calicular diameter to lesser calicular diameter;

**H:D** Ratio of height to diameter of a solitary corallum;

**LCD** Lesser calicular diameter;

**PD** Pedicel diameter;

**GPD** Greater pedicel diameter;

**PD:GCD** Ratio of pedicel diameter to greater calicular diameter of a solitary corallum;

**S_x_, C_x_, P_x_** Septa, costae, or pali (respectively) of cycle designated by the number;

**S_x_ > S_y_** Septa of cycle × wider than those of cycle y’

## Systematic account

### Order Scleractinia

#### Family Caryophylliidae Dana, 1846

##### Anomocora

Taxon classificationAnimaliaScleractiniaCaryophylliidae

Studer, 1877

6B155C47-2CC6-574C-A8B1-6645EB224CA0

###### Diagnosis.

Solitary, subcylindrical, free. Tendency to bud new coralla from margin zone with subsequent loss of organic connection. Wall thin. Columella trabecular, no pali.

###### Type species.

*Coelosmiliafecunda* Pourtalès, 1871, by monotypy.

##### Anomocora fecunda

Taxon classificationAnimaliaScleractiniaCaryophylliidae

(Pourtalès, 1871)

2DD4AF37-B2A1-554D-B442-C7B602613E98

Fig. 1A, B

Coelosmilia fecunda Pourtalès, 1871: 21–22 (in part: pl. 1, fig. 12, pl. 6, figs 14–15).
Coenosmilia fecunda . –Zibrowius 1980: 131–133 (in part: pl. 67, figs A–K).
Parasmilia fecunda . –Gardiner and Waugh 1939: 229.
Anomocora fecunda . –Cairns 1979: 127–129, pl. 24, figs 6–8, Map 35. –Cairns 2000: 128. –Reyes et al. 2009: 25–26, fig. 4L, M. –Kitahara and Cairns 2021: 452–454, figs 244E–G, 245.

###### Type locality.

Off Southern Straits, Florida; 124–576 m (Cairns 1979).

###### Type material.

Six syntypes are deposited at the MCZ (Cairns 1979).

###### Material examined.

SAMC_A073042 (1 specimen): 53 km from Shaka’s Rock/46 km off Zinkwasi Estuary, 29°32'53.88"S, 31°47'12.11"E; 200 m.

###### Description.

Corallum cylindrical, straight to gently curved, tapering towards a broken base. Axial corallite of examined specimen measures 8.1 mm in CD and 48.9 mm in H, and bears 18 secondary corallites. Secondary corallites bud irregularly, usually perpendicular to axial corallite. Calices circular to slightly elliptical (GCD:LCD = 1.00–1.1), calicular margin slightly serrated. Specimen examined has two scars of former buds on theca, with secondary corallites rarely exceeding 5 mm in CD. Costae well developed, particularly at calicular margin, corresponding with septa in size. C_1_ most prominent, higher costae progressively smaller. Intercostal striae shallow and narrow. Theca thin. Corallum white, with C_1–2_ having light greenish brown tints.

Septa hexamerally arranged in four cycles, the last cycle being incomplete, according to formula: S_1_ > S_2_ > S_3_ > S_4_ (46 septa). S_1_ most exsert, reaching columella with straight and vertical axial margins. S_2_ less exsert and half the width of S_1,_ also with straight axial margins bearing slender ribbons. S_3_ not exsert, ^2^/_3_ the size of S_2,_ also bearing ribbons that intermingle with those of S_2._ S_4_ rudimentary, with sinuous to slightly dentate axial margins. Septa mostly smooth, with growth lines along septal margin, and faint granulation perpendicular to septal margin. Fossa deep, with poorly developed trabecular columella, which is indistinguishable from S_2_ and S_3_ intermingled slender ribbons.

###### Distribution.

Regional: Eastern margin of South Africa, off Shaka’s Rock; 200 m. Elsewhere: Bahamas; Caribbean (Reyes et al. 2009); eastern Gulf of Mexico; northeastern Brazil (off Maranhão); St. Peter and St. Paul Rocks (Cairns 1979); Azores; Madeira; Canary Islands (Zibrowius 1980); New Caledonia (Kitahara and Cairns 2021); 37–640 m.

###### Remarks.

According to Cairns (1979), *Anomorocafecunda* closely resembles *Coenosmiliaarbuscula* Pourtalès, 1871, but differs in having a more elongated corallum with thin costae forming continuous ridges. Further differences are highlighted in budding: in *A.fecunda* buds appear randomly and detach from the theca of parent corallum before the third generation appears. However, in *C.arbuscula*, new buds appear equally spaced around the calicular perimeter and remain firmly attached, resulting in a small bushy colony of which the main corallite gives rise to five successive generations. Differences in columella are also noted between the two species, with *A.fecunda* having a faint trabecular columella and *C.arbuscula* a well-developed papillose columella. Examined specimen represents a new record to the southwest Indian Ocean.

**Figure 1.**
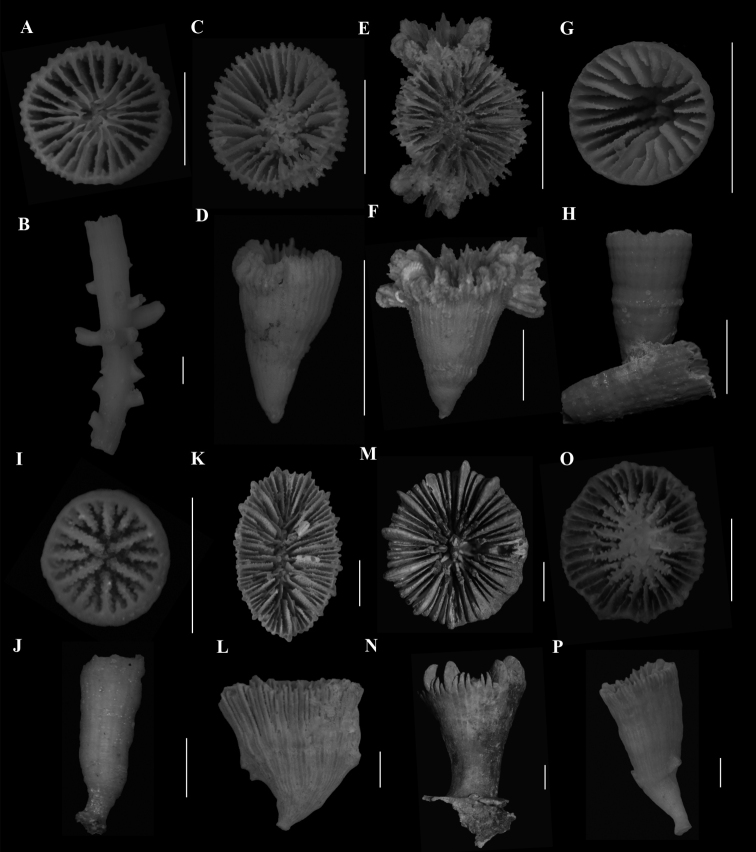
**A**, **B***Anomocorafecunda* (SAMC_A073042, off Shaka’s Rock, 200 m) **A** calicular view **B** lateral view **C**, **F***Anomocoramarchadi***C**, **D** (SAMC_A090093, off Durban, 49 m) **C** calicular view **D** lateral view **E**, **F** (SAM_H3100, off Shaka’s Rock, 66 m) **E** calicular view **F** lateral view **G**, **H***Aulocyathusjuvenescens* (Mortensen25, off Durban, 424 m) **G** calicular view **H** lateral view **I**, **J**Aulocyathus sp. cf. matricidus (DIa1, locality data unknown) **I** calicular view **J** lateral view **K**, **L**Caryophyllia (Acanthocyathus) grayi (USNM 91541, off Kosi Bay, 98 m) **K** calicular view **L** lateral view **M**, **N**Caryophyllia (Caryophyllia) diomedeae (MN_SM129, off Margate, 850 m) **M** calicular view **N** lateral view **N**, **O**Caryophyllia (Caryophyllia) ephyala (SAMC_A072974, off Paternoster, 440 m) **O** Calicular view **P** Lateral view. Scale bars: 10 mm.

##### Anomocora marchadi

Taxon classificationAnimaliaScleractiniaCaryophylliidae

(Chevalier, 1966)

FC242EC4-941F-5E38-8D84-554BD1F5624B

Fig. 1C–F

Ceratotrochus johnsoni . –Gardiner 1904: 118–119, pl. 1, figs 5A–C, pl. 2, fig. M. –Gardiner and Waugh 1938: 188.
Dasmosmilia marchadi Chevalier, 1966: 944–949, pl. 5, figs 3, 4.
Asterosmilia marchadi . –Cairns and Keller 1993: 249, fig. 6A, B.
Balanophyllia capensis . –Boshoff 1981: 40 (in part).
Anomocora marchadi . –Cairns 2000: 130–131. – Cairns 2004: 276. –Reyes et al. 2009: 26, 27, fig. 4A–Q.

###### Type locality.

Off Cape Verde, Senegal (RV ‘Gerard Tréca’ stn 18–2–1954); 97–98 m (Chevalier 1966).

###### Type material.

The holotype is deposited at the MNHN (Cairns 1979).

###### Material examined.

SAMC_A090093 (1 specimen): Eastern margin, 14 km off Durban/12 km Mbokodweni Estuary, 29°58'00.00"S, 31°01'59.99"E; 49 m. SAM_H1683 (1 specimen): Western margin, 22 km off Cape Town/13 km off Elsies Estuary, 34°04'59.99"S, 18°19'59.99"E; depth unknown. SAM_H3098 (2 specimens): Eastern margin, 6 km off Durban/9 km off Umgeni Estuary, 29°52'59.99"S, 31°03'05.00"E; 86 m. SAM_H3099 (7 specimens): Eastern margin, 19 km from Shaka’s Rock/3 km off Mdloti Estuary, 29°38'59.99"S, 31°07'59.99"E; 71–73 m. SAM_H3100 (1 specimen): Eastern margin, 9 km from Shaka’s Rock/2 km off Tongati Estuary, 29°34'00.00"S, 31°10'59.99"E; 66 m. ORI_EIa4 (1 specimen): no locality data. USNM 91561 (1 specimen): Eastern margin, 41 km south of Ponta Do Ouro/26 km off Kosi Bay Estuary, 27°13'03.60"S, 32°49'18.00"E; 60 m.

###### Imagery data.

**BMNH 1950.1.10.118 (2 specimens)**, BMNH1950.3.22.13 (2 specimens): no locality data. SAM_H1456 (1 specimen): Eastern margin, 9 km from Shaka’s Rock/2 km off Tongati Estuary, 29°34'00.00"S, 31°10'59.99"E; 66 m. SAM_H2806 (1 specimen): no locality data. MN_ZH17 (1 specimen): Eastern margin, 59 km off Cape Vidal/10 km of Mgobezeleni Estuary, 27°37'00.00"S, 32°40'54.00"E; 65–70 m.

###### Description.

Corallum solitary, ceratoid, tapering to a free pointed base. Axial corallite with ≤ two secondary corallites. Corallites bud from margin zone of parent corallum. Budding extra-tentacular. Calices round to slightly elliptical (GCD:LCD = 1.0–1.1), with serrated calicular margin. Largest specimen examined (SAMC_A90093) 9.8 × 8.9 mm in CD and 16.1 mm in H. Costae prominent and unequal in width. C_1–2_ wider than C_3–4_. All costae granulated and separated by broad intercostal striae extending from calicular margin to base. Corallum white to reddish brown.

Septa hexamerally arranged in four cycles according to formula: S_1_ > S_2_ > S_3_ > S_4_ (48 septa). S_1_ highly exsert, with straight axial margin that fuse to columella deep in fossa. S_2_ slightly less wide and less exsert than S_1_, but otherwise similar in profile. S_3_ ½ width and less exsert than S_2_, with dentate axial margin, each bearing a paliform lobe (P_3_). In each system, a pair of P_3_ fuse before S_2_ near columella. S_4_ as exsert as S_3,_ but rudimentary in development. Septal faces bear granules arranged perpendicular to septa margin. Fossa of moderate depth, containing a crispate columella which is usually indistinguishable to pali.

###### Distribution.

Regional: Western and eastern margin of South Africa, from Cape Town towards the Kosi Bay estuary, (41 km south of Ponta Do Ouro: Mozambique); 49–86 m. Elsewhere: off Pensacola; Florida; southern Caribbean from Colombia to Peninsula de Paria Venezuela (Cairns 1979; Reyes et al. 2009); from Spanish Sahara to Gabon (Zibrowius 1980); Philippines; Indonesia (Cairns and Zibrowius 1997); South China Sea; Mozambique; off Pemba; Tanzania; Maldives (Gardiner and Waugh 1938; Cairns and Keller 1993); 32–229 m.

###### Remarks.

*Anomocoramarchadi* differs from *A.prolifera* (Pourtalès, 1871) in having P_3_ fusing before S_2_ near the columella (Cairns 1979). However, *A.prolifera* has not yet been reported from South Africa, thus *A.marchadi* differs from the only other species reported in the region (*A.fecunda*), in having paliform lobes indistinguishable from the columellar elements. *Anomocoramarchadi* was first reported from South Africa by Gardiner (1904), off Cape Natal. Subsequently, Boshoff (1981) incorrectly identified this species as *Balanophylliacapensis*. Nonetheless, material examined herein extends the known South African distribution of this species further north towards Durban.

##### Aulocyathus

Taxon classificationAnimaliaScleractiniaCaryophylliidae

von Marenzeller, 1904a

120935E4-E6E4-5547-BB29-3611CE743A45

###### Diagnosis.

Corallum solitary, ceratoid, and free. Evidence of budding from a longitudinally fragmented of the parent corallum common. Costae poorly defined. Upper, distal septal margins join theca below upper thecal margin usually forming a calicular thecal rim. Slender paliform lobes occasionally present before S_1–3_. Columella trabecular.

###### Type species.

*Aulocyathusjuvenescens* von Marenzeller, 1904a, by monotypy.

##### Aulocyathus juvenescens

Taxon classificationAnimaliaScleractiniaCaryophylliidae

von Marenzeller, 1904a

D2547411-0B7D-508A-A33B-FD791EB07143

Fig. 1G, H

Aulocyathus juvenescens von Marenzeller, 1904a: 301–302, pl. 18, fig. 17. –Zibrowius 1980: 107. –Cairns and Keller 1993: 247. –Cairns 1994: pl. 26, figs H, I. –Cairns and Zibrowius 1997: 130. –Cairns 1999a: 104, fig. 15H. –Kitahara and Cairns 2021: 409–411, figs 221, 222G–I.

###### Type locality.

Off Pemba and Zanzibar Island, Tanzania (SS ‘Valdivia’ stn. 243 and 245: 6°39'1"S, 39°30'8"E, 5°27'9"S, 39°18'8"E, respectively); 400–463 m (von Marenzeller 1904a).

###### Type material.

Syntypes are deposited at the ZMB (Cairns and Keller 1993).

###### Material examined.

None.

###### Imagery data.

‘Galathea Expedition’ stn. 196 (2 specimens): Eastern margin, 33 km off Durban/31 km off Beachwood Mangroves, 29°55'00.00"S, 31°19'59.99"E; 425–430 m. ‘Mortensen-Java Expedition’ stn. 25 (1 specimen): Eastern margin, off Durban, 424 m.

###### Description.

Corallum solitary, ceratoid, and usually attached to a fragment of the parent corallum. Calice circular, with finely serrated calicular margin. Largest imaged specimen (*Galathea Expedition* stn. 196) 6.5 × 6.5 mm in CD, and ≤ 11.0 mm in H. Costae restricted to calicular margin, with low intercostal striae. Theca smooth and porcelaneous. Corallum white, with longitudinal light brown pigmentation.

Septa hexamerally arranged in four cycles, the last cycle being incomplete, according to the formula: S_1_ > S_2_ > S_3_ > S_4_ (30 septa). S_1_ most exsert septal cycle. S_2_ slightly less exsert than S_1_, being ^3^/_4_ the width of S_1._ S_3–4_ progressively less exsert. S_3_ dimorphic in size: those in half systems lacking S_4_ only ½ the width of S_2_, and those flanked by a pair of S_4_ attain the same width as S_2._ If present, S_4_ ½ the size of S_3._ All septa with vertical and slightly sinuous axial margin, S_3–4_ bearing dentate axial margins deeper on fossa. All septal faces with fine granulations. Fossa deep containing a rudimentary columella.

###### Distribution.

Regional: Eastern margin of South Africa, off Durban; 424–430 m. Elsewhere: Vanuatu; Philippines (Cairns 1999a); New Caledonia (Kitahara and Cairns 2021); Tanzania (von Marenzeller 1904a; Cairns and Keller 1993); 182–790 m.

###### Remarks.

The imagery records of *Aulocyathusjuvenescens* represent a distributional range extension for this species further south of Tanzania and are also new records for South Africa. Although these specimens have no more than 30 septa, their morphology is consistent with the taxonomic diagnosis of the species detailed by von Marenzeller (1904a). Amongst the four recent species of the genus, *A.juvenescens* has the smallest CD, least number of septa, and a porcelaneous theca (Cairns 1999a).

##### Aulocyathus sp. cf. matricidus

Taxon classificationAnimaliaScleractiniaCaryophylliidae

(Kent, 1871)

E3AB432E-1590-5EDE-A540-4F50A5EE8420

Fig. 1I, J

**Figure 2.**
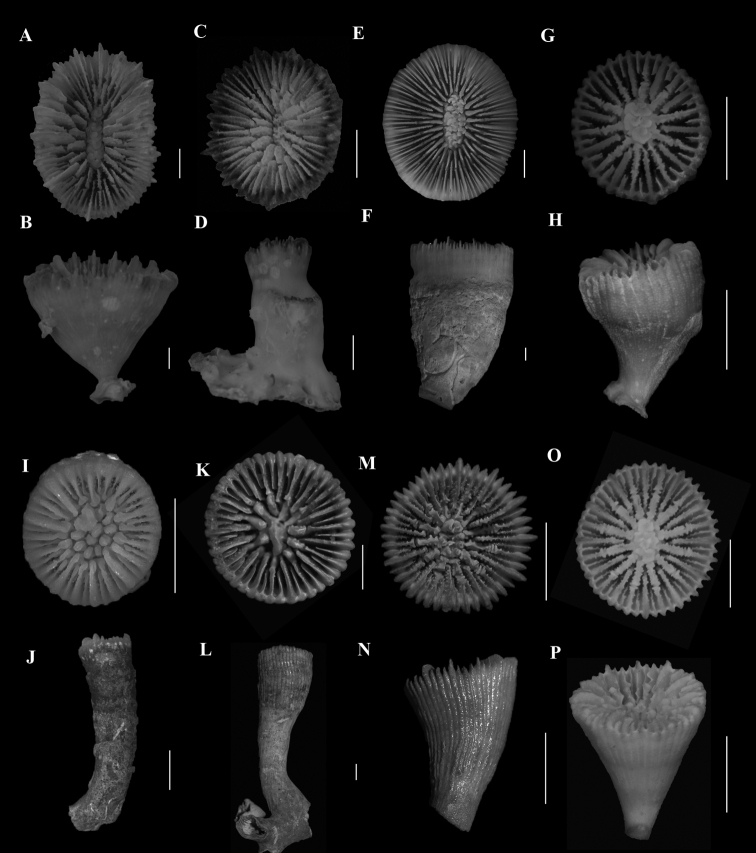
**A**, **B**Caryophyllia (Caryophyllia) grandis (SAMC_A073150, off Mazeppa Bay, 480–490m) **A** calicular view **B** lateral view **C**, **D**Caryophyllia (Caryophyllia) lamellifera (SAMC_A090155, off Sodwana Bay, 120 m) **C** calicular view **D** lateral view **E**, **F**Caryophyllia (Caryophyllia) profunda (DTEValdivia Stn 108, off Agulhas, 126 m) **E** calicular view **F** lateral view **G**, **H**Caryophyllia (Caryophyllia) quadragenaria (USNM 91539, Eastern coast, off Shaka’s Rock, 350 m) **G** calicular view **H** lateral view **I**, **J**Caryophyllia (Caryophyllia) rugosa (SAMC_A090071, off Kosi-Kumpungwini (Sifungwe) Estuary, 370 m) **I** calicular view **J** lateral view **K**, **N**Caryophyllia (Caryophyllia) sarsiae**K**, **L** (MN_SM226, off Mazeppa Bay, 710–775 m) **K** calicular view **L** lateral view **M**, **N** (MN_SM174, off Kidds Beach, 760 m) **M** calicular view **N** lateral view **O**, **P**Caryophyllia (Caryophyllia) scobinosa (SAM_H1248, off Durban, 91m) **O** calicular view **P** lateral view. Scale bars: 10 mm.

Flabellum matricidum Kent, 1871: 276, pl. 23: fig. 2A–C.
Fragilocyathus conotrochoides Yabe & Eguchi, 1932a: 388, 389, fig. 1. –Yabe and Eguchi 1941a: 101. –Yabe and Eguchi 1942b: 116, 145. pl. 9: fig. 15. –Eguchi 1965a: 288, 4 figs. –Eguchi and Miyawaki 1975: 57.
Aulocyathus cf. matricidus . –Yabe and Eguchi 1942b: 112, 116.
Aulocyathus matricidus . –Cairns 1999a: 104. –Cairns 1994: 60, pl. 26, figs C–G, pl. 42, figs B–D.

###### Type locality.

Off Japan, 84 m (Zibrowius 1980; Cairns 1994).

###### Type material.

Two syntypes are deposited at the NHM (Cairns 1994).

###### Material examined.

ORI_DIa1 (2 specimens): no locality data.

###### Description.

Corallum solitary, attached, and conical to elongate. Calice circular, calicular margin smooth. Largest of two specimens examined (ORI_DIa1) 5.2 × 5.1 mm in CD, and ≤ 15.4 mm in H. Costae wide. Theca and costae granular. Corallum light brown.

Septa hexamerally arranged in four cycles, the last cycle being incomplete, according to the formula: S_1_ > S_2_ > S_3_ > S_4_ (32 septa). S_1–2_ most exsert septa. S_1_ extend almost towards centre of fossa, with vertical to slightly sinuous axial margin. S_2_ ¼ less wide than S_1,_ also has vertical axial margin, and being granular deeper in fossa. S_3–4_ progressively less exsert (if at all). S_3_ dimorphic in size: those half-systems lacking S_4_ only ½ the width of S_2,_ while those flanked by S_4_ attain almost the same width of S_2._ If present, S_4_ ½ the width of S_3._ Axial margins of S_3–4_ dentate. Septal faces finely granular. Fossa deep containing a rudimentary columella.

###### Distribution.

Regional: Eastern margin of South Africa; depth unknown. Elsewhere: Tsugara Strait; and Japan Sea (Cairns 1994); 84–207 m.

###### Remarks.

Among all congeners, specimens examined closely resemble *Aulocyathusmatricidus* in having a smooth calicular margin, upper peripheral septa not notched, rudimentary columella, and a slender pedicel. However, *A.matricidus* is only known from Japan and, therefore, its occurrence in the southwestern Indian Ocean would represent a disjunct distribution.

##### Caryophyllia

Taxon classificationAnimaliaScleractiniaCaryophylliidae

 Lamarck, 1816

62DECBA0-3A91-5D97-B3B9-128930A5759B

###### Diagnosis.

Corallum solitary, attached or free: if attached, corallum cylindrical, trochoid, or ceratoid; if free, corallum usually cornute. Calice circular, elliptical, or compressed; thecal margin spines present on species having compressed coralla. Septal symmetry variable, but hexameral symmetry with four cycles of septa most common. One crown of paliform lobes present before penultimate or rarely the antepenultimate cycle of septa. Columella fascicular, composed of several twisted laths. Exclusively azooxanthellate and common in deep water.

##### Caryophyllia (Acanthocyathus)

Taxon classificationAnimaliaScleractiniaCaryophylliidae

Milne-Edwards & Haime, 1848

B76AB481-B046-515F-A092-AB6D9DE025F7

###### Diagnosis.

*Caryophyllia* with thecal margin spines or crests.

###### Type species.

*Acanthocyathusgrayi* Milne-Edwards & Haime, 1848a, by subsequent designation (Milne-Edwards and Haime 1850b).

##### Caryophyllia (Acanthocyathus) dentata

Taxon classificationAnimaliaScleractiniaCaryophylliidae

(Moseley, 1881)

8527E938-A241-5A1D-B61A-3EB047FE90EA

Acanthocyathus sp. –Moseley 1876: 550.
Acanthocyathus dentatus Moseley, 1881: 143, pl. 2, fig. 7A–C.
Caryophyllia (Acanthocyathus) dentata . –Cairns and Zibrowius 1997: 98–99, fig. 8A–D. –Kitahara et al. 2010: 92, 112.

###### Type locality.

Off Kandavu Islands, Fiji (HMS ‘Challenger’ stn. 174D: 19°05'50"S, 178°16'20"E); 384 m (Moseley 1881).

###### Type material.

The holotype is deposited at the NHMUK (Kitahara et al. 2010).

###### Material examined.

**USNM 91540** (1 damaged specimen): Eastern margin, 23 km from Shaka’s Rock/off Tongati Estuary, 29°41'14.39"S, 31°21'10.80"E; 85 m.

###### Distribution.

Regional: Eastern margin of South Africa, off Shaka’s Rock; 85 m. Elsewhere: Fiji (Moseley 1881); and Indonesia (Cairns and Zibrowius 1997); 90–384 m.

###### Remarks.

The specimen reported herein is severely damaged and therefore tentatively added to the South African coral fauna- thus representing a new record for the region. Nonetheless, representatives from Indonesia have been well described by Cairns and Zibrowius (1997), who noted the discrepancies in the septal symmetry between the holotype (hexameral) and the specimens they examined (decameral). Apart from the symmetry, the same authors also pointed out a distinctively ridged C_1–2_ of their studied specimens in relation to the holotype.

##### Caryophyllia (Acanthocyathus) grayi

Taxon classificationAnimaliaScleractiniaCaryophylliidae

(Moseley, 1881)

1713B507-8A96-5367-B65A-7123ED1078D4

Fig. 1K, L

Acanthocyathus grayi Milne-Edwards & Haime, 1848a: 293, pl. 9, fig. 2. –Alcock 1898: 15. –van der Horst 1931: 6. –Umbgrove 1938: 264–265. –Umbgrove 1950: 641–642, pl. 81, figs 27–32. –Wells 1984: 209, pl. 2, figs 5–9. –Zou 1988: 76, figs 8–9.
Caryophyllia (Acanthocyathus) grayi . –Cairns 1994: 49, pl. 21, figs I–K. –Cairns and Zibrowius 1997: 97–98, figs 7C, F, I. –Cairns 1998: 377. –Cairns 1999a: 76. –Cairns 2004: 276.
Caryophyllia grayi . –Kitahara et al. 2010: 102, figs 53–55, 58–59.

###### Type locality.

Unknown (Cairns and Zibrowius 1997; Kitahara et al. 2010).

###### Type material.

One syntype is deposited at the NHMUK (Cairns and Zibrowius 1997; Kitahara et al. 2010).

###### Material examined.

**USNM 91541 (1 specimen)**: Eastern margin, 32 km south of Ponta Do Ouro/20 km off Kosi Bay Estuary, 27°08'10.79"S, 32°52'07.20"E; 98 m.

###### Description.

Corallum ceratoid, curved, unattached, with a slender and slightly curved pedicel (PD:GCD = 0.09). Calice compressed (GCD:LCD = 1.6). Calicular margin serrate. Only specimen examined: 19.9 × 12.4 mm in CD, 1.8 mm in PD, and 22.1 mm in H. Thecal margins rounded, with three thecal spines. Costae rounded, equal in width, and extending towards pedicel. C_1_ more prominent than remaining costae. Intercostal striae narrow. Examined specimen eroded with a light brown appearance.

Septa in 15 sectors arranged in four cycles, the last cycle being incomplete, according to the formula: 15:15:30:8 (68 septa). Primary septa most exsert, and extend ¾ distance to columella, with straight to slightly sinuous axial margin. Higher cycle septa less exsert (if at all). Secondary septa ¼ the width of primaries, with sinuous axial margins. Tertiary septa ¾ less the width of secondaries also having sinuous axial margins. S_4_ rudimentary. A total of 15 (P_3_) with sinuous axial margins encircle an elongated fascicular columellar. Fossa of moderate depth.

###### Distribution.

Regional: Eastern margin of South Africa, off Kosi Bay Estuary (32 km south of Ponta Do Ouro: Mozambique); 98 m. Elsewhere: Japan (Cairns 1994); Philippines; Indonesia (Cairns and Zibrowius 1997); Wallis and Futuna Islands; Vanuatu (Cairns 1999a); New Caledonia (Kitahara and Cairns 2021); Australia (Cairns 2004; Kitahara et al. 2010); Andamans Islands (van der Horst 1931); 37–490 m.

###### Remarks.

Caryophyllia (Acanthocyathus) grayi differs from *C.dentata*, the only other *Acanthocyathus* from the region, in having 15 primary septa and in bearing spines on both thecal edges only on one side in *C.dentata*. Although *C.grayi* was previously reported from South Africa (Cairns and Zibrowius 1997; Cairns 1999a; Kitahara et al. 2010), none of the authors presented the locality information. Nonetheless, South African representatives of *C.grayi* are similar to the Australian specimens in S_1_ bearing a sinuous axial margin, whereas they are both different from the Japanese specimens in that regard. However, this South African specimen differs from both the Japanese and Australian representatives in its septa arranged in 15 sectors, with four pairs of S_4_ (15:15:30:8); instead of 14 or 18 sectors.

##### Caryophyllia (Caryophyllia)

Taxon classificationAnimaliaScleractiniaCaryophylliidae

Lamarck, 1806

A1249918-2E21-56E8-A2A7-AF0DAFC9C5DB

###### Diagnosis.

*Caryophyllia* without thecal margin spines or crests.

###### Type species.

*Madreporacyathus* Ellis & Solander, 1786, by subsequent designation (Stokes and Broderip 1828).

##### Caryophyllia (Caryophyllia) cinticulata

Taxon classificationAnimaliaScleractiniaCaryophylliidae

(Alcock, 1898)

BB813AD6-D366-5494-9A2F-1B4C51A3EA8B

Thecocyathus cinticulatus Alcock, 1898: 17–18, pl. 2, figs 5, 5A.
Trochocyathus cinticulatus . –Gardiner 1904: 99, 103–104, pl. 2, fig. 2. –Cairns et al. 1999: 24.
Caryophyllia cinticulata . –Kitahara et al. 2010a: 98, 113, figs 17–21.
Caryophyllia (Caryophyllia) cinticulata . –Kitahara and Cairns 2021: 465–466, 468, figs 252, 253A–F.

###### Type locality.

Off the Maldives (HMS ‘Investigator’); 84 m (Alcock 1898; Kitahara et al. 2010).

###### Type material.

The holotype is purportedly deposited at the IM (Kitahara et al. 2010).

###### Material examined.

None.

###### Distribution.

Regional: Eastern margin of South Africa, off East London; 59 m (Gardiner 1904). Elsewhere: Maldives (Alcock 1898); and New Caledonia (Kitahara et al. 2010; Kitahara and Cairns 2021); 282–384 m.

###### Remarks.

*Caryophylliacinticulata* representatives from New Caledonia are well described by Kitahara et al. (2010), in which these authors mention that it displays circumferential transverse ridges on the theca, and differs from its congeners by having decameral symmetry, adults attaining > 10 mm in GCD, S_3_ smaller than S_2_, and extremely sinuous S_1_ and S_2_ axial margins. No specimens of this species were found in the material examined and therefore the entry is based on Gardiner’s (1904) record, who reported the species off East London.

##### Caryophyllia (Caryophyllia) diomedeae

Taxon classificationAnimaliaScleractiniaCaryophylliidae

von Marenzeller, 1904

BBA8A636-514C-545A-BA07-926AD0F6AB5E

Fig. 1M, N

Caryophyllia diomedeae von Marenzeller, 1904b: 79–80, pl. 1, fig. 2. –Durham and Barnard 1952: 10, 82, pl. 9, fig. 43. –Cairns 1991: 11–13, pl. 4, figs C–E. –Cairns 1995: 49–50, pl. 9, figs A–D. –Cortès 1997: 330. –Cairns and Zibrowius 1997: 88. –Koslow and Gowlett-Holmes 1998: 38. –Cairns 1999a: 74. –Cairns et al. 1999: 20. –Piñón 1999: 20, 81. –Cairns 2004: 264, 277, 328. –Cairns et al. 2005: 17, 25, 28, fig. 2D–E. –González-Romero et al. 2008: 1–2, fig. 1. –Kitahara et al. 2010: 100, 102, figs 37–46.
Caryophyllia profunda . –Cairns 1982: 17–19 (in part: ‘Eltanin’–1403).
Caryophyllia sarsiae . –Cairns and Parker 1992: 20, figs 5C, E, F.

###### Type locality.

Off Panama (USS ‘Albatross’ stn. 3358: 6°30'N, 81°44'W); 1043 m (von Marenzeller 1904b).

###### Type material.

One syntype is deposited at NHMUK (Kitahara et al. 2010).

###### Imagery data.

MN_SM 85 (1 specimen): Eastern margin, 20 km off Cape Vidal/23 km off St Lucia Estuary, 27°59'30.00"S, 32°40'47.99"E; 550 m. MN_SM 129 (7 specimens): Eastern margin, 17 km off Margate/ km off Boboyi Estuary, 30°53'24.00"S, 30°31'41.99"E; 850 m. MN_SM 226 (1 specimen): Southern margin, 32 km off Mazeppa Bay/24 km off Kobole Estuary, 32°28'36.00"S, 28°58'48.00"E; 710–775 m.

###### Description.

Corallum straight to slightly curved, and attached to substrate by a broad pedicel (PD:GCD ~ 0.5) that expands into an encrusting base. Largest imaged specimen (MN_SM 85) 13.0 × 12.0 mm in CD, 5.0 mm in PD, and 29.0 mm in H. Calice elliptical (GCD:LCD = 1.08–1.14), calicular margin jagged. Costae prominent at calicular margin, with shallow intercostal striae, sometimes bearing granules. Theca thick and porcelaneous. Corallum white to beige.

Septa hexamerally arranged in four cycles according to the formula: S_1_ ≥ S_2_ > S_3_ > S_4_ (48 septa). S_1–2_ most exsert, equal in width, and almost reach columella with vertical to slightly sinuous axial margins. S_3–4_ progressively narrower and less exsert. S_3_ ¾ the width of S_1–2_, each bearing a sinuous pali (12 P_3_), and consistently 1 mm in width. S_4_ slightly smaller than S_3._ Higher cycle septa with sinuous axial margin, especially S_3_. Septal faces somewhat smooth near calicular margin, but becoming granulated towards fossa. Fossa of moderate depth containing a fascicular columella, encircled by a P_3_ crown.

###### Distribution.

Regional: Southern and Eastern margins of South Africa, off Mazeppa Bay extending towards Cape Vidal; 550–850 m. Elsewhere: Australia (Cairns and Parker 1992, 1998, 2004a; Kitahara et al. 2010); New Zealand (Cairns 1982, 1995); New Caledonia (Kitahara et al. 2010; Kitahara and Cairns 2021); Cocos and Galapagos Islands (Cairns 1991); Philippines; Indonesia (Cairns and Zibrowius 1997); Vanuatu (Cairns 1999a); Mediterranean to the Azores (Cairns et al 1999); Bermuda; Cook Island (Cairns 1995); Chile (Cairns et al. 2005); northern Pacific (González-Romero et al. 2008); 225–2200 m.

###### Remarks.

Among congeners that are attached and display hexamerally arranged septa in four complete cycles, Caryophyllia (C.) diomedeae closely resembles *C.sarsiae* Zibrowius, 1974a (Cairns 1991). It is therefore no surprise that Cairns and Parker (1992) mistook their records of *C.diomedeae* for *C.sarsiae*, in which they mention the latter to occur in South Africa but did not provide the associated locality data for their South African records. Nonetheless, the imaged specimens (listed under imagery data) seem to have been mixed with *C.sarsiae* and have therefore been separated based on: (i) having a smooth theca and costae restricted to calicular margin (Cairns 1995), as compared with *C.sarsiae* which has costae prominent throughout corallum, and (ii) pali consistently being 1 mm in width as compared with *C.sarsiae* which bears pali being three times less the width of the pali bearing septa (key in Kitahara et al. 2010).

##### Caryophyllia (Caryophyllia) ephyala

Taxon classificationAnimaliaScleractiniaCaryophylliidae

Alcock, 1891

050231F4-D871-5D07-9F14-94F63D5FEF7F

Fig. 1O, P

Caryophyllia ephyala Alcock in Wood-Mason & Alcock, 1891: 6–2. –Alcock 1898: 13–14, pl. 1, fig. 4, 4A. –Gardiner 1904: 117–118. –Yabe and Eguchi 1932a: 388–389. –Cairns and Keller 1993: 219.

###### Type locality.

Off the western margin of Andaman Sea, India (HMS ‘Investigator’ stn. 56); 439–402 m (Wood-Mason and Alcock 1891).

###### Type material.

Types are presumably deposited at the IM.

###### Material examined.

SAMC_A072974 (2 specimens): Western margin, 168 km off PATERNOSTER/173 km off Brak Estuary, 32°05'41.99"S, 16°19'47.99"E; 440 m.

###### Description.

Corallum ceratoid, attached, tapering to a slightly curved and slender pedicel (PD:GCD = 0.20). Calice slightly elliptical (GCD:LCD = 1.1), with slightly lancet calicular margin. Largest specimen examined (SAMC_A072974) 11.2 × 9.9 mm in CD, 2.4 mm in PD, and 23.0 mm in H. Costae poorly developed, but C_1–2_ more prominent and double the width of C_3–4._ C_3–4_ equal in width. All costae prominent at calicular margin, disappearing towards base, and separated by shallow and thin intercostal furrows. Theca glistening, with faint costal ridges. Corallum white to light brown.

Septa hexamerally arranged in four complete cycles according to the formula: S_1_ ≥ S_2_ > S_3_ > S_4_ (48 septa). S_1_ highly exsert, and extend towards columella with slightly sinuous axial margins. S_2_ equal to or slightly less wide and exsert than S_1_, but otherwise similar in profile. Higher cycle septa progressively less exsert, but S_4_ more exsert than S_3_. S_4_ joining neighbouring S_1–2_ and forming a slightly lanceted calicular margin. S_3_^2^/_3_ the width of S_2,_ but have a more sinuous axial margin. Each S_3_ bears a thin and sinuous pali (12 P_3_). S_4_ rudimentary, with straight to slightly sinuous axial margins. All septa and pali covered in granules. Fossa of moderate depth, containing a fascicular columella composed of five or six ribbon-like elements.

###### Distribution.

Regional: Western and eastern (Gardiner 1904) margin of South Africa, off Paternoster extending towards Buffalo River mouth (Gardiner 1904); 146–567 m. Elsewhere: Japan (Yabe and Eguchi 1932a); Andaman Sea (Alcock 1891); 146–1289 m (Cairns and Keller 1993).

###### Remarks.

Caryophyllia (C.) ephyala belongs to the largest morphological group of the genus, having septa hexamerally arranged in four complete cycles, and when keyed out comes closest to *C.huinayensis* Cairns, Häussermann & Försterra, 2005. However, *C.ephyala* is distinguished from *C.huinayensis* in its calicular margin being slightly lanceted and having S_3_ larger than S_4._ Furthermore, these species differ in distributional range, of which *C.ephyala* is known from the Indian Ocean and *C.huinayensis* recorded in the South Pacific. Among the South African caryophylliids, *C.ephyala* superficially resembles *C.scobinosa* Alcock, 1902 in having a slightly lanceted calicular margin, poorly developed costae, and a thin pedicel, but can be differentiated by having a circular calice, corallum attached, and consistently having four complete septa cycles. This species was previously reported from South Africa, off Mossel Bay extending north of Richards Bay (Gardiner 1904 – specimens could not be traced). Thus, the new record reported herein extends its regional distribution to the South Atlantic, off Lamberts Bay.

##### Caryophyllia (Caryophyllia) grandis

Taxon classificationAnimaliaScleractiniaCaryophylliidae

Gardiner & Waugh, 1938

FF4679EC-F501-5A34-B4B2-C40EEBAA22E1

Fig. 2A, B

Caryophyllia clavus . –von Marenzeller 1904a: 281 (in part: pl. 16, figs 9–9I).
Caryophyllia grandis Gardiner & Waugh, 1938: 177, pl. 1, fig. 2. –Zibrowius and Gili 1990: 32. –Cairns 1991: 12. –Cairns and Keller 1993: 234.–Cairns and Zibrowius 1997: 96, fig. 9G–H. –Cairns 1998: 376. –Cairns et al. 1999: 20. –Cairns 2004a: 277. –Kitahara et al. 2010: 102, figs 47–52.
Caryophyllia arcuata . –Boshoff 1980: 36.

###### Type locality.

Off the west of Fadiffolu, Maldives (HEMS ‘Mabahiss’ stn. 145E: 4°58'42"N, 73°16'24"E); 494 m (Gardiner and Waugh 1938).

###### Type material.

Four syntypes are deposited at the NHMUK (Cairns and Keller 1993; Kitahara et al. 2010).

###### Material examined.

SAMC_A073150 (1 specimen): Southern margin, 26 km off Mazeppa Bay/33 km off Great Kei Estuary, 32°41'12.12"S, 28°43'54.12"E; 480–490m. SAM_H2813 (1 specimen): Eastern margin, 20 km off Cape Vidal/23 km off St Lucia Estuary, 27°59'30.00"S, 32°40'47.99"E; 550 m. **ORI_DIIIa3 (1 specimen)**: Eastern margin, other locality information unknown. **USNM 62497 (4 specimens)**: Eastern margin, 4 km from Shaka’s Rock/5 km off Mhlali Estuary, 29°29'23.46"S, 31°15'48.92"E; 183–220 m.

###### Description.

Corallum large, trochoid, usually free, and curved with a narrow pedicel (GCD:PD = 0.08–0.13) Calice elliptical (GCD:LCD = 1.41–1.75), calicular margin jagged. Largest specimen examined (ORI_DIIIa3) 35.0 × 20.0 mm in CD, 3.0 mm in PD, and 30.0 mm in H. C_1–3_ slightly ridged or absent. Upper theca and septal faces light beige, with white or discoloured lower theca.

Septa hexamerally arranged in five complete cycles according to the formula: S_1–3_ > S_4_ > S_5_ (96 septa). S_1–3_ highly exsert, with straight to slightly sinuous axial margins. S_4_ less exsert than S_1–3_, each bearing a paliform lobe (24 P_4_). S_5_ more exsert than S_4_, and fuse to adjacent septa at the calicular margin forming lancets. Septal faces and pali bear granules arranged parallel to septal margin. Pali thin, with sinuous axial margin, usually narrower than S_4_, and forming crown encircling columella. Fossa moderately deep, composed of a fascicular columella composed of 6–8 broad twisted elements.

###### Distribution.

Regional: Southern to eastern margin of South Africa, off Mazeppa Bay extending towards off Cape Vidal; 183–550 m. Elsewhere: Australia (Cairns 1998, 2004); New Caledonia (Kitahara et al. 2010); Indonesia (Cairns and Zibrowius 1997); Sumatra (von Marenzeller, 1904); Mozambique (Cairns and Keller 1993); and Maldives (Gardiner and Waugh 1938); 183–595 m.

###### Remarks.

Caryophyllia (C.) grandis closely resembles *C.ambriosa* Alcock, 1898, for which similarities exist in the number of septa (< 96 septa), corallum shape, and substrate relationship (both unattached). However, *C.grandis* is distinguished by its narrower pedicel and in having S_4_ wider than S_5_. *Caryophylliagrandis* also has a brownish theca (Cairns and Keller 1993), as compared with *C.ambriosa*, which is uniformly white. *Caryophylliagrandis* was first recorded from South African (off the Southern margin, off Knysna, 500 m) by von Marenzeller (1904a), who reported several *Caryophyllia* under the name *C.clavus* Scacchi, 1835 (*C.grandis* being one of them). Subsequently, Boshoff (1981) identified *C.grandis* as *C.arcuata* (Milne-Edwards & Haime, 1848a) in his annotated checklist of the southern Africa fauna. Thus, Zibrowius and Gili (1990) represent the first historically reliable record of *C.grandis* in the South African region, and authors mention that this species is represented in various localities off Natal but did not list the South African material examined. Cairns and Keller (1993) built on Zibrowius and Gili’s (1990) also reported *C.grandis* in Mozambique. The new records of *C.grandis* (SAM_H2813 and SAMC_A073150) are within the previously reported localities (von [Bibr B177][Bibr B177]; Zibrowius and Gili 1990; Cairns and Keller 1993).

##### Caryophyllia (Caryophyllia) lamellifera

Taxon classificationAnimaliaScleractiniaCaryophylliidae

Moseley, 1881

0F748792-C81A-58E1-AEDE-181F77072DAD

Fig. 2C, D

Caryophyllia lamellifera Moseley, 1881: 140–141, pl. 1, fig. 7A, B. –Hutton 1904: 315. –Cairns 1991: 12. –Cairns 1995: 51–52, pl. 9, fig. I, pl. 10, figs A–C. –Cairns and Zibrowius 1997: 90. –Cairns 1999a: 74–75. –Cairns et al. 1999: 20. –Cairns 2004: 278. –Kitahara et al. 2010: 104, 105, 113, 115, figs 64–68.
Caryophyllia (Caryophyllia) lamellifera . –Kitahara and Cairns 2021: 483–485, figs 263F–G, 264.

###### Type locality.

Kermadec Ridge (HMS ‘Challenger’ stn. 170: 29°55'S, 178°14'W); 1152 m (Moseley 1881; Kitahara et al. 2010).

###### Type material.

Two uncatalogued syntypes are deposited at the NHMUK (Kitahara et al. 2010).

###### Material examined.

**SAMC_A090155 (1 specimen)**: Eastern margin, Sodwana, 120 m.

###### Description.

Corallum trochoid and attached to substrate by a broad pedicel (PD:GCD = 1.5) that expands into an encrusting base. Specimen examined 12.8 × 10.7 mm in CD, 32.5 mm in H, and displays a case of rejuvenescence. Calice elliptical (GCD:LCD = 1.27), with a jagged calicular margin. Costae poorly developed, but prominent near calicular margin and progressively disappearing towards base. Theca covered with thin transverse ridges. Corallum mostly white, but brownish purple near calicular margin.

Septa hexamerally arranged in five cycles, the last incomplete, according to the formula: S_1–2_ > S_3_ > S_4_ > S_5_ (52 total septa). S_1–2_ equal in width and most exsert septa, with straight to slightly sinuous axial margins. S_3–4_ progressively less exsert, with sinuous axial margins, S_3_ being most sinuous. S_3_^1^/_3_ less wide than S_1–2_, bearing a thick and sinuous pali (13 P_3_). S_4_ dimorphic in size: half systems with S_5_ the S_4_ neighbouring S_1_ being ^1^/_3_ less wide than S_3_, and S_4_ flanked by S_5_ being the same width as S_3;_ whilst in half systems without S_5_ S_4_ is ^1^/_5_ less wide than S_3._ S_5_ slightly less wide than S_4._ Septal and palar faces covered with granules arranged in a perpendicular manner. Fossa moderately deep, containing a fascicular columella composed of 12 twisted elements.

###### Distribution.

Regional: Eastern margin of South Africa, off Sodwana; 120 m. Elsewhere: Australia (Cairns 1995, 2004a); New Zealand (Cairns 1995); New Caledonia (Kitahara et al. 2010; Kitahara and Cairns 2021); Philippines; Indonesia (Cairns and Zibrowius 1997); Wallis and Futuna Islands; and Vanuatu (Cairns 1999a); 89–1152 m.

###### Remarks.

As noted by Kitahara et al. (2010), Caryophyllia (C.) lamellifera differs from the other five congeners (*C.rugosa* Moseley, 1880, *C.corrugata* Cairns, 1979, *C.cinticulata* (Alcock, 1898), *C.versicolorata* Kitahara, Cairns & Miller, 2010a, and *C.aspera* Kitahara, Cairns & Miller, 2010a) that have theca covered with transverse ridges, by its septal arrangement and profile. Although *C.lamellifera* resembles *C.corrugata* and *C.aspera* in having hexamerally arranged septa, it differs from these two species in having S_1_ = S_2_ as compared with S_1_ > S_2_ as in *C.corrugata* and *C.aspera*. Septal exsertness is another distinguishing feature, particularly when comparing *C.lamellifera* with *C.versicolorata*, for which the former displays a highly exsert S_1_ (2 mm) as compared with 1.5 mm in *C.versicolorata*. Furthermore, all septa of *C.lamellifera* have sinuous axial margins while the S_1_ of *C.aspera* are straight. *Caryophyllialamellifera* differs from the other two South African congeners (*C.rugosa* and *C.cinticulata*) in having septa hexamerally arranged in five incomplete cycles in contrast to septa octamerally (may sometimes be decamerally) arranged in three cycles in *C.rugosa*, and decamerally arranged in three cycles in *C.cinticulata*. The only examined South African specimen of *C.lamellifera* varies from those from New Caledonia in having 13 pali instead of 12, having septa arranged in five incomplete cycles (6:6:12:24:4), and in the dimorphic size shown by S_4_. This account represents a new record for South Africa and extends the previous Pacific distribution of *C.lamellifera* into the Indian Ocean.

##### Caryophyllia (Caryophyllia) profunda

Taxon classificationAnimaliaScleractiniaCaryophylliidae

Moseley, 1881

9422C605-266F-59AD-A538-3B2E94DD2DE7

Fig. 2E, F

Caryophyllia profunda Moseley, 1881: 138–139 pl. 1, figs 6, 6b.–von Marenzeller 1904a, 298. –Gardiner 1913: 688–689. –Gardiner 1929: 126. –Gardiner 1939: 331.–Ralph 1948: 108, fig. 2. –Squires 1964, pl. 11. –Squires 1969: 16–17, pl. 6, map 1. –Ralph and Squires 1962: 3, 6–7, pl. 1, figs 8–11. –Squires and Keyes 1967: 15, 17, 23, pl. 2, figs 1–4. –Zibrowius 1974b: 751–755, pl. 1, figs 1–10. –Cairns 1979: 206. –Cairns 1982: 17–19, pl. 5, figs 1–5. –Zibrowius and Gili 1990: 25–26, pl. 4, figs L–R. –Cairns and Keller 1993: 235–236. –Cairns 1995: 44–45, pl. 7, fig. I, pl.8, figs A–C. –Kitahara et al. 2010: 114.
Caryophyllia cyathus .–von Marenzeller 1904a: 295, pl. 16, figs 6, 6A.–Hoffmeister 1933: 14, pl. 4, figs 4–5.–Gardiner 1939: 330–331.–Squires 1961: 7.
Caryophyllia planilamellata . –Dennant 1906: 157–158, pl. 6, figs 4A, B. –Squires 1961: 18.
Caryophyllia clavus . –Wells 1958: 265, pl. 1, figs 12, 13.
Caryophyllia cf. C.maculata. –Ralph 1948: 108, fig. 2. –Ralph and Squires 1962: 3, 7, pl. 2, figs 1–2. –Squires and Keyes 1967: 15, 17, 23, pl. 2, figs 4, 5.

###### Type locality.

Off Nightingale Island, Sub-Antarctica (HMS ‘Challenger’ stn. 135: 37°0'50"S, 12°19'10"W); 183–274 m (Moseley 1881).

###### Type material.

Twenty syntypes are deposited at the NHMUK (Cairns 1982).

###### Material examined.

None.

###### Imagery data.

**DTEValdivia Stn. 108 (1 specimen)**: Southern margin, 60 km off AGULHAS/69 km off De Mond-Heuningnes Estuary, 35°19'18.00"S, 20°15'17.99"E; 126 m.

###### Description.

Adapted from Cairns (1982) and von Marenzeller (1904a): Corallum trochoid, slightly curved, and attached to substrate by a broad pedicel. Calice slightly elliptical (GCD:LCD = 1.04), calicular margins slightly serrate. Imaged specimen 25.0 × 24.0 mm in CD and 54.0 mm in H. Costae flat, more prominent near calicular margin, and equal in width. C_1–3_ sometimes slightly ridged. Theca thick, porcelaneous, and finely granulated. Corallum white with brown theca.

Septa hexamerally arranged in five complete cycles according to the formula: S_1–2_ > S_3_ > S_4_ > S_5_ (96 septa). S_1–2_ moderately exsert, equal in width, and bearing axial margins that join columella deep in fossa. Higher cycle septa progressively less exsert. S_3_^1^/_5_ less wide than S_1–2._ S_4_ ~ ¾ the width of S_3_, each bearing a broad pali (24 P_4_), with vertical inner margin. S_5_ ¾ the width of S_4._ All septal faces slightly granulated and septal margins straight, except for S_4_ which may be slightly sinuous. Fossa moderately deep containing a fascicular columella encircled by a paliform crown.

###### Distribution.

Regional: Southern margin of South Africa, off Agulhas; 126 m. Elsewhere: St Paul and Amsterdam Islands (Zibrowius 1974b); Australia (Hoffmeister 1933); New Zealand (Ralph and Squires 1962; Squires and Keyes 1967; Cairns 1995); Sub-Antarctic Islands (Moseley 1881; von Marenzeller 1904a; Zibrowius 1974b; Cairns 1982); 35–1116 m.

###### Remarks.

Caryophyllia (C.) profunda is one of the several *Caryophyllia* species reported under the name of *Caryophylliacyathus* (Ellis & Solander, 1786) by von Marenzeller (1904a), a long-standing confusion that Zibrowius (1974b) discusses in his account of the corals of the St Paul and Amsterdam Islands. *Caryophylliaprofunda* differs from *C.cyathus* in its S_1_ being straight as compared with slightly sinuous in the latter, and having 24–25 instead of 20 P_4_. Nonetheless, von Marenzeller’s (1904a) records form the basis of the occurrence of this species in South African territory, as no other known South African samples have been reported subsequently.

##### Caryophyllia (Caryophyllia) quadragenaria

Taxon classificationAnimaliaScleractiniaCaryophylliidae

Alcock, 1902

3F6F50BF-531E-5FE8-8BF7-07DB1B8323E7

Fig. 2G, H

Caryophyllia quadragenaria Alcock, 1902a: 91–92. –Alcock 1902b: 10, pl. 1, figs 4, 4A. –Keller 1981: 18. –Cairns 1991: 12. –Cairns 1994: 46–47, pl. 20, figs C–H, pl. 51, figs C–D. –Cairns 1995: 45–46, pl. 7, figs G–H. –Cairns and Zibrowius 1997: 88, 93. –Cairns 1998: 375, –Cairns 1999a: 73. –Cairns et al. 1999: 20. –Cairns 2004: 278. –Gonźalez-Romero et al. 2008: 1–2, fig. 2. –Kitahara et al. 2010a: 107, figs 78–81.
Caryophyllia scobinosa . –Yabe and Eguchi 1942b: 119 (in part).
Caryophyllia scobinosa decapali Yabe & Eguchi, 1942b: 120, 149, pl. 10, figs 6–7. –Eguchi 1968: C33–34. –Eguchi and Miyawaki 1975: 56. –Cairns 1991: 12.
Caryophyllia profunda . –Squires and Keyes 1967: 23 (in part).
Caryophyllia decapali . –Grygier 1983: 420. –Zibrowius and Grygier 1985: 120, figs 10–11.
Caryophyllia (Caryophyllia) quadragenaria . –Kitahara and Cairns 2021:490–491, 493, figs 268, 269A–C.

###### Type locality.

Off Makassar Strait, Banda and Timor seas (Indonesia) (HMS ‘Siboga’ stns 90, 251, 289: 1°17'50"N, 12°19'10"W; 5°28'40"S, 132°02'00"W; and 9°0'30"S, 122°24'5"W respectively); 54–281 m (Alcock 1902a).

###### Type material.

Two syntypes are deposited at the ZMA (Cairns 1995; Cairns and Zibrowius 1997; Kitahara et al. 2010a).

###### Material examined.

USNM 91538 (3 damaged specimens): Eastern margin, 69 km off Durban/68 km off Beachwood Mangroves, 30°08'59.99"S, 31°37'12.00"E; 930 m. USNM 91539 (1 specimen): Eastern margin, 44 km from Shaka’s Rock/45 km off Mhlali Estuary, 29°41'59.99"S, 31°37'47.99"E; 350 m.

###### Description.

Corallum small, ceratoid to subcylindrical, slightly curved, and attached to substrate by a robust but narrow pedicel (PD:GCD = 0.3). Calice circular to slightly elliptical (GCD:LCD = 1.1), with a slightly lanceted calicular margin. Largest examined specimen (USNM 91539) 8.2 × 7.4 mm, 9.3 mm in H, and 2.5 mm in PD. Costa equal in width, separated by moderately deep furrows that fade towards base. All costae covered with low granules. Theca thick. Corallum white to light brown.

Septa decamerally arranged in three cycles according to the formula: S_1_ > S_2_ > S_3_ (40 septa). S_1_ highly exsert and almost meet columella with straight to slightly sinuous axial margins. S_2_ ~ ¾ the width of S_1,_ least exsert, but bear the most sinuous axial margins. Each S_2_ bears a tall (extending above columella elements) and sinuous pali (10 P_2_). S_3_ slightly less wide than S_2_ and bearing a less sinuous axial margin. All septal faces granular. Fossa moderately deep, with a fascicular columella composed of 6–8 ribbon-like elements.

###### Distribution.

Regional: Eastern margin of South Africa, off Durban extending towards off Shaka’s Rock; 350–930 m. Elsewhere: Japan (Cairns 1994); South China Sea (Cairns and Zibrowius 1997); Indonesia (Alcock 1902a; Cairns and Zibrowius 1997); New Caledonia (Kitahara et al. 2010a; Kitahara and Cairns 2021); Vanuatu, Wallis, and Futuna (Cairns 1999a); New Zealand (Cairns 1995); Australia (Cairns 1998); North Pacific (Gonźalez-Romero et al. 2008); 54–1669 m.

###### Remarks.

Among the *Caryophyllia* that have decameral septal symmetry, Caryophyllia (C.) quadragenaria most closely resembles *C.perculta* Cairns, 1991, but can be differentiated by having smooth or slightly granular instead of extremely granular or carinate septal faces . *C.quadragenaria* may also be mistaken for *C.cinticulata* (Alcock, 1898) and *C.rugosa* Moseley, 1881 but may be differentiated from these two South African congeners by lacking circumferential thecal transverse ridges reported. The specimens reported herein represent new records for the Indian Ocean.

##### Caryophyllia (Caryophyllia) rugosa

Taxon classificationAnimaliaScleractiniaCaryophylliidae

Moseley, 1881

7C72558B-957E-581D-8E67-92B46CBD4EC4

Fig. 2I, J

Caryophyllia rugosa Moseley, 1881: 141–143, pl. 1, fig. 8. –Wells 1954: 469, pl. 177, figs 5, 6. –Cairns 1984: 11–13, pl. 2, figs A, B, pl. 4, fig. I. –Cairns 1991: 20. –Cairns and Keller 1993: 236, fig. 3I. –Kitahara et al. 2010a: 108, figs 93–97.
Caryophyllia paraoctopali Yabe & Eguchi, 1942b: 120, 150, pl. 10, fig. 12.
Caryophylliia (Caryophyllia) rugosa . –Cairns 1994: 47, pl. 20, fig. I, pl. 21, fig. A. –Cairns and Zibrowius 1997: 91–92. –Cairns 1998: 375. –Kitahara and Cairns 2021: 495–496, 498, figs 269H–I, 271, 272A–C.

###### Type locality.

Off Banda and Sulu Seas, Indonesia (HMS ‘Challenger’ stns 192 and 201); 187–230 m (Moseley 1881).

###### Type material.

Syntypes are deposited at the BMNH (Cairns 1994).

###### Material examined.

SAMC_A073073 (1 specimen): Eastern margin, 29 km off Richards Bay/20 km off Nhlabane Estuary, 28°44'23.99"S, 32°23'12.11"E; 320–340 m. SAMC_A073180 (1 specimen): Southern margin, 33 km from Mazeppa Bay/24 km off Cwili Estuary, 32°45'47.88"S, 28°36'24.12"E; 240–250 m. SAMC_A090071(1 specimen): Eastern margin, 15 km south of Ponta Do Ouro/17 km off Kosi-Kumpungwini (Sifungwe) Estuary, 26°55'30.00"S, 33°01'05.99"E; 370 m. SAMC_A090077 (1 specimen): Eastern margin, 34 km off Port Dunford/38 km off Kosi-Kumpungwini (Sifungwe) Estuary, 29°10'00.00"S, 32°04'59.99"E; 170 m. **USNM 91529 (1 specimen)**: Eastern margin, 37 km south of Ponta Do Ouro/23 km off Kosi Bay Estuary, 27°11'05.99"S, 32°50'53.88"E; 100 m.

###### Description.

Corallum small, ceratoid to trochoid, and attached through a robust pedicel (PD:GCD = 0.3–0.8) that expands into a large encrusting base. Calice circular to elliptical (GCD:LCD = 1.0–1.2), with serrated calicular margin. Largest examined specimen (USNM 91529) 6.9 × 5.8 mm in CD, 2.4 mm in PD, and 12.5 mm in H. Costae covered with well-defined transverse ridges; ridges split and re-join around corallum. Corallum white to light brown.

Septa octamerally arranged in three cycles according to the formula: S_1_ > S_2_ > S_3_ (32 septa). However, one specimen (SAMC_A090071) has decamerally arranged septa (40 septa). S_1_ highly exsert, extending almost to columella. S_2–3_ becoming progressively narrower and less exsert. All septa bear sinuous axial margins, sinuosity being extreme in S_1–2_. S_2_ bearing a highly sinuous and thick pali (8 P_2_). Fossa shallow containing a prominent fascicular columella.

###### Distribution.

Regional: Eastern margin of South Africa, off Kei Mouth extending towards Kosi Bay Estuary, (15 km south of Ponta Do Ouro: Mozambique); 100–370 m. Elsewhere: New Caledonia (Kitahara et al. 2010a; Kitahara and Cairns 2021); Hawaii (Cairns 1984); Japan; East China Sea (Cairns 1994); Philippines; Indonesia (Cairns and Zibrowius 1997); Wallis and Futuna Islands; Vanuatu (Cairns 1999a); Australia (Cairns 1998, 2004; Kitahara et al. 2010); New Zealand (Cairns 1995); Mozambique; Kenya; and Maldives (Cairns and Keller 1993); 71–581 m.

###### Remarks.

Kitahara et al. (2010) noted that Caryophyllia (C.) rugosa is easily distinguished from congeners by the presence of transverse ridges and its extremely sinuous septa and pali. Although some specimens have a hexameral or decameral symmetry, the most common septal symmetry is octameral (Kitahara et al. 2010). South African specimens have both septal symmetries (octameral and decameral), for which the specimen (SAMC_A090071) that have decamerally arranged septa also displays rejuvenescence of the corallum. *Caryophylliarugosa* was previously reported from South African waters by Cairns and Keller (1993) off Lake Kosi (in KwaZulu-Natal), and the examined specimens herein extend its regional distribution further north towards the Mozambican border.

##### Caryophyllia (Caryophyllia) sarsiae

Taxon classificationAnimaliaScleractiniaCaryophylliidae

Zibrowuis, 1974

FFD186A5-BB93-5571-A97E-D300C6D0A21B

Fig. 2K–N

Caryophyllia cylindracea . –Jourdan 1895: 11 (in part).
Balanophyllia cornu . –Jourdan 1895: 27, pl. 2, fig. 21A, B.
Caryophyllia clavus . –Gravier 1920: 16 (in part).
Caryophyllia arcuata . –Gravier 1920: 25 (in part), pl. 2, figs 26, 29.
Caryophyllia sarsiae Zibrowius, 1974b: 779–782, pl. 3, figs A–F. –Zibrowius et al. 1975: 95, fig. 3A–E. –Zibrowius 1980: 62–63, pl. 24, figs A–J.
Caryophyllia sp. –Zibrowius 1974c: 755–756, pl. 1, fig. 11, pl. 2, fig. 1.

###### Type locality.

Southeast of Le Chapelle Bank, west of Brittany (RV ‘Sarsia’ stn. 1: 47°19'00"N, 06°36'00"W); 880–980 m (Zibrowius 1974b).

###### Type material.

Types are deposited at the NHMUK (Zibrowius 1974b).

###### Material examined.

MN_SM 162 (2 specimen): Southern margin, 40 km off Kei Mouth/29 km off Cwili Estuary, 32°55'00.00"S, 28°31'00.00"E; 630 m. MN_SM 174 (1 specimen): Southern margin, 26 km off Kidds Beach/27 km off Ncera Estuary, 33°19'36.00"S, 27°52'23.99"E; 760 m. MN_SM 226 (19 specimens): Southern margin, 32 km off Mazeppa Bay/24 km off Kobole Estuary, 32°28'36.00"S, 28°58'48.00"E; 710–775 m.

###### Description.

Corallum ceratoid, straight to slightly curved, and attached to substrate through a robust pedicel (PD:GCD < 0.60). Calice circular to slightly elliptical (GCD: LCD = 1.0–1.1), with serrated calicular margin. Largest imaged specimen (MN_SM 226) 15.0 × 15.0 mm in CD, 8.0 mm in PD, and 42.0 mm in H. Costae equal, flat, and smooth. Theca granular. Corallum white, with beige theca.

Septa hexamerally arranged in four cycles according to the formula: S_1–2_ > S_3_ ≥ S_4_ (48 septa). S_1–2_ equal in width, highly exsert, thick, and extend to columella with straight (although S_1_ may be slightly sinuous in some specimens) axial margins. S_3_ ¾ the width of S_1–2_, less exsert, with extremely sinuous axial margins, which bear narrow and sinuous pali (12 P_3_). S_4_ equal to slightly smaller than S_3_, but equally exsert, bearing straight axial margins. All septal faces appearing smooth, with small and randomly arranged granules deeper in fossa. Fossa of moderate depth, with fascicular columella composed of 6–9 twisted laths.

###### Distribution.

Regional: Southern margin of South Africa, off Kei Mouth extending towards Kidds Beach; 630–760 m. Elsewhere: Madeira and Azores, extending to off Portugal and Ireland (Zibrowius 1974b); the Mediterranean (Zibrowius 1980); 520–2200 m.

###### Remarks.

All the imaged specimens represented here have: (i) septa hexamerally arranged in four cycles; (ii) S_1–3_ bearing sinuous axial margins, S_3_ being the most extreme; and (ii) S_4_ having straight axial margins. Further to the septa symmetry and profile, specimens have granulated costae throughout corallum. Zibrowius (1974b) noted the intraspecific variation that may exist in the corallum shape of *C.sarsiae*, which is highly dependent on the environmental conditions, particularly in relation to substrate attachment. Some of the examined specimens (for example MN_SM 229) are attached to *Solenosmiliavariabilis* Duncan, 1873, and do indeed display a more curved corallum. This account of *C.sarsiae* in South African territory extends the species distributional range further south and, therefore, represents a new record for the region.

##### Caryophyllia (Caryophyllia) scobinosa

Taxon classificationAnimaliaScleractiniaCaryophylliidae

Alcock, 1902

0A2BB9E0-AF23-5F69-B88D-8936E19081ED

Fig. 2O, P

Caryophyllia scobinosa Alcock, 1902a: 90. –Alcock 1902b: 8, pl. 1, figs 2, 2A. –Gardiner and Waugh 1938: 177–178. –Cairns 1991: 12. –Cairns and Keller 1993: 235. –Cairns 1994: 45–46, pl. 20, figs A, B (in part). –Cairns 1995: 52–53, pl. 10, figs G–I, pl. 11, figs A–C. –Cairns and Zibrowius 1997: 94. –Cairns 1999a: 75. –Cairns et al. 1999: 20. –Kitahara 2007: 498, 507, 510, fig. 2K. –Kitahara et al. 2008: 16, fig. 2D. –Cairns 2004: 278. –Kitahara et al 2010a: 109, figs 113,117.
Caryophyllia cultrifera Alcock, 1902b: 7–8, figs 1, 1A. –Faustino 1927: 67–68, pl. 8, figs 8, 9. –Veron 1986: 905.
Caryophyllia clavus . –von Marenzeller 1904a: 281 (in part ‘Valdivia’–246), pl. 16, figs 9C–G.

###### Type locality.

Off Flores and Sulu Seas (HMS ‘Siboga’ stns. 45 and 102: 7°24'00"S, 118°15'20"E and 6°04'10"N, 120°44'00"E, respectively); 535–794 m (Alcock 1902a).

###### Type material.

Six syntypes are deposited at the ZMA (Cairns 1994; Kitahara et al. 2010).

###### Material examined.

SAM_H1248 (1 specimen): Eastern margin, 17 km from DURBAN/8 km off Mdloti Estuary, 29°42'34.21"S, 31°05'50.82"E; 91 m.

###### Description.

Corallum ceratoid, unattached, and curved to a narrow pedicel (PD:GCD = 0.2). Calice slightly elliptical (GCD:LCD = 1.1), with jagged calicular margin. Specimen examined 9.5 × 8.6 mm in CD, 2.0 mm in PD, and 11.3 mm in H. Costae granular, flat, and separated by narrow intercostal furrows that fade towards base. Corallum white.

Septa hexamerally arranged in four cycles according to the formula: S_1–2_ > S_3_ > S_4_ (48 septa). S_1–2_ thick, slightly exsert, and extend to columella deep in fossa with straight to slightly sinuous axial margins. S_2–3_ progressively less exsert. S_3_ axial margin more sinuous than remaining septa. S_3_ ~ ^2^_/3_ the width to S_1–2_, each bearing a sinuous pali (12 P_3_). S_4_ rudimentary, but joining adjacent S_1_ and S_2_ at calicular margin to form rectangular lancets. All septal faces bear small pointed granules. Fossa relatively shallow containing a fascicular columella composed of five ribbon-like elements.

###### Distribution.

Regional: Eastern margin of South Africa, off Durban; 91 m. Elsewhere: Brazil (Kitahara 2007); New Zealand (Cairns 1995); New Caledonia (Kitahara et al. 2010; Kitahara and Cairns 2021); Australia (Cairns 1995, 2004; Kitahara et al. 2010); Tanzania (von Marenzeller 1904; Gardiner and Waugh 1938); Madagascar Plateau; Walter Shoal (Cairns and Keller 1993); Sulu Sea; Celebes Sea (Alcock 1902a); off Tonga and Samoa (Cairns 1995); Philippines; Indonesia (Cairns and Zibrowius 1997); Wallis and Futuna Islands; and Vanuatu (Cairns 1999a); 253–2450 m.

###### Remarks.

As noted by Kitahara et al. (2010), Caryophyllia (C.) scobinosa can be distinguished from other unattached Indo-Pacific *Caryophyllia* in having 48–72 septa, 12–14 pali, and a jagged calicular margin. The South African representative of *C.scobinosa* superficially resembles *C.stellula* Cairns, 1998 but can be distinguished by its S_4_ being more exsert than S3, and also by joining neighbouring S_1_ and S_2_ at the calicular margin forming triangular lancets. Corallum size and density also differentiate the adult forms of these two species. Although *C.scobinosa* has been previously reported in neighbouring areas (Cairns and Keller 1993), the current study confirms the occurrence of this species further south in the South African territory.

##### Caryophyllia (Caryophyllia) stellula

Taxon classificationAnimaliaScleractiniaCaryophylliidae

Cairns, 1998

37ADDC7A-4002-56F8-83BF-DE2D3BF2CC11

Fig. 3A, B

Caryophyllia epithecata . –Gardiner 1904: 114–117, pl. 1, figs 3 A–C (in part: localities I–V). –Cairns and Keller 1993: 219.
Caryophyllia (Caryophyllia) stellula Cairns, 1998: 375–376, fig. 2A–C. –Cairns 2004a: 278.

###### Type locality.

Off west of Rottnest Island, Australia (RV ‘Diamantina’ stn. 25: 31°48'00.0"S, 114°58'12.0"E); 402 m (Cairns 1998).

###### Type material.

The holotype is deposited at the WAM (Cairns 1998).

###### Material examined.

DEFF_SVMEC–INV190 (11 specimen): Southern margin, 54 km from Cape Point/56 km off Buffels Wes Estuary, 34°43'48.62"S, 18°07'14.02"E; 386–392m. DEFF/SAEON_A32776 (1 specimen): Southern margin, 200 km from KNYSNA/210 km off Ratels Estuary, 34°43'48.62"S, 18°07'14.02"E; 636 m. SAMC_A073140 (1 specimen): Eastern margin, 6 km off Cape Vidal/17 km off St Lucia Estuary, 28°08'17.88"S, 32°36'54.00"E; 200 m. SAMC_A088909 (8 specimens): Southern margin, 140 km off Agulhas/144 km off Ratels Estuary, 36°02'29.58"S, 19°41'24.61"E; 445 m. SAMC_A088922 (3 specimens): Southern margin, 140 km off Agulhas/144 km off Ratels Estuary, 36°02'29.58"S, 19°41'24.61"E; 445–463 m. SAMC_A088923 (4 specimens): Southern margin, 140 km off Agulhas/144 km Ratels off Bulura Estuary, 36°02'29.58"S, 19°41'24.61"E; 445–463 m. SAMC_A088928 (2 specimens): Southern margin, 65 km off Cape St. Francis/70 km off Slang Estuary, 34°47'05.01"S, 24°45'42.30"E; 392 m. SAMC_A090127 (1 specimen): Southern margin, 240 km off Agulhas/247 km off De Mond-Heuningnes Estuary, 36°45'34.13"S, 21°12'46.61"E; 513 m. SAMC_A090145 (1 specimen): Southern margin, 116 km off Knysna/off Goukamma Estuary, 35°07'11.27"S, 23°02'41.91"E; 333 m. **SAM_H1378 (2 specimens)**: Southern margin: 11 km off East London/5 km off Gouda Estuary, 33°05'03.24"S, 27°49'33.40"E; 146–238 m. **SAM_H1394 (25 specimens)**: No locality data. SAM_H1396 (2 specimens): Western margin, 14 km off Saldanha/31 km off Berg River I Floodplain Estuary, 33°06'29.99"S, 18°01'59.99"E; 347 m. SAM_H1418 (8 specimens): Western margin, 1 km off Cape Point/8 km off Buffels Wes Estuary, 34°21'42.64"S, 18°30'12.06"E; 549 m. SAM_H1421 (2 specimens): Western margin1 km off Cape Point/8 km off Buffels Wes Estuary, 34°21'42.64"S, 18°30'12.06"E; 567–1024 m. SAM_H1433 (16 specimens): Western margin, 1 km off Cape Point/8 km off Buffels Wes Estuary, 34°21'42.64"S, 18°30'12.06"E; 574–732 m. SAM_H1436 (44 specimens): Southern margin, 2 km off Mosselbaai/11 km off Hartenbos Estuary, 34°11'10.12"S, 22°09'40.59"E; 165–183 m. SAM_H1448 (92 specimens): Southern margin, 241 km off Agulhas/247 km off De Mond-Heuningnes Estuary, 36°40'00.00"S, 21°25'59.99"E; 200 m. SAM_H1485 (72 specimens): Southern margin, 241 km off Agulhas/247 km off De Mond-Heuningnes Estuary, 36°40'00.00"S, 21°25'59.99"E; 200 m. SAM_H3056 (21 specimen): Southern margin, 2 km off Mosselbaai/11 km off Hartenbos Estuary, 34°11'10.12"S, 22°09'40.59"E; 229 m. SAM_H3059 (2 specimens): South Africa, no other locality data.

###### Imagery data.

BMNH 1939.7.20.249–251 (2 specimens): South Africa, locality data unknown. RV ‘Galathea’ stn. 202 (3 specimens): Eastern margin, KwaZulu-Natal, depth unknown.

###### Description.

Corallum ceratoid, unattached, curved, having a slender pedicel (PD:GCD = 0.1–0.2). Calice slightly elliptical (GCD:LCD = 1.1), with smooth to slightly serrated calicular margin. Largest specimen examined (SAM_H1485) 11.4 × 10.2 mm in CD, 1.8 mm in PD, and 22.4 mm in H. Costae poorly developed, slightly granular, and separated by shallow intercostal furrows. Costae extending towards base. Theca thick. Corallum white, with yellowish brown theca.

Septa hexamerally arranged in four cycles according to the formula: S_1–2_ > S_3_ > S_4_ (48 septa). S_1–2_ slightly exsert and extend to columella. S_3_^2^/_3_ the width of S_1–2_, and bear a sinuous pali (12 P_3_). S_4_ ¼ the width of S_3._ All septal have sinuous axial margins, S_3_ being the most sinuous. Higher cycle septa (S_3–4_) progressively less exsert (if at all). Fossa relatively deep, with a fascicular columella composed of 4–6 ribbon-like elements.

###### Distribution.

Regional: Western to eastern margin of South Africa, off Saldanha extending towards Cape Vidal; 200–567 m. Elsewhere: Western Australia (Cairns 1998); 240–402 m.

###### Remarks.

Of the *Caryophyllia* reported from the South African territory, *C.stellula* may be confused with *C.scobinosa*, but differs in having a more robust corallum, a thicker calicular margin, and different pattern of septal exsertness. Caryophyllia (C.) stellula was first reported from South Africa as *C.epithecata* (Gardiner 1904) who elevated Duncan’s (1873)C.clavusvar.epithecata to species level. However, this goes against the International Code of Zoological Nomenclature (1985: article 45g) and, therefore, the correct author of *C.epithecata* is Duncan (1873). Despite this, Duncan’s C.clavusvar.epithecata is a junior synonym of *C.smithi* (Zibrowius 1974c). In South Africa, *C.stellula* shows a broad regional distribution and the several specimens examined herein enabled the observation of intraspecific morphological variation. Some representatives have curved corallum with a narrow pedicel while others display a more robust and thicker pedicel, features also illustrated by Gardiner (1904). Nonetheless, the species consistently have septa hexameral symmetry in four complete cycles, and a total of 12 well-defined P_3_, each positioned before S_3_. Unfortunately, Gardiner’s (1904) specimens were untraceable but his records are within the reported distribution proposed herein. The new material presented here increases the known depth range of this species by 75 m.

**Figure 3.**
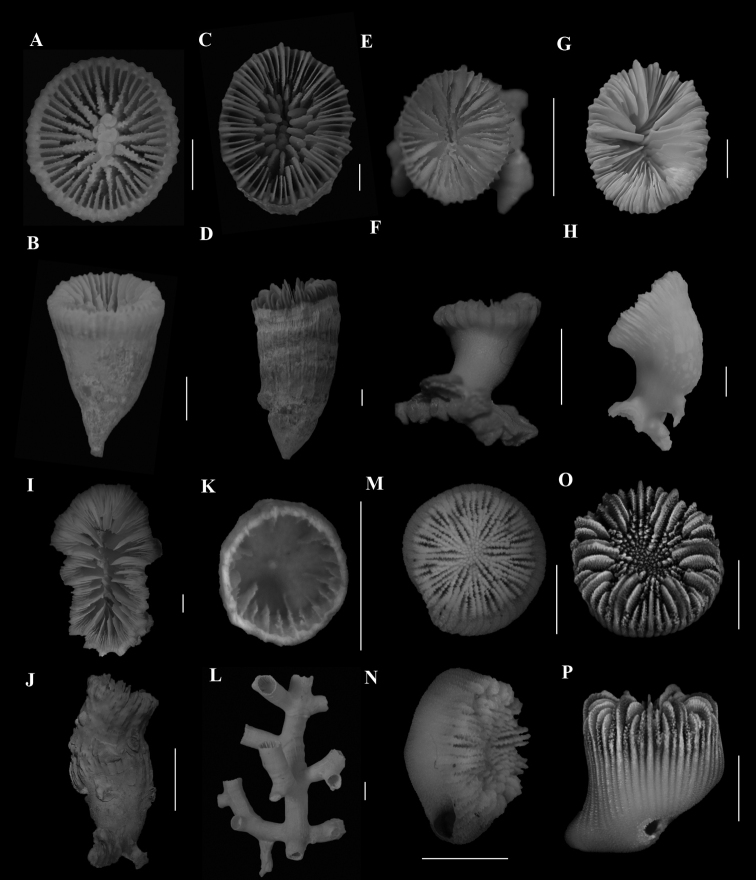
**A**, **B**Caryophyllia (Caryophyllia) stellula (SAM_H1485, off Agulhas, 200 m) **A** calicular view **B** lateral view **C**, **D**Caryophyllia (Caryophyllia) valdiviae (SAM_H3108, off Durban, depth unknown) **C** calicular view **D** lateral view **E**, **F***Crispatotrochuscornu* (UCT_NAD 17 F, off Isipingo, 49 m) **E** calicular view **F** lateral view **G**, **J***Desmophyllumdianthus***G**, **H** (SAMC_A077974, off Paternoster, 440 m) **G** calicular view **H** lateral view **I**, **J** (BMNH.1939.7.20.218, locality data unknown) **I** calicular view **J** lateral view **K**, **L***Desmophyllumpertusum* (SAM_H1605, off Melkbosstrand, depth unknown) **K** calicular view **L** lateral view **M**, **N***Goniocorelladumosa* (SAM_H3190, off Kidds Beach, 760 m) **M** calicular view **N** lateral view **O**, **P***Heterocyathusaequicostatus* (SAMC_A073186, off Durban, 150 m) **O** calicular view **P** lateral view Scale bars: 10 mm (**A–I**, **K–P**);100 mm (**J**).

##### Caryophyllia (Caryophyllia) valdiviae

Taxon classificationAnimaliaScleractiniaCaryophylliidae

Zibrowuis & Gili, 1990

B75F2EE5-12B2-5F86-A133-43163E8E4CE9

Fig. 3C, D

Caryophyllia clavus . –von Marenzeller 1904a: 281 (in part ‘Valdivia’–291–292), pl. 16, figs 9K–M.
Caryophyllia epithecata . –Boshoff 1981: 35.
Caryophyllia valdiviae Zibrowius & Gili, 1990: 28, 30, 32, pl. 2, figs P–R, pl. 3, figs A–T.

###### Type locality.

Walvis Ridge, west of Namibia (‘Benguela VI Expedition’ stn. BB12: 25°34'00.0"S, 6°07'00.0"E); 886 m (Zibrowius and Gill 1990).

###### Type material.

The holotype is deposited at the NHMUK (Zibrowius and Gill 1990).

###### Material examined.

SAM_H3108 (2 specimens): Eastern margin, 35 km from Durban/33 km off Mbokodweni Estuary, 30°03'49.62"S, 31°15'30.89"E; depth unknown. ORI_DIIIa1 (7 specimens): Eastern margin, no other locality data.

###### Description.

Corallum ceratoid, unattached, curved, and with a small pedicel (PD:GCD = 0.1). Calice subcylindrical, slightly compressed (GCD:LCD = 0.9–1.2), and with a jagged calicular margin. Largest specimen examined (SAM_H3108) 26.8 × 23.0 mm in CD, 2.8 mm in PD, and 55.3 mm in H. Costae poorly developed and flat, except for C_1–2_, which are prominent and high. C_1–2_ slightly wider than C_3–4_. All costae covered with fine granules, extending towards base, and separated by narrow intercostal striae. Theca thick. Corallum white, with beige theca.

Septa octamerally arranged in four cycles according to the formula: S_1_ > S_2_ > S_3–4_ (64 septa). S_1_ most wide, with straight axial margins that meet columella deep in fossa. S_2_ slightly less wide than S_1,_ with straight to slightly sinuous axial margins. S_1–2_ most exsert. S_3–4_^1^/_3_ less wide than S_2_, and bear sinuous axial margins. S_3_ the most sinuous and each bearing a tall and thin pali (16 P_3_). All septal faces covered with sparsely arranged granules. Fossa relatively deep, with a fascicular columella composed of 4–12 ribbon-like elements.

###### Distribution.

Regional: Western (Zibrowius and Gili 1990) and eastern margins of South Africa, off Alexander Bay (Zibrowius and Gili 1990) and southeast of Durban; 442–882 m. Elsewhere: Walvis Ridge (Zibrowius and Gili 1990); 882–2670 m.

###### Remarks.

The examined specimens match the characteristics described by Zibrowius and Gili’s (1990)Caryophyllia (C.) valdiviae specimens in having: (i) octamerally arranged septa, (ii) four cycles, and (iii) 16 P_3_. *Caryophylliavaldiviae* is similar to *C.cornulum* Cairns & Zibrowius, 1997 in adult specimens having 48–72 septa and bearing four to five septa size classes (Kitahara et al. 2010 – key). However, *C.valdiviae* can be distinguished by having septa highly exsert (1.8–4.2 mm) as compared with 0.7–1.1 mm in *C.cornulum*, S_1–2_ being equally exsert, and also in septal formula: S_1_ > S_2_ > S_3–4_ in *C.valdiviae* and S_1_ > S_3_ ≥ S_2_ in *C.cornulum*. Among the other three unattached *Caryophyllia* recorded in South Africa (*C.grandis*, *C.scobinosa*, and *C.stellula*), *C.valdiviae* overlaps in distribution and superficially resembles *C.grandis*, but may be distinguished by its adult corallum having septa octamerally arranged in four cycles (64 septa) as compared with septa hexamerally arranged in five cycles (96 septa). The number of resultant pali also differs between these two species, with *C.valdiviae* having 16 P_3_ and *C.grandis* 24 P_3._ Furthermore, *C.valdiviae* bears a robust and trochoid corallum, while *C.grandis* have a less robust and ceratoid corallum. *Caryophylliavaldiviae* is also one of the species reported by von Marenzeller (1904a) as *C.clavus* (*Valdivia Expedition* stn. 83) (Zibrowius and Gill 1990). Apart from von Marenzeller’s (1904a) misidentification of the Atlantic (Walvis Ridge) record, Boshoff (1980) reported an Indian ocean record of *C.valdiviae* as *C.epithecata*, a sample collected through UCTES and a sub-sample of SAM_H3108. Nonetheless, this KwaZulu-Natal record represents a disjunction in the previously known Atlantic distribution of this species, indicating that this species might occur all around the South African continental slope.

##### Crispatotrochus

Taxon classificationAnimaliaScleractiniaCaryophylliidae

Tenison-Woods, 1878

187815D6-BE1E-5C67-BA01-6EFC26134585

###### Diagnosis.

Corallum solitary, ceratoid to turbinate, and usually attached. Septotheca costate or covered with transverse ridges. Pali absent; columella fascicular composed of discrete, twisted elements.

###### Type species.

*Crispatotrochusinortatus* Tenison-Woods, 1878, by monotypy.

##### Crispatotrochus cornu

Taxon classificationAnimaliaScleractiniaCaryophylliidae

(Moseley, 1881)

80700D49-A5DB-51BB-AFAA-425F28C4BDE1

Fig. 3E, F

Cyathoceras cornu Moseley, 1881: 156–157, pl. 4, fig. 7 (in part). –Cairns 1979: 67, pl. 12, figs 1, 3.
Crispatotrochus cornu . –Cairns 1991: 15. –Kitahara and Cairns 2008: 63. –Cairns and Polonio 2013: 70–71, figs 2L–M, 3A–B, 10.

###### Type locality.

Off Rio de la Plata, Uruguay (HMS ‘Challenger’ stn. 320: 37°17'00"S, 53°52'00"W); 1097 m (Moseley 1881).

###### Type material.

Lectotype and paralectotype are deposited at the NMNH (Cairns 1979).

###### Material examined.

None.

###### Imagery data.

UCTES_NAD 17 F (1 specimen): Eastern margin, off Isipingo; 49 m.

###### Description.

Corallum ceratoid, attached through a robust pedicel (PD:GCD = 0.4) that expands into an encrusting base. Calice elliptical (GCD:LCD = 1.2) with a serrated calicular margin. Only imaged specimen examined (NAD 17 1F) 5.9 × 5.0 mm in CD, 2.2 mm in PD, and 7.0 mm in H. Theca granulated. Costae prominent at calicular margin, with C_1––2_ more prominent and distinctive. No intercostal striae. Corallum white.

Septa hexamerally arranged in four complete cycles according to the formula: S_1–2_ > S_3_ > S_4_ (48 septa). S_1–2_ equal in size and exsertness, with slightly sinuous axial margins, and almost meet columella. Remaining septa (S_3–4_) becoming progressively less exsert. S_3_ ~ ¾ the width of S_1–2_, and have the most sinuous axial margins. S_4_ rudimentary, also with straight to slightly sinuous axial margins. Septal faces bear small and blunt granules. Fossa moderately deep, with a rudimentary fascicular columella composed of two twisted elements.

###### Distribution.

Regional: Eastern margin of South Africa, off Isipingo; 49 m. Elsewhere: Seamount or ridge on Heezen fracture zone, South Pacific; 549 m (Cairns 1982).

###### Remarks.

The imaged specimen, that forms the basis of this species report, is consistent with the known description of *Crispatotrochuscornu* in having a robust pedicel and septa hexamerally arranged in four complete cycles (S_1–2_ > S_3_ > S_4_), but differs in having an exceptionally sinuous as opposed to a straight S_3_ as previously reported (Cairns 1979, Kitahara and Cairns 2008). This species groups with the other *Crispatotrochus* that have septa hexamerally arranged in four cycles (*C.inornatus*, *C.galapagensis* Cairns, 1991, *C.rugosus* Cairns, 1995, and *C.irregularis* Cairns, 1982), all previously reported from the Pacific Ocean. *Crispatotrochuscornu* appears to be a juvenile form and is similar to the specimens collected by RV *Gerda* and RV *Pillsbury* stations which display a pedicel measuring ½ of the calicular diameter (Cairns 1979). However, Cairns (1979) highlights that these specimens were omitted from the description/account on the bases of immaturity and their small size. Species may have septa arranged in a decameral or hexameral fashion (Cairns 1979, 1991), the latter being the pattern observed in the imaged specimen reported herein (UCTES_NAD17F). Nonetheless, the South African record (UCTES_NAD17F) was collected at a much shallower depth than the previously known. Despite this, specimen is added to the account and represents a new record for the *Crispatotrochus* in the southwest Indian Ocean.

##### Desmophyllum

Taxon classificationAnimaliaScleractiniaCaryophylliidae

Ehrenberg, 1834

9F12EBE7-32E8-5E19-B4A0-5F363F626AB3

###### Diagnosis.

Solitary, trochoid, fixed. Pali absent. Columella absent or rudimentary. Sparse endothecal dissepiments.

###### Type species.

*Madreporadianthus* Esper, 1794, by subsequent designation (Cairns 1994).

##### Desmophyllum dianthus

Taxon classificationAnimaliaScleractiniaCaryophylliidae

(Esper, 1794)

FB71C335-0CC8-5DDB-B390-177D054727DA

Fig. 3G–J

Madrepora dianthus Esper, 1794: pl. 69, figs 1–3. –Esper 1795: 85–86. –Scheer 1990: 406.
Desmophyllum cristagalli Milne-Edwards & Haime, 1848a: 253, pl. 7, figs 10, 10a. –Milne-Edwards and Haime 1857: 76. –Duncan 1873: 321. –Pourtalès 1878: 203 (in part: Blake Stn. 2). –Pourtalès 1880: 96, 106 (in part: BL–288). –Verrill 1885: 150. –Agassiz 1888: 151. –Alcock 1902c: 28. –von Marenzeller 1904a: 267–268, pl. 15, fig. 2A–B. –Vaughan 1907: 67, pl. 7, figs 3, 3A–B. –Verrill 1908: 494. –Stephens 1909: 25. –Döderlein 1913: 126, pl. 8, figs 45, 45A. –Gravier 1920: 72–76 (in part), pl. 8, figs 130–135. –Gardiner 1929: 125–126. –Zibrowius 1974a: 758–761, pl. 3, figs 1–10. –Zibrowius et al. 1975: 98, pl. 4, fig. A–B. –Zibrowius and Grieshaber 1977: 379. – Zibrowius 1978: 535. –Zibrowius 1979: 19, pl. 1, figs 5–6. –Cairns 1979: 117–119, pl. 21, figs 7–8, pl. 22, fig. 8. –Zibrowius 1980: 117–121, pl. 61, figs A–O, pl. 62, figs A–M. –Cairns 1981: 10. –Cairns 1982: 29, pl. 8, figs 9–12, pl. 9, figs 1–3. –Zibrowius 1988: 136. –Zibrowius and Gili 1990: 35–36. –Cairns 1991: 17, pl. 6, figs G–I. –Cairns and Parker 1992: 28–29, fig. 8B–C. –Tyler and Zibrowius 1992: 227. –Cairns and Keller 1993: 246.
Desmophyllum capense Gardiner, 1904: 96–97. –Gardiner 1939: 329–330. –Wells 1958: 262. –Cairns 1979: 206.
Desmophyllum capensis . –Squires 1961: 23, fig. 5.
Desmophyllum dianthus . –Ehrenberg 1834: 299–300. –Milne-Edwards and Haime 1848a: 254–255. –Milne-Edwards and Haime 1857: 77–78. –Yabe and Eguchi 1942b: 113–114, pl. 9, figs 1–3. – Eguchi 1965a: 290. –Cairns 1994a: 26–27, pl. 9, figs 9A–D. –Cairns 1995: 77, pl. 21, figs D–F. –Cairns and Zibrowius 1997: 131, fig. 17G–H. –Cairns 1998: 385–386. –Cairns 1999a: 104–105. –Cairns et al. 1999: 22. –Stolarski 2003: 508, fig. 7A–G. –Cairns 2004a: 281. –Cairns 2006: 47. –Kitahara 2007: 502, 503, fig. 3K–L. –Pires 2007: 269. –Cairns 2009: 13. –Kitahara and Cairns 2021: 520–523, figs 284D–E, 289.

###### Type Locality.

Sagami Bay, Japan, depth unknown (Cairns 2004a).

###### Type material.

The neotype is deposited at the NMNH (Cairns 1994a).

###### Material examined.

SAMC_A072968 (2 specimens): no locality data. SAMC_A072974 (2 specimens): Western margin, 168 km off Paternoster/173 km off Brak Estuary, 32°05'41.99"S, 16°19'47.99"E; 440 m. SAMC_A073013 (2 specimens): Eastern margin, 34 km from Coffee Bay/18 km off Ntlonyane Estuary, 32°17'23.99"S, 29°05'35.87"E; 340–450 m. SAMC_A073015 (2 specimens): Southern margin, 32 km from Mazeppa Bay/19 km off Mendu Estuary, 32°25'00.11"S, 28°58'18.11"E; 330–340 m. SAMC_A073263 (1 specimen): Eastern margin, Wright Canyon; 171 m. SAMC_A088918 (3 specimens): Southern margin, 23 km from Plettenberg Bay/25 km off Piesang Estuary, 34°16'15.60"S, 23°24'50.40"E; 95 m. SAMC_A088919 (2 specimens): Southern margin, 172 km from Agulhas/182 km off De Mond-Heuningnes Estuary, 36°20'22.20"S, 20°24'06.11"E; 166 m. SAMC_A088929 (1 specimen): Southern margin, 140 km off Agulhas/144 km Ratels off Bulura Estuary, 36°02'29.58"S, 19°41'24.61"E; 445–463 m. SAMC_A090122 (1 specimen): Eastern margin, 15 km south of Ponta Do Ouro/17 km off Kosi-Kumpungwini (Sifungwe) Estuary, 26°55'30.00"S, 33°01'05.88"E; 370 m. SAM_A090128 (1 specimen): Western margin, 46 km from Paternoster/71 km off Berg River V Estuary Estuary, 32°56'32.93"S, 17°25'14.16"E; 325 m. SAM_A090130 (1 specimen): Western margin, 147 km from Groen River/144 km off Brak Estuary, 31°40'45.59"S, 16°23'07.80"E; 360 m. SAM_A090131 (1 specimen): Southern margin, 74 km from Agulhas/79 km off Ratels Estuary, 35°27'43.79"S, 19°51'20.99"E; 154 m. SAM_A090132 (1 specimen): Western margin, 86 km from Hondeklipbaai/84 km off Spoeg Estuary, 30°47'55.25"S, 16°34'37.98"E; 235 m. SAM_A090133 (1 specimen): Western margin, 113 km from Groen River/114 km off Groen Estuary, 31°13'55.85"S, 16°34'37.98"E; 310 m. SAM_A090134 (1 specimen): Western margin, 195 km from Port Nolloth/194 km off Buffels Estuary, 30°04'07.79"S, 15°05'26.69"E; 393 m. SAM_A090135 (1 specimen): Western margin, 165 km from Port Nolloth/165 km off Holgat Estuary, 29°45'08.82"S, 15°16'20.22"E; 183 m. SAMC_A090151 (1 specimen): Southern margin, 116 km from Knysna/ off Goukamma Estuary, 35°07'11.27"S, 23°02'41.91"E; 333 m. **SAM_H1475 (1 specimen)**: Western margin, 3 km from Pringle Bay/6 km off Buffels Oos Estuary, 34°23'11.29"S, 18°49'49.39"E; 80 m. SAM_H3049 (2 specimens): Eastern margin, 20 km off Cape Vidal/23 km off St Lucia Estuary, 27°59'30.00"S, 32°40'47.99"E; 550 m. SAM_H3050 (1 specimen): Eastern margin, 16 km from Margate/off Boboyi Estuary, 30°52'59.99"S, 30°31'00.00"E; 850 m.

###### Imagery data.

BMNH 1939.7.20.218 (2 specimens), MCZ (1 specimen): locality data unknown. SS ‘Pickle’ stn. 1480 (1 specimen): Western margin, off Hout Bay; 131 m.

###### Description.

Corallum variable, ranging from serpentine to ceratoid, attached to substrate by a pedicel (PD:GCD = 0.3–0.8) that expands into an encrusting base. Calice elliptical (GCD:LCD = 1.1–2.5), calicular margin serrate. Largest specimen examined (SAMC_A088919) 57.4 × 22.8 mm in CD, 30.0 mm in PD, and 119.3 mm in H. Theca granular. Costae more prominent near calicular margin, disappearing towards base. Corallum white to beige.

Septa hexamerally arranged in five complete cycles according to formula: S_1–2_ > S_3_ > S_4_ > S_5_ (96 septa). S_1–2_ most exsert, extend furthest to fossa (sometimes almost meeting opposite septa), with vertical and straight axial margins. S_3_ half as exsert and ⅓ smaller than S_1–2_, also with vertical and straight axial margin. S_4_ least exsert septa and ^4^/_5_ the size of S_3,_ with straight to slightly sinuous axial margin. S_5_ more exsert than S_4_ and fuses to adjacent S_1_–_3_ at calicular margin. S_5_ ~ ^1^/_2_ the width of S_4_, with straight upper axial margins that become sinuous deeper in fossa. Fossa deep, columellar absent.

###### Distribution.

Regional: Western to eastern margin of South Africa, off Lambert’s Bay extending towards Kosi-Kumpungwini (Sifungwe) Estuary (15 km south of Ponta Do Ouro: Mozambique); 80–850 m. Elsewhere: Cosmopolitan except from continental Antarctica and Boreal Pacific (Cairns 1994a); 8–2460 m.

###### Remarks.

*Desmophyllumdianthus* is the most well-studied azooxanthellate solitary coral. The species was first reported in South Africa by Gardiner (1904) sample SAM_H1475 off Cape Hangklip (False Bay area) at 80 m deep.

##### Desmophyllum pertusum

Taxon classificationAnimaliaScleractiniaCaryophylliidae

(Linnaeus, 1758)

CC7DA842-F3E4-54F0-AF85-2A3BCA1BE9E1

Fig. 3K, L

**Figure 4.**
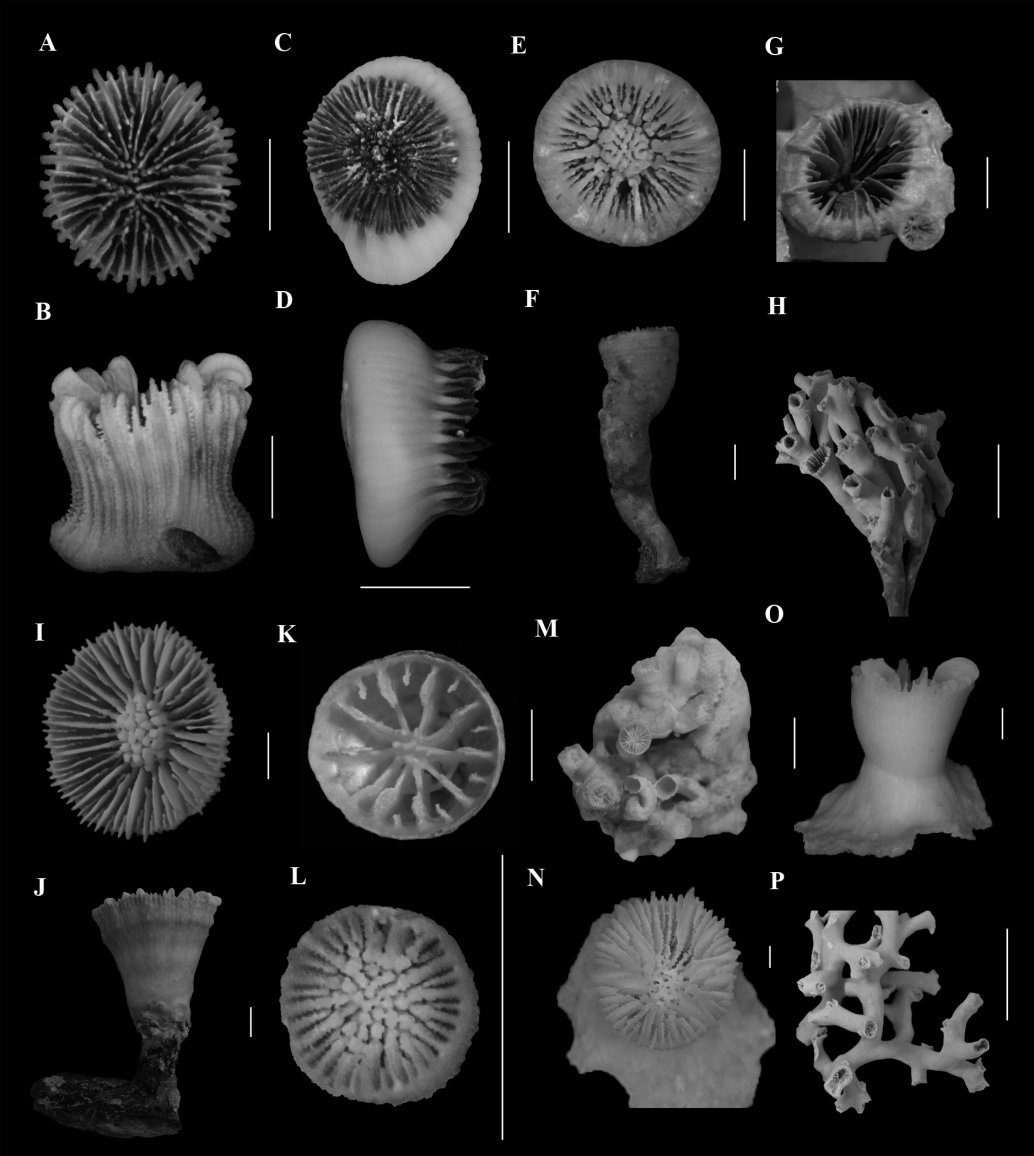
**A**, **B***Heterocyathusalternatus* (ORI_DIIIe1_1, locality data unknown) **A** calicular view **B** lateral view **C**, **D***Heterocyathusmonileseptatum* sp. nov. (SAM_H1431, off Durban Harbour, 99 m) **C** calicular view **D** lateral view **E**, **F***Heterocyathussulcatus* (SAMC_A073123, off Shaka’s Rock, 100–105 m) **E** calicular view **F** lateral view **G**, **H***Labyrinthocyathusdelicatus* (SAM_H2836, off East London, 146–238 m) **G** calicular view **H** lateral view **I**, **K***Monohedotrochuscapensis* comb.nov. **I**, **J** (SAMC_A088924, off Kidds Beach, 247–147m) **I** calicular view **J** lateral view **K** (SAM_H3210, off Scottburgh, 690 m) calicular view **L**, **M***Polycyathus* sp. (USNM 91677, off Port Dunford, 69 m) **L** calicular view **M** full view **N**, **O***Rhizosmiliarobusta* (USNM 91689, off Kosi Bay Estuary, 74 m) **N** calicular view **O** lateral view **P***Solenosmiliavariabilis* (SAM_H3158, off Cintsa, 630 m) **P** full view. Scale bars: 10 mm (**A–G**, **I–O**); 100 mm (**H**, **P**).

**Figure 5.**
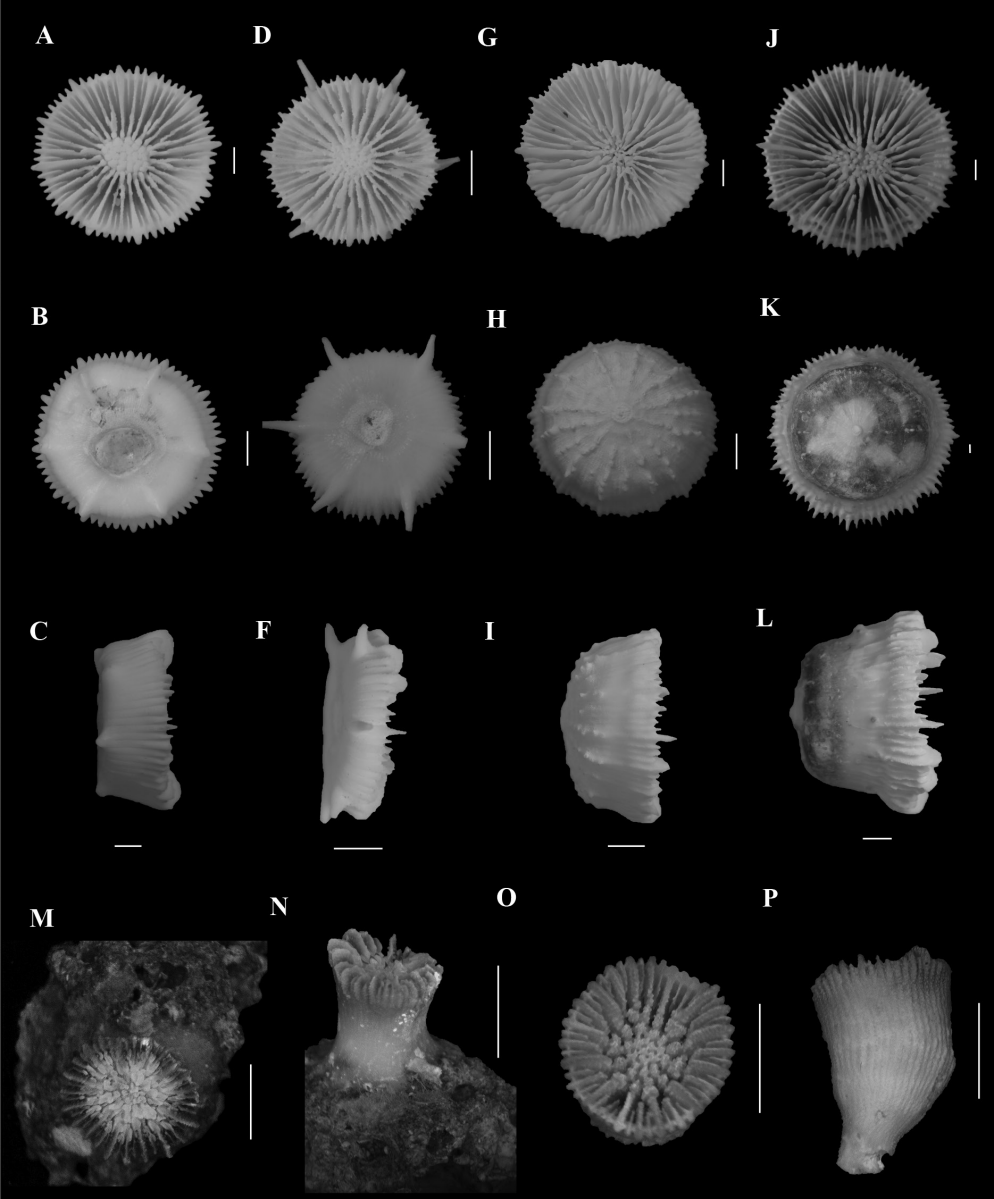
**A**, **F**Stephanocyathus (Acinocyathus) explanans: **A**, **C** (USNM 62500, off Durban, 183–220 m) Calicular view **B** basal view **C** lateral view **D**, **F** (SAMC_A090144, off Port St Johns, 96–98 m) **D** calicular view **E** basal view **F** lateral view **G**, **I**Stephanocyathus (Odontocyathus) campaniformis (SAM_H3178, off Coffee Bay, 1420 m) **G** calicular view **H** basal view **I** lateral view **J**, **L**Stephanocyathus (Odontocyathus) nobilis (SAM_H1697, off Groot Berg Estuary, 1050 m) **J** calicular view **K** basal view **L** lateral view **M**, **N***Tethocyathusvirgatus* (SAMC_A073180, off Mazeppa Bay, 240–250 m) **M** calicular view **N** lateral view **O**, **P**Trochocyathus (Trochocyathus) sp. 1 (SAM_H1244, off East London, 59 m) **O** calicular view **P** lateral view. Scale bars: 10 mm.

**Figure 6.**
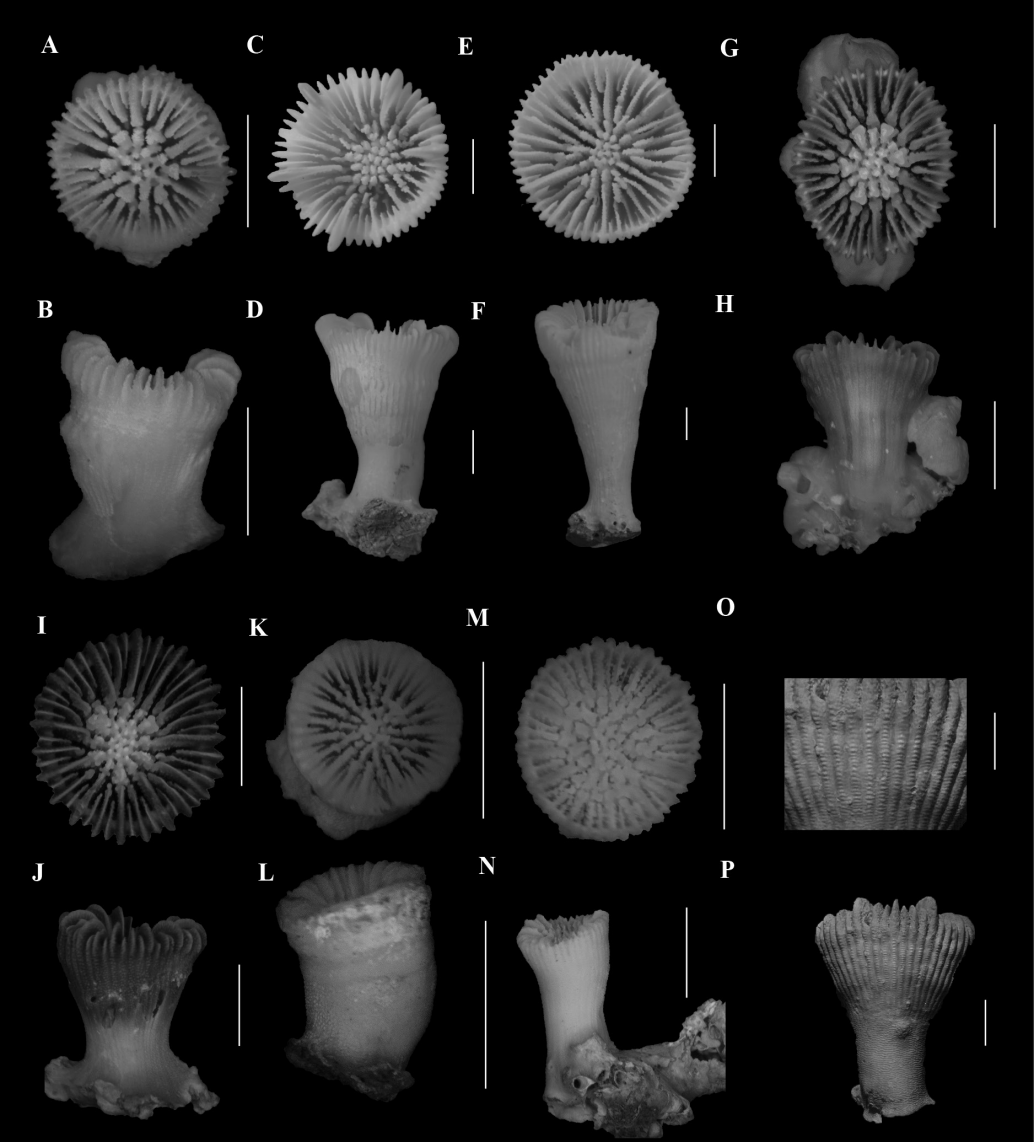
**A**, **B**Trochocyathus (Trochocyathus) sp. 1 (SAM_H1449, off Mazeppa Bay, 174 m) **A** calicular view **B** lateral view **C**, **F**Trochocyathus (Trochocyathus) sp. 2 **C**, **D** (SAM_H1388, East London, 90 m) **C** calicular view **C** lateral view **E**, **F** (SAM_H1407, off Mosselbaai, 192 m) **E** calicular view **F** lateral view **G**, **L**Trochocyathus (Trochocyathus) sp. cf. rawsonii sensu Cairns & Keller, 1993 **G**, **J** (SAM_H1440, off East London, 90 m) **G** calicular view **H** lateral view **I** calicular view **J** lateral view. 1993 **K**, **L** (SAMC_A090156, off Sedgefield, 74 m) **K** calicular view **L** lateral view **M**, **P**Trochocyathus (Trochocyathus) sp. 3 **M**, **N** (SAM_H3124, off East London, 90 m) **O** calicular view **P** lateral view **O**, **P** (BMNH 1939.7.20.47, off Richards Bay, 165 m) **O** calicular view **P** lateral view. Lateral view. Scale bars: 10 mm.

Madrepora pertusa Linnaeus, 1758: 797.
Madrepora prolifera Pallas, 1766: 307.
Lophelia prolifera . –Milne-Edwards and Haime 1850a: 81. –Cecchini 1917: 149. –Laborel 1970: 156. –Cairns 1979:125–127, pl. 24: figs 1–5. –Cairns 1982: 30–31, pl. 9: fig. 6. –Cairns 1991a:17–18, pl. 6: fig. J.
Lophohelia prolifera . –Milne-Edwards and Haime 1857: 117. –Pourtalès 1871: 24–25, pl. 1, figs 3–5. –Duncan 1873: 328–332, pl. 42, figs 7–8. –Moseley 1881: 178–179, pl. 8, figs 7–8 (not Challenger–109). –Verrill 1883: 63–64. –Agassiz 1888: 151, fig. 472. –Jordon 1895: 25. –von Marenzeller 1904a: 307, pl. 15, figs 3, 3A. –Gourret 1906: 121, pl. 11, fig. 10, pl. 12, fig. 10A. –Gravier 1920: 87–89 (in part: not pl. 10, fig. 157). –Nobre 1931: 67–68, pl. 19–20.
Lophelia affinis Pourtalès, 1868: 135.
Lophohelia tubulosa Studer, 1878: 631, pl. 1, fig. 8A–E
Bathelia candida . –Jourdan 1895: 27.
Lophelia californica Durham, 1947: 36, pl. 1: figs 13, 16; pl. 2: fig. 11. –Cairns 1991: 17.
Dendrosmilia nomlandi . –Durham and Barnard 1952: 85, pl. 10: fig. 47. –Cairns 1979: 126. –Bythell 1986:16, pl. 10: fig. F.
Desmophyllum cristagalli . –Squires 1959a: 18–22 (in part: figs 8–10).
Lophelia pertusa . –Zibrowuis 1974b: 761, pl. 2, figs 6–9. –Zibrowuis 1980: 126–130, pl. 66, figs A–L. –Zibrowius and Gill 1990: 36–38. –Cairns and Keller 1993: 218. –Cairns 1994: 27–28: pl. 9. figs E–I. –Cairns 2000: 100–102.
Desmophyllum pertusum Addamo et al., 2016: 10–11, fig. 1A, B, D, E, fig. 3F–I.

###### Type locality.

Southern California, depth unknown (Cairns 1994a).

###### Type material.

The type is presumed lost.

###### Material examined.

SAMC_A072974 (2 fragments): Western margin, 168 km off Paternoster/173 km off Brak Estuary, 32°05'41.99"S, 16°19'47.99"E; 440 m. SAMC_A088910 (1 fragment): Southern margin, 92 km from Oubosstrand/89 km off Tsitsikamma Estuary, 34°53'21.93"S, 24°06'56.47"E; 355 m. SAMC_A088911 (1 fragment): Southern margin, 92 km from Oubosstrand/89 km off Tsitsikamma Estuary, 34°53'21.93"S, 24°06'56.47"E; 355 m. SAMC_A088912 (1 fragment): Southern margin, 92 km from Oubosstrand/89 km off Tsitsikamma Estuary, 34°53'21.93"S, 24°06'56.47"E; 355 m. SAMC_A088914 (1 fragment): Southern margin, 92 km from Oubosstrand/89 km off Tsitsikamma Estuary, 34°53'21.93"S, 24°06'56.47"E; 355 m. SAMC_A088915 (1 fragment): Western margin, 53 km from Saldanha/80 km off Berg River V Estuary, 33°06'35.77"S, 17°23'01.26"E; 375 m. SAM_A090136 (1 specimen): Western margin, 162 km from Hondeklipbaai/159 km off Spoeg Estuary, 31°10'25.80"S, 15°54'55.79"E; 434 m. SAM_H1605 (5 fragments): Western margin, 43 km off Melkbosstrand/7 km off Dwars (North) Estuary, 33°24'39.01"S, 18°10'11.80"E; depth unknown. SAM_H1608 (6 fragments): Western margin, 13 km from Cape Town/6 km off Diep Estuary, 33°52'59.66"S, 18°25'34.08"E; depth unknown. SAM_H3129 (8 fragments): Eastern margin, 30 km off Coffee Bay/20 km off Bulungulu Estuary, 32°15'00.00"S, 29°09'06.00"E; 500–520 m.

###### Description.

Colony dendroid to bushy (branching pattern variable). Majority of budding intratentacular. Slender terminal branches bearing sympodially arranged corallites. Calicular size variable, reaching ≤ 15.6 × 9.0 mm in CD. Calice circular to elliptical (GCD:LCD = 1.0–1.3), with slightly serrated calicular margin. Peritheca finely granular, resulting in a smooth texture. Costae short and ridged, generally corresponding to primary septa. Corallum white.

Septa not arranged in regular systems nor cycles. Seven to nine slightly exsert primary septa extend deep into fossa. Secondary septa slightly less exsert and less wide than primaries, sometimes also extending deep into fossa. Tertiaries smaller than secondary septa, being rudimentary deep in fossa. Tertiaries sometimes missing in some systems. All septa with vertical and straight axial margins. Fossa deep and curved. Columella absent.

###### Distribution.

Regional: Western to eastern margin of South Africa, extending from off Clanwilliam extending towards Coffee Bay; 350–520 m. Elsewhere: Cosmopolitan in temperate and tropical waters (Zibrowius and Gill 1990; Cairns 2000), being common in the Atlantic and rarely collected off the Indo-Pacific (Cairns 1999b); 60–2170 m.

###### Remarks.

Recent molecular studies suggest that *Lophelia* is a synonym of *Desmophyllum* (Addamo et al. 2012, 2016). Such findings are demonstrated by the overwhelming genetic similarities between *L.pertusa* and *D.dianthus*, however, we recommend the sequencing of additional genes as a priority for future studies pertaining to this name change. *Lopheliapertusa* is one of the most well-known and studied azooxanthellate framework-building coral and was first reported in South African waters by Zibrowius and Gill (1990), who did not list locality data.

##### Goniocorella

Taxon classificationAnimaliaScleractiniaCaryophylliidae

 Yabe & Eguchi, 1932

3D2CEC78-4B75-5812-9E8C-EEED94A6C11B

###### Diagnosis.

Colonial, extra-tentacular budding forming bushy colonies. Branch anastomosis common, the branches also united by slender, tubular coenosteal bridges. No pali nor columella. Tabular endothecal dissepiments common and widely spaced.

###### Type species.

*Pourtalosmiliadumosa* Alcock, 1902c, by original designation.

##### Goniocorella dumosa

Taxon classificationAnimaliaScleractiniaCaryophylliidae

(Alcock, 1902)

64E90238-927D-5C5E-BE66-2860085B119E

Fig. 3M, N

Pourtalosmilia dumosa Alcock, 1902c: 36–37, pl. 5, fig. 33.
Goniocorella dumosa . –Yabe and Eguchi 1932a: 389–390. –Eguchi 1965b: 291, 2 figs. –Cairns 1982: 31–34, pl. 9, figs 7–9, pl. 10, figs 1, 2. –Cairns and Keller 1993: 250. fig. 6E. –Cairns 1995: 80–81, pl. 22, figs E, F.

###### Type locality.

Banda Sea, Indonesia (HMS ‘Siboga’ stns. 156 and 259: 0°29'02.00"S, 130°05'03.00"E and 5°29'02.00"S, 132°52'05.00"E, respectively); 469–487 m (Alcock 1902c).

###### Type material.

The syntypes are deposited at the ZMA (Cairns 1994a).

###### Material examined.

SAMC_A088913 (1 fragment): Southern margin, 110 km from Oubosstrand/off Tsitsikamma Estuary, 35°02'25.19"S, 23°59'33.60"E; 915 m. SAMC_A090137 (1 fragment): Southern margin, 203 km from Gouritsmond/214 km off Goukou Estuary, 36°08'21.59"S, 22°23'39.59"E; 997 m. SAMC_A090138 (1 fragment): Southern margin, 35 km from Knysna/33 km off Goukamma Estuary, 34°21'01.79"S, 22°51'01.19"E; 87 m. SAMC_A090139 (1 fragment): Southern margin, 25 km from Jeffreys Bay/20 km off Gamtoos Estuary, 34°04'46.20"S, 25°11'24.60"E; 69 m. SAM_H3185 (2 fragments): Eastern margin, 20 km from Cape Vidal/23 km off St Lucia Estuary, 27°59'30.00"S, 32°40'47.99"E; 550 m. SAM_H3186 (2 fragments): Southern margin, 31 km from Port Alfred/20 km off Kleinemond Estuary, 33°39'24.00"S, 27°11'42.00"E; 86 m. SAM_H3187 (1 fragment): Southern margin, 54 km from Port Edward/ km off Mdumbi Estuary, 33°00'00.00"S, 30°27'11.99"E; 900 m. SAM_H3188 (2 fragments): Eastern margin, 17 km from Margate/off Boboyi Estuary, 30°53'24.00"S, 30°31'41.99"E; 850 m. SAM_H3189 (16 fragments): Eastern margin, 36 km off Port Shepstone/49 km off Mtentu Estuary, 30°43'11.99"S, 30°48'47.99"E; 900 m. **SAM_H3190 (14 fragment)**: Southern margin, 26 km from Kidds Beach/27 km off Ncera Estuary, 33°19'36.00"S, 27°52'23.99"E; 760 m.

###### Description.

Corallum bushy. New branches formed from extra-tentacular budding at right angles from parent branch. Colonies reinforced by coenosteal bridges, which unite adjacent branches. Coenosteum with low and round granules. Branches cylindrical and straight, with circular to slightly elliptical (GCD:LCD = 1.0–1.4) corallites. Costae poorly developed, terminal corallites with slightly ridged C_1–2_.

Septa hexamerally arranged in three cycles according to the formula: S_1_ > S_2_ > S_3._ Upper region of all septa usually narrower than lower part. S_1_ slightly exsert, with straight and vertical axial margins. S_2_^1^/_3_ the width of S_1_, also with straight and vertical margins. S_3_ rudimentary and bearing dentate axial margins. Fossa deep, usually filled with tabular endothecal dissepiments. Columella absent.

###### Distribution.

Regional: Southern to eastern margin of South Africa, Port Alfred extending towards Cape Vidal; 86–997 m. Elsewhere: Indonesia (Cairns and Zibrowius 1997); Japan (Yabe and Eguchi 1932a; Cairns 1994a); New Zealand (Cairns 1995); Antarctic and Sub-Antarctic regions (Eguchi 1965b; Cairns 1982); 100–760 m.

###### Remarks.

*Goniocorelladumosa* is known to contribute to the three-dimensional habitat structures in deep waters (Cairns 1982, 1995; Le Goff-Vitry et al. 2004). It is distinctive from the other framework-building caryophylliids (e.g., *Solenosmiliavariabilis* and *Lopheliapertusa*) in asexually reproducing by extra-tentacular budding, having coenosteal bridges that reinforce the colony, and also by having prominent ridges on terminal corallites. The species was first reported from South Africa by Cairns and Keller (1993), a sub-sample from their South African record was also examined (SAM_H3190) in the present study.

##### Heterocyathus

Taxon classificationAnimaliaScleractiniaCaryophylliidae

Milne-Edwards & Haime, 1848

09D93AC9-968B-58E0-B894-B4578F0706FF

###### Diagnosis.

Corallum free and usually encapsulating a gastropod or scaphopod shell inhabited by a sipunculan worm. Costae at lateral theca distinct and either equal or unequal in thickness. At base costae transform into granulations. Lower part of corallum shows a relatively large worm opening (occasionally two) and several small pores.

###### Type species.

*Heterocyathusaequicostatus* Milne-Edwards & Haime, 1848, by subsequent designation (Milne-Edwards & Haime, 1850b).

##### Heterocyathus aequicostatus

Taxon classificationAnimaliaScleractiniaCaryophylliidae

Milne-Edwards & Haime, 1848

FE6B17DD-53C2-53A6-8786-32CCA9905021

Fig. 3O, P

Heterocyathus aequicostatus Milne-Edwards & Haime, 1848a: 324, pl. 10, fig. 8. –Milne-Edwards and Haime 1857: 51. –Alcock 1893: 141. –Gardiner 1904: 105–112, 125 (in part), pl. 3, figs 1–11, 22–32, 39–43. –Gardiner 1905: 955. –Bourne 1905: 193–194, 213–226, pl. 3, pl. 4, figs 12–21. –Harrison and Poole 1909a: 898–899, pl. 85, fig. lA–F. –Harrison and Poole 1909b: 913. –Harrison 1911: 1026, pl. 58, fig. 12. –Faustino 1927: 83–87, pl. 8, figs 1–7. –Yabe and Eguchi 1932b: 443. –Gardiner and Waugh 1938: 186–187. –Umbgrove 1938: 265. –Eguchi 1941: 417. –Yabe and Eguchi 1941b: 213, fig. 6A, B. –Yabe and Eguchi 1941c: 270, figs 3–4. –Crossland 1952: 102–103. –Durham and Barnard 1952: 87–88, pl. 11, fig. 49A–D. –Scheer and Pillai 1983: 158, pl. 36, fig. 9. –Wells 1984: 310, fig. 4.1. –Zibrowius and Grygier 1985: 121. –Pillai 1986: 188. –Veron 1986: 558–559 (in part). –Hu 1988: 146, 147, pl. 3, figs 9, 12, 13, 16, 17. –Hoeksema and Best 1991: 226–230, figs 1–11. –Hodgson and Carpenter 1995: 243. –Cairns 1998: 382–384, fig. 3A, B. –Cairns et al. 1999: 22. –Veron 2000: 412–413, figs 1–4. –Stolarski et al. 2001: 324, figs 6A–D. –Cairns 2004a: 281. –Cairns 2009: 12. –Kitahara and Cairns 2021: 528–529, 531, figs 290, 291A–G.
Heterocyathus roussaeanus Milne-Edwards & Haime, 1848a: 324–325, pl. 10 fig. 9, 9A. –Vaughan and Wells 1943: pl. 41 figs 16, 16A.
Heterocyathus cochlea . –Gray 1849: 77, pl. 2, figs l, 2A. –Gray 1850: 410.
Psammoseris hemispherica . –Milne-Edwards and Haime 1851: 127. –Veron 1986: 610.
Stephanoseris rousseaui . –Milne-Edwards and Haime 1851: 127.
Stephanoseris lamellosa Verrill, 1865: 149.
Heterocyathus philippinensis Semper, 1872: 254 (in part: pl. 20 figs 13, 14). –Eguchi 1941a: 414.
Heterocyathus japonicus . –Yabe and Eguchi 1942b: 127–128, pl. 11 fig. 6 A, B.

###### Type locality.

Unknown.

###### Type material.

The type material was never traceable (Cairns 2004a).

###### Material examined.

SAMC_A073100 (5 specimens): Eastern margin, 414 km south of Ponta Do Ouro/41 km off Groot Berg Estuary, 27°13'30.00"S, 32°49'30.00"E; 78 m. SAMC_A073106 (1 specimen): Eastern margin, 66 km from Cape Vidal/7 km off Mgobezeleni Estuary, 27°33'11.88"S, 32°43'00.12"E; 140 m. SAMC_A073186 (1 specimen): Eastern margin, 20 km from Durban/13 km off Mbokodweni Estuary, 30°01'05.87"S, 31°03'11.88"E; 150 m. USNM 90840 (1 specimen): 26 km from Port St. Johns/off Bulolo Estuary, 29°34'47.99"S, 31°41'59.99"E; 138 m.

###### Description.

Corallum free, squat and always encapsulating a gastropod shell colonised by a sipunculid worm. Sipunculid efferent pore < 2 mm in diameter, located aborally. Base flat, but area with pore more prominent, giving a pear-shaped appearance. Calice slightly elliptical (GCD:LCD = 1.1). Largest specimen examined (SAMC_A073186) 8.2 × 7.8 mm in CD, and 4.6 mm in H. Costae equidistant, separated by narrow intercostal striae. Calicular margin lanceted. Corallum creamy.

Septa crowded and hexamerally arranged in four cycles according to the formula: S_1_ > S_2_ > S_4_ > S_3_ (48 septa). S_1_ most exsert, with rounded upper and oblique axial margins. S_1_ extend towards columella and bear a paliform lobe. S_2_ less exsert, ¾ the width of S_1,_ and bearing multiple paliform lobes, which extend towards columella. S_3_ least exsert and smallest septa, but bear ≤ five paliform lobes. S_4_ almost as exsert as S_2_, bearing a paliform lobe (P_4_) that fuses to adjacent P_3._ All paliform lobes (P_1–4_) inclined towards columella. All septa have straight axial margins. Septal and palar faces granulated. Fossa shallow, containing a papillose columella composed of 10–25 cylindrical elements which are indistinguishable from pali.

###### Distribution.

Regional: Eastern margin of South Africa, 19 km from Durban extending towards Groot Berg Estuary (414 km south of Ponta Do Ouro: Mozambique); 78–145 m. Elsewhere: Zanzibar (Gardiner and Waugh 1938); Australia (Cairns 1998); Japan (Verrill 1866); China; Thailand (Gray 1849); Philippines; and Indonesia (Hoeksema and Best 1991); New Caledonia (Kitahara and Cairns 2021); 0–268 m.

###### Remarks.

*Heterocyathusaequicostatus* resembles *H.alternatus* in septal formula (S_1_ > S_2_ > S_3_ > S_4_), septa having straight axial margins, and in S_3_ bearing ≤ five pali, but can be distinguished in S_1_ bearing only one palus as compared with bearing two to three paliform lobes as in *H.alternatus*. Differences in costae among the two species may also be a distinguishing feature: *H.aequicostatus* has same sized costae as compared with those of *H.alternatus* sensu  Hoeksema and Best 1991 that vary in size (with C_1–2_ sometimes being slightly narrower than other costae). *Heterocyathusaequicostatus* was first reported off South Africa by Gardiner (1904), who detailed the intraspecific variation by comparing the eastern margin to the western margin representatives. The examined eastern margin South African representatives add no new taxonomic knowledge to what is already known apart from extending the depth range reported by Hoeksema and Best (1991). Additionally, specimens examined include zooxanthellate representatives (> 40 m) (Hoeksema and Best 2015) and should therefore not be considered in biodiversity assessments focusing on azooxanthellate forms.

##### Heterocyathus alternatus

Taxon classificationAnimaliaScleractiniaCaryophylliidae

Verrill, 1865

C133DCF3-DDDA-5ECD-B63F-0BBBC95B4D16

Fig. 4A, B

Heterocyathus alternata Verrill, 1865: 149
Heterocyathus alternatus . –Folkeson 1919: 10–11, pl. 1, figs 10–11. –Hoeksema and Best 1991: 230–231, figs 12–18. –Cairns 1998: 384, fig. 3D–E. –Cairns 1999a: 99–100, fig. 14E–F. –Cairns 2004a: 281.
Heterocyathus aequicostatus . –Boshoff 1981: 37 (in part).

###### Type locality.

Gaspar Straits, between the Bangka and Belitung Islands, Sumatra, (Indonesia); depth unknown (Hoeksema and Best 1991).

###### Type material.

The holotype is deposited at the YPM (Hoeksema and Best 1991).

###### Material examined.

SAMC_A073105 (1 specimen): Eastern margin, 36 km from Cape Vidal/32 km off Mgobezeleni Estuary, 27°48'54.00"S, 32°38'24.00"E; 52 m. SAMC_A073126 (5 specimens): Eastern margin, 25 km from Cape Vidal/23 km off St Lucia Estuary, 27°54'42.11"S, 32°36'42.11"E; 42–50 m. SAMC_A073214 (1 specimen): Eastern margin, 37 km from Cape Vidal/32 km off Mgobezeleni Estuary, 27°48'47.88"S, 32°38'53.87"E; 50 m. SAM_H2808 (10 specimens): Eastern margin, 20 km from Cape Vidal/22 km off St Lucia Estuary, 27°59'04.99"S, 32°40'08.00"E; 550 m. SAM_H3118 (19 specimens): Eastern margin, 2 km from Durban/8 km off Umgeni Estuary, 29°52'00.00"S, 31°00'00.00"E; 99 m. SAM_H3119 (1 specimen): Southern margin, 3 km from Plettenberg Bay/2 km off Piesang Estuary, 34°03'06.80"S, 23°22'48.65"E; 30–35 m. **ORI_DIIIe1_1 (1 specimen)**: Locality data unknown.

###### Description.

Corallum squat, free, and with aboral or lateral efferent pores smaller than 1.5 mm in diameter. Base flat, but a prominent pore result in an asymmetric corallum. Calice circular to elliptical (GCD:LCD = 1.0–1.1). Largest specimen examined (SAMC_A073126) 9.5 × 8.6 mm in CD, and 6.4 mm in H. Costae well-defined, finely granulated, and usually unequal in width. C_1–2_ sometimes slightly narrower than other costae. Intercostal striae relatively deep but disappear towards base. Base finely granulated. Central part of corallum darker than costae and associated septa.

Septa loosely packed and having a star-like appearance, hexamerally arranged in four complete cycles according to the formula: S_1_ > S_2_ > S_4_ > S_3_ (48 septa). S_1_ highly exsert, each bearing two or three paliform lobes. S_2_ slightly less exsert than S_1_. S_3_ smallest septa, not as exsert as S_1–2_, and bearing one or two paliform lobes. S_4_ wider than S_3_ and bears four or five paliform lobes, which are distinguishable from columellar elements. At calicular margin, S_4_ fuses to adjacent S_1–2_ forming prominent lancets. All septa appear to have straight axial margins, with granules arranged perpendicular to septal faces. All paliform lobes cylindrical, rising slightly above columellar elements, with traces of small and sparsely arranged granules. Fossa relatively deep, containing a papillose columella composed of 5–20 cylindrical elements.

###### Distribution.

Regional: Southern to eastern margin of South Africa, from Plettenberg Bay extending towards Cape Vidal; 30–150 m. Elsewhere: Off Indonesia (Hoeksema and Best 1991; Cairns 2004a); Vanuatu; and Wallis and Futuna Islands (Cairns 1999a); 0–319 m.

###### Remarks.

Examined specimens closely resemble specimens reported by Hoeksema and Best (1991) as *Heterocyathusalternatus*. However, some of the examined specimens (e.g., SAMC_A073126) have a darker centre and costal pigmentation, characters which Hoeksema and Best (1991) suggested to be representative of *H.sulcatus* (see key from Hoeksema and Best 1991 [page 222]). Cairns (1999) also noted specimens of *Heterocyathus* that are conspecific to *H.sulcatus*, but lacked pigmentation, and other specimens of *Heterocyathus* that display colouration but are distinctively different from *H.sulcatus*. Based on this, we suggest that colouration is of low taxonomic importance. Nonetheless, *H.alternatus* closely resembles *H.aequicostatus* among the South African congers. Apart from the differences in septa and costae profile highlighted in the *H.aequicostatus* account (see remarks section), the less crowded septa and star-like appearance (a result of S_4_ inclination towards S_3_) of *H.alternatus* distinguishes it from *H.aequicostatus*. Furthermore, the pali of *H.alternatus* are distinguishable from columellar elements in comparison to that of *H.aequicostatus*, which are indistinguishable. *Heterocyathusalternatus* was first reported in South Africa by Boshoff (1991), who identified it as *H.aequicostatus* (ORI_DIIIe1). Additionally, specimens examined include zooxanthellate representatives (> 40 m) (Hoeksema and Best 2015) and should therefore not be considered in biodiversity assessments focusing on azooxanthellate forms.

##### Heterocyathus monileseptatum

Taxon classificationAnimaliaScleractiniaCaryophylliidae

Filander & Kitahara sp. nov.

CA38ACB2-ED2D-5984-9219-8DB52D525B58

http://zoobank.org/5020B866-48D5-4686-8BDA-7769397C7D5A

Fig. 4C, D

###### Type locality.

Off Durban Harbour, South Africa, 99 m.

###### Type material/.

***Holotype*.** SAM_H1431A: eastern margin, 5 km from Durban/7 km off Umgeni Estuary, (RV ‘Pieter Faure’: 29°52'00.00"S, 31°03'00.00"E); 99 m. ***Paratypes*.** SAM_H1431B (4 specimens): eastern margin, 5 km from Durban/7 km off Umgeni Estuary, (RV ‘Pieter Faure’: 29°52'00.00"S, 31°03'00.00"E); 99 m. SAM_H1246 (8 specimens): Locality data unknown.

###### Etymology.

The species name *monileseptatum* (derived from Latin *monile* meaning “string of beads” and *septum* meaning “enclosure, wall, fence”) which alludes to the beaded septal margins.

###### Description.

Corallum unattached and tall, with lateral efferent pores ranging in diameter from 1.00 ≤ 2.00 mm. Base flat, but a prominent basal pore results in an asymmetric corallum with an irregularly shaped base (BD = 1.1–1.6). Calice circular to elliptical (GCD:LCD = 1.0–1.1), with serrate calicular margin. Holotype (SAM_H1431) 11.0 × 9.7 mm in CD, 11.1 × 6.9 mm in BD, and 10.6 mm in H. Paratypes having one or two aboral pores randomly positioned. Costae equidistant and progressively decreasing in width towards base. All costae finely granulated. C_4_ bears distinctive low spine-like granules. At base each costae become a row of granules. Intercostal striae equal in width and depth. Corallum white, with theca and columella with blackish pigment.

Septa thin, spaced out, delicate, and hexamerally arranged in four complete cycles (which follow a Pourtalès plan) according to the formula: S_1_ ≥ S_2_ > S_4_ > S_3_ (48 septa). S_1_ most exsert and extend to columella with straight and smooth axial margins. S_2_ only slight less exsert and ca. as wide as S_1_. S_2_ axial margins slightly sinuous. S_3_ least exsert septa and ^2^/_3_ the size of S_2_. In each half-system, a pair of S_4_ joins common S_3_ deep in fossa, and extends towards columella as one septum. S_3–4_ junctions beaded. S_4_ more exsert than S_3_, and also with sinuous axial margin. S_4_ dimorphic in size: those adjacent to S_1_ being wider and more exsert than those adjacent to S_2._ At calicular margin, S_4_ fuses to adjacent S_1–2_ forming high rectangular lancets. Septal faces granular. No pali. Fossa deep, containing a poorly developed papillose columella composed of ≤ seven sparsely arranged rods.

###### Distribution.

Regional: Eastern margin of South Africa, off Durban; 99 m.

###### Remarks.

Amongst the six extant *Heterocyathus* species (*H.aequicostatus*, *H.alternatus*, *H.antoniae* Reyes, Santodomingo & Cairns, 2009, *H.hemisphaericus*Gray 1849, *H.japanicus* (Verrill, 1866), and *H.sulcatus*) (Hoeksema and Cairns 2021), the specimens reported herein are distinctive in the lack of pali, height of corallum, and by having a beaded axial margin at the S_3–4_ junctions. However, there are intermediate similarities with each of these species, for example *Heterocyathusmonileseptatum* sp. nov. resembles *H.aequicostatus*, *H.alternatus*, and *H.hemisphaericus* in having septa arranged in four cycles (S_1_ > S_2_ > S_4_ > S_3_ = 48 septa), but differs from the first two species in having no pali rather than P_1–4_ (in case of *H.aequicostatus*) or P_1, 3, 4_ (in case of *H.alternatus*). On the contrary, the lack of pali in *Heterocyathusmonileseptatum* sp. nov. is a similarity shared with *H.hemisphaericus* but this species can be distinguished by their septa profile: all septa are thin (max 0.6 mm) and solid in *Heterocyathusmonileseptatum* sp. nov. compared with being thick (max 0.9 mm) and porous in *H.hemisphaericus* (Cairns 1998). The maximum height observed in *Heterocyathusmonileseptatum* sp. nov. (10.9 mm) is higher than that reported in the Atlantic *H.antoniae* (9.0 mm) and both species also have septa arranged in a Pourtalès plan fashion. Nonetheless, differences between *Heterocyathus* sp. nov. and *H.antoniae* include (i) septa being arranged according to S_1_ > S_2_ > S_4_ > S_3_ and S_1_ = S_2_ = S_4_ >> S_3_, respectively; (ii) lack of pali in *Heterocyathusmonileseptatum* sp. nov.; (iii) columella of *Heterocyathusmonileseptatum* sp. nov. being papillose and composed of sparsely arranged rods versus a spongy columella composed of crispate elements in *H.antoniae*. *Heterocyathusjapanicus* is also reported to have septa arranged in a Pourtalès plan (Zibrowuis 1997). However, *Heterocyathusmonileseptatum* sp. nov. can be differentiated by beaded S_3–4_ axial margins, a feature that is unique in relation to all *Heterocyathus*.

##### Heterocyathus sulcatus

Taxon classificationAnimaliaScleractiniaCaryophylliidae

(Verrill, 1866)

8AEDD6A5-3A00-540C-BF7F-148403D787E2

Fig. 4E, F

Stephanoseris sulcata Verrill, 1866: 48. –Vaughan 1905: 416.
Psammoseris cyclicioides Tenison-Woods, 1879 (in part): 10–11, pl. 1, figs 1–5. –Tenison-Woods 1880: 299–300.
Heterocyathus pulchellus Rehberg, 1892: 8–9, pl. 1, fig. 7A–B.
Homophyllia incrustans Dennant, 1906: 161, pl. 6, fig. 3A–B.
Heterocyathus aequicostatus . –Folkeson 1919: 8–10 (in part), pl. 1, figs 4–7. –Boshoff 1981: 37 (in part).
Heterocyathus cyclicioides . –Wells 1964: 109.
Heterocyathus sulcatus . –Hoeksema and Best 1991: 231–233, figs 19–23. –Cairns 1998: 384. –Cairns 1999a: 98–99, figs A–D. –Cairns et al. 1999: 22. –Stolarski et al. 2001: 320. –Randall 2003: 135. –Cairns 2004a: 281–282, fig. 3K. –Cairns 2009: 13. –Kitahara and Cairns 2021: 531–533, figs 291H–J, 292.

###### Type locality.

Off Ceylon, Sri Lanka, depth unknown (Verrill 1866).

###### Type material.

The holotype is deposited at the YPM (Verrill 1866).

###### Material examined.

SAMC_A073054 (1 specimen): Eastern margin, 33 km from Richards Bay/39 km off Mlalazi Estuary, 29°04'00.00"S, 32°10'00.00"E; 50 m. SAMC_A073071 (1 specimen): Eastern margin, 9 km from Shaka’s Rock/13 km off Tongati Estuary, 29°34'23.87"S, 31°17'53.88"E; 60 m. SAMC_A073089 (1 specimen): Eastern margin, 67 km south of Ponta Do Ouro/14 km off Mgobezeleni Estuary, 27°26'12.11"S, 32°44'12.11"E; 55–60 m. SAMC_A073105 (1 specimen): Eastern margin, 36 km from Cape Vidal/32 km off Mgobezeleni Estuary, 27°48'54.00"S, 32°38'24.00"E; 52 m. SAMC_A073108 (1 specimen): Eastern margin, 42 km south of Ponta Do Ouro/27 km off Kosi Bay Estuary, 27°13'36.12"S, 32°49'18.11"E; 75 m. SAMC_A073123 (24 specimens): Eastern margin, 51 km from Shaka’s Rock/41 km off Zinkwasi Estuary, 29°30'17.99"S, 31°45'44.99"E; 100–105 m. SAMC_A073144 (1 specimen): Eastern margin, 35 km off Cape Vidal/32 km off St Lucia Estuary, 27°49'41.87"S, 32°38'12.11"E; 54 m. SAMC_A073156 (1 specimen): Eastern margin, 35 km from Cape Vidal/32 km off St Lucia Estuary, 27°49'41.87"S, 32°38'12.11"E; 54 m. SAMC_A073161 (1 specimen): Eastern margin, 26 km from Port St. Johns/off Bulolo Estuary, 31°49'59.99"S, 29°39'59.99"E; 140–145 m. SAM_H1245 (15 specimens), SAM_H1430 (1 specimen): Locality data unknown. SAM_H1472 (2 specimens): Eastern margin, 2 km from Durban/8 km off Umgeni Estuary, 29°52'00.00"S, 31°00'00.00"E; 99 m. SAM_H1512 (2 specimens): Eastern margin, locality data unknown; 55–165 m. SAM_H3112 (7 specimens): Eastern margin, 9 km off Shaka’s Rock/2 km off Tongati Estuary, 29°34'18.96"S, 31°11'05.25"E; 66 m. ORI_DIIIe1_2 (1 specimen): Locality data unknown.

###### Description.

Corallum unattached and variable in shape. All specimens examined encapsulate a gastropod shell. Shape of corallum correlates with size and shape of gastropod shell. Aboral efferent pore not exceeding 2.0 mm in diameter. Calice circular to elliptical (GCD:LCD = 1.0–1.1). Largest specimen examined (SAMC_A073161) 9.8 × 9.0 mm in CD, and 6.4 mm in H. Costae granulated, unequal in size with C_3–4_ wider than C_1–2_, and progressively diminishing in size towards base. Base smooth. Upper parts of corallum, columella, and S_1–2_ darker than other corallum elements.

Septa hexamerally arranged in four complete cycles according to the formula: S_1_ ≥ S_2_ > S_3_ > S_4_ (48 septa). S_1_ most exsert, extend to columella with sinuous axial margins and bear a well-developed pali (P_1_). S_2_ slightly less exsert and may be equal or less wide than S_1_. S_2_ also have sinuous axial margins bordered by a smaller pali. S_3_ least exsert, also with sinuous axial margins bordered by variable sized pali. S_4_ dimorphic in development: those adjacent to S_1_ being wider and more exsert than those adjacent to S_2_. Approximately ^1^/_2_ distance to columella each S_4_ fuses to adjacent S_1–2_ forming a V-shaped pattern. Pali cylindrical and bear meniane-like ridges. Fossa shallow, containing a papillose columella.

###### Distribution.

Regional: Eastern margin of South Africa, off Port St. Johns extending towards Kosi Bay Estuary (42 km south of Ponta Do Ouro: Mozambique); 50–164 m. Elsewhere: Indonesia (Hoeksema and Best 1991; Cairns 2004a); Australia (Cairns 1998); Vanuatu; Wallis and Futuna Islands (Cairns 1999a); New Caledonia (Kitahara and Cairns 2021); 11–351 m.

###### Remarks.

*Heterocyathussulcatus* differs from the other three South African congeners in having S_3_ > S_4_ as compared with S_4_ > S_3_ as in *H.aequicostatus*, *H.alternatus*, and *Heterocyathusmonileseptatum* sp. nov. The presence of meniane-like structures on the palar faces of *H.sulcatus* further differentiates it from the other South African representatives. Part of the specimens reported herein were identified by Boshoff (1981) as *H.aequicostatus*, thus this account serves as a first record for the species in South African territory.

##### Labyrinthocyathus

Taxon classificationAnimaliaScleractiniaCaryophylliidae

Cairns, 1979

92182952-9575-5CD5-88DC-C0089B0C5689

###### Diagnosis.

Corallum solitary, ceratoid to subcylindrical, and firmly attached. Costae poorly defined or composed of transverse epithecal ridges. Pali absent. Columella well developed and composed of an interconnected maze of lamellar plates.

###### Type species.

*Labyrinthocyathuslangae* Cairns, 1979, by original designation.

##### Labyrinthocyathus delicatus

Taxon classificationAnimaliaScleractiniaCaryophylliidae

(von Marenzeller, 1904)

E0300B90-81F7-5F6C-8F40-11E314A09FEF

Fig. 4G, H

Ceratotrochus delicatus von Marenzeller, 1904a: 302, pl. 18, fig. 18.
Cyathoceras cornu . –Gardiner 1904: 121–122.
Labyrinthocyathus sp. –Cairns 1979: 70, pl. 11, figs 10–11.
Paracyathus indicus . –Boshoff 1981: 38.
Labyrinthocyathus delicatus . –Zibrowuis and Gili 1990: 44. –Cairns and Keller 1993: 244.

###### Type locality.

Off the Agulhas Bank, South Africa (SS ‘Valdivia’ stn. 104: 35°16'00"S, 22°26'00"E); 155 m (von Marenzeller 1904a).

###### Type material.

Unknown.

###### Material examined.

DEFF_AI2–INV 135 (2 specimens): Eastern margin, 37 km from Cintsa/21 km off Cwili Estuary, 32°49'59.99"S, 28°30'00.00"E; 228 m. SAMC_A073158 (2 specimens): Eastern margin, 10 km from Port Edward/24 km off Bilanhlolo Estuary, 31°05'48.00"S, 30°18'47.99"E; 140 m. SAMC_A073173 (1 specimen): Eastern margin, 24 km from Coffee Bay/17 km off Mdumbi Estuary, 31°58'00.00"S, 29°22'59.99"E; 200 m. SAMC_A073180 (13 specimens): Southern margin, 33 km from Mazeppa Bay/24 km off Cwili Estuary, 32°45'47.88"S, 28°36'24.12"E; 240–250 m. SAM_H1482 (4 specimens): Southern margin, 11 km from East London/5 km off Gouda Estuary, 33°05'03.24"S, 27°49'33.40"E; 146–238 m. SAM_H2805 (1 specimen): Eastern margin, locality data unknown; 550 m. SAM_H2828 (1 specimen): Southern margin, 11 km from East London/5 km off Gouda Estuary, 33°05'03.24"S, 27°49'33.40"E; 146–238 m. SAM_H2832 (1 specimen): Southern margin, 3 km from Kei Mouth/off De Mond-Heuningnes Estuary, 32°42'31.81"S, 28°21'54.38"E; 159 m. SAM_H2834 (3 specimens): Southern margin, 25 km from Gonubie/24 km off Gqunube Estuary, 33°06'17.99"S, 28°10'59.99"E; 155 m. SAM_H2835 (20 specimens): Southern margin, 29 km from Cintsa/3 km off Morgan Estuary, 32°42'30.47"S, 28°22'07.88"E; 159 m. SAM_H2836 (7 specimens): Southern margin, 11 km from East London/5 km off Gouda Estuary, 33°05'03.24"S, 27°49'33.40"E; 146–238 m. SAM_H2837 (2 specimens): Southern margin, 11 km from East London/3 km off Buffalo Estuary, 33°00'53.67"S, 27°55'50.67"E; 128 m. SAM_H2845 (2 specimens): Eastern margin, 17 km from Margate/off Boboyi Estuary, 30°53'24.00"S, 30°31'41.99"E; 850 m. **SAM_H3131 (2 specimens)**: Southern margin, 40 km from Cintsa/29 km off Cwili Estuary, 32°55'00.00"S, 28°31'00.00"E; 630 m. SAM_H3132 (1 specimen): Eastern margin, 28 km from Coffee Bay/19 km off Bulungulu Estuary, 32°14'53.99"S, 29°10'23.99"E; 620–560 m. SAM_H3133 (7 specimens): Southern margin, 32 km off Mazeppa Bay/24 km off Kobole Estuary, 32°28'36.00"S, 28°58'48.00"E; 710–775 m. SAM_H3134 (1 specimen): Southern margin, 28 km from Mazeppa Bay/27 km off Kobole Estuary, 32°32'59.99"S, 28°55'00.00"E; 775–790 m. SAM_H4242 (1 specimen): Eastern margin, 11 km from Port St. Johns/15 km off Bulolo Estuary, 31°39'42.00"S, 29°38'59.99"E; 300–540 m. ORI_DIIIg1 (1 specimen): Eastern margin, other locality unknown.

###### Imagery data.

AB_357 E (1 specimen): Eastern margin, 34 km from Port Dunford/38 km off Mlalazi Estuary, 29°10'00.00"S, 32°04'59.99"E; 168 m. BMNH 1973.2.20.26 (2 specimens): Eastern margin, 22 km from Port Shepstone/21 km off Damba Estuary, 30°46'59.99"S, 30°40'00.00"E; 457 m.

###### Description.

Corallum solitary, ceratoid to trochoid, attached to substrate through a pedicel (PD:GCD = 0.3–0.5) that expands into an encrusting base. Calice circular to slightly elliptical (GCD:LCD = 1.0–1.1), calicular margin thick and slightly serrated. Largest specimen examined (SAM_H2836) 11.6 × 11.4 mm in CD, 4.8 mm in PD, and 17.8 mm in H. Theca bears thin transverse ridges composed of fine granules. Costae poorly developed, granular, separated by faint and narrow intercostal striae, and more prominent near calicular margin. Corallum white.

Septa hexamerally arranged in four cycles according to the formula: S_1–2_ > S_3_ > S_4_ (48 septa). S_1–2_ largest septa and equal to only slightly more exsert than S_2_, almost reaching columella. S_3_^4^/_5_ the size of S_1–2._ Higher cycle septa (S_3–4_) progressively less exsert. S_4_ ½ to ¾ the width of S_3_, with the least sinuous axial margin. All septal faces bear low rounded granules. Fossa moderately deep, containing a well-defined labyrinthiform columella composed of interconnected lamellar plates.

###### Distribution.

Regional: Southern to eastern margin, off East London extending towards Port Dunford; 128–790 m. Elsewhere: off south-eastern Mozambique (Cairns and Keller 1993); 155–1000 m.

###### Remarks.

*Labyrinthocyathusdelicatus* was described by von Marenzeller (1904a) based on specimens sampled from off the Agulhas Bank, South Africa. Subsequently, Gardiner (1904) also reported on specimens collected off the Agulhas region but as *Cyathocerascornu*. Zibrowius and Gili (1991) noted that Gardiner’s (1904) specimens of *C.cornu* were representatives of *L.delicatus*. As Gardiner’s (1904) specimens were not examined during the course of the present study, their identification remains tentative but in agreement with Zibrowius and Gili (1990) and Cairns and Keller (1993) as *L.delicatus*. Boshoff (1981) further reported the species in the region, but as *Paracyathusindicus* Duncan, 1889.

##### Monohedotrochus

Taxon classificationAnimaliaScleractiniaCaryophylliidae

Kitahara & Cairns, 2005

8D3BD564-B908-5605-9A09-11440E1FFD90

###### Diagnosis.

Corallum solitary, attached, straight, and elongate-conical to trochoid. Base monocyclic. Septotheca costate. Pedicel and base thick. Pali may be present, indistinguishable from columella. Columella papillose.

###### Type species.

*Monohedotrochuscapitolii* Kitahara & Cairns, 2005, by original designation.

##### Monohedotrochus capensis

Taxon classificationAnimaliaScleractiniaCaryophylliidae

(Gardiner, 1904) comb. nov.

E3DFB0B3-A37D-5E75-8F72-AE72D8CD380D

Fig. 4I–K

Caryophyllia capensis Gardiner, 1904: 113–114, pl. 1, fig. 4A–D. –Boshoff 1981: 36. –Zibrowius and Gill 1990: 44.
Desmophyllum cristagalli . –Boshoff 1981: 37.
Balanophyllia capensis . –Boshoff 1981: 40 (in part).
Paraconotrochus capensis . –Cairns and Parker 1992: 21.

###### Type locality.

Off East London, South Africa (33°03'00"S, 27°57'00"E); 59 m (Gardiner 1904).

###### Type material.

The holotype is lodged at the NMNH.

###### Material examined.

SAMC_A072992 (1 specimen): Southern margin, other locality data unknown. SAMC_A073228 (1 specimen): Southern margin, False Bay; depth unknown. SAMC_A073233 (1 specimen): Southern margin, 14 km from Cape Point/10 km off Buffels Wes Estuary, 34°13'59.99"S, 18°30'00.00"E; 42 m. SAM_A073245 (2 specimens): Eastern margin, 34 km off Coffee Bay/7 km off Ntlonyane Estuary, 32°15'11.99"S, 28°57'42.00"E; 47 m. SAMC_A088924 (1 specimen): Southern margin, 23 km from Kidds Beach/24 km off Ncera Estuary, 33°18'01.37"S, 27°51'30.58"E; 247–147m. SAMC_A088927 (1 specimen): Southern margin, 92 km from Oubosstrand/89 km off Tsitsikamma Estuary, 34°53'21.93"S, 24°06'56.47"E; 355 m. SAM_A090072 (1 specimen): Western margin, 13 km from Pringle Bay/off Buffels Oos Estuary, 34°18'36.00"S, 18°42'53.99"E; 71 m. SAMC_A090073 (1 specimen): Western margin, 23 km from Pringle Bay/20 km off Buffels Oos Estuary, 34°10'05.99"S, 18°47'03.00"E; 36 m. SAMC_A090074 (2 specimens): Eastern margin, 34 km from Coffee Bay/7 km off Ntlonyane Estuary, 32°15'11.99"S, 28°57'42.00"E; 47 m. SAMC_A090075 (1 specimen): Western margin, 49 km from Cape Point/51 km off Buffels Wes Estuary, 34°43'18.00"S, 18°12'29.99"E; 360–365 m. SAMC_A090076 (1 specimen): Southern margin, 16 km from Cape Point/off Buffels Wes Estuary, 34°15'00.00"S, 18°36'00.00"E; 51 m. SAMC_A090078 (1 specimen): Southern margin, 14 km from Cape Point/10 km off Buffels Wes Estuary, 34°13'59.99"S, 18°30'00.00"E; 42 m. SAMC_A090081 (1 specimen): Southern margin, 12 km from Pringle Bay/11 km off Buffels Oos Estuary, 34°16'59.99"S, 18°45'00.00"E; 58 m. SAMC_A090140 (1 specimen): Southern margin, 92 km from Oubosstrand/89 km off Tsitsikamma Estuary, 34°53'21.93"S, 24°06'56.47"E; 355 m. SAM_H1365 (2 specimens): Southern margin, 28 km from Port Alfred/3 km off Old Woman’s Estuary, 33°30'00.00"S, 27°08'59.99"E; 40 m. SAM_H1366 (1 specimen): Southern margin, 5 km from East London/3 km off Blind Estuary, 33°00'13.79"S, 27°56'59.99"E; 59 m. SAM_H1374 (7 specimens): Southern margin, 241 km from Agulhas/247 km off De Mond-Heuningnes Estuary, 36°40'00.00"S, 21°25'59.99"E; 200 m. SAM_H1406 (2 specimens): Western margin, off Somerset West; depth unknown. SAM_H1439 (1 specimen): Southern margin, 25 km from Gonubie/24 km off Gqunube Estuary, 33°06'17.99"S, 28°10'59.99"E; 155 m. SAM_H1474 (1 specimen): Southern margin, 11 km from East London/5 km off Gouda Estuary, 33°05'03.24"S, 27°49'33.40"E; 146–238 m. SAM_H3060 (1 specimen): Southern margin, 28 km from Gonubie/27 km off Buffalo Estuary, 33°09'29.99"S, 28°03'06.00"E; 86 m. SAM_H3061 (4 specimens): Southern margin, 28 km from Port Alfred/3 km off Old Woman’s Estuary, 33°30'00.00"S, 27°08'59.99"E; 93 m. SAM_H3210 (4 specimens): Eastern margin, 30 km from Scottburgh/20 km off Fafa Estuary, 30°33'24.00"S, 30°48'35.99"E; 690 m. SAM_H3211 (1 specimen): Eastern margin, 17 km from Margate/off Boboyi Estuary, 30°53'24.00"S, 30°31'41.99"E; 850 m. SAM_H3398 (2 specimens): Southern margin, 11 km from Gansbaai/38 km off Bot River Lagoon, 34°39'27.93"S, 19°17'03.00"E; 36 m. ORI_DIIIa2 (2 specimens), ORI_DIIId1 (2 specimens), ORI_EIa4_2 (5 specimens): Eastern margin, other locality data unknown. USNM 100854 (1 specimen): Southern margin, 5 km from Port Elizabeth/3 km off Bakens River Estuary, 33°56'52.31"S, 25°37'20.70"E; depth unknown.

###### Imagery data.

BMNH 1939.7.20.37–39 (1 specimen), BMNH 1950.1.11.63 (1 specimen): South Africa, other locality data unknown. SAM_H1377 (2 specimens): Southern margin, 28 km from Port Alfred/3 km off Old Woman’s Estuary, 33°30'00.00"S, 27°08'59.99"E; 93 m.

###### Description.

Corallum ceratoid to trochoid, firmly attached to substrate by a slender pedicel (PD:GCD = 0.3–0.7) that expands into a monocyclic encrusting base. Calice slightly elliptical (GCD:LCD = 1.1–1.3). Largest specimen examined (SAMC_A090140) 26.2 × 23.3 mm in CD, 9.6 mm in PD, and 48.1 mm in H. Costae flat to slightly convex, being prominent at calicular margin. Costae bear irregular and small granules. Intercostal striae thin and shallow. Both costae and intercostal striae become progressively faint towards base. Corallum white with theca being slightly light brown.

Septa hexamerally arranged in five cycles, sometimes the last cycle being incomplete, according to the formula: S_1–3_ > S_3_ > S_4_ > S_5_ (96 septa). S_1–2_ highly exsert and equal in width, extending to columella with straight axial margins. S_3_ less exsert, ^1^/_3_ less the width of S_1–2_, but in larger specimens meet columella deeper in fossa. However, in smaller specimens (< 13.0 mm in CD), S_3_ do not join columella and has a slightly dentate axial margin. S_4_ less exsert and ½ the width of S_3_, with dentate axial margin. S_5_ not exsert, rudimentary, with the most dentate axial margin. Fossa of moderate depth, containing a papillose columella composed of a group of 15–20 well-defined rods.

###### Distribution.

Regional: Western to eastern margin of South Africa, off Gansbaai extending towards Scottburgh; 36–365 m. Elsewhere: No other distributional records are known.

###### Remarks.

The species was first reported in South African boundaries by Gardiner (1904) as *Caryophylliacapensis*, and later tentatively placed in the genus *Paraconotrochus* (Cairns and Parker 1992). However, among the three genera (*Caryophyllia*, *Paraconotrochus*, and *Monohedotrochus*) only the latter lacks paliform lobes before S_3_. In addition, after examining Gardiner’s (1904: [fig. 4C]) illustrations, no pali were observed. Furthermore, the coralla of specimens examined display well-defined granular costae, whereas those of *Paraconotrochus* are generally smooth and costae absent or poorly defined. Based on these differences, we propose the new combination *Monohedotrochuscapensis* (Gardiner, 1904). Within the examined material some are represented by small specimens (i.e., SAM_H3210 [Fig. 4K] and SAM_H3211), which resemble *Gardineria* Vaughan, 1907 in having transversely wrinkled epitheca and outer septal margins being separated from calicular margin by a notch. However, juvenile *Monohedotrochuscapensis* and *Gardineria* species are difficult to distinguish and thus these two samples (SAM_H3210 and SAM_H3211) are added here with caution.

##### Polycyathus

Taxon classificationAnimaliaScleractiniaCaryophylliidae

Duncan, 1876

3FFF13B2-4E54-5145-AD34-085A736B4B0A

###### Diagnosis.

Corallum colonial. Corallites cylindrical to slightly conical bud from a common coenosteum or from stolons. Septotheca costate. Three to four cycles of septa. Pali present before all but last septal cycle. Columella papillose.

###### Type species.

*Polycyathusatlanticus* Duncan, 1876, by monotypy.

##### Polycyathus

Taxon classificationAnimaliaScleractiniaCaryophylliidae

sp.

7FDB45F0-80DE-5DF6-8C3D-3ECEFA7A49EF

Fig. 4L, M

###### Material examined.

**USNM 91677 (1 colony)**: Eastern margin, 33 km from Port Dunford/35 km off Mlalazi Estuary, 29°10'59.99"S, 32°01'59.99"E; 69 m.

###### Description.

The colony consists of 12 ceratoid to cylindrical corallites that reach ≤ 4.9 mm in H, and bud from a common coenosteum. Calice circular to slightly elliptical (GCD:LCD = 1.0–1.1), calicular margin thin and serrated. Theca glistening and covered by low-profile granules. Costae poorly developed. Corallum predominantly beige; but pali, columella, and base white.

Septa hexamerally arranged in four cycles, the last cycle being incomplete, according to the formula: S_1_ ≥ S_2_ > S_3_ > S_4_ (≤ 34 septa). S_1_ most exsert, and as wide or only slightly wider than S_2_. Both S_1–2_ with sinuous axial margin, bearing pali which are as thick as septa. S_3_ not exsert, ^3^/_4_ the width of S_1–2,_ and have vertical axial margin. S_4_ rudimentary. Septal faces granulated and slightly sinuous. Pali present and distinct in all but last septal cycle (14–16 pali). Half-systems with S_4,_ P_3_ sometimes join neighbouring P_2_ resulting in a V-shaped appearance. All paliform lobes terminate at same level and form a crown encircling columella. Paliform lobes sometimes indistinguishable from columellar elements. Fossa of moderate depth containing a papillose columella composed of a group of fairly spaced rods.

###### Distribution.

Regional: Eastern margin, Port Dunford; 69 m.

###### Remarks.

Although corallites of the examined colony are mostly damaged, features that characterise the genus are still distinguishable. However, more specimens are required to enable a thorough comparison to the other *Polycyathus* representatives. This colony represents a new record of the genus in South Africa.

##### Rhizosmilia

Taxon classificationAnimaliaScleractiniaCaryophylliidae

Cairns, 1978

B53863B2-4ED8-5BAC-8D7E-D6F5963DE855

###### Diagnosis.

Colonies formed by extra-tentacular budding from a common basal coenosteum. Corallite base increase in diameter by adding exothecal dissepiments over raised costae producing concentric rings of partitioned chambers that resemble polycyclic development. Septotheca costate and granular. Septal axial margins smooth. Paliform lobes occur before penultimate septal cycle. Columella papillose or lamellar. Vesicular endotheca present.

###### Type species.

*Rhizosmiliagerdae* Cairns, 1978, by original designation.

##### Rhizosmilia robusta

Taxon classificationAnimaliaScleractiniaCaryophylliidae

Cairns in Cairns & Keller, 1993

11AC3418-622C-56AF-BC31-35E26472402C

Fig. 4N, O

Rhizosmilia robusta Cairns in Cairns & Keller, 1993: 250–253, pl. 6, figs F–I. –Cairns and Zibrowius 1997: 133–134. –Cairns 1999a: 107. –Cairns et al. 1999: 24. –Cairns 2009: 14. –Kitahara et al. 2010a 108, figs 88–89. –Kitahara et al. 2010b: 9. –Kitahara and Cairns 2021: 554–556, figs 305D–I, 306.

###### Type locality.

Off Inhaca Island, Mozambique (RV ‘Anton Bruun’ stn. 373B: 26°00'00"S, 33°05'00"E); 135 m (Cairns and Keller 1993).

###### Type material.

The holotype is deposited at the NMNH (Cairns and Keller 1993).

###### Material examined.

**USNM 91689 (3 specimens)**: Eastern margin, 42 km south of Ponta Do Ouro/27 km off Kosi Bay Estuary, 27°13'30.00"S, 32°49'30.00"E; 74 m.

###### Description.

Adapted from Cairns and Keller 1993: Colony phaceloid. Corallites trochoid, firmly attached through a massive pedicle (PD:GCD = 0.03), that expands into a thin encrusting base. Lower pedicel reinforced by concentric rings of hollow chambers. Calice elliptical (GCD:LCD = 1.18), with a jagged calicular margin. Holotype (USNM 91681) 31 × 26.20 mm in CD, 16.80 mm in PD, and 25 mm in H. Costae equal and bearing low and rounded granules. Intercostal striae shallow. Corallum white.

Septa arranged in five cycles according to the formula: S_1_ > S_2_ > S_3_ > S_4_ > S_5_. S_1_ moderately exsert, and extend to columella with vertical to straight axial margins. Higher cycle septa (S_2–5_) progressively less exsert and less wide, except for those S_5_ adjacent to S_1_ which are more exsert than S_4._ Axial margins of S_2_ also straight, but those of S_3–4_ slightly sinuous. S_5_ rudimentary and irregular in profile (i.e., may be straight to slightly sinuous or sinuous to dentate). All septal faces smooth, bearing only sparsely spaced low-profile granules. Small pali present before penultimate cycle, usually indistinguishable from columellar elements. Fossa deep and bearing endothecal dissepiments. Columella trabecular, composed of an irregular group of intertwined elements.

###### Distribution.

Regional: Eastern margin of South Africa, off Kosi Bay Estuary (41 km south of Ponta Do Ouro: Mozambique); 75 m. Elsewhere: Mozambique; Madagascar (Cairns and Keller 1993); Philippines (Cairns and Zibrowius 1997); New Caledonia (Kitahara and Cairns 2021); 66–996 m.

###### Remarks.

As no new samples have been examined, the description above is adapted from Cairns and Keller (1993), who noted that although the holotype is a phaceloid colony with four corallites, paratypes (specimen examined and imaged herein) represent individual corallites that were broken apart from a larger colony or have not yet formed a colony. Variation in septal cycles correlates to corallum size: the fourth septal cycle is complete at a GCD between 8 and 9 mm, whereas the fifth is completed when the corallite is > 19–21 mm in GCD. The largest corallite (Philippines, see Cairns and Zibrowius 1997 [page 134]), measures 29.8 × 36.7 mm in CD, 18.1 mm in PD, 41.6 mm in H, have eight pairs of S_6,_ and one pair of S_7_ (total of 116 septa). Notable differences between *Rhizosmiliarobusta* from the Pacific and southwestern Indian Ocean are the position of paliform lobes, which are present before the penultimate cycle (P_4_, if S_5_ is present; and P_3_ if S_5_ is absent) in the latter and occur before S_4_ (irrespective of the presence of S_6–7_) in the former. This is the only representative of the genus in South African waters.

##### Solenosmilia

Taxon classificationAnimaliaScleractiniaCaryophylliidae

Duncan, 1873

5AD8D3C5-6BA2-567F-97FF-C5F15913FD99

###### Diagnosis.

Corallum firmly attached, colonial. Colony dendroid or sub-phaceloid formed by intratentacular budding. Stereome granular, costae sometimes correspond to first septal cycle. Tabular endothecal dissepiments. Columella small.

###### Type species.

*Solenosmiliavariabilis* Duncan, 1873, by monotypy.

##### Solenosmilia variabilis

Taxon classificationAnimaliaScleractiniaCaryophylliidae

Duncan, 1873

DA11729C-5353-598B-8BE0-8DB927E01B18

Fig. 4P

Solenosmilia variabilis Duncan, 1873: 328, pl. 42, figs 11–18. –Pourtalès 1878: 206. pl. 1, figs 1–3. –Pourtalès 1880: 108. –Moseley 1881: 181, pl. 9, figs 1–5. –von Marenzeller 1904: 310–311, pl. 15, figs 4, 4A. –Zibrowius 1974a: 768–769. –Gravier 1920: 94–96, pl. 9, figs 153–156. –Hoffmeister 1933: 14, pl. 4, fig. 7. –Gardiner and Waugh 1939: 229–230. –Cairns 1979: 136–138, pl. 26, figs 2–4. –Zibrowius 1980: 143–145, pl. 75, figs A–N.– Scheer and Pallai 1983: 160. –Cairns and Parker 1992: 29–30, pl. 8, figs D, E.– Cairns and Keller 1993: 250, fig. 6D. –Cairns 2004a: 284.
Solenosmilia jeffreyi Alcock, 1898: 27–28, pl. 3, fig. 3, 3A, B.

###### Type locality.

Off Spain (HMS ‘Porcupine’ stns. 17 and 32: 39°42'00"N, 9°43'00"W and 35°41'00"N, 7°08'00"W, respectively); 1190–2003 m (Duncan 1873).

###### Type material.

The syntype is deposited at the NHMUK (Cairns 1979, 2004a).

###### Material examined.

DSCS–INV 122 (1 fragment): Southern margin, 240 km from Agulhas/247 km off De Mond-Heuningnes Estuary, 36°45'34.13"S, 21°12'46.61"E; 513 m. SAMC_A088916 (1 fragment): Southern margin, 280 km from Cape St. Francis/287 km off Slang Estuary, 36°43'40.13"S, 25°08'53.47"E; 622 m. SAMC_A090142 (1 fragment): Southern margin, 240 km from Agulhas/247 km off De Mond-Heuningnes Estuary, 36°45'34.13"S, 21°12'46.61"E; 513 m. SAMC_A090143 (1 fragment): Southern margin, 287 km from Cape St. Francis/291 km off Slang Estuary, 36°47'35.77"S, 24°38'35.69"E; 520 m. SAM_H1397 (1 fragment): Southern margin, 3 km from East London/1 km off Buffalo Estuary, 33°01'29.99"S, 27°55'00.00"E; 566–928 m. SAM_H2807 (1fragment): Eastern margin, 20 km from Cape Vidal/22 km off St Lucia Estuary, 27°59'04.99"S, 32°40'08.00"E; 550 m. SAM_H2840 (14 fragments): Eastern margin, 19 km from Cape Vidal/22 km off St Lucia Estuary, 27°59'30.00"S, 32°40'00.00"E; 550 m. SAM_H3034 (13 fragments): Eastern margin, 36 km off Port Shepstone/49 km off Mtentu Estuary, 30°43'11.99"S, 30°48'47.99"E; 900 m. SAM_H3035 (10 fragments): Eastern margin, 16 km from Port Shepstone/off Boboyi Estuary, 30°49'05.99"S, 30°34'59.99"E; 930 m. SAM_H3036 (21 fragments): Eastern margin, 36 km from Port Shepstone/28 km off Mhlabatshane Estuary, 30°43'00.00"S, 30°48'47.99"E; 780 m. **SAM_H3037 (1 fragment)**: Eastern margin, 17 km from Margate/off Boboyi Estuary, 30°53'24.00"S, 30°31'41.99"E; 850 m. SAM_H3140 (17 fragments): Southern margin, 26 km from Kidds Beach/27 km off Ncera Estuary, 33°19'36.00"S, 27°52'23.99"E; 760 m. **SAM_H3141 (13 fragments)**: Southern margin, 32 km off Mazeppa Bay/24 km off Kobole Estuary, 32°28'36.00"S, 28°58'48.00"E; 710–775 m. SAM_H3142 (3 fragments): Southern margin, 22 km from Gonubie/21 km off Gqunube Estuary, 33°06'00.00"S, 28°08'17.99"E; 700–650 m. **SAM_H3158 (38 fragments)**: Southern margin, 40 km from Cintsa/29 km off Cwili Estuary, 32°55'00.00"S, 28°31'00.00"E; 630 m. SAM_H3179 (1 fragment): Southern margin, 31 km from Port Alfred/20 km off Kleinemond (Oos) Estuary, 33°39'24.00"S, 27°11'42.00"E; 86 m.

###### Description.

Corallum bushy achieved by intra-tentacular budding. Budding dichotomous. Calice rarely exceeds 6 mm in CD. Coenosteum completely smooth or granular. Costae ridged, with granules arranged in a longitudinal manner. Corallum white and sometimes light brown.

Septa hexamerally arranged in three or four cycles according to the formula: S_1_ > S_2_ > S_3_ > S_4._ S_1_ highly exsert and extend deep into fossa with straight axial margins. S_2_ less exsert and ^1^/_3_ the width of S_1,_ but otherwise similar in profile. S_3_ not exsert and forms in upper fossa, appearing rudimentary. S_4_ usually absent, but when present has dentate or laciniate axial margin. Septal faces bear tall and slender granules. Tabular endothecal dissepiments common and widespread. Columella usually absent, but some corallites have a small spongy columella deep in fossa.

###### Distribution.

Regional: Southern to eastern margin of South Africa, off Cape St. Francis extending towards Cape Vidal; 86–930 m. Elsewhere: Cosmopolitan, except from the continental Antarctica; 220–2165 m.

###### Remarks.

Together with *Madreporaoculata*, *Lopheliapertusa*, and *Goniocorelladumosa*, *Solenosmiliavariabilis* is one of the most studied framework-building coral species. The equal distomedial budding in *S.variabilis* makes it easily distinguishable from the other deep-water framework-building species. *Solenosmiliavariabilis* was first reported from South Africa off the Agulhas region (von Marenzeller 1904a). Subsequently, Cairns and Keller (1993) documented its northward regional distribution to off Durban. Part of the examined material includes sub-samples (SAM_H3037, SAM_H3141, and SAM_H3158) of Cairns and Keller’s (1993) records, all of which are broken fragments. Therefore, details on the size of the base and end branches are not included in the description. Nevertheless, Cairns (1979) provides a more detailed description of these features.

##### Stephanocyathus

Taxon classificationAnimaliaScleractiniaCaryophylliidae

Seguenza, 1864

A486C870-C29E-5831-AD4E-9EF50F296390

###### Diagnosis.

Solitary, patellate to bowl-shaped, and free. Costae usually well developed, some of which are sometimes highly spinose. Paliform lobes usually present on all septa. Columella trabecular, papillose, or a solid fusion of axial septal margins.

###### Type species.

*Stephanocyathuselegans* Seguenza, 1864, by subsequent designation (Wells 1936).

##### Stephanocyathus Acinocyathus

Taxon classificationAnimaliaScleractiniaCaryophylliidae

Wells, 1984

74B49396-5C02-50BE-AF87-93E7EA5F38B1

###### Diagnosis.

*Stephanocyathus* with six elongate spines corresponding to C_1_.

###### Type species.

*Stephanotrochusspiniger* von Marenzeller, 1888, by original designation.

##### Stephanocyathus (Acinocyathus) explanans

Taxon classificationAnimaliaScleractiniaCaryophylliidae

(von Marenzeller, 1904)

8D473D65-64A8-5D8A-849F-34848B4A079F

Fig. 5A–F

Stephanotrochus explanans von Marenzeller, 1940a: 304–307, pl. 8, fig. 19A, B. –Gardiner and Waugh 1938: 192.
Stephanocyathus nobilis . –Boshoff 1981: 39.
Stephanocyathus (Acinocyathus) spiniger . –Cairns and Keller 1993: 243–244.
Stephanocyathus (Acinocyathus) explanans . –Cairns and Zibrowius 1997: 119, fig. 14E. –Cairns 1998: 38. –Cairns 2004a: 285.

###### Type locality.

Off Sumatra, Zanzibar Island and Pemba (SS ‘Valdivia’ stns. 194, 243 and 245: 0°15'02"N, 98°08'08"E, 6°39'01"S, 39°30'08"E, and 05°27'09"S, 39°18'08"E, respectively); 245–614 m (von Marenzeller 1904a).

###### Type material.

Ten syntypes are deposited at the ZMB (Cairns 2004a).

###### Material examined.

SAMC_A090144 (2 specimens): Eastern margin, 8 km from Port St. Johns/11 km off Bulolo Estuary, 31°39'43.19"S, 29°36'38.16"E; 96–98 m. **ORI_DIIIk2 (I specimen)**: Eastern margin, 33 km from Durban/31 km off Beachwood Mangroves, 29°55'00.00"S, 31°19'59.99"E; 442 m. **USNM 62500 (4 specimens)**: Eastern margin, 11 km from Durban/4 km off Beachwood Mangroves, 29°46'29.16"S, 31°04'18.42"E; 183–220 m.

###### Imagery data.

RV ‘Galathea’ stn. 202 (1 specimen): Eastern margin, Natal.

###### Description.

Corallum bowl-shaped, free, with a slightly rounded base. BD smaller than CD; base has a basal attachment scar. All specimens bear short, straight, and slender C1 costal spines, and are usually longer (in relation to CD) in juveniles. Calice circular to slightly elliptical (GCD:LCD = 1.0–1.1), with a serrated calicular margin. Largest specimen examined (USNM 62500) 30.4 × 27.6 mm in CD, 26.6 in BD, and 11.2 mm in H. Costae slightly convex and granular, extending from calicular margin and disappearing towards base. Costae absent at base epicentre, which is usually eroded. Corallum white.

Septa hexamerally arranged in five cycles, the last cycle being incomplete, according to the formula: S_1_ ≥ S_2_ > S_3_ > S_4_ > S_5_ (≤ 72 septa). S_1_ highly exsert and extend to columella with straight axial margin. A deep notch separates P_1_ from S_1_. P_1_ fuse to columella. S_2_ as exsert and as large to only slightly smaller than S_1_. P_2_ also fuse to columella, but is slightly smaller than P_1_. S_3_ ~ 1 mm less exsert and ¾ the width of S_1–2_. P_3_ broad and separated from S_3_ by a narrow notch. S_4_ flanked by S_5_ as wide and exsert as S_3_, but those unflanked S_4_ are the least exsert septa, and only ½ the width of S_3_. S_4_ become rudimentary deeper in fossa. P_4_ fuse to P_3_. S_5_ closely resembling unflanked S_4._ All septal margins straight. Paliform lobes also straight, and appear to be arranged in a single palar crown. Fossa relatively shallow, containing a papillose columella, composed of ≤ 20 granular and closely-packed rods.

###### Distribution.

Regional: Eastern margin of South Africa, off Port St. Johns extending towards Durban; 96–695 m. Elsewhere: Indonesia (Cairns and Zibrowius 1997); Tanzania; Madagascar (Cairns and Keller 1993); 180–1016 m.

###### Remarks.

Stephanocyathus (A.) explanans differs from the only other subgeneric extant species [*S.spiniger* (von Marenzeller, 1888)], in its calicular margin, number of septa, and costal spines. Cairns and Zibrowius (1997) elaborated on these taxonomic differences, highlighting that *S.explanans* may be distinguished in having a serrated calicular margin (lanceted in *S.spiniger*), 48–72 septa (instead of 96 septa), and 6–11 marginal circular spines (consistently 6 elongated and basally compressed in *S.spiniger*). *Stephanocyathusexplanans* was first reported from South Africa by Gardiner and Waugh (1938), however no further locality information apart from noting that specimens were collected “off South Africa” was provided. Subsequently, Boshoff (1981) incorrectly identified the species as Stephanocyathus (O.) nobilis (von Marenzeller, 1904a) (ORI**_**D111k2), a record later corrected as *S.explanans* by Cairns and Keller (1993). The South African representatives add no new morphological information apart from S_1_ being slightly wider than S_2_ in some of the examined specimens.

##### Stephanocyathus (Odontocyathus)

Taxon classificationAnimaliaScleractiniaCaryophylliidae

Moseley, 1881

35444DD3-E3C1-5FC9-8C3C-86DA06079A38

###### Diagnosis.

*Stephanocyathus* with 12–18 short basal spines or tubercles (C_1–2_, sometimes C_3_), sometimes fusing into a basal rim.

###### Type species.

*Platytrochuscoronatus* Pourtalès, 1867, by monotypy.

##### Stephanocyathus (Odontocyathus) campaniformis

Taxon classificationAnimaliaScleractiniaCaryophylliidae

(von Marenzeller, 1904)

E5A48166-B655-5D69-B79D-45424EC57855

Fig. 5G–I

Stephanotrochus campaniformis von Marenzeller, 1904a: 302, pl. 18, figs 20, 20A.
Stephanocyathus campaniformis . –Gardiner and Waugh 1938: 189, 191. –Zibrowius 1980: 103. –Zibrowius and Gili 1990: 32–35, pl. 4, figs A–F, pl. 5, figs E–J
Stephanocyathus (Odontocyathus) campaniformis . –Cairns and Keller 1993: 243, fig. 5A, B.

###### Type locality.

Off Walvis Ridge, Namibia (SS ‘Valdivia’ stn. 83: 25°25'03"S, 6°12'04"E); 981 m (von Marenzeller 1904a).

###### Type material.

Two syntypes are deposited at the ZMB (Zibrowius and Gili 1990).

###### Material examined.

SAM_H3178 (1 specimen): Eastern margin, 39 km from Coffee Bay/31 km off Hluleka Estuary, 32°00'42.00"S, 29°33'00.00"E; 1420 m.

###### Description.

Corallum highly variable in shape, ranging from flat, cylindrical, bulbous, to bowl-shaped (as in the examined specimen) with rounded base. BD smaller than CD, and having a basal scar indicating point of attachment. Calice slightly elliptical (GCD:LCD = 1.1), with serrated calicular margin. Only specimen examined 29.8 × 27.5 mm in CD, 24.3 in BD, and 12.6 mm in H. Lower costae unevenly sized and spaced, with C_1–2_ prominent and extending from calicular margin to base, where they bear basal tubercles. C_3–5_ equal, separated by fine striae, and less distinct towards base. All costae covered with fine granules. Corallum white.

Septa hexamerally arranged in five cycles, the last cycle being incomplete, according to the formula: S_1–2_ > S_3_ > S_4_ > S_5_ (≤ 80 septa). S_1–2_ highly exsert, and extend to columella with straight axial margins. Each S_1–2_ bears a palus separated from its respective septum by a deep notch. P_1_ fuse to columella. S_3_ less exsert and ¾ the width of S_1–2_, with a straight axial margin, and separated from respective P_3_ by a notch. When flanked by S_4_, S_3_ and S_4_ join deeper in fossa, meeting columella as one septum. S_4_ less exsert, ½ the width of S_3_, with straight to slightly dentate axial margins. S_5_ equally exsert as surrounding S_4_, but rudimentary in development. Septal faces covered with fine granules. Pali variable in size and shape. Fossa relatively shallow, but reported to be deep in larger specimens. An under-developed papillose columella present, usually a result of pali fusing.

###### Distribution.

Regional: Eastern margin of South Africa, off Coffee Bay; 1420 m. Elsewhere: Madagascar Plateau (Cairns and Keller 1993); Walvis Ridge (off Namibia) (Zibrowius and Gili 1990); 882–1610 m.

###### Remarks.

Among the Atlantic extant species of Stephanocyathus (Odontocyathus), *S.campaniformis* closely resembles *S.nobilis* (Moseley, 1873), but differs in having a considerably smaller adult size (7.5–11.5 mm) compared with that reported for *S.nobilis* (10.0–15.0) (Cairns and Keller 1993; Zibrowius and Gili 1990), and a higher H:GCD ratio (0.4–1.3) unlike that of *S.nobilis* (0.4–0.8 mm) (Cairns and Keller 1993; Zibrowius and Gili 1990). The resemblance of the Indian and Atlantic Ocean *S.campaniformis* representatives has been historically noted (Cairns and Keller 1993), and specimens reported herein add no taxonomic knowledge to this discussion. Specimens examined herein represent a new South African record, thus extending the Indian Ocean records from the Madagascar Plateau further southwards.

##### Stephanocyathus (Odontocyathus) nobilis

Taxon classificationAnimaliaScleractiniaCaryophylliidae

(Moseley, 1873)

3BE7BF17-3CB9-5FEE-8EEB-A7A25903DD24

Fig. 5J–L

Ceratrochus nobilis Moseley in Thomson, 1873: 402, fig. 3. –Moseley in Thomson 1876: 554.
Stephanotrochus nobilis . –Moseley 1881: 155, pl. 3, fig. 3A–B. –Jourdan 1895: 20.
Stephanotrochus nitens Alcock in Wood-Mason & Alcock, 1891: 7–8.
Stephanotrochus platypus . –Jourdan 1895: 19, pl. 2, figs 14–16.
Stephanotrochus diadema var. nobilis . –Gravier 1920: 47–51, pl. 5, figs 80–86, pl. 6, figs 87–89, pl. 14, figs 205, 206.
Sabinotrochus opulens Gravier, 1920: 54, pl. 6, figs 101–103.
Stephanocyathus nobilis . –Gardiner and Waugh 1938: 189–192, pl. 6, figs 13, 15. –Pillai and Scheer 1976: 16. –Zibrowius 1980: 101–108, pl. 51, figs A–K.
Stephanocyathus (Odontocyathus) nobilis . –Cairns 1979: 110–111, pl. 20, figs 7, 10. –Cairns and Keller 1993: 242, fig. 5D, E.

###### Type locality.

South of Flores, Azores (HMS ‘Challenger’ stn. 38°30'00"N, 31°14'00"W); 1830 m (Moseley 1876).

###### Type material.

The holotype is deposited at the NHMUK (Zibrowius 1980).

###### Material examined.

SAM_H1697 (1 specimen): Eastern margin, 58 km south of Ponta Do Ouro/46 km off Mgobezeleni Estuary, 27°21'18.00"S, 33°03'53.99"E; 1050 m.

###### Description.

Corallum bowl-shaped with rounded base. BD smaller than CD. Base eroded but usually having a basal scar indicating point of previous attachment. Prominent basal tubercles corresponding to C_1–2_. Calice slightly elliptical (GCD:LCD = 1.03), with serrated calicular margin. Only specimen examined 41.3 × 40.0 mm in CD, 29.3 in BD, and 24.9 mm in H. Lower costae unevenly sized and spaced. C_1–2_ prominent and extending from calicular margin to base. Theca mostly smooth and glossy, with traces of costae, which change to 2–3 tubercles at basal inflection point, and progressively becoming less prominent at basal centre. C_3–5_ subequal, with thin and deep striae at calicular margin, becoming faint and slowly disappearing towards base. All costae covered by fine granules. Corallum white.

Septa hexamerally arranged in five cycles, the last cycle being incomplete, according to the formula: S_1_ ≥ S_2_ > S_3_ > S_4_ > S_5_ (≤ 76 septa). S_1–2_ highly exsert and extend to columella with straight axial margins. P_1–2_ separated from their respective septum by a shallow notch. P_1_ reaches/fuses to columella. S_3_ less exsert and ^3^/_4_ the width of S_1–2_. S_3_ axial margins straight to slightly sinuous and bear a palus (P_3_). P_3_ sometimes joined by adjacent S_4_ deeper in fossa. S_4_ less exsert and ½ the width of S_3_, with straight axial margins. S_5_ rudimentary but as exsert as S_4._ All septal faces covered with fine granules. Pali variable in size, shape, and height. P_1–3_ form an inner crown encircling the well-developed papillose columella composed of closely packed rods. Fossa relatively deep, but reported to be shallow in smaller specimens.

###### Distribution.

Regional: Eastern margin of South Africa, off Mgobezeleni Estuary (58 km south of Ponta Do Ouro: Mozambique); 1050 m. Elsewhere: Mozambique (Cairns and Keller 1993); off Zanzibar; off Kenya (Gardiner and Waugh 1938); Madagascar (Gardiner and Waugh 1938); Arabian Sea (Wood-Mason and Alcock 1891a); Maldives (Gardiner and Waugh 1938); off England; Azores; Gulf of Guinea (Zibrowius 1980); 609–2200 m.

###### Remarks.

As mentioned in the remarks section of Stephanocyathus (O.) campaniformis, *S.nobilis* may be confused with *S.campaniformis* but differs in corallum size and height in relation to GCD. Both species have overlapping distributional patterns and are reported to occur in the Indian and Atlantic Ocean basins, this observation leading up to historical discussions around the validity of the Indian Ocean representatives as *S.nobilis*. However, Cairns and Keller (1993) agreed with Zou (1988) and confirmed that the Indian and Atlantic Ocean representatives are indeed one species of *S.nobilis*. The current Indian Ocean record represents a southern range extension from Mozambique and is a new record for South Africa.

##### Tethocyathus

Taxon classificationAnimaliaScleractiniaCaryophylliidae

Kühn, 1933

7824EC68-9265-5E29-8CFB-B576C9DF93D8

###### Diagnosis.

Corallum solitary, turbinate to trochoid, fixed or free. Septotheca covered by thick epitheca. Paliform lobes before all but last septal cycle in two distinct crowns. Columella papillose at top.

###### Type species.

*Thecocyathusmicrophyllus* Reuss, 1871, by original designation.

##### Tethocyathus virgatus

Taxon classificationAnimaliaScleractiniaCaryophylliidae

(Alcock, 1902)

65CFDE4A-AA35-5BDD-A916-1222F8B95CFF

Fig. 5M, N

Trochocyathus (Thecocyathus) virgatus Alcock, 1902a: 98–99. –Alcock 1902c: 16–17, pl. 2, fig. 13.
Tethocyathus virgatus . –Cairns 1995: 65–66, pl. 16C–F. –Cairns and Zibrowius 1997: 114–115. –Cairns 1999a: 86. –Cairns 2004a: 286. –Cairns 2009: 9. –Kitahara et al. 2010a: 115. –Kitahara and Cairns 2021: 570–571, 573, figs 316, 317A–C.

###### Type locality.

Off Sulu Archipelago (HMS ‘Siboga’ stns. 96 and 105: South–east of Pearl Bank and 6°08'00"S, 121°19'00"E, respectively); 15–275 m (Alcock 1902c).

###### Type material.

Two syntypes are deposited at the ZMA (Cairns 2004a).

###### Material examined.

SAMC_A073083 (1 specimen): Eastern margin, 28 km from Richards Bay/40 km off Mlalazi Estuary, 29°00'54.00"S, 32°12'06.12"E; 215 m. SAMC_A073180 (2 specimens): Southern margin, 33 km from Mazeppa Bay/24 km off Cwili Estuary, 32°45'47.88"S, 28°36'24.12"E; 240–250 m. USNM 91674 (1 specimen): Eastern margin, 32 km from Port Dunford/37 km off Mlalazi Estuary, 29°05'10.79"S, 32 08'10.79"E; 95 m.

###### Description.

Corallum solitary, ceratoid to subcylindrical, and attached to substrate by a thick pedicel (PD:GCD = 0.2–0.7) that expands into a polycyclic encrusting base. Calice elliptical (GCD:LCD = 1.1–1.2), with a jagged calicular margin. Largest specimen examined (USNM 91674) 13.4 × 11.8 mm in CD, and 26.3 mm in H. Costae unequal, flat, granular, separated by shallow intercostal striae, and becoming less prominent towards base. Epitheca may be thick and penetrated by lenticular pores of acrothoracid barnacles, or thin with no incrustation. Corallum pigmented purple-black, that fades into a white colouration towards base.

Septa hexamerally arranged in five cycles, the last cycle being incomplete, according to the formula: S_1_ > S_2_ > S_3_ ≥ S_4_ > S_5_ (< 68 septa). Septa closely packed. S_1–2_ exsert, independent, thickest, and most swollen. Higher cycle septa (S_3–4_) progressively less exsert and less thick. S_3_^1^/_2_ the width of S_1–2._ S_4_ dimorphic in size: those adjacent to S_1_ equal to or slightly wider than S_3_; and those adjacent to S_2_ usually less wide than S_3._ S_5_ same width as S_4_ adjacent to S_1_, if present. All septa have straight axial margins except for S_3,_ which have slightly sinuous ones. Septal faces granular. Pali large, closely packed, and present before all septal cycles but last, forming a high paliform crown encircling a papillose columella composed of a group of closely-packed and low-profile rods.

###### Distribution.

Regional: Southern to eastern margin of South Africa, from Kei Mouth extending towards Port Dunford; 95–250 m. Elsewhere: Philippines; Indonesia (Cairns and Zibrowius 1997); Vanuatu; Wallis and Futuna Islands (Cairns 1999a); New Caledonia (Kitahara and Cairns 2021); New Zealand (Cairns 1995); Australia (Cairns 2004a); 95–1200 m.

###### Remarks.

*Tethocyathusvirgatus* is distinguished from the other Recent congeners in having a pigmented CS_1_, S_1–2_ being the thickest septa, well-developed lamellar pali, and by attaining the largest corallum size. The examined specimens represent a new record for the region. The South African representative differs from the Australian in having septa of the fifth cycle (< 68 septa) as compared with only four (48 septa), and in having S_3_ that bear a slightly sinuous rather than a straight axial margin. Nonetheless, all the other taxonomic features agree with what is known to be characteristic of the species.

##### Trochocyathus

Taxon classificationAnimaliaScleractiniaCaryophylliidae

Milne-Edwards & Haime, 1848

69116D65-AD1A-50B5-B1B6-BF38A0DE47CF

###### Diagnosis.

Corallum solitary, turbinate to ceratoid, or bowl-shaped, fixed or free. Transverse division may be present. Septotheca costate, sometimes covered with a thin epitheca. Pali before all but last cycle of septa. Columella papillose.

###### Type species.

*Turbinoliamitrata* Goldfuss, 1827, by subsequent designation (Milne-Edwards and Haime 1850b).

##### Trochocyathus (Trochocyathus)

Taxon classificationAnimaliaScleractiniaCaryophylliidae

Milne-Edwards & Haime, 1848

39A865D7-5758-5623-9490-A28E4953C10B

###### Diagnosis.

*Trochocyathus* lacking basal costal spines and with other than discoidal coralla.

###### Type species.

*Turbinoliamitrata* Goldfuss, 1827, by subsequent designation (Milne-Edwards and Haime 1850b).

##### Trochocyathus (Trochocyathus)

Taxon classificationAnimaliaScleractiniaCaryophylliidae

sp. 1

D1B7D02E-CA2B-5A14-922B-594FF7F659D6

Figs 5O, P , 6A, B

Trochocyathus rawsonii . –Gardiner 1904: 100–103, 124, pl. 1, fig. 2A, B, pl. 2, fig. D, E, G, H, J, K . –Cairns and Keller 1993: 241.
Caryophyllia gigas . –Boshoff 1981: 36.
Endopachys grayi . –Boshoff 1981: 42 (in part).

###### Material examined.

SAMC_A073233 (2 specimens): Southern margin, 14 km from Cape Point/10 km off Buffels Wes Estuary, 34°13'59.99"S, 18°30'00.00"E; 42 m. SAM_H1244 (1 specimen): Southern margin, 7 km from East London/5 km off Buffalo Estuary, 33°02'59.99"S, 27°56'59.99"E; 59 m. SAM_H1449 (1 specimen): Southern margin, 14 km from Mazeppa Bay/20 km off Great KeiEstuary, 32°34'00.00"S, 28°33'00.00"E; 174 m. SAM_H3115 (1 specimen): Southern margin, 2 km off Mosselbaai/10 km off Hartenbos Estuary, 34°10'37.57"S, 22°09'19.14"E; 55 m. SAM_H3117 (1 specimen): Southern margin, 246 km from Mazeppa Bay/243 km off Mendu Estuary, 33°43'11.99"S, 30°48'47.99"E; 780 m. SAM_H3177 (2 specimens): Southern margin, 15 km from Port Alfred/11 km off Riet Estuary, 33°39'18.00"S, 27°11'35.99"E; 90 m. SAM_H3833 (1 specimen): Eastern margin, 34 km from Coffee Bay/7 km off Ntlonyane Estuary, 32°15'11.99"S, 28°57'42.00"E; 47 m. SAM_H3834 (1 specimen): Southern margin, 23 km from Port Elizabeth/22 km off Bakens River Estuary, 33°50'41.99"S, 25°47'30.00"E; 36 m. ORI_DIIIa4_3 (1 specimen): Locality data unknown, 300 m. ORI_EId1 (2 specimens): Eastern margin, other locality data unknown. USNM 77220 (3 specimens): Eastern margin, 28 km from Coffee Bay/19 km off Bulungulu Estuary, 32°14'53.99"S, 29°10'23.99"E; 620–560 m.

###### Imagery data.

BMNH 1950.03.22.17 (1 specimen), **BMNH 1950.01.10.112 (1 specimen)**, NHMUK 1970.01.26.11–20 (2 specimens): South Africa, other locality data unknown. Mortensen Java Expedition (12 specimens): Eastern margin, off Durban, 128 m.

###### Description.

Corallum solitary, ceratoid to trochoid, mostly attached through a variably sized pedicel (PD:GCD = 0.3–0.7). Calice circular to slightly elliptical (GCD:LCD = 1.0–1.1), calicular margin slightly serrated. Largest specimen examined (ORI_DIIIa4_3) 11.1 × 10.1 mm in CD, 12.0 mm in H, and 6.6 mm in PD. Costae prominent from calicular margin to base, similar in width to associated septa, equidistant, low, bearing small granules, and separated by thin intercostal furrows. Corallum white.

Septa hexamerally arranged in four cycles according to the formula: S_1_ ≥ S_2_ > S_3_ > S_4_ (48 septa); sometimes a pair of S_5_ present in a half-system (e.g., ORI_EId1). S_1_ most exsert, equal or slightly wider than S_2_, with straight or slightly sinuous axial margin. S_2_ ¼ wider than S_3_ with moderately sinuous axial margin. S_3_ as sinuous as S_2_. S_4_ rudimentary and bearing dentate axial margin. S_3–4_ progressively less exsert than S_2_. Pali present before all but last septal cycle. P_2–3_ joining deeper in fossa, forming thick chevrons before S_2._ Pali tall, being distinctively higher than columellar elements. All septal and palar faces covered with small granules. Fossa moderately deep, containing a papillose columella composed of a group of 6–18 loosely-packed, low-profile rods.

###### Distribution.

Regional: Southern to eastern margin of South Africa, from off Cape Point towards Coffee Bay; 36–780 m.

###### Remarks.

The validity of Gardiner’s (1904) specimens as Trochocyathus (T.) rawsonii has long been questioned due to their well-developed costae, slender pedicel, and rarely bearing S_5_ (Cairns 1979; Zibrowius and Gili 1990; Cairns and Keller 1993). The examined material conforms to these characteristics and closely resembles Gardiner’s (1904) illustrations. Nevertheless, the specimens reported herein, together with Gardiner’s (1904), might represent a new species. However, as *Trochocyathus* have great intraspecific variation, a formal description is postponed. Nonetheless, these new records extend the known distribution of this taxon towards the eastern margin of South Africa and also expand its known depth range (both upper and lower).

##### Trochocyathus (Trochocyathus)

Taxon classificationAnimaliaScleractiniaCaryophylliidae

sp. 2

43D164ED-1C76-536F-A20D-89298F0880A3

Fig. 6C–F

###### Material examined.

DEFF/SAEON_A33997 (1 specimen): Southern margin, 39 km from Cape Padrone/40 km off Boknes Estuary, 34°03'53.52"S, 26°42'11.58"E; 100 m. DSCS–INV 529 (1 specimen): Southern margin, 35 km from Cape St. Francis/70 km off Slang Estuary, 34°47'05.0"S, 24°45'42.3"E; 392–418 m SAMC_A073141 (3 specimens): Eastern margin, 33 km from Coffee Bay/18 m off Ntlonyane Estuary, 32°16'41.88"S, 29°06'00.00"E; 300 m. SAMC_A073150 (3 specimens): Southern margin, 26 km from Mazeppa Bay/33 km off Great Kei Estuary, 32°41'12.12"S, 28°43'54.12"E; 480–490 m. SAMC_A073166 (1 specimen): Eastern margin, 19 km off Port St. Johns/21 km off Bulolo Estuary, 31°45'00.00"S, 29°40'47.99"E; 125 m. SAMC_A073175 (1 specimen): Eastern margin, 28 km from Coffee Bay/16 km off Hluleka Estuary, 31°55'58.79"S, 29°25'12.00"E; 300 m. SAMC_A073211 (3 specimens): Eastern margin, 5 km from Cape Vidal/16 km off St Lucia Estuary, 28°08'24.00"S, 32°36'24.00"E; 165 m. SAMC_A073235 (2 specimens): Southern margin, False Bay; depth unknown. SAMC_A088920 (1 specimen): Southern margin, 38 km from Cintsa/22 km off Cwili Estuary, 32°50'30.72"S, 28°30'41.33"E; 250–226 m. SAMC_A088921 (3 specimens): Southern margin, 140 km from Agulhas/144 km off Ratels Estuary, 36°02'29.58"S, 19°41'24.61"E; 445–463 m. SAMC_A088925 (1 specimen): Southern margin, 140 km from Agulhas/144 km off Ratels Estuary, 36°02'29.58"S, 19°41'24.61"E; 445–463 m. SAMC_A088926 (8 specimens): Southern margin, 140 km from Agulhas/144 km off Ratels Estuary, 36°02'29.58"S, 19°41'24.61"E; 445–463 m. SAMC_A090146 (5 specimens): Southern margin, 68 km from Cape St. Francis/70 km off Slang Estuary, 34°47'35.77"S, 24°38'35.69"E; 520 m. SAMC_A090147 (2 specimens): Southern margin, 140 km from Agulhas/144 km off Ratels Estuary, 36°02'29.58"S, 19°41'24.61"E; 445–463 m. SAM_H1243 (3 specimens): Southern margin, 11 km from Infanta/12 km off Duiwenhoks Estuary, 34°27'00.00"S, 20°58'00.00"E; 47 m. SAM_H1370 (1 specimen): Southern margin, 28 km from Gonubie/27 km off Buffalo Estuary, 33°09'29.99"S, 28°03'06.00"E; 86 m. SAM_H1384 (1 specimen): Southern margin, 2 km from Mosselbaai/11 km off Hartenbos Estuary, 34°11'10.12"S, 22°09'40.59"E; 95 m. SAM_H1388 (14 specimens): Southern margin, 11 km from East London/5 km off Gouda Estuary, 33°05'03.24"S, 27°49'33.40"E; 146–238 m. SAM_H1391 (1 specimen): Southern margin, 24 km from Cape Padrone/35 km off Boknes Estuary, 33°58'00.00"S, 26°21'00.00"E; 46 m. SAM_H1400 (1 specimen): Southern margin, 74 km off Cape St. Francis/83 km off Slang Estuary, 34°52'00.00"S, 24°56'59.99"E; 137 m. SAM_H1407 (1 specimen): Southern margin, 2 km from Mosselbaai/11 km off Hartenbos Estuary, 34°11'10.12"S, 22°09'40.59"E; 192 m. SAM_H1408 (1 specimen): Southern margin, 14 km from Mazeppa Bay/20 km off Great Kei Estuary, 32°34'00.00"S, 28°33'00.00"E; 174 m. SAM_H1419 (1 specimen): Southern margin, 4 km from Plettenberg Bay/7 km off Piesang Estuary, 34°06'00.00"S, 23°23'59.99"E; 146 m. SAM_H1443 (26 specimens): Southern margin, 241 km from Agulhas/247 km off De Mond-Heuningnes Estuary, 36°40'00.00"S, 21°25'59.99"E; 200 m. SAM_H1487 (13 specimens): Southern margin, 2 km from Mosselbaai/11 km off Hartenbos Estuary, 34°11'10.12"S, 22°09'40.59"E; 229 m. SAM_H3103 (2 specimens): Southern margin, 2 km from Mosselbaai/11 km off Hartenbos Estuary, 34°11'10.12"S, 22°09'40.59"E; 165–183 m. SAM_H3106 (9 specimens): Southern margin, 846 km from Port St. Johns/842 km off Mkweni Estuary, 36°40'00.00"S, 21°25'59.99"E; 366 m. SAM_H3111 (1 specimen): Eastern margin, 18 km from Cape Vidal/27 km off Mfolozi Estuary, 28°16'18.00"S, 32°38'48.00"E; 670 m. SAM_H3114 (2 specimens): Southern margin, 11 km from East London/5 km off Gouda Estuary, 33°05'03.24"S, 27°49'33.40"E; 146–238 m. SAM_H3116 (9 specimens): Eastern margin, 22 km from Cape Vidal/28 km off Mfolozi Estuary, 28°17'23.99"S, 32°40'47.99"E; 550 m. SAM_H3170 (15 specimens): Eastern margin, 28 km from Coffee Bay/19 km off Bulungulu Estuary, 32°14'53.99"S, 29°10'23.99"E; 620–560 m. SAM_H3172 (3 specimens): Southern margin, 29 km from Mazeppa Bay/25 km off Kobole Estuary, 32°29'30.00"S, 28°57'06.00"E; 650–700 m. SAM_H3173 (1 specimen): Southern margin, 47 km from Port Alfred/14 km off Mgwalana Estuary, 33°30'18.00"S, 27°22'05.99"E; 80 m. SAM_H3175 (1 specimen): Eastern margin, 30 km from Coffee Bay/20 km off Bulungulu Estuary, 32°15'00.00"S, 29°09'06.00"E; 520–500 m. SAM_H3176 (3 specimens): Eastern margin, 30 km from Scottburgh/20 km off Fafa Estuary, 30°33'24.00"S, 30°48'35.99"E; 690 m. SAM_H3830 (2 specimens): Western margin, 49 km from Cape Point/51 km off Buffels Wes Estuary, 34°43'18.00"S, 18°12'29.99"E; 360–365 m. SAM_H3832 (3 specimens): Southern margin, 14 km from Cape Point/10 km off Buffels Wes Estuary, 34°13'59.99"S, 18°30'00.00"E; 42 m.

###### Description.

Corallum ceratoid to trochoid, and attached to a variably sized pedicel (PD:GCD = 0.2–0.7). Calice circular to slightly elliptical (GCD:LCD = 1.0–1.2), with serrate calicular margin. Largest specimen examined (SAM_H1400) 17.4 × 17.2 mm in CD, 6.0 mm in PD, and 39.6 mm in H. Costae prominent from calicular margin to base, ca. the same width as associated septa, equidistant, low, bearing small granules, and separated by thin intercostal furrows. Corallum white.

Septa hexamerally arranged in five cycles, the last cycle being incomplete, according to formula S_1_ > S_2_ ≥ S_3_ > S_4_ > S_5_ > S_6_ (≤ 60 septa). S_1_ most exsert, independent, slightly wider than S_2_, and bearing straight to slightly sinuous axial margin. S_2_ slightly less exsert than S_1_, and extending slightly less to centre of calice with moderately sinuous axial margin. S_3_ equal or slightly smaller than S_2,_ but have axial margin equally sinuous. S_4_ predominantly restricted to upper calice in specimens with four septal cycles, but dimorphic and variable in development half-systems bearing S_5_ or S_5–6_ (e.g., SAMC_A090146 = 6:6:12:24:10:2, SAM_H1407 = 6:6:12:24:12). In the latter, S_4_ closer to S_1_ have the same rudimentary profile as S_5_ and S_4_ flanked by S_5_ having a similar profile as S_3_ or slightly less wide. S_4_ axial margin coarsely dentated. If present, S_6_ restricted to calicular margin. S_3–5_ progressively less exsert than S_1–2_. Pali present before all but last septal cycle. P_2_ most recessed. P_2–3_ form a chevron-like pattern before S_2,_ a pattern also taken by S_4_ flanked by S_5_ which sometimes bear pali. All septal and palar faces granulated. Fossa moderately deep, containing a papillose columella composed of a group of 17–30 rod elements which mostly are as high as pali elements.

###### Distribution.

Regional: Western to eastern margin of South Africa, off Cape Point extending towards Cape Vidal; 42–700 m.

###### Remarks.

Although specimens examined closely resemble Trochocyathus (T.) sp. 1 cf. *T.rawsonii* sensu Gardiner (1904) in having well-developed costae extending from the calicular margin to base, an often curved coralla, and the presence of S_5,_*Trochocyathus* sp. 2 occasionally has a pair of S_6_, even though the fifth cycle is incomplete (SAMC_A090146 = 6:6:12:24:10:2), and an independent S_1_ which extends further to the columella. Apart from that, the specimens reported herein differ from Trochocyathus (T.) sp. 1 cf. *T.rawsonii* sensu Gardiner (1904) in having a systematic development of septal cycles (irrespective of an increase mm in CD). While this material may represent a new species of *Trochocyathus*, a name will not be introduced until the intraspecific variation is better understood and compared with that from congeners.

##### Trochocyathus Trochocyathus sp. cf. rawsonii

Taxon classificationAnimaliaScleractiniaCaryophylliidae

sensu Cairns in Cairns & Keller, 1993

AA3A993B-3718-530C-A8CD-29241F1EEB42

Fig. 6G–L

Trochocyathus rawsonii sensu Cairns 1979: 77, fig. 6, pl. 13. –Cairns and Keller 1993: 241–242, figs 4E, H.

###### Type locality.

Off Barbados, eastern Caribbean Island (USCSS ‘Hassler’); 183 m (Cairns 1979).

###### Type material.

Three syntypes are deposited at the MCZ (Cairns 1979).

###### Material examined.

DEFF/SAEON_A32823 (1 specimen): Southern margin, 131 km from Gouritsmond/off Goukamma Estuary, 35°14'57.1"S, 22°50'48.1"E; 511 m. SAMC_A073015 (1 specimen): Southern margin, 32 km from Mazeppa Bay/19 km off Mendu Estuary, 32°25'00.11"S, 28°58'18.11"E; 330–340 m. SAMC_A073206 (2 specimens): Eastern margin, 36 km from Richards Bay/49 km off Nhlabane Estuary, 29°02'12.11"S, 32°19'36.12"E; 760–800 m. SAMC_A073220 (1 specimen): Southern margin, 13 km from Port St. Johns/12 km off Bulolo Estuary, 31°44'48.12"S, 29°33'00.00"E; 370 m. SAMC_A090091 (1 specimen): Eastern margin, 152 km from Margate/off Bilanhlolo Estuary, 31°39'07.00"S, 29°39'42.00"E; 300–540 m. SAMC_A090156 (3 specimens): Southern margin, 14 km from Sedgefield/off Cunge Estuary, 34°09'17.46"S, 22°48'37.32"E; 74 m. SAM_H1360 (1 specimen): Southern margin, 11 km from East London/3 km off Buffalo Estuary, 33°00'53.67"S, 27°55'50.67"E; 128 m. SAM_H1415 (1 specimen): Southern margin, 8 km from Pringle Bay/6 km off Buffels Oos Estuary, 34°17'55.37"S, 18°49'10.85"E; 33 m. SAM_H1440 (3 specimens): Southern margin, 11 km from East London/5 km off Gouda Estuary, 33°05'03.24"S, 27°49'33.40"E; 146–238 m. SAM_H3110 (26 specimens): Eastern margin, 18 km from Cape Vidal/27 km off Mfolozi Estuary, 28°16'18.00"S, 32°38'48.00"E; 670 m. SAM_H3113 (2 specimens): Southern margin, 11 km from East London/3 km off Buffalo Estuary, 33°00'53.67"S, 27°55'50.67"E; 128 m. SAM_H3171 (8 specimens): Southern margin, 40 km from Cintsa Mouth/29 km off Cwili Estuary, 32°55'00.00"S, 28°31'00.00"E; 630 m. SAM_H3174 (1 specimen): Southern margin, 22 km from Gonubie/21 km off Gqunube Estuary, 33°06'00.00"S, 28°08'17.99"E; 700–650 m. SAM_H3829 (1 specimen): Southern margin, 98 km from Gansbaai/103 km off Buffels Oos Estuary, 35°15'18.00"S, 18°39'18.00"E; 547 m.

###### Description.

Corallum ceratoid to trochoid, attached to a narrow pedicel (PD:GCD = 0.3–0.5). Calice circular to slightly elliptical (GCD:LCD = 1.0–1.3), calicular margin slightly serrate. Largest specimen examined (SAM_H3113) 12.2 × 11.2 mm in CD, 4.9 mm in PD, and 17.5 mm in H. Costae prominent and bearing small granules near calicular margin, progressively becoming narrower towards base. Costae separated by deep furrows. Corallum white, but sometimes purplish with white columella.

Septa hexamerally arranged in four cycles according to the formula: S_1_ ≥ S_2_ > S_3_ > S_4_ (48 septa). S_1–2_ usually equally exsert, but sometimes S_2_ may be slightly less exsert than S_1_. S_1_ meets columella with straight to slightly sinuous axial margins. S_1_ slightly wider to equal in width to S_2._ S_2_ axial margins straight to slightly sinuous. Higher cycle septa (S_3–4_) progressively less exsert. S_3_ ~ ¼ less wide than S_2,_ with moderately sinuous axial margins. S_4_ ½ the width of S_3,_ and bear extremely sinuous and sometimes dentate axial margins. Pali present before all but last septal cycle, being taller than columellar elements, slightly sinuous, and thick, with some half-systems having P_2–3_ joining in front of S_2_ before extending towards columella. All septal and palar faces being granulated. Fossa moderately deep, containing a papillose columella composed of a group of low-profile and compact intertwined rods.

###### Distribution.

Regional: Southern to eastern margin of South Africa, from off Pringle Bay extending towards Cape Vidal; 33–800 m. Elsewhere: north-western Madagascar and off Walter Shoal (Cairns and Keller 1993); 760–780 m.

###### Remarks.

Although specimens resemble Gardiner’s (1904)Trochocyathus (T.) sp. in having four cycles with the following septal formula S_1_ ≥ S_2_ > S_3_ > S_4,_ they differ in S_4_ consistently bearing sinuous to dentate axial margins unlike the exclusively dentate appearance observed in Gardiner’s (1904) representatives. The level of granulation on septal and palar faces may also distinguish the two forms, of which Trochocyathus (T.) sp. cf. *T.rawsonii* sensu Cairns and Keller (1993) has low coverage appearance (with large granules prominent along the septal faces) as compared with the fine and dense granules throughout both the pali and septa (giving specimens a rough appearance) in Gardiner’s (1904)*Trochocyathus* forms. Furthermore, the pali are compact in Trochocyathus (T.) sp. cf. *T.rawsonii* sensu Cairns and Keller (1993) rather than being loosely packed (Trochocyathus (T.) sp. 1 cf. *T.rawsonii* sensu Gardiner (1904)). However, some specimens of Trochocyathus (T.) sp. cf. *T.rawsonii* sensu Cairns and Keller (1993) diverge from the typical compact pali, thus bearing a tall and thin pali (SAM_H1415 and SAMC_A090156).

##### Trochocyathus (Trochocyathus)

Taxon classificationAnimaliaScleractiniaCaryophylliidae

sp. 3

99F0A8B7-E331-54CD-B506-C62A29997437

Fig. 6M–P

Caryophyllia berteriana . –Gardiner 1904: 112–113.
Trochocyathus (Trochocyathus) sp. –Zibrowius 1982: 114, pl. 1, figs 1, 2.
Trochocyathus (Trochocyathus) sp. A. –Cairns and Keller 1993: 240, fig. 4C, D.

###### Material examined.

SAMC_A073157 (1 specimen): Eastern margin, 10 km from Port Edward/24 km off Bilanhlolo Estuary, 31°05'48.11"S, 30°18'47.88"E; 140 m. SAMC_A073213 (1 specimen): Eastern margin, 29 km from Durban/14 km off Mbokodweni Estuary, 30°06'24.12"S, 31°00'47.88"E; 160–170 m. SAM_H3124 (1 specimen): Southern margin, 11 km from East London/5 km off Gouda Estuary, 33°05'03.24"S, 27°49'33.40"E; 146–238 m. USNM 91530 (3 specimens): Eastern margin, 37 km south of Ponta Do Ouro/23 km off Kosi Bay Estuary, 27°11'03.59"S, 32°50'32.39"E; 100 m. **USNM 91566 (1 specimen)**: Eastern margin, 42 km south of Ponta Do Ouro/27 km off Kosi Bay Estuary, 27°13'30.00"S, 32°49'30.00"E; 74 m.

###### Imagery data.

**BMNH 1939.7.20.47 (1 specimen**): Eastern margin, 20 km from Richards Bay/8 km off Nhlabane Estuary, 28°40'00.00"S, 32°15'00.00"E; 165 m.

###### Description.

Corallum ceratoid, mostly attached to substrate through a narrow pedicel (PD:GCD = 0.4–0.6) and a thin encrusting base. Calice circular to slightly elliptical (GCD:LCD = 1.0–1.1), calicular margin slightly serrate. Largest specimen examined (USNM 91566) 8.2 × 7.3 mm in CD, and 9.0 mm in H. Costae prominent and bearing small granules near calicular margin, and progressively disappear towards pedicel. Theca becomes transversely ridged beyond 3 mm from calicular margin. Corallum white, and sometimes light brown around calicular margin.

Septa hexamerally arranged in four cycles according to the formula: S_1_ ≥ S_2_ > S_3_ ≥ S_4_ (48 septa). However, largest specimen (USNM 91566) displays S_1–2_ > S_4–3._ S_1_ most exsert and extend slightly further into centre of calice than S_2_. S_1_ axial margins straight to slightly sinuous. S_2_ equally wide or ¼ less wide than S_1,_ with moderately sinuous axial margins. Higher cycle septa (S_3–4_) progressively less exsert (if at all). S_3_ with most sinuous axial margins. S_4_ rudimentary and bear straight to slightly sinuous axial margins. Pali present before all but last septal cycles. P_2–3_ joining deeper in fossa, forming a chevron-like structure before S_2._ Septal and palar faces finely granulated. Fossa moderately deep, pali indistinguishable from the closely-packed rods of the papillose columella.

###### Distribution.

Regional: Southern to eastern margin of South Africa, from East London extending towards Kosi Bay Estuary (37 km south of Ponta Do Ouro: Mozambique); 74–170 m. Elsewhere: Mozambique, Madagascar, and Saya de Malha Bank; 74–315 m (Cairns and Keller 1993).

###### Remarks.

The examined specimens closely resemble Trochocyathus (T.) sp. A reported by Cairns and Keller (1993) in all features, except for its septa all having sinuous axial margins instead of only S_3_ having sinuous axial margins. Zibrowius (1982) also noted a *Trochocyathus* specimen (BMNH 1939.7.20.47) that resembles Cairns and Keller’s (1993) specimens in all septa being straight except S_3_ and in septa being arranged according to S_1–2_ > S_4–3._ The representative images of BMNH 1939.7.20.47 unfortunately do not show the tertiary septa’s axial margin, and for that reason this *Trochocyathus* specimen is added to the records of species accounted for here. Nevertheless, a new name will not be introduced until the variation within Trochocyathus (T.) rawsonii is better understood.

##### Vaughanella

Taxon classificationAnimaliaScleractiniaCaryophylliidae

Gravier, 1915

97552464-0204-50ED-954D-3021ED86ADA3

###### Diagnosis.

Corallum solitary, patellate to trochoid, and usually firmly attached by a robust pedicel. Septotheca costate. Paliform lobes present on all but last septal cycle. Columella papillose.

###### Type species.

*Caryophylliamargaritata* Jourdan, 1895, by monotypy.

##### Vaughanella concinna

Taxon classificationAnimaliaScleractiniaCaryophylliidae

Gravier, 1915

2CB9B70F-54F1-5F0E-9C21-B328DA6A7A5D

Fig. 7A–D

Vaughanella concinna Gravier, 1915: 10. –Gravier 1920: 63, pl. 9, fig. 138–143. –Zibrowius 1980: 104–105, pl. 52, figs A–K, pl. 53, figs A–L. –Cairns 1999a: 90–91, fig. 11G, H. –Cairns et al. 1999: 25. –Cairns 2009: 12. –Kitahara and Cairns 2021: 439–440, figs 237D–F, 238.
Vaughanella oreophila . –Cairns 1995: 70, pl. 18, figs D, E.

###### Type locality.

South of São Jorge, Azores (‘Prince de Monaco Expedition’ stn. 1349: 38°35'30"N, 28°05'45"W); 1250 m (Gravier 1920),

###### Type material.

The lectotype and 6 paralectotypes are deposited at the MOM (Zibrowius 1980).

###### Material examined.

SAMC_A072973 (1 specimen): Locality data unknown; 517 m.

###### Imagery data.

MN_SM134 (1 specimen): Eastern margin, 36 km off Port Shepstone/49 km off Mtentu Estuary, 30°43'11.99"S, 30°48'47.99"E; 900 m.

###### Description.

Corallum trochoid, robust, and firmly attached to substrate through a thick and reinforced pedicel (PD:GCD = 0.40–0.5) and encrusting base. Calice circular to slightly elliptical (GCD:LCD = 1.0–1.1), sometimes slightly flared, with serrated calicular margin. Largest imaged specimen (MN_SM134) 20.0 × 18.0 mm in CD, 8.0 mm in PD, and 19.0 mm in H. Costae well-developed, but unequal in width. C_1–2_ wider than remaining costae. C_3–5_ progressively narrower. Intercostal striae, broad. All costae finely granular. Corallum white.

Septa hexamerally arranged in five cycles, the last cycle being incomplete, according to the formula: S_1_ ≥ S_2_ > S_3_ > S_4_ > S_5_ (only one pair of S_5_ present in the specimens examined = 50 septa). S_1–2_ highly exsert, extending towards columella with straight axial margins. Each S_1–2_ bears a pointed and tall palus. P_1_ fuses to columellar elements. S_3_ less exsert and ¾ the width of S_2_, bearing tall and well-defined pali, which are separated from septa by a wide notch. S_4_ less exsert and dimorphic in size: generally, ¼ the width of S_3_ (S_4_ rudimentary in SAMC_A072973) but same size as S_3_ when flanked by S_5_. S_5_ restricted to calicular margin. All septa have straight axial margins, except for S_3_ which have straight to slightly sinuous axial margins. Septal faces slightly granular. Pali smooth, indistinguishable from columellar elements, except for P_3_ which are thicker than P_1–2_ and rise higher in fossa. Fossa moderately deep, containing a papillose columella composed of a compact group of rods.

###### Distribution.

Regional: Eastern margin of South Africa, off Port Edward; 517–900 m. Elsewhere: Wallis and Futuna (Cairns 1999a); New Caledonia (Kitahara and Cairns 2021); New Zealand (Cairns 1995); Celtic Sea, Azores, and Madeira Archipelago (Zibrowius 1980); 500–3018 m.

###### Remarks.

Both specimens reported (one of which is represented by imagery data) meet the known taxonomic distinction of *Vaughanella*, particularly *V.concinna* in having all higher cycle septa (S_1–3_) bearing pali (P_1–3_). However, they differ in costae extending from calicular margin towards base, as compared with theca being smooth and porcelaneous (Cairns 1995). The presence of P_3_ and one palus per septa distinguishes this species from the other two Pacific congeners, one of which lacks P_3_ (*V.oreophila* Keller, 1981) and the other has multiple lobes per septa (*V.multipalifera* Cairns, 1995). The South African representative (SAMC_A072973) displays convergence of S_4_ towards S_3_, a feature reported in the Atlantic forms, but differs in pali being smooth as compared with being granulated (Zibrowius 1980). Nonetheless, regional distribution is based on MN_SM134 (imagery data) as SAMC_A072973 lacks locality information.

**Figure 7.**
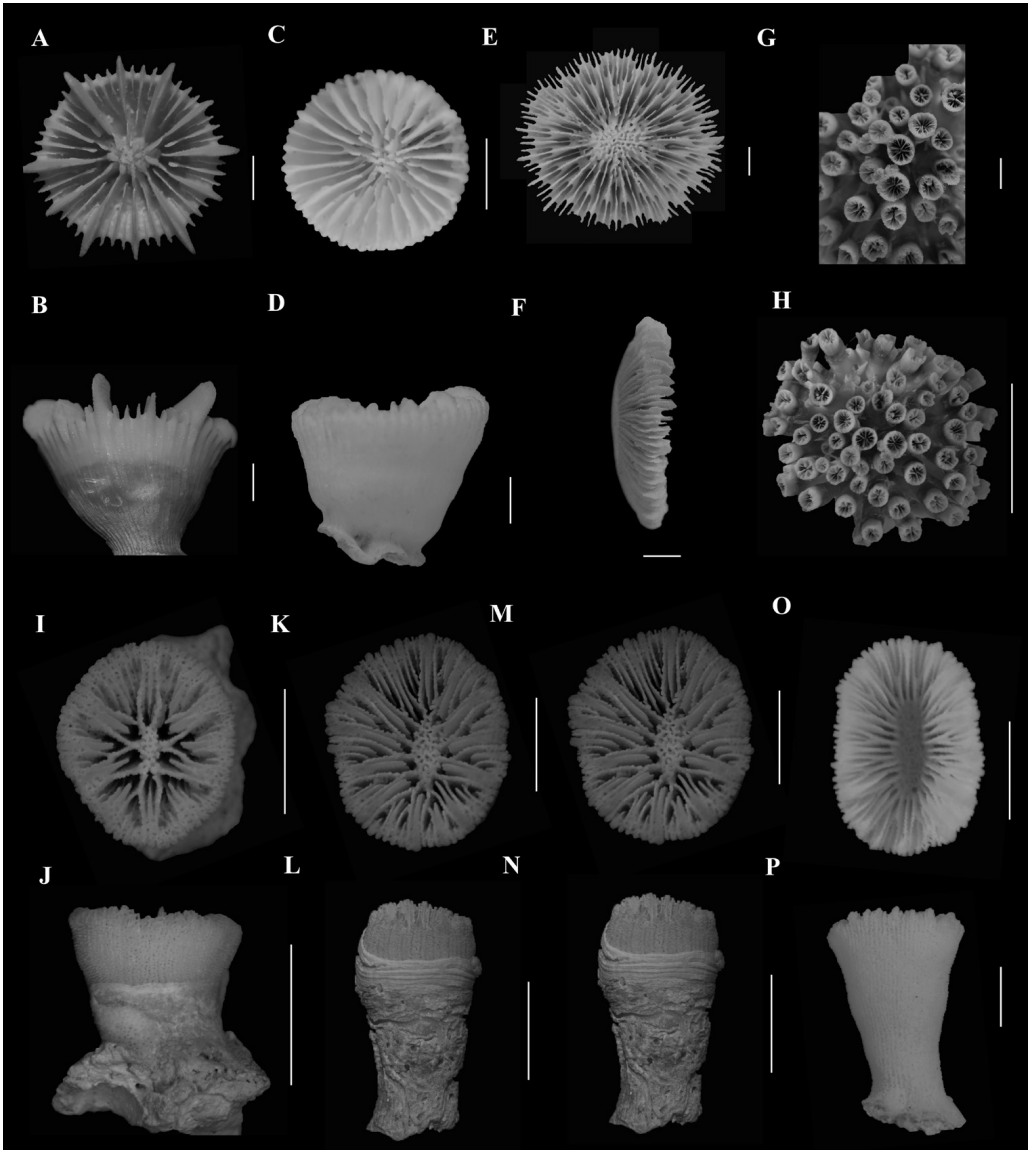
**A**, **D***Vaughanellaconcinna***A**, **B** (MN_SM134, off Port Edward, 900 m) **A** calicular view **B** lateral view **C**, **D** (SAMC_A072973, locality data unknown, 517 m) **C** calicular view **D** lateral view **E**, **F***Deltocyathusrotulus* (USNM 91550, off Scottburgh, 1360 m) **E** calicular view **F** lateral view **G**, **H***Atlantiadenticulata* sp. nov. (SAMC_A090157, off Gouritsmond, 170 m) **G** close-up of corallites **H** full view **I**, **J**Balanophyllia (Balanophyllia) bonaespei (USNM 1423303, off the Agulhas, 32 m) **I** calicular view **J** lateral view **K**, **N**Balanophyllia (Balanophyllia) capensis**K**, **M** (SAM_H3048, off Cape Point, depth unknown) calicular view **L**, **N** (BMNH_ 1939.7.20.479-500, locality data unknown) lateral view **O**, **P**Balanophyllia (Balanophyllia) diademata (SAMC_A073016, off Richards Bay, 500 m) **O** calicular view **P** Lateral view. Scale bars: 10 mm (**A–H**, **K–P**); 100 mm (**I**, **J**).

#### Family Deltocyathidae Kitahara, Cairns, Stolarski & Miller, 2012

##### Deltocyathus

Taxon classificationAnimaliaScleractiniaDeltocyathidae

Milne-Edwards & Haime, 1848

28A6700B-7E74-5DBA-9B6F-17A0E27A2DE8

###### Diagnosis.

Solitary, discoidal to patellate, usually free. Septotheca costate. Septa arranged in 4–5 cycles, only S_1_ being independent. Pali before all but last cycle. Axial margins of higher cycle pali join to faces of adjacent septa (deltas). Columella papillose.

###### Type species.

*Turbinoliaitalica* Michelotti, 1838, by monotypy. 181

##### Deltocyathus italicus

Taxon classificationAnimaliaScleractiniaDeltocyathidae

(Michelotti, 1838)

A7FA29CC-C5A5-5F59-81E2-473544259F94

Turbinolia italica Michelotti, 1838: 51, pl. 1, fig. 8.
Deltocyathus agassizii . –Moseley 1876: 546, 551 (in part).
Deltocyathus italicus . –Pourtalès 1880: 101 (in part), pl. 1, figs 2, 3. –Moseley 1881: 145–147 (in part). –von Marenzeller 1904a: 281 (in part). –Gravier 1920: 34–36 (in part). –Keller 1975: 177, pl. 2, figs 1–4B. –Kitahara 2007: 502–503. –Kitahara and Cairns 2009: 236.
Deltocyathus sp. cf. D.italicus. –Cairns 1979: 95–97, pl. 17, figs 1–3. –Reyes et al. 2009: 16, fig. 3E, F.
Deltocyathus conicus Zibrowius, 1980: 83–85, pl. 39, figs A–L. –Zibrowius and Gili 1990: 35, pl. 2, figs M–O.

###### Type locality.

Tortona, Italy (Miocene) (Cairns 1979).

###### Type material.

The holotype is lost (Cairns 1979).

###### Material examined.

None.

###### Distribution.

Regional: Western margin of South Africa, off Alexander Bay and Port Nolloth; 882–1412 m (Zibrowius and Gili 1991). Elsewhere: widespread in the Caribbean and Gulf of Mexico (Cairns 1978, 1979; Cairns et al. 2009); off Rio de Janeiro in Brazil; Bermuda (Cairns 1979; Kitahara 2007); Colombia (Reyes et al. 2009); Gulf of Gasco; Azores; Morocco; Miocene of Italy; Gulf of Guinea; Angola (Zibrowius 1980); 403–2634 m.

###### Remarks.

This species is well described by Cairns (1979) and Zibrowius (1980), who both noted that the corallum is patellate and free, with a prominent conical base bearing no basal scar. The costae are ridged, unequal in size, and with a dentate appearance. The regional occurrence of *Deltocyathusitalicus* is based on the Zibrowius and Gili (1991) record as no other specimens have been examined. Apart from the inter-species variation in the H:D ratio and columella development, highlighted by Zibrowius and Gili (1991), *D.italicus* may be differentiated from the other Atlantic species of *Deltocyathus* in having a strongly conical base (Cairns 1979b; Zibrowius 1980).

##### Deltocyathus rotulus

Taxon classificationAnimaliaScleractiniaDeltocyathidae

(Alcock, 1898)

EB4211EC-2917-5E2C-85F3-0914E866972B

Fig. 7E, F

Trochocyathus rotulus Alcock, 1898: 16, pl. 2, figs 1, 1A.
Deltocyathus fragilis Alcock, 1902a: 99, 100. –Alcock 1902c: 21, pl. 1, figs 15, 15A.
Deltocyathus rotulus . –van der Horst 1931: 6. –Gardiner and Waugh 1938: 196. –Yabe and Eguchi 1937: 129. –Keller 1982: 50. –Cairns and Keller 1993: 245, pl. 5, fig. I. –Cairns 1994: 55, 56, pl. 24, figs J, K. –Cairns and Zibrowius 1997: 125, 126, fig. 16A–C. –Cairns 1999a: 91–92. –Cairns 2004a: 280. –Kitahara and Cairns 2009: 238, fig. 1B. –Kitahara and Cairns 2021: 399, 401, figs 204, 216A–D.

###### Type locality.

North of Maldive Atoll, Flores Seas (HMS ‘Investigator’ stn. 216: 7°24'S, 118°15.2'E); 794 m (Alcock 1898; Kitahara and Cairns 2009).

###### Type material.

The holotype is presumed to be deposited at the IM (Cairns 2004a; Kitahara and Cairns 2009).

###### Material examined.

**USNM 91550 (1 specimen)**: Eastern margin, 28 km from Scottburgh/21 km off Mkomazi, 30°11'59.99"S, 32°01'00.00"E; 1360 m.

###### Description.

Corallum discoidal, unattached, with a flat to slightly bowl-shaped base. Calice slightly elliptical (GCD:LCD = 1.2), with a lanceted and scalloped calicular margin. Specimen examined 30.4 × 25.5 mm in CD. All costae serrated and prominent at calicular margin. Intercostal spaces deep and wider at calicular margin, becoming progressively narrower towards base. Costae granular, resulting in a serrated costal margin. Corallum white, being light reddish brown around columella.

Septa hexamerally arranged in five cycles according to the formula: S_1–2_ > S_3_ > S_4_ > S_5_ (total of 96 septa). S_1–2_ appearing independent septa, but fusing to neighbouring septa through porous connections. S_1–2_ moderately exsert, bear pali, each of which is separated from its septum by a notch. S_3_ ¾ the width of S_1–2_, less exsert, and sometimes each bearing a small palus. S_4_ less wide than S_3_, but equally exsert, and each bearing a tall palus, which form a distinctive crown around columella. S_5_ rudimentary. All septa fuse to adjacent septa, but position of fusion varies: S_1–4_ join neighbouring septa near columella whilst S_5_ join S_4_ near calicular margin. P_1–3_ small, with P_1–2_ being ¼ or ½ the size of P_4_, and P_3_ consistently half the size of P_1–2_, positioned at porous fusions. P_4_ positioned further away from columella, alluding to a crown of 24 pali. Upper margins of all septa smooth. Fossa shallow, containing a papillose columella composed of irregularly shaped papillae.

###### Distribution.

Regional: Eastern margin of South Africa, off Scottburgh; 1360 m. Elsewhere: Mozambique; Zanzibar (Cairns and Keller 1993); Maldives (Alcock 1898; Gardiner and Waugh 1938); Gulf of Aden (Gardiner and Waugh 1938); Sri Lanka (van der Horst 1931); Tanzania (Gardiner and Waugh 1938); Japan (Cairns 1994); Philippines; Indonesia; Malaysia (Alcock 1902c; Cairns and Zibrowius 1997); Vanuatu; Wallis, and Futuna region (Cairns 1999a); Australia (Cairns 2004a); New Caledonia (Kitahara and Cairns 2009, 2021); 210–2340 m.

###### Remarks.

Among the *Deltocyathus* s from South Africa, the Indo-Pacific *D.rotulus* differs from the Atlantic *D.italicus* in having a large and discodial corallum, a total of five cycles of septa (the highest cycle sometimes incomplete), costae giving a serrated appearance, and the reddish brown pigmentation around the columella.

#### Family Dendrophylliidae Gray, 1847

##### Atlantia

Taxon classificationAnimaliaScleractiniaDendrophylliidae

López & Capel, 2020

9E3F6876-FBC2-5F01-A346-86E1EAC9265C

###### Diagnosis.

Colonies bushy, phaceloid to dendroid, achieved by extra-tentacular budding (frequently from theca of a parent corallite at an acute angle). No epitheca. Septa normally arranged and granular. Columella poorly to moderately developed. Gender: feminine.

###### Type species.

*Atlantiacaboverdiana* (Ocaña & Brito, 2015), by subsequent designation (Capel et al. 2020).

###### Remarks.

Although *Atlantia* resembles *Cladopsammia* Lacaze-Duthiers, 1897; *Astroides* Quoy & Gaimard, 1827; *Enallopsammia* Sismonda, 1871; *Tubastraea* Lesson, 1829; and *Dendrophyllia* de Blainville, 1830 in having new corallites budding from the common basal coenosteum of colony or from the edge zone of corallites, it differs by: (i) always being attached (ii) having normally arranged septa, (iii) poorly developed, and (iv) porosity of corallum consistently uniform (Capel et al. 2020).

##### Atlantia denticulata

Taxon classificationAnimaliaScleractiniaDendrophylliidae

Filander & Kitahara sp. nov.

87C1A4DD-55C7-5107-8391-41B0CB4A05AF

http://zoobank.org/B9DAF54B-6F94-4B92-B70D-7E96E980C812

Fig. 7G, H

###### Type locality.

Off Gouritsmond, Southern margin of South Africa (FV ‘Compass Challenger’, 35°31'52.31"S, 22°07'25.28"E); 170 m.

###### Material.

***Holotype***: SAMC_A090157, Southern margin, 132 km from Gouritsmond/143 km off Goukou Estuary, 35°31'52.31"S, 22°07'25.28"E; 170 m. ***Paratypes***: DEFF/SAEON_CCH009 (15 specimens): Southern margin, 132 km from Gouritsmond/133 km off Gourits Estuary, 35°31'52.31"S, 22°07'25.28"E; 170 m. DEFF/SAEON_A32786 (3 specimens): Southern margin, 145 km from Gouritsmond/152 km off Goukou Estuary, 35°39'19.79"S, 22°02'10.68"E; 175 m. UCTES_SST91P (4 specimens): Southern margin, other locality data unknown.

###### Etymology.

The species name *denticulata* (derived from the Latin *dens* for small tooth + suffix –*ātus*) refers to the axial margins of S_3_ being dentate.

###### Description.

Corallum phaceloid to dendroid. Budding extra-tentacular from base or theca of parent corallite. Holotype consists of 84 corallites, and is 84.6 mm in H. Calice circular to elliptical (GCD:LCD = 1.0–1.1). Epitheca absent. Theca porous, especially near calicular margin. Costae thick, equal in width, granular, and extend to base. Intercostal striae deep. Corallum white.

Septa hexamerally arranged in a normal fashion, with four complete cycles according to the formula: S_1_ > S_2_ > S_3_ > S_4_ (48 septa). S_1_ almost meet opposite septa with vertical axial margins. S_2_^1^/_3_ to ½ width of S_1,_ also with vertical axial margins. S_3_^1^/_3_ the width of S_2_ and have slightly laciniate to dentate axial margins. S_4_ rudimentary, bearing dentate to laciniate axial margins. Septa not exsert. Septal faces bear granules sparsely arranged. Fossa deep, containing a rudimentary columella.

###### Distribution.

Regional: Southern margin, off Gouritsmond; 170 m. Elsewhere: Only known from the type locality.

###### Remarks.

The genus *Atlantia* was recently described by Capel et al. (2020), who outlined the significance of the normally arranged septa (i.e., Pourtalès Plan absent), poorly developed columella, and uniform corallum porosity in distinguishing the genus from other dendrophylliids. The colonies examined herein represents a new species in the genus. *Atlantiadenticulata* sp. nov. differs from the type species (*A. caboverdiana)* in its primary septa extending further into columella (almost meeting with other primaries), tertiary septa never fusing to neighbouring primaries, the rudimentary nature of the columella, and the axial margins of S_3_ consistently being dentate to laciniate.

##### Balanophyllia

Taxon classificationAnimaliaScleractiniaDendrophylliidae

Wood, 1844

B69E9BF5-02F4-5CB7-B582-26D5A8466DCC

###### Diagnosis.

Corallum solitary, turbinate to trochoid, fixed or free. Costae usually well developed. Synapticulotheca especially well developed near calice. Septa arranged in Pourtalès plan. Pali may or may not be present. Columella spongy.

##### Balanophyllia (Balanophyllia)

Taxon classificationAnimaliaScleractiniaDendrophylliidae

Wood, 1844

F1F135AD-FA50-58DE-B77B-FE0549B578BB

###### Diagnosis.

Having a conical corallum firmly attached through a polycyclic base.

###### Type species.

*Balanophylliacalyculus* Wood, 1844, by monotypy.

##### Balanophyllia (Balanophyllia) bonaespei

Taxon classificationAnimaliaScleractiniaDendrophylliidae

van der Horst, 1938

69226F05-9482-5E27-ABD5-2AA0754467FC

Fig. 7I, J

Balanophyllia bonaespei van der Horst, 1938: 142–145, pl. 5, figs 2–5.

###### Type locality.

Oudekraal, Cape Peninsula, South Africa, depth unknown (van der Horst 1938).

###### Type material.

Unknown.

###### Material examined.

USNM 1423303 (neotype: 1 specimen): Southern margin, 11 km off Agulhas/9 km off De Mond-Heuningnes Estuary, 34°47'12.12"S, 20°08'35.87"E; 32 m.

###### Description.

Corallum trochoid and fixed to substrate by a thin encrusting base. Calice circular to elliptical (GCD:LCD = 1.2). Only specimen examined (USNM 1423303) 9.0 × 7.8 mm in CD and 9.3 mm in H. Epitheca thin from middle corallum to encrusting base. Epitheca bear transverse ridges. Costae conspicuous near calicular margin, being equal in width, granular, and porous. Intercostal striae deep and as porous as costae. Corallum white.

Septa hexamerally arranged in a prominent Pourtalès plan, with five cycles, the last cycle being incomplete, according to the formula: S_1_ > S_2_ ≥ S_4_ > S_3_ = S_5_ (≤ 55 septa). S_1–2_ independent and not reaching columella. S_1_ most exsert, and have straight to dentate axial margins. S_2_ slightly less exsert, and ^1^/_3_ smaller than S_1_, also with straight to dentate axial margins. S_3–4_ progressively less exsert. S_3_^1^/_3_ the width of S_2_, and displaying the most dentate axial margins. S_4_ dimorphic in size: in half-systems without S_5,_ S_4_ are as wide as S_2_. Whilst in half-systems with S_5_, S_4_ neighbouring S_1_ as wide as S_3_ and S_4_ neighbouring S_2_ as wide to slightly less wide than S_2_. Furthermore, half-systems without S_5_, S_4_ fuse before S_2_ and extend to columella as one septum, but in complete half-systems, S_5_ joins in front of S_2_ and extend to columella as one septum. S_5_, if present, similar in size as S_3_. Fossa of moderate depth, containing a slender and elongate spongy columella aligned with GCD.

###### Distribution.

Regional: Western (van der Horst 1938) and Southern margin of South Africa, extending from off Oudekraal (van der Horst 1938) towards the Agulhas; 32 m. Elsewhere: Only known from South Africa.

###### Remarks.

Only one specimen of Balanophyllia (B.) bonaespei has been collected subsequent to its original description. Since the type is untraceable, we therefore assign this examined specimen as a neotype here. The examined specimen agrees with van der Horst’s (1938) description and illustration in that S_5_ (when present in half-system) are arranged in a Pourtalès plan, fusing with S_3_ in front of S_4_, and then merging with each other before meandering towards S_2_. This new record further extends the distribution of *B.bonaespei* to the Southern margin of South Africa. *Balanophylliabonaespei* closely resembles *B.capensis*, and distinguishing features are outlined in the remarks section of that species.

##### Balanophyllia (Balanophyllia) capensis

Taxon classificationAnimaliaScleractiniaDendrophylliidae

Verrill, 1865

CAC282C0-AC4C-505B-8E97-751C6A2244CF

Fig. 7K, N

Balanophyllia capensis Verrill, 1865: 149. –van der Horst 1938: 140–142. pl. 6, figs 1–6. –Boshoff 1981: 40. –Cairns 1999b: 25.
Balanophyllia bonaespeii . –Boshoff 1981: 40.
Balanophyllia cummingii . –Boshoff 1981: 41.
Balanophyllia (Balanophyllia) capensis . –Cairns 2001: 16.

###### Type locality.

Off Simonstown, South Africa; 567 m (Verrill 1865; van der Horst 1938).

###### Type material.

The holotype is deposited at the YPM (Gall 2020).

###### Material examined.

DEFF/SEAON**_**A33997 (2 specimens): Southern margin, 39 km from Cape Padrone/40 km off Boknes Estuary, 34°03'53.52"S, 26°42'11.58"E; 100 m. DEFF_BD17.INV02B (2 specimens): Southern margin, 27 km off Cintsa Mouth/25 km off Bulura Estuary, 33°01'22.58"S, 28°17'18.05"E; 122 m. DEFF_NANSEN–INV 16 (1 specimen): Eastern margin, 16 km from Scottburgh/off Mahlongwana Estuary, 30°18'01.19"S, 30°54'47.40"E; 226 m. **ORI_EIa3 (28 specimens)**, ORI**_**EIa4 (1 specimen), ORI**_**EIa5 (1 specimen): Eastern margin, other locality data unknown. SAMC_A073003 (2 specimens): Southern margin, other locality data unknown. SAMC_A073020 (8 specimens): Southern margin, Wavecrest Rocks; depth unknown. SAMC_A073032 (1 specimen): Eastern margin, 9 km from Shaka’s Rock/12 km off Mhlali Estuary, 29°32'06.00"S, 31°19'47.99"E; 50 m. SAMC_A073034 (12 specimens): Eastern margin, 53 km from Shaka’s Rock/46 km off Zinkwasi Estuary, 29°33'29.87"S, 31°46'59.88"E; 180 m. SAMC_A073080 (3 specimens): Eastern margin, 49 km from Mtunzini/42 km off Nyoni Estuary, 29°21'24.12"S, 31°56'12.11"E; 180 m. SAMC_A073111 (1 specimen): Southern margin, off Buffalo Bay; 10 m. SAMC_A073121 (2 specimens): Eastern margin, 19 km from Shaka’s Rock/off Mhlali Estuary, 29°32'53.88"S, 31°25'30.00"E; 65 m. SAMC_A073122 (2 specimens): Eastern margin, 39 km from Port St. Johns/13 km off Mkweni Estuary, 31°30'06.11"S, 29°55'12.00"E; 200 m. SAMC_A073163 (2 specimens): Eastern margin, 18 km from Coffee Bay/16 km off Mdumbi Estuary, 32°01'59.87"S, 29°19'05.88"E; 200–210 m. SAMC_A073174 (8 specimens): Eastern margin, 14 km from Coffee Bay/9 km off Mdumbi Estuary, 31°58'48.00"S, 29°16'48.00"E; 90 m. SAMC_A073188 (1 specimen): Western margin, 31 km from Cape Point/13 km off Krom Estuary, 34°09'24.00"S, 18°16'29.99"E; 75 m. SAMC_A073194 (1 specimen): Eastern margin, 46 km from Gonubie/5 km off Berg River V Estuary, 32°57'11.87"S, 28°02'48.12"E; 30 m. SAMC_A073201 (2 specimens): Eastern margin, 10 km from Shaka’s Rock/12 km off Mhlali Estuary, 29°31'59.88"S, 31°19'59.88"E; 51 m. SAMC_A073224 (1 specimen): Southern margin, Cape Point; 13 m. SAMC_A073227 (5 specimens): Southern margin, 18 km from Pringle Bay/16 km off Buffels Oos Estuary, 34°12'36.00"S, 18°46'54.00"E; 40 m. SAMC_A073229 (2 specimens): Southern margin, False Bay; depth unknown. SAMC_A073230 (2 specimens): Southern margin, False Bay; depth unknown. SAMC_A073231 (12 specimens): Southern margin, False Bay; depth unknown. SAMC_A073234 (1 specimen): Southern margin, False Bay; depth unknown. SAMC_A073244 (2 specimens): Southern margin, other locality data unknown. SAMC_A073246 (1 specimen): Eastern margin, 34 km from Coffee Bay/7 km off Ntlonyane Estuary, 32°15'11.99"S, 28°57'42.00"E; 47 m. SAMC_A090104 (1 specimen): Southern margin, 24 km from Pringle Bay/21 km off Buffels Oos Estuary, 34°09'18.00"S, 18°49'36.00"E; 18 m. SAMC_A090106 (1 specimen): Western margin, off Paternoster; depth unknown. SAMC_A090107 (1 specimen): Southern margin, 15 km off Arniston/21 km off De Mond-Heuningnes Estuary, 34°46'59.99"S, 20°19'00.00"E; 80 m. SAMC_A090108 (3 specimens): Southern margin, False Bay; depth unknown. SAMC_A090109 (1 specimen): Southern margin, 8 km from Port Elizabeth/3 km off Bakens River Estuary, 33°58'05.99"S, 25°38'53.99"E; 9 m. SAMC_A090110 (1 specimen): Southern margin, 11 km off Pringle Bay/8 km off Buffels Oos Estuary, 34°16'29.99"S, 18°49'29.99"E; 14–17 m. SAMC_A090111 (6 specimen): Southern margin, 19 km from Pringle Bay/16 km off Buffels Oos Estuary, 34°12'36.00"S, 18°46'41.99"E; 40 m. SAMC_A090120 (2 specimens): Western margin, 16 km from Cape Town/off Diep Estuary, 33°58'59.99"S, 18°21'00.00"E; 17 m. SAM_H1368 (9 specimens): Southern margin, 29 km from Kenton On Sea/off Boesmans Estuary, 33°53'39.99"S, 26°51'00.00"E; 121 m. SAM_H1371 (1 specimen): Southern margin, 3 km from East London/2 km off Buffalo Estuary, 33°00'48.00"S, 27°55'18.73"E; intertidal. SAM_H1377 (1 specimen): Southern margin, 28 km from Port Alfred/3 km off Old Woman’s Estuary, 33°30'00.00"S, 27°08'59.99"E; 93 m. SAM_H1383 (1 specimen): Southern margin, 18 km from Cape Padrone/30 km off Boknes Estuary, 33°49'00.00"S, 26°16'59.99"E; 65 m. SAM_H1402 (2 specimens): Southern margin, 16 km from Cape Padrone/28 km off Boknes Estuary, 33°54'15.00"S, 26°22'59.99"E; depth unknown. SAM_H1423 (1 specimen): Southern margin, off Great Fish Point Lighthouse, 183 m. SAM_H1438 (1 specimen): Southern margin, 8 km from Pringle Bay/6 km off Buffels Oos Estuary, 34°17'55.37"S, 18°49'10.85"E; 33 m. SAM_H1471 (3 specimens): Eastern margin, 27 km from Mtunzini/25 km off Matigulu Estuary, 29°10'36.00"S, 31°51'00.00"E; 115 m. SAM_H1479 (3 specimens): Southern margin, 11 km from East London/5 km off Gouda Estuary, 33°04'59.99"S, 27°49'29.99"E; 146–283 m. SAM_H1483 (9 specimens): Eastern margin, 27 km from Mtunzini/25 km off Matigulu Estuary, 29°10'36.00"S, 31°51'00.00"E; 115 m. SAM_H1484 (2 specimens): Western margin, 14 km from Cape Town/8 km off Diep Estuary, 33°53'59.99"S, 18°23'59.99"E; 40 m. SAM_H1501 (1 specimen): Eastern margin, 207 km from Coffee Bay/off Mdumbi Estuary, 33°12'00.00"S, 30°49'00.00"E; 73 m. SAM_H3043 (6 specimens): Southern margin, 28 km from Port Alfred/3 km off Old Woman’s Estuary, 33°30'00.00"S, 27°08'59.99"E; 93 m. SAM_H3044 (9 specimens): Southern margin, 17 km from Cape Point/7 km off Elsies Estuary, 34°12'36.38"S, 18°27'45.71"E; 31 m. SAM_H3045 (1 specimen): Southern margin, 29 km from Kenton On Sea/off Boesmans Estuary, 33°53'39.99"S, 26°51'00.00"E; 121 m. SAM_H3046 (3 specimens): Southern margin, 6 km from Kenton On Sea/5 km off Boknes Estuary, 33°43'07.59"S, 26°37'37.95"E; 90 m. SAM_H3047 (6 specimens): Southern margin, 2 km from Mosselbaai/10 km off Hartenbos Estuary, 34°10'37.57"S, 22°09'19.14"E; 54 m. SAM_H3048 (1 specimen): Southern margin, 20 km from Cape Point/4 km off Elsies Estuary, 34°11'30.71"S, 18°25'55.55"E; depth unknown. SAM_H3143 (1 specimen): Southern margin, 177 km from Gonubie/18 km off Berg River V Estuary, 33°04'36.00"S, 28°06'35.99"E; 90 m. SAM_H3144 (19 specimens): Southern margin, 47 km from Port Alfred/14 km off Mgwalana Estuary, 33°30'18.00"S, 27°22'05.99"E; 80 m. SAM_H3145 (19 specimens): Southern margin, 15 km from Port Alfred/11 km off Riet Estuary, 33°39'18.00"S, 27°01'36.00"E; 90 m. SAM_H3146 (1 specimen): Southern margin, 46 km from Port Alfred/12 km off Mgwalana Estuary, 33°29'24.00"S, 27°21'11.99"E; 80 m. SAM_H3367 (2 specimens): Southern margin, 15 km from Port Elizabeth/16 km off Bakens River Estuary, 33°49'59.99"S, 25°40'00.00"E; depth unknown. SAM_H3835 (1 specimen): Southern margin, 21 km from East London/18 km off Buffalo Estuary, 33°08'59.99"S, 28°01'59.99"E; 84 m. USNM 91776 (9 specimens): Eastern margin, 26 km from Durban/22 km off Beachwood Mangroves, 29°48'00.00"S, 31°16'00.00"E; 232 m.

###### Imagery data.

BMNH 1939.7.20.479–500 (2 specimens), SS ‘Valdivia’ (1 specimen): South Africa, other locality data unknown. MN_SM179 (1 specimen): Southern margin, 47 km from Port Alfred/14 km off Mgwalana Estuary, 33°30'18.00"S, 27°22'05.99"E; 80 m. YPM 6827 (holotype): Western margin, off SIMONSTOWN; 567 m.

###### Description.

Corallum ceratoid to trochoid, straight to slightly curved, and attached through a robust pedicel (PD:GCD = 0.4–0.7) that expands into a thin encrusting base. Calice circular to elliptical (GCD:LCD = 1.1–1.3), calicular margin finely serrate. Largest specimen examined (SAM_H3146) 16.1 × 13.0 mm in CD, and 31.9 mm in H. Thick epitheca covering most of corallum, with transverse ridges. Costae conspicuous near calicular margin, equal in width, slightly convex, separated by porous and thin intercostal striae, and covered with fine pointed granules. Intercostal striae slightly sinuous. Epitheca slightly more solid than upper theca. Corallum white.

Septa hexamerally arranged in five cycles, the last being incomplete, according to the formula: S_1_ ≥ S_2_ > S_3_ > S_4_ > S_5_ or S_1_ ≥ S_2_ > S_3_ > S_5_ > S_4_ (≤ 74 septa). S_1–2_ independent but not reaching columella. S_1_ most exsert, with straight to dentate axial margins. S_2_ slightly less exsert, sometimes being slightly less wide, also having straight to dentate axial margins. S_3–4_ progressively less exsert. S_3_ half width of S_2_, with most dentate axial margins. S_4_ commonly arranged in a Pourtalès plan: in a half-system a pair of S_4_ fuses before S_3_, and then fuses before S_2,_ before merging to columella. S_4_ axial margins laciniate. Sometimes S_5_ merge before S_3,_ before meeting columella as one septum. In other cases, S_5_ restricted to calicular margin. Fossa of moderately depth, containing a varied slender and elongate spongy columella.

###### Distribution.

Regional: Western to southern margin of South Africa, extending from off Cape Town towards Mtunzini; 9–232 m. Elsewhere: Only known from South Africa.

###### Remarks.

Balanophyllia (B.) capensis has overlapping distribution and common taxonomic characteristics (such as independent S_1–2_ that never meet columella, S_4_ arranged in a Pourtalès plan, and presence of epitheca) with *B.bonaespei*, but may be distinguished by its larger corallum (*B.capensis* with GCD = 16.1 mm vs. *B.bonaespei* GCD = 9.0 mm), and thicker epitheca that does not have transverse ridges.

##### Balanophyllia (Balanophyllia) diademata

Taxon classificationAnimaliaScleractiniaDendrophylliidae

van der Horst, 1927

99B385DF-3481-580A-9ED7-B5AE9F34403B

Fig. 7O, P

Balanophyllia diademata van der Horst, 1927: 4–5, pl. 2, figs 8, 9. –Cairns and Keller 1993: 220.
Balanophyllia (Balanophyllia) diademata . –Cairns 2001: 16.

###### Type locality.

Off the north-west of Port Dunford; 165 m (van der Horst 1927).

###### Type material.

Unknown.

###### Material examined.

DEFF_FHolon–INV 1 (1 specimen): Eastern margin, off Sodwana; 120 m. SAMC_A073016 (1 specimen): Eastern margin, 31 km from Richards Bay/46 km Mlalazi Estuary, 29°00'54.00"S, 32°15'35.99"E; 500 m.

###### Description.

Corallum ceratoid, straight to slightly curved, and attached by a robust pedicel (PD:GCD = 0.5) that expands into a thin encrusting base. Calice slightly elliptical (GCD:LCD = 1.4–1.5). Largest examined specimen (SAMC_A073016) 12.7 × 8.5 mm in CD, 6 mm in PD, and 24.8 mm in H. Examined specimens lack epitheca. Costae corresponding to septa size (with C_3–5_ being narrow), porous, densely covered with granules, and separated by narrow and deep intercostal furrows.

Septa hexamerally arranged in five incomplete cycles according to the formula: S_1_ > S_2_ > S_3_ > S_5_ > S_4_ (≤ 82 septa). S_1–2_ most exsert, independent, and reach columella with straight and laciniate axial margins. Higher cycle septa (S_3–5_) progressively less exsert. S_3_ dimorphic in size: those in half-systems with S_5_ ~ ½ size of S_2_; but ¼ the size of S_2_ in half-systems without S_5_. S_3_ have slightly dentate to laciniate axial margins. S_4_ also dimorphic in size: ¼ width of S_3_ in half-systems with S_5;_ but double the width of S_3_ in half-systems that lack S_5._ A pair of S_4_ merges in front of flanked S_3_ before joining columella as one septum. S_4_ axial margins slightly more dentate than S_3_. S_5_ (when present) has double the width of S_4_, fusing towards flanked S_4_ before extending to columella as one septum. S_5_ bear the most dentate axial margins. All septal faces covered with granules. Septa appear slightly crowded. Fossa of moderate depth, containing a slender, low-profile, and elongated spongy columella.

###### Distribution.

Regional: Eastern margin of South Africa, off Richards Bay extending towards Sodwana; 120–500 m. Elsewhere: Only known from South Africa.

###### Remarks.

The examined material complements van der Horst’s (1927) description of Balanophyllia (B.) diademata, particularly in the irregularity of the number of septal cycles per half-system. However, the examined specimens lack epitheca, whilst van der Horst’s (1927) figured specimen show epitheca reaching ≤ 2.0 mm from calicular margin.

##### Balanophyllia (Balanophyllia) diffusa

Taxon classificationAnimaliaScleractiniaDendrophylliidae

Harrison & Poole, 1909

592A21B0-BA2C-56D5-B5F8-5FF72F503EDC

Fig. 8A, B

Balanophyllia diffusa Harrison & Poole, 1909a: 906, pl. 85, fig. 4A, B. –Gardiner and Waugh 1939: 239–240. –Cairns and Keller 1993: 275, fig. 13 A–D.
Balanophyllia (Balanophyllia) diffusa . –Cairns 2001: 16.

###### Type locality.

Off Mergui Archipelago, Myanmar; 5–37 m (Harrison and Poole 1909).

###### Type material.

Unknown.

###### Material examined.

**USNM 91780 (1 specimen)**: Eastern margin, 27 km south of Ponta Do Ouro/18 km off Kosi Bay Estuary, 27°06'00.00"S, 32°52'58.80"E; 74 m. **USNM 91782 (1 specimen)**: Eastern margin, 26 km from Port St. Johns/off Bulolo Estuary, 29°34'47.99"S, 31°41'59.99"E; 138 m.

###### Description.

Corallum ceratoid to subcylindrical, straight to slightly curved, attached. Calice elliptical (GCD:LCD = 1.1–1.3), with slightly lanceted calicular margin. Largest specimen examined (USNM 91782) 11.9 × 9.2 mm in CD, and 34.0 mm in H. Epitheca absent. Costae well developed throughout corallum, broad, separated by porous and thin intercostal striae, and covered with low-profile granules. Corallum white.

Septa hexamerally arranged in five cycles, last cycle being incomplete, according to the formula: S_1_ ≥ S_2_ > S_4_ > S_3;_ or if S_5_ present S_1–2_ > S_3_ > S_5_ > S_4_ (≤ 81 septa). S_1–2_ most exsert, both independent, sometimes reaching columella with straight axial margins. Higher cycle septa (S_3–5_) progressively less exsert, if at all. S_3_ ½ size (USNM 91780), or only slightly smaller than S_2_ (USNM 91782). S_3_ axial margins straight and vertical. S_4_ dimorphic in size: those neighbouring S_1_ ~ as wide as S_2_; but those neighbouring S_2_ being the same size as S_3_, with dentate axial margins (USNM 91780). In half-systems with S_5,_ S_4_ ~ ½ the width of S_3_, whereas in half systems without S_5_, S_4_ similar in size to S_1_. S_4_ bear straight and slightly dentate axial margins (USNM 91782). In most cases a pair of S_4_ merge before S_3_ and extend to columella as one septum but sometimes the S_5_ is arranged in a Pourtalès Plan. Septal face granulation perpendicularly arranged. Fossa shallow, containing a spongy columella that sometimes is slender and aligned in GCD plane (USNM 91782), or relatively small and restricted to fossa (USNM 91780).

###### Distribution.

Regional: Southern to eastern margin of South Africa, from off Shaka’s Rock extending towards Kosi Bay Estuary (27 km south of Ponta Do Ouro: Mozambique); 74–138 m. Elsewhere: Mozambique (Cairns and Keller 1993); Pemba (Gardiner and Waugh 1939); Tanzania (Cairns and Keller 1993); northern banks of Kenya (Cairns and Keller 1993); Maldives (Gardiner and Waugh 1939); Red Sea (Gardiner and Waugh 1939); 6–274 m.

###### Remarks.

Records of Balanophyllia (B.) diffusa listed herein are those reported by Cairns and Keller (1993), who noted that the quasi-colonial nature of Gardiner and Waugh’s (1939 [page 239])) specimens is a result of specimens settling closer to one another. The largest examined specimen (USNM 91782) has closely packed septa, and a relatively complex and elongated columella, as compared with the other specimen (USNM 91782), which exhibits loosely packed septa and a fairly simple and short columella.

**Figure 8.**
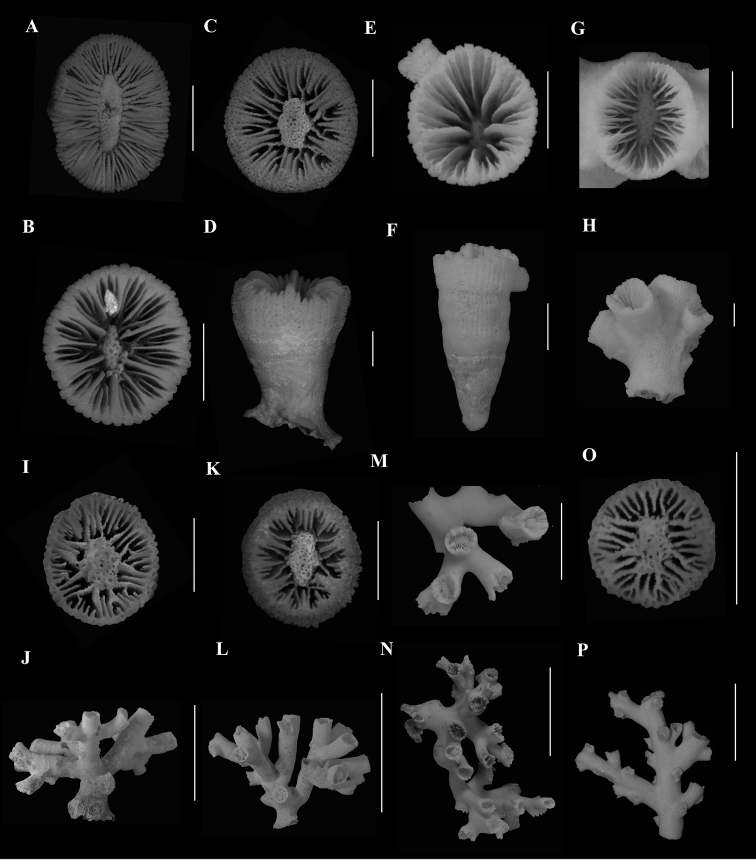
**A**, **B**Balanophyllia (Balanophyllia) diffusa**A** (USNM 91782, off Port St. Johns, 138 m) calicular view **B** (USNM 91780, off Kosi Bay Estuary, 74 m) calicular view **C**, **D**Balanophyllia (Balanophyllia) sp. cf. malouinensis**C** (SAM_H3069, off East London, 146–238 m) calicular view **D** (SAM_H3068, off Mazeppa Bay, 174 m) lateral view **E**, **F**Balanophyllia (Eupsammia) stimpsonii (SAM_H3831, off Cape Point, 97–99 m) **E** calicular view **F** lateral view **G**, **H***Dendrophylliaarbuscula* (SAMC_A073119, off Cape Vidal, 65–70 m) **G** calicular view **H** lateral view **I**, **J***Dendrophylliacladonia***I** (SAM_H1445, off Plettenberg Bay, 146 m) calicular view **J** (SAM_ H2833, off Gonubie, 155 m) lateral view **K**, **L***Dendrophylliacornigera***K** (USNM 91827, off Durban, 232 m) calicular view **L** (SAM_ H3841, off Pringle Bay, depth unknown) lateral view **M**, **N***Dendrophylliadilatata* (SAMC_A073016, off Richards Bay, 500 m) **M** calicular view **N** lateral view **O**, **P***Dendrophylliaijimai***O** (USNM 91844, off Shaka’s Rock, 68–70 m) calicular view **P** (SAMC_A090121, off Port Dunford, 85 m) lateral view. Scale bars: 10 mm (**A**–**I**, **K**, **M**, **O**); 100 mm (**J**, **L**, **N**, **P**).

##### Balanophyllia Balanophyllia sp. cf. malouinensis

Taxon classificationAnimaliaScleractiniaDendrophylliidae

Squires, 1961

FF225C06-1676-557A-9BF3-594D99DB2D5A

Fig. 8C, D

Balanophyllia malouinensis Squires, 1961: 15, 39–40, 46, fig. 5. –Squires 1969: 17–18. pl. 6, map 2. –Cairns 1979: 206. –Cairns 1982: 52–53, pl. 16, figs 4–7, pl. 17, figs 1–3, pl. 18, fig. 7. –Cairns 2005: 43.
Balanophyllia (Balanophyllia) malouinensis . –Cairns 2001: 17.

###### Type locality.

South of East Falkland Island, Atlantic archipelago (52°32'00"S, 61°15'00"W); 358 m (Cairns 1982).

###### Type material.

The holotype is deposited at the AMNH (Cairns 1982).

###### Material examined.

DEFF_BD17–INV02.B (2 specimens): Southern margin, 27 km from Cintsa Mouth/25 km off Bulura Estuary, 33°01'22.58"S, 28°17'18.05"E; 122 m. SAMC_A073031 (3 specimens): Eastern margin, 12 km from Port St. Johns/11 km off Bulolo Estuary, 31°44'17.87"S, 29°32'42.00"E; 300 m. SAMC_A073085 (1 specimen): Eastern margin, 7 km south of Ponta Do Ouro/8 km off Kosi-Kumpungwini (Sifungwe) Estuary, 26°54'40.32"S, 32°55'05.88"E; 47 m. SAMC_A073183 (3 specimens): Eastern margin, 27 km from Richards Bay/40 km off Mlalazi Estuary, 29°00'24.11"S, 32°12'00.00"E; 152 m. SAMC_A073184 (2 specimens): Eastern margin, 39 km from Scottburgh/36 km off Fafa Estuary, 30°34'00.00"S, 31°00'00.00"E; 900–625 m. SAMC_A073186 (1 specimen): Eastern margin, 20 km from Durban/13 km off Mbokodweni Estuary, 30°01'05.87"S, 31°03'11.88"E; 150 m. SAM_H833 (1 specimen): Southern margin, 11 km from East London/5 km off Gouda Estuary, 33°05'03.24"S, 27°49'33.40"E; 146–238 m. SAM_H3065 (1 specimen): Southern margin, 11 km from East London/5 km off Gouda Estuary, 33°05'03.24"S, 27°49'33.40"E; 146–238 m. SAM_H3066 (1 specimen): Southern margin, 11 km from East London/5 km off Gouda Estuary, 33°05'03.24"S, 27°49'33.40"E; 146–238 m. SAM_H3067 (15 specimens): Southern margin, 11 km from East London/5 km off Gouda Estuary, 33°05'03.24"S, 27°49'33.40"E; 146–238 m. SAM_H3068 (3 specimens): Southern margin, 14 km from Mazeppa Bay/20 km off Great Kei Estuary, 32°34'00.00"S, 28°33'00.00"E; 174 m. SAM_H3069 (1 specimen): Southern margin, 11 km from East London/5 km off Gouda Estuary, 33°05'03.24"S, 27°49'33.40"E; 146–238 m. SAM_H3070 (1 specimen): Southern margin, 25 km from Gonubie/24 km off Gqunube Estuary, 33°06'17.99"S, 28°10'59.99"E; 155 m.

###### Description.

Corallum ceratoid to sub-cylindrical, straight to slightly curved, attached to substrate by a robust pedicel (PD:GCD = 0.30–0.6) that expands into a thin encrusting base. Calice circular to elliptical (GCD:LCD = 1.0–1.1), with serrate calicular margin. Largest specimen examined (SAM_H3069) 14.5 × 13.4 mm in CD, 4.0 mm in PD, and 24.0 mm in H. Synapticulotheca well developed and porous, visible at calicular margin. Costae poorly developed, separated by porous and thin intercostal striae, and covered with fine pointed granules. Epitheca thin, irregularly banded. Corallum white.

Septa hexamerally arranged in five cycles, last cycle being incomplete, according to the formula: S_1–2_ > S_4_ > S_3_ > S_5_ (≤ 52 septa). S_1–2_ most exsert, independent, and reach columella deeper in fossa with straight axial margins. Higher cycle septa (S_3–4_) progressively less exsert. S_3_^1^/_4_ the width of S_2_, with straight to slightly sinuous axial margins. S_4_ as wide as S_2_ and arranged in a Pourtalès plan: in each half-system a pair of S_4_ fuses before S_3_ and extend to columella as one septum with laciniate axial margins. S_5_, if present, sometimes restricted to calicular margin, or arranged in Pourtalès plan before common S_4_. Septal faces bear granules sparsely arranged. Fossa of moderate depth, containing a thick and elongate spongy columella.

###### Distribution.

Regional: Southern to eastern margin of South Africa, extending from off East London extending towards Kosi-Kumpungwini (Sifungwe) Estuary (7 km south of Ponta Do Ouro: Mozambique); 47–900 m. Elsewhere: Sub-Antarctic and Antarctica (Cairns 1982); Chile (Cairns et al. 2005); 75–1137 m.

###### Remarks.

Although the examined specimens closely resemble Balanophyllia (B.) malouinensis in their: (i) spongy columella, (ii) synapticulotheca being restricted to the calicular margin, (iii) thin epitheca, and (iv) poorly developed costae, variation in the septal development exists. The examined material consistently has exsert septa (particularly the primaries) unlike those examined by Cairns (1982) which are not exsert. Furthermore, the examined specimens have the septal formula S_1–2_ > S_4_ > S_3_ > S_5,_ whereas that of *B.malouinensis* is S_1–2_ > S_5_ > S_3_ > S_4._ Nonetheless, dendrophylliids are known to exhibit plasticity, making it difficult to evaluate intra-specific variations.

##### Balanophyllia (Balanophyllia) vanderhorsti

Taxon classificationAnimaliaScleractiniaDendrophylliidae

Cairns, 2001

2289CEF3-F26C-521E-9A2C-761CD58F9DC6

Balanophyllia ponderosa van der Horst, 1926: 49–50, pl. 3, figs 6, 7. –Cairns and Keller 1993: 274. –Cairns 1994: 83, pl. 3A, B.
Balanophyllia (Balanophyllia) vanderhorsti Cairns, 2001: 16 (replacement name for junior homonym). –Tachikawa 2005: 11, Pl. 4, figs I, J.

###### Type locality.

Off the Maldives; 53 m (van der Horst 1926).

###### Type material.

The holotype is deposited at the NHMUK (Cairns and Keller 1993).

###### Material examined.

None.

###### Distribution.

Regional: Eastern margin of South Africa, off Richards Bay; 51 m. Elsewhere: Seychelles (van der Horst 1926); Maldives (van der Horst 1926); off Sri Lanka (Alcock 1893); Japan (Eguchi 1968; Cairns 1994; Tachikawa 2005); 14–59 m.

###### Remarks.

No new records of Balanophyllia (B.) vanderhorsti were examined during the present study. Therefore, the occurrence of this species in South Africa is based solely on previous published records. Boshoff’s (1981) record of *B.vanderhorsti* (reported as *B.ponderosa* (which is a junior homonym)) has basal stolons and therefore represents *Rhizopsammia* Verrill, 1870. To reiterate, Cairns and Keller (1993) observed that this South African species closely resembles *B.regalis* (Alcock, 1893) in lacking an epitheca.

##### Balanophyllia (Eupsammia)

Taxon classificationAnimaliaScleractiniaDendrophylliidae

Milne-Edwards & Haime, 1848

35CDD8BB-FA4D-5679-85A5-9CB7C8A34E62

###### Diagnosis.

Corallum solitary, conical, and usually free, having a monocyclic base; corallum often curved or bent. Asexual budding may occur from margin zone. Epitheca present or absent (present in type species); synapticulotheca always costate. Costae bear short, hispid spines. Pourtalès plan present; fifth cycle of septa often present. Columella elongate, spongy. Endotheca absent.

###### Type species.

*Madreporatrochiformis* Pallas, 1766, by subsequent designation (Milne-Edwards and Haime 1850b).

##### Balanophyllia (Eupsammia) stimpsonii

Taxon classificationAnimaliaScleractiniaDendrophylliidae

(Verrill, 1865)

B0F6CAEE-D122-5142-BA3D-9D9247059F45

Fig. 8E, F

Eupsammia stimpsonii Verrill, 1865: 150.
Eupsammia stimpsoniana .– Verrill 1866: 29, pl. 2, figs 3, 3a.
Rhodopsammia socialis Semper, 1872: 260–261, pl. 20, figs 1–4. –Faustino 1927: 229, pl. 75, figs 9–12.– Alcock 1893: 147.
Rhodopsammia affinis Semper, 1872: 261–262, pl. 19, fig. 7A, B.
Rhodopsammia incerta Semper, 1872: 264, pl. 19, fig. 8A, B.– Faustino 1927: 231, pl. 75, figs 3, 4.
Leptopsammia conica van der Horst, 1922: 68–69, pl. 8, figs 14, 15.
Balanophyllia affinis . –Faustino 1927: 228–232, pl. 75, figs 1, 2. –van der Horst 1922: 62. –van der Horst 1931:10. –Gardiner and Waugh 1939: 240.
Balanophyllia stimpsonii . –Zibrowius 1985: 234–235, figs 1–14. –Zibrowius and Grygier 1985: 126–127. –Cairns and Keller 1993: 274. –Cairns and Zibrowius 1997: 176–177. –Cairns 2004a: 313.
Balanophyllia (Eupsammia) stimpsoni . –Cairns 2001: 19. –Kitahara and Cairns 2021: 289–290, figs 142, 152H–J.

###### Type locality.

North China Sea; depth unknown (Verrill 1865).

###### Type material.

Two syntypes are deposited at the YPM (Zibrowius 1985; Cairns 2004a).

###### Material examined.

SAMC_A073157 (2 specimens): Eastern margin, 10 km from Port Edward/24 km off Bilanhlolo Estuary, 31°05'48.11"S, 30°18'47.88"E; 140 m. SAM_H3196 (1 specimen): Eastern margin, 9 km from Shaka’s Rock/2 km off Tongati Estuary, 29°34'00.00"S, 31°10'59.99"E; 66 m. SAM_H3831 (1 specimen): Southern margin, 16 km from Cape Point/18 km off Buffels Oos Estuary, 34°23'17.99"S, 18°39'24.00"E; 97–99 m.

###### Description.

Corallum ceratoid, slightly curved, unattached. Calice circular to slightly elliptical (GCD:LCD = 1.0–1.1), with serrate calicular margin. Largest specimen examined (SAM_H3831) 10.2 × 9.4 mm in CD, and 21.5 mm in H. Theca well developed. Upper theca porous, and lower theca more solid (epitheca). Costae poorly developed near calicular margin, becoming distinctively visible towards base, and covered with fine pointed granules. Epitheca irregularly banded. Intercostal striae thin and porous. Corallum creamy.

Septa hexamerally arranged in four complete cycles according to the formula: S_1_ > S_2_ > S_4_ > S_3_ (48 septa). S_1–2_ most exsert, with straight axial margins. S_1_ independent and extend towards columella. S_2_ slightly less wide than S_1_. Higher cycle septa (S_3–4_) progressively less exsert, and bear laciniate axial margins. S_3_^1^/_3_ the width of S_2_. In complete half-systems a pair of S_4_ fuses before S_3_ and extend to columella as one septum. All septa eventually join columella deep in fossa. Septal faces granular, with granules arranged perpendicular to septal margin. Fossa of moderate depth, containing a low, slender, and elongated spongy columella.

###### Distribution.

Regional: Southern to eastern margins of South Africa, off Cape Point extending towards Shaka’s Rock; 66–99 m. Elsewhere: Philippines; Indonesia (Cairns and Zibrowius 1997); Australia (Cairns 2004a); New Caledonia (Kitahara and Cairns 2021); Chesterfield Islands (Cairns and Zibrowius 1997); Somalia; Mozambique (Zibrowius and Grygier 1985); Seychelles; Reunion (Zibrowius 1985); Gulf of Oman (Zibrowius and Grygier 1985); Sri Lanka (van der Horst 1926; Gardiner and Waugh 1939); Gulf of Manaar (Bourne 1905); Maldives (Gardiner and Waugh 1939); Andaman Sea (Alcock 1893); Mergui Archipelago; Myanmar (Harisson and Poole 1909); 18–95 m.

###### Remarks.

Balanophyllia (E.) stimpsonii resembles *B.carinata* (Semper, 1872) amongst the other four Recent unattached congeners (*B.caribbeana* Cairns, 1977a, *B.imperialis* Kent, 1871, *B.pittieri* Vaughan, 1919, and *B.regalis* (Alcock, 1983) in its septal arrangement in four cycles (in contrast to five incomplete ones). As stated by Cairns and Zibrowius (1997), Balanophyllia (E.) stimpsonii differs from *B.carinata* in having a less compressed corallum, lack of keeled thecal edges, and S_1_ > S_2_. Balanophyllia (E.) stimpsonii is also known to be a host for an ascothoracidan barnacle species (Zibrowius and Grygier 1985). Balanophyllia (E.) stimpsonii is common shallow water Indo-Pacific species, which was previously reported off False Bay and Natal (Zibrowius 1985).

##### Dendrophyllia

Taxon classificationAnimaliaScleractiniaDendrophylliidae

Blainville, 1830

55C16BF3-5CD5-52AC-B170-D09ACD5CCB07

###### Diagnosis.

Colonies formed by extra-tentacular budding, resulting in three general forms: arborescent colonies with axial corallites; small bushy colonies with sparse branching; or dendroid colonies with sympodial branching. All forms originate from a single basal stem. Synapticulothecate. Costae usually clearly defined. Septa arranged in Pourtalès plan. Pali may be present. Columella spongy. Tabular endothecal dissepiments may be present.

###### Type species.

*Madreporaramea* Linnaeus, 1758, by subsequent designation (Milne-Edwards and Haime 1850b).

##### Dendrophyllia arbuscula

Taxon classificationAnimaliaScleractiniaDendrophylliidae

van der Horst, 1922

9F2B0516-F36D-536E-874B-219F7384FE47

Fig. 8G, H

Dendrophyllia arbuscula van der Horst, 1922: 53, pl. 8, fig. 6 (in part: ‘Siboga’ Stn. 277, pl. 8, fig. 6). –Yabe and Eguchi 1942b: 162, 166–167. –Crossland 1952: 92. –Eguchi 1968: C55–C56, pl. C21, figs 5, 13. –Wells 1964: 108. –Pillai and Scheer 1974: 462, fig. 7A. –Pillai 1983: 89. –Veron 1986: 578. –Cairns 1994: 90–91, pl. 38, figs I–L. –Cairns 1995: 125–126, pl. 43, figs E, F. –Ogawa and Takahashi 1995: 17, pl. 1, figs 1–7. –Cairns and Zibrowius 1997: 192–193, fig. 29A–C. –Cairns 1998: 408–409. –Cairns 1999a: 133–134. –Cairns et al. 1999: 26. –Cairns 2001: 34. –Cairns 2004a: 267, 315. –Lam et al. 2009, 732–733, fig. 1E–I.
Dendrophyllia micranthus . –van der Horst 1922: 50.
Dendrophyllia subcornigera Eguchi, 1968: C64, pl. C32, figs 3, 4.
Dendrophyllia horsti Gardiner & Waugh, 1939: 237–238, pl. 2, figs 5, 6. –Fricke and Schuhmacher 1983: 184, fig. 14D.
Dendrophyllia sp. cf. D.horsti. –Cairns and Keller 1993: 278, pl. 13, figs F, I.

###### Type locality.

Off Banda Sea, Indonesia (HMS ‘Siboga’ stns. 260 and 277); 45–90 m (van der Horst 1922).

###### Type material.

Three syntypes are deposited at the ZMA (Cairns 1994).

###### Material examined.

SAMC_A073119 (1 colony): Eastern margin, 39 km from Cape Vidal/29 km off Mgobezeleni Estuary, 27°47'23.99"S, 32°38'53.87"E; 65–70 m. SAM_H3064 (1 colony): Southern margin, 18 km from Cape Padrone/30 km off Boknes Estuary, 33°49'00.00"S, 26°16'59.99"E; 65 m. SAM_H5104 (1 colony): Eastern margin, 59 km from Cape Vidal/9 km off Mgobezeleni Estuary, 27°36'38.45"S, 32°40'02.99"E; 59 m. **USNM 91815 (2 colonies)**: Eastern margin, 90 km from Shaka’s Rock/9 km off Boesmans Estuary, 29°32'12.11"S, 31°19'47.99"E; 50 m. **USNM 91816 (3 colonies)**: Eastern margin, 17 km south of Ponta Do Ouro/10 km off Kosi-Kumpungwini (Sifungwe) Estuary, 27°00'11.87"S, 32°54'18.00"E; 68 m. **USNM 91817 (1 colony)**: Eastern margin, 17 km south of Ponta Do Ouro/11 km off Kosi-Kumpungwini (Sifungwe) Estuary, 27°00'24.11"S, 32°55'12.00"E; 66 m. **USNM 91818 (2 colonies)**: Eastern margin, 19 km south of Ponta Do Ouro/12 km off Kosi-Kumpungwini (Sifungwe) Estuary, 27°01'05.87"S, 32°55'05.88"E; 69–73 m. **USNM 91819 (1 colony)**: Eastern margin, 29 km south of Ponta Do Ouro/19 km Kosi Bay Estuary, 27°06'47.87"S, 32°52'54.12"E; 74 m. **USNM 91820 (1 colony)**: Eastern margin, 41 km south of Ponta Do Ouro/26 km off Kosi Bay Estuary, 27°13'00.11"S, 32°49'41.87"E; 72 m.

###### Description.

Colony small, attached, and bear few corallites, of which one is axial; remaining budding from lower or upper corallum of axial corallite. Primary corallite elongate cylindrical, and firmly attached to substrate through a robust pedicel (PD:GCD = 0.9). Secondary corallites low (< 5 mm in H). Calice slightly elliptical (GCD:LCD = 1.1–1.4), calicular margin lanceted. Largest corallite examined (axial) 12.0 × 10.7 mm in CD, and 31.2 mm in H. Costae conspicuous, broad, equal in width, flat, slightly porous, and granular. Intercostal furrows shallow and quite porous. Corallum white.

Septa hexamerally arranged in four complete cycles according to the formula: S_1_ ≥ S_2_ > S_3_ > S_4_ or S_1_ ≥ S_2_ > S_4_ > S_3_ (48 septa), with S_5_ sometimes present. At calicular margin, upper outer margin of S_4_ fuses to neighbouring septa (S_1_ or S_2_) forming small triangular lancets. S_1_ independent, slightly exsert, and extend towards columella with straight axial margins. S_2_ also independent, slightly less exsert, as wide to only slightly smaller than S_1_, and also bearing straight axial margins. Higher cycle septa (S_3–4_) becoming progressively less exsert, if at all. S_3_ dimorphic in size, being ^1^/_3_ the width of S_2_ or sometimes rudimentary, with dentate to laciniate axial margins. S_4_ arranged in a Pourtalès plan: a pair of S_4_ curves towards common S_3_ fusing before it deep in fossa, may be a ^1^/_3_ wider than S_3_, axial margins dentate to laciniate. Septal faces finely granular. Fossa deep, containing a massive columella usually swirled in a clockwise direction.

###### Distribution.

Regional: Southern and Eastern margin of South Africa, off Cape Padrone extending towards Kosi-Kumpungwini (Sifungwe) Estuary (17 km south of Ponta Do Ouro: Mozambique); 50–73 m. Elsewhere: Japan, China Sea (Cairns 1994); Philippines, Indonesia (Cairns and Zibrowius 1997); Vanuatu (Cairns 1999a); New Caledonia (Kitahara and Cairns 2021); Australia (Cairns 1998); New Zealand (Cairns 1995); Red Sea (Fricke and Schuhmacher 1983); Maldives; Pemba; Tanzania (Gardiner and Waugh 1939; Cairns and Keller 1993); 2–386 m.

###### Remarks.

*Dendrophylliaarbuscula* is historically known from the eastern margin of South Africa. One of the examined specimens (SAM_H3064) extends the regional distribution south towards Cape Padrone. Although Gardiner and Waugh (1939) noted the resemblance of *D.horsti* to *D.arbuscula*, an observation also made by Cairns and Keller (1993), it was only four years later that *D.horsti* was listed as a junior synonym of *D.arbuscula* (Cairns & Zibrowius, 1997). Nonetheless, based on growth form, *D.arbuscula* is within Cairns’(1994) second *Dendrophyllia* morphological group. Two other South African congers (*D.cladonia* van der Horst, 1927 and *D.cornigera* (Lamarck, 1816)) are known to form small and bushy colonies that originate from an axial corallite (with relatively few additional corallites to the primary), however, dissimilarities will be discussed in these species’ accounts.

##### Dendrophyllia cladonia

Taxon classificationAnimaliaScleractiniaDendrophylliidae

van der Horst, 1927

E59D67BD-7D2E-5A71-9BC4-47453CF20399

Fig. 8I, J.

Dendrophyllia cladonia van der Horst, 1927: 3–4, pl. 1, figs 5, 6, pl. 2, fig. 7. –Zibrowius and Gili 1990: 44. –Cairns and Keller 1993: 279. –Cairns 2001: 34.

###### Type locality.

Off Port Shepstone (?), South Africa (RV ‘Pieter Faure’ at ca. 30°44'15.3"S, 30°27'35.0"E); 457 m (van der Horst 1927).

###### Type material.

Type specimen is possibly deposited at the NHMUK (GBIF 2020).

###### Material examine.

DEFF_BD13–INV 03 (6 fragments): Southern margin, 25 km from Cintsa Mouth/off Bulura Estuary, 32°58'59.08"S, 28°19'10.82"E; 104 m. DEFF/SAEON_D00491 (1 fragment): Southern margin, 138 km from Agulhas/145 km off De Mond-Heuningnes Estuary, 35°54'16.19"S, 20°45'46.19"E; 135 m. DEFF/SAEON_D00584 (1 fragment): Southern margin, 56 km from Knysna/57 km off Goukamma Estuary, 34°34'32.40"S, 23°06'04.20"E; 111 m. DEFF/SEAON_D00829 (1 fragment): Southern margin, 153 from Agulhas/157 km off De Mond-Heuningnes Estuary, 35°51'57.60"S, 21°07'14.87"E; 122 m. DEFF/SAEON_D00832 (1 fragment): Southern margin, 171 km from Stilbaai/172 km off Goukou Estuary, 35°54'51.47"S, 21°42'48.59"E; 165 m. DEFF/SAEON_D00851 (1 fragment): Southern margin, 50 km from Agulhas/55 km off De Mond-Heuningnes Estuary, 35°08'14.27"S, 20°24'55.08"E; 113 m. SAMC_A073012 (1 fragment): Eastern margin, 10 km from Port Edward/27 km off Bilanhlolo Estuary, 31°06'46.79"S, 30°17'48.12"E; 120–125 m. SAMC_A073015 (2 fragments): Southern margin, 32 km from Mazeppa Bay/19 km off Mendu Estuary, 32°25'00.11"S, 28°58'18.11"E; 330–340 m. SAMC_A073028 (4 fragments): Eastern margin, 26 km from Cape Vidal/25 km off St Lucia Estuary, 27°54'18.00"S, 32°37'59.87"E; 105 m. SAMC_A073042 (1 fragments): Eastern margin, 53 km from Shaka’s Rock/46 km off Zinkwasi Estuary, 29°32'53.88"S, 31°47'12.11"E; 200 m. SAMC_A073046 (10 fragments): Eastern margin,9 km from Port Edward/9 km off Blinde Estuary, 31°05'23.99"S, 30°18'00.00"E; 125 m. SAMC_A073057 (1 fragment): Eastern margin, 6 km from Cape Vidal/17 km off St Lucia Estuary, 28°08'17.88"S, 32°36'54.00"E; 200 m. SAMC_A073076 (1 fragment): Locality unknown. SAMC_A073165 (1 fragment): Eastern margin, 28 km south of Ponta Do Ouro/17 km off Kosi Bay Estuary, 27°06'18.00"S, 32°52'00.12"E; 50 m. SAMC_A073212 (4 fragment): Eastern margin, 5 km from Cape Vidal/16 km off St Lucia Estuary, 28°07'05.88"S, 32°36'35.99"E; 145 m. SAM_H1362 (1 fragment): Southern margin, 28 km from Port Alfred/3 km off Old Woman’s Estuary, 33°30'00.00"S, 27°08'59.99"E; 90 m. SAM_H1412 (6 fragments): Eastern margin, 2 km from Port Shepstone/Mzimkulu Estuary, 30°44'14.23"S, 30°27'34.72"E; depth unknown. SAM_H1445 (1 fragment): Southern margin, 4 km from Plettenberg Bay/7 km off Piesang Estuary, 34°06'00.00"S, 23°23'59.99"E; 146 m. SAM_H1447 (1 fragment): Eastern margin, 38 km from St. Lucia/31 km off Nhlabane Estuary, 28°40'59.99"S, 32°34'00.00"E; 73 m. SAM_H1513 (1 fragment): Southern margin, 68 km from Kidds Beach/off Gouda Estuary, 33°36'00.00"S, 28°10'59.99"E; 174 m. SAM_H2833 (1 fragment): Southern margin, 25 km from Gonubie/24 km off Gqunube Estuary, 33°06'17.99"S, 28°10'59.99"E; 155 m. SAM_H3042 (1 fragment) Southern margin, 28 km from Port Alfred/3 km off Old Woman’s Estuary, 33°30'00.00"S, 27°08'59.99"E; 93 m. SAM_H3058 (1 fragment): Southern margin,9 km from Pringle Bay/7 km off Buffels Oos Estuary, 34°17'30.00"S, 18°48'00.00"E; 33 m. SAM_H3064 (1 fragment): Southern margin, 18 km from Cape Padrone/30 km off Boknes Estuary, 33°49'00.00"S, 26°16'59.99"E; 65 m. SAM_H3413 (1 fragment): Southern margin, 18 km from Gansbaai/38 km off Ratels Estuary, 34°44'12.00"S, 19°25'36.00"E; 70–50 m. SAM_H3838 (1 fragment): Eastern margin, 7 km from Port Shepstone/off Boboyi Estuary, 30°48'00.00"S, 30°29'05.99"E; depth unknown. **USNM 91823 (48 fragments)**: Eastern margin, 26 km from Port St. Johns/off Bulolo Estuary, 29°34'47.99"S, 31°41'59.99"E; 138 m. **USNM 91825 (7 fragments)**: Eastern margin, 35 km from Port Dunford/38 km off Mlalazi Estuary, 29°10'00.00"S, 32°04'59.99"E; 168 m.

###### Description.

Colony with axial corallite reaching ≤ 93.5 mm. Primary corallite elongate, cylindrical, and firmly attached to substrate through a robust pedicel (PD:GCD = 1.2–1.5). Secondary corallites ceratoid/trochoid to cylindrical. Calice slightly circular to elliptical (GCD:LCD = 1.0–1.3); calicular margin lanceted. Largest corallite examined (axial) 11.1 × 10.7 mm in CD, 17.0 mm in PD, and 93.5 mm in H. Costae conspicuous throughout corallum, broad, equal in width, flat, slightly porous, and granular. Intercostal furrows shallow and quite porous. Corallum white.

Septa hexamerally arranged in four complete cycles according to the formula: S_1_ ≥ S_2_ > S_4_ > S_3_ (48 septa). S_1_ independent and reach columella with vertical and straight axial margins. S_2_ also independent, and as wide to only slightly smaller than S_1_. S_2_ axial margins also vertical and straight. S_3_ smallest septa, and bear dentate to laciniate axial margins. S_4_ arranged in a Pourtalès plan: in each system the S_4_ neighbouring S_1_ fuses before S_2_, before meeting columella. S_4_ also have dentate to laciniate axial margins. Septal faces finely granular. Fossa deep, containing a massive spongy columella.

###### Distribution.

Regional: Southern and Eastern margin of South Africa, off Gansbaai extending towards Kosi Bay Estuary (276 km south of Ponta Do Ouro: Mozambique); 33–340 m. Elsewhere: Mozambique (Cairns and Keller 1993); 49–457 m.

###### Remarks.

As in *Dendrophylliaarbuscula*, *D.cladonia* belongs to the second *Dendrophyllia* group (Cairns 2001), which form small and bushy colonies originating from an axial corallite and with relatively few additional corallites to the primary. *Dendrophylliacladonia* may be distinguished by its Pourtalès plan arrangement, whereby S_4_ adjacent to S_1_ are wider than S_4_ neighbouring S_2_, and merge towards S_2_ before joining the columella (Cairns and Keller 1993), and by its smaller axial corallite GCD (< 9 mm). Under-developed *D.cladonia* may be confused with *Balanophylliacapensis* but can be differentiated by having S_4_ > S_3_ (S_3_ > S_4_ in *B.capensis*). Apart from the septal arrangement, *D.cladonia* can further be distinguished by its S_4_ merging towards S_2,_ and have a more robust pedicel. Although the calicular margin of *D.cladonia* is rarely intact, making it difficult to evaluate septa exsertness, the arrangement of septa assists in distinguishing species from both *D.arbuscula* and *B.capensis* (as highlighted above).

##### Dendrophyllia cornigera

Taxon classificationAnimaliaScleractiniaDendrophylliidae

(Lamarck, 1816)

C02F65C7-B029-5AB8-B153-974F196A3AF6

Fig. 8K, L

Madrepora ramea . –Linnaeus 1758: 797.
Caryophyllia cornigera Lamarck, 1816: 228.
Dendrophyllia cornigera . –Milne-Edwards and Haime 1848c: 100. –Lacaze-Duthiers 1879: 216, pl. 11, fig. 8. –von Marenzeller 1904a: 313, pl. 18, fig. 21. –Moseley 1881: 198. –Gravier 1920: 104, pl 12, figs 186–192. –Joubin 1922: 8, figs 4, 5. –van der Horst 1926: 44. –van der Horst 1928: 1, pl. 1, fig. 1. –Best 1970: 315, fig. 15. –Zibrowius et al. 1975: 97, pl. 5G. –Zibrowius 1979: 19, pl. 1, figs 1, 2. –Zibrowius 1980: 172–175, pl. 87, fig. 1A. –Blainville 1834: 354. –Ocaña et al. 2015: 44–43. –Cairns 2001: 33.

###### Type locality.

Unknown, presumed to be off the Mediterranean (Zibrowius 1980).

###### Type material.

Type specimen is presumably lost (Zibrowius 1980).

###### Material examined.

DEFF/SAEON_D00817 (1 specimen): Southern margin, 23 km from Plettenberg Bay/25 km off Piesang Estuary, 34°16'15.60"S, 23°24'50.40"E; 95 m. SAMC_A072969 (1 specimen): Locality data unknown; 125 m. SAMC_A072982 (2 specimens): Southern margin, 14 km from Pringle Bay/16 km off Buffels Oos Estuary, 34°23'48.00"S, 18°41'05.99"E; 88 m. SAMC_A073003 (9 specimens): Locality data unknown. SAMC_A073010 (2 specimens): Eastern margin, 20 km from Durban/13 km off Mbokodweni Estuary, 30°01'05.87"S, 31°03'11.88"E; 150 m. SAMC_A073023 (3 specimens): Locality data unknown. SAMC_A073031 (3 specimens): Eastern margin, 12 km from Port St. Johns/11 km off Bulolo Estuary, 31°44'17.87"S, 29°32'42.00"E; 300 m. SAMC_A073087 (3 specimens): Eastern margin, 32 km from Port Dunford/33 km off Mlalazi Estuary, 29°11'24.00"S, 31°59'23.99"E; 50 m. SAMC_A073148 (3 specimens): Eastern margin, 26 km from Cape Vidal/25 km off St Lucia Estuary, 27°54'18.00"S, 32°37'59.87"E; 105 m. SAMC_A073174 (1 specimen): Eastern margin, 132 km from Coffee Bay/13 km off Bitou Rivier Estuary, 31°58'48.00"S, 29°16'48.00"E; 90 m. SAMC_A073272 (1 specimen): Locality data unknown; 101 m. SAMC_A090119 (2 specimens): Eastern margin, 28 km south of Ponta Do Ouro/18 km off Kosi Bay Estuary, 27°06'29.99"S, 32°52'54.00"E; 70 m. SAM_H1361 (2 specimens): Southern margin, off CAPE ST BLAIZE; 212 m. SAM_H3062 (1 specimen): Southern margin, 11 km from East London/5 km off Gouda Estuary, 33°05'03.24"S, 27°49'33.40"E; 146–238 m. SAM_H3063 (1 specimen): Southern margin, 28 km from Gonubie/27 km off Buffalo Estuary, 33°09'29.99"S, 28°03'06.00"E; 86 m. SAM_H3147 (1 specimen): Southern margin, 15 km from Port Alfred/11 km off Riet Estuary, 33°39'18.00"S, 27°01'36.00"E; 90 m. SAM_H3148 (1 specimen): Eastern margin, 32 km from Coffee Bay/9 m off Ntlonyane Estuary, 32°15'00.00"S, 29°00'47.99"E; 90 m. SAM_H3412 (1 specimen): Southern margin, 14 km from Gansbaai/31 km off Bot River Lagoon, 34°36'35.99"S, 19°12'36.00"E; 78 m. SAM_H3839 (1 specimen): Southern margin, 9 km from East London/6 km off Buffalo Estuary, 33°04'00.00"S, 27°56'59.99"E; depth unknown. SAM_H3840 (1 specimen): Southern margin, 15 km from Pringle Bay/14 km off Buffels Oos Estuary, 34°15'06.00"S, 18°44'48.00"E; 53 m. SAM_H3841 (1 specimen): Southern margin, 12 km from Pringle Bay/10 km off Buffels Oos Estuary, 34°17'35.99"S, 18°45'00.00"E; depth unknown. SAM_H3842 (1 specimen): Southern margin, 19 km from Pringle Bay/16 km off Buffels Oos Estuary, 34°12'36.00"S, 18°46'41.99"E; 40 m. SAM_H3843 (1 specimen): Southern margin, 12 km from Pringle Bay/10 km off Buffels Oos Estuary, 34°17'35.99"S, 18°45'00.00"E; depth unknown. SAM_H3844 (1 specimen): Southern margin, 12 km from Pringle Bay/11 km off Buffels Oos Estuary, 34°17'59.99"S, 18°44'30.00"E; 40 m. USNM 91827 (6 specimens): Eastern margin, 26 km from Durban/22 km off Beachwood Mangroves, 29°48'00.00"S, 31°16'00.00"E; 232 m.

###### Imagery data.

**BMNH 1939.7.20.8 (2 specimens)**: Eastern margin, off Umhloti River mouth; depth unknown. BMNH 1939.7.20.317 (2 specimens): Locality data unknown. **ZMA 1194 (2 specimens**): Southern margin; 95 m.

###### Description.

Colony bushy, stemming from an elongate, cylindrical, and firmly attached axial corallite that has a robust pedicel (PD:GCD = 1.1). Secondary corallites ceratoid/trochoid to cylindrical. Calice circular to slightly elliptical (GCD:LCD = 1.0–1.1); calicular margin lanceted. Largest corallite examined (axial) 9.3 × 8.3 mm in CD, 9.9 mm in PD, and 55.0 mm in H. Costae conspicuous throughout corallum, broad, equal in width, flat, slightly porous, and granular. Intercostal furrows shallow and quite porous. Corallum white.

Septa hexamerally arranged in four complete cycles according to the formula: S_1_ ≥ S_2_ > S_4_ > S_3_ (48 septa). S_1_ independent, not reaching columella, and bear dentate (occasionally smooth) axial margins. Remaining septa (S_2–4_) also bear dentate axial margins, of which that of S_4_ having conspicuously longer teeth. S_2_ also independent, as wide to only slightly smaller than S_1_. S_3_ least wide septa. S_4_ arranged in a Pourtalès plan: in each system a pair of S_4_ neighbouring S_1_ curves towards common S_2_, fusing with S_4_ closer to S_2_ deeper in fossa before reaching columella. Septal faces finely granular. Fossa moderately deep, containing a spongy columella.

###### Distribution.

Regional: Western to eastern margins of South Africa, from off Pringle Bay extending towards off Kosi Bay Estuary (28 km south of Ponta Do Ouro: Mozambique); 40–300 m. Elsewhere: Mediterranean (Duncan 1873; Ocaña et al. 2015); Morocco (Zibrowius 1983); Celtic Sea (Joubin 1922); off France (Lacaze-Duthiers 1879; Moseley 1881); Bay of Biscay (Milne-Edwards and Haime 1848c); Portugal (Zibrowius 1980); Canary Islands (Gravier 1920); Maldives; Almirantes; and Providence (van der Horst 1926); 89–600 m.

###### Remarks.

*Dendrophylliacornigera* has been systematically recorded in the southwest Indian Ocean (van der Horst 1926), specifically off the southern Agulhas (von Marenzeller 1904a; van der Horst 1927) and KwaZulu-Natal region (van der Horst 1927; Cairns and Keller 1993). Nonetheless, the examined specimens are in accordance with the description of Mediterranean specimens (Zibrowius 1980). *Dendrophylliacornigera* is distinguished from *D.arbuscula* and *D.cladonia* by S_4_ bearing a distinctively long dentate axial margins as compared with S_4_ bearing slightly dentate to laciniate axial margins (*D.arbuscula* and *D.cladonia*).

##### Dendrophyllia dilatata

Taxon classificationAnimaliaScleractiniaDendrophylliidae

van der Horst, 1927

87AC83D0-AD95-5DC2-9EC3-4A61E10354A0

Fig. 8M, N

Dendrophyllia dilatata van der Horst, 1927: 2–3, figs 2–4. –Zibrowius and Gili 1990: 44. –Cairns and Keller 1993: 278–279. –Cairns 1994: 89. –Cairns 2001: 34.

###### Type locality.

Off Richards Bay, South Africa (RV ‘Pieter Faure’ stn. 12103: ca. 28°47'00.0"S, 32°20'00.0"E); 97 m (van der Horst 1927).

###### Type material.

The holotype is deposited at the ZMA (Creuwels 2020).

###### Material examined.

SAMC_A073016 (2 specimens): Eastern margin, 31 km from Richards Bay/46 km Mlalazi Estuary, 29°00'54.00"S, 32°15'35.99"E; 500 m. SAMC_A073068 (1 specimen): Eastern margin, 47 km from Cape Vidal/28 km off Mgobezeleni Estuary, 27°43'11.99"S, 32°40'36.11"E; 100 m.

###### Description.

Colony unattached and arborescent, with sympodial branching formed by extra-tentacular budding. Corallum ceratoid to sub-cylindrical, straight to slightly curved. Largest specimen examined (SAMC_A073016) 105.5 mm in H, with ≤ 30 corallites. Calice elliptical (GCD:LCD = 0.9–1.2), with serrated calicular margin. Epitheca absent. Costae well developed at upper corallum, rounded, granular, non-perforate, and separated by thin, porous, intercostal striae. Corallum white.

Septa hexamerally arranged in four complete cycles according to the formula: S_1–2_ > S_4_ > S_3_ (48 septa). S_1–2_ both independent, reaching the columella with straight axial margins, which may also be finely serrated. S_1_ most exsert, followed by S_2._ S_3_ ~ ^1^/_3_ the width of S_2_. S_4_ slightly wider than flanked S_3,_ arranged in Pourtalès plan: in each half-system a pair of S_4_ fuses before S_3_ with jaggedly dentate axial margins. Fossa deep, containing a large, and slightly raised spongy columella.

###### Distribution.

Regional: Eastern margin of South Africa, off Richards Bay towards Cape Vidal; 97–500 m. Elsewhere: Mozambique (Cairns and Keller 1993); 97–132 m.

###### Remarks.

*Dendrophylliadilatata* resembles *Dendrophyllia* sp. 1 in its growth form: i.e., large, dendroid colonies with fairly regular sympodial arranged corallites (Cairns 1994). However, it can be distinguished by having four cycles of septa as compared with three (*Dendrophyllia* sp. 1), a well-developed Pourtalès plan development (poorly developed in *Dendrophyllia* sp. 1), lacking P_2_ which is present in *Dendrophyllia* sp. 1, and a larger columella (smaller in *Dendrophyllia* sp. 1).

##### Dendrophyllia ijimai

Taxon classificationAnimaliaScleractiniaDendrophylliidae

Yabe & Eguchi, 1934

CBBBFB62-6650-5AE9-AB56-B7056EC4F8F3

Fig. 8O, P

Dendrophyllia ijimai Yabe & Eguchi, 1934: 2026. –Eguchi 1965a: 294, 2 figs. –Eguchi 1968: C65 (in part: pl. C16, figs 1, 2, pl. C22, fig. 1). –Kikuchi 1968: 9, pl. 15, fig. 2. –Eguchi and Miyawaki 1975: 54. –Cairns and Keller 1993: 280, fig. 13G. –Cairns 1994: 89, pl. 38C, F. –Cairns 1999a: 133. –Cairns et al. 1999: 26. –Cairns 2001: 34. –Cairns 2004a: 267, 315. –Kitahara and Cairns 2021: 310, 312, figs 164, 165A–C.
Dendrophyllia micranthus . –Eguchi 1965a: 294, fig. 1. –Eguchi 1968: C66, pl. C24, figs 2, 3.
Dendrophyllia minuscula . –van der Horst 1922: 51–52, pl. 8, fig. 30. –Utinomi 1965: 257. –Boshoff 1981: 42. –Tribble and Randall 1986: 159.
Dendrophyllia subcornigera cylindrica Eguchi, 1968: C64–C65, pl. C32, figs 1, 2.
Dendrophyllia subcornigera . –Wells 1984: 215–216, fig. 5.
Dendrophyllia sp. –Zibrowius and Grygier 1985: 123, 126, figs 22, 23.
Dendrophyllia sp. cf. D.ijimai. –Cairns and Zibrowius 1997: 191–192, fig. 29E.

###### Type locality.

Presumably off Japan (Cairns 1994).

###### Type material.

Presumably lost (Cairns 1994).

###### Material examined.

SAMC_A073008 (4 specimens): Eastern margin, 33 km from Port Dunford/38 km off Mlalazi Estuary, 29°05'30.11"S, 32°09'06.11"E; 95 m. SAMC_A090121 (1 specimen): Eastern margin, off 33 km from Port Dunford/37 km off Mlalazi Estuary, 29°08'59.99"S, 32°05'24.00"E; 85 m. **USNM 91843 (1 specimen)**: Eastern margin, 39 km from Cape Vidal/29 km off Mgobezeleni Estuary, 27°47'21.59"S, 32°39'03.60"E; 62–84 m. **USNM 91844 (1 specimen)**: Eastern margin, 28 km from Shaka’s Rock/19 km off Mdlotane Estuary, 29°26'59.99"S, 31°31'11.99"E; 68–70 m.

###### Description.

Colony composed of one elongate, straight to slightly curved axial corallite, from which secondary corallites bud. Secondary corallites robust and bud in all directions, reaching ≤ 130 mm in H. Tertiary corallites small (< 3 mm in H). Corallites circular to slightly elliptical (GCD:LCD = 1.0–1.1), with lanceted calicular margins Costae well defined, slightly ridged, and highly granular. Intercostal furrows deep and porous. Corallum white.

Septa hexamerally arranged in four cycles, S_5_ occasionally present in some half-systems, in a strongly developed Pourtalès plan according to the formula: S_1_ ≥ S_2_ > S_4_ > S_3_ > S_5_ (≤ 60 septa). S_1_ independent and with straight axial margins. S_2_ as wide to only slightly smaller than S_1_, and have slightly sinuous axial margins. Both S_1_ and S_2_ extend to columella. S_3_ narrowest, also with slightly sinuous axial margins. S_4_ dimorphic in size, with laciniate axial margins: in half-systems without S_5_, S_4_^1^/_5_ smaller than S_2_; however, in half-systems with S_5_, S_4_ half the size of S_4._ S_4_ arranged in Pourtalès plan: curving towards common S_3_, and fusing before extending to columella as one septum. However, in half-systems with S_5,_ the S_5_ is arranged in Pourtalès plan: merging in front of flanked S_4,_ before meandering towards S_3_ and joining S_4_ neighbouring S_2._ Septal faces finely granular. Fossa shallow to moderately deep, with a non-discrete spongy columella.

###### Distribution.

Regional: Eastern margin of South Africa, from off Shaka’s Rock extending towards Cape Vidal; 62–95 m. Elsewhere: Japan (Yabe and Eguchi 1934; Cairns 1994); Philippines; Indonesia (Cairns and Zibrowius 1997); Australia (Cairns 2004a); New Zealand; Red Sea (Scheer and Pillai 1983); Zanzibar (Cairns and Keller 1993); 10–366 m.

###### Remarks.

*Dendrophylliaijimai* is the only *Dendrophyllia* species in the region that has arborescent colonies bearing large axial corallites that give off shorter corallites budding in an irregular form (Cairns 1994). This growth form makes it easily distinguishable from the other South African congeners. Only one other western Pacific species is known to exhibit such a growth form (*D.cribrosa* Milne-Edwards & Haime, 1851) and *D.ijimai* may be distinguished by its non-anastomotic branches (and exsert corallites) which are not flushed as in the case of *D.cribrosa* (see Cairns 1994). As noted by Cairns and Keller (1993), this species may be mistaken with *Tubastraeamicranthus*, but differs in having its septa arranged in a well-developed Pourtalès plan.

##### Dendrophyllia

Taxon classificationAnimaliaScleractiniaDendrophylliidae

sp. 1

DD826A79-4D81-5259-AFF6-333D9A30E268

Fig. 9A, B

###### Material examined.

DSCS–INV 29 (1 fragment): Southern margin,140 km from Agulhas/144 km off Ratels Estuary, 36°02'29.58"S, 19°41'24.61"E; 445–463 m. DSCS–INV 30 (1 fragment Southern margin,140 km from Agulhas/144 km off Ratels Estuary, 36°02'29.58"S, 19°41'24.61"E; 445–463 m. DSCS–INV 31 (1 fragment): Southern margin,140 km from Agulhas/144 km off Ratels Estuary, 36°02'29.58"S, 19°41'24.61"E; 445–463 m. DSCS–INV 33 (1 fragment): Southern margin,140 km from Agulhas/144 km off Ratels Estuary, 36°02'29.58"S, 19°41'24.61"E; 445–463 m. DSCS–INV 35 (1 fragment): Southern margin,140 km from Agulhas/144 km off Ratels Estuary, 36°02'29.58"S, 19°41'24.61"E; 445–463 m. DSCS–INV 44 (1 fragment): Southern margin,140 km from Agulhas/144 km off Ratels Estuary, 36°02'29.58"S, 19°41'24.61"E; 445–463 m. DSCS_INV 527 (1 specimen): Southern margin, 65 km from Cape St. Francis/70 km off Slang Estuary, 34°47'05.01"S, 24°45'42.30"E; 392–418 m. SAMC_A090158 (1 fragment): Southern margin, 116 km from Gouritsmond/off Goukamma Estuary, 35°07'11.34"S, 23°02'41.91"E; 333 m.

###### Imagery data.

SAM_H1441 (in part: 3 fragments): Southern margin, 3 km from Mosselbaai/11 km off Hartenbos Estuary, 34°10'59.99"S, 22°10'00.00"E; 216 m.

###### Description.

Colonies uniplanar with sympodial budding and dendroid, formed by extra-tentacular budding. Calices occur in lateral plane of branching. Diameter of largest basal branch examined (SAMC_A090158) 7.0 mm. Calice circular, with slightly serrate margin, reaching a maximum CD of 5.5 mm. Costae prominent, but occasionally inconspicuous in some calices. Costal granulation consisting of small spines better developed on terminal branches. Intercostal ridges narrow. Corallum white.

Septa hexamerally arranged in three complete cycles according to the formula: S_1_ > S_2_ > S_3_ (≤ 24 septa). S_1_ widest and extend to columella with straight and vertical to slightly concave axial margins. S_2_ ~ ^1^/_2_ width of S_1_, also with straight axial margins, sometimes bearing a palus. S_1–2_ sometimes slightly exsert. S_3_ smallest septa and have laciniate axial margins. All septa joining columella deep in fossa. Poorly developed Pourtalès plan only visible deeper in fossa. Fossa deep, containing a tall and thick lamellar columella.

###### Distribution.

Regional: Southern margin of South Africa, off Agulhas extending towards Gouritsmond; 212–445 m.

###### Remarks.

Specimens examined closely resemble the Atlantic *Dendrophylliaalternata* Pourtalès, 1880

but disagree with its diagnosis in costae being poorly developed, having three cycles of septa, S_1–2_ sometimes being slightly exsert, and its columella being a solid and elongated rod. However, the uniplanar, dichotomous branching, sympodial budding, and spines on corallum resemble *D.alternata*.

**Figure 9.**
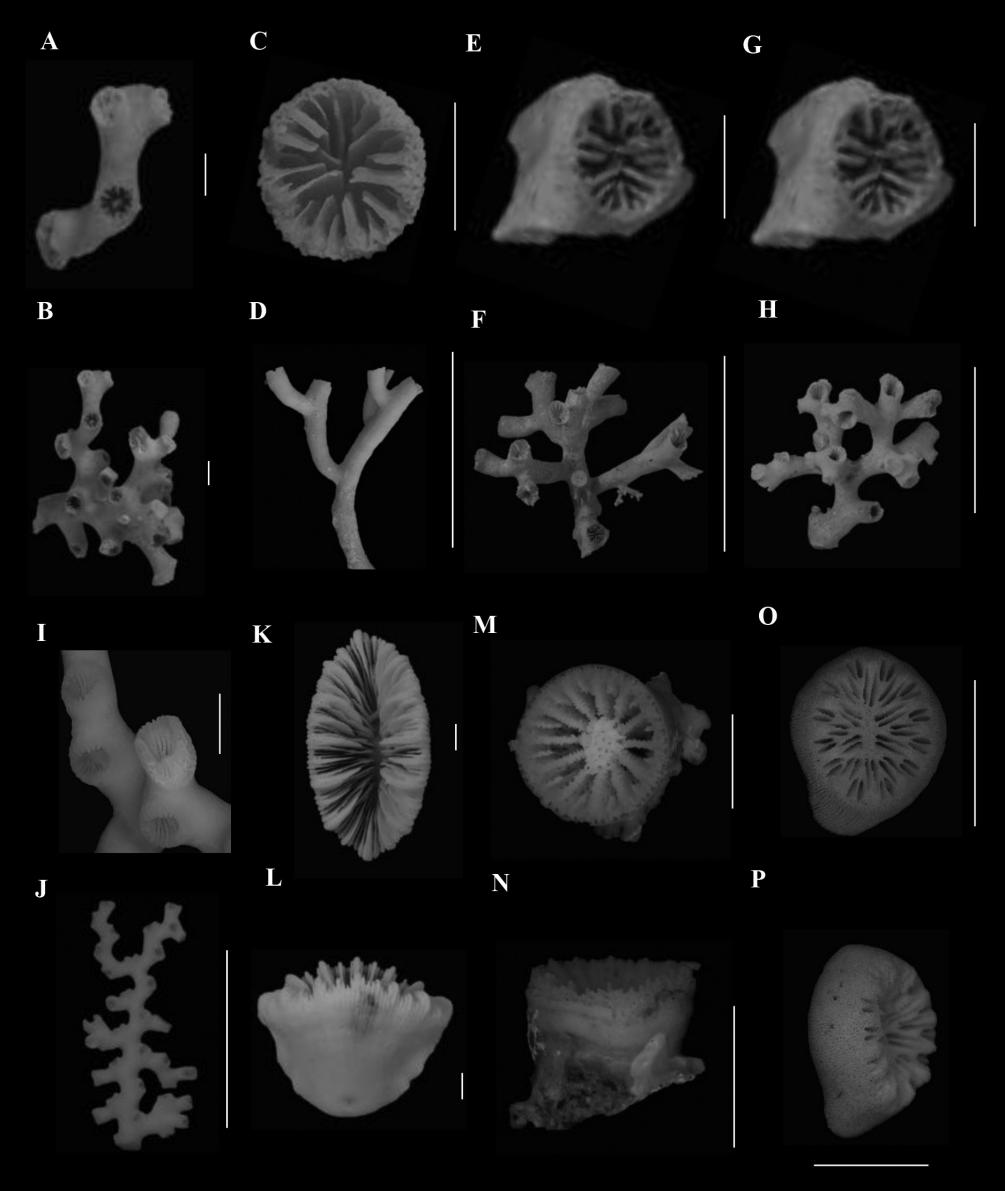
**A**, **B***Dendrophyllia* sp. 1 (SAMC_A090158, off Knysna, 333 m **A** close-up calicular view **B** lateral view **C**, **G***Ednapsammiacolumnapriva* sp. nov. **C**, **D** (SAM_H1441, off Mossel Bay, 212 m) **C** calicular view **D** lateral view **E-G** (SAMC_A090159, off Gouritsmond, 333 m) **E** calicular view **F** lateral view **G** calicular view **H***Enallopsammiapusilla* (DSCS-INV238, off Gouritsmond, 333 m): lateral view. **I**, **J***Enallopsammiarostrata* (SAMC_A073270, off Port St Johns, 200 m) **I** close up calicular view **J** lateral view **K**, **L***Endopachysgrayi* (DEFF_NANSEN-INV 32, off Shaka’s Rock, 185 m) **K** calicular view **L** lateral view **M**, **N***Endopsammiaphilippensis* (DIIIb1, off Durban, 442 m) **M** calicular view **N** lateral view **O**, **P***Heteropsammiacochlea* (EIe1, locality data unknown) **O** calicular view **P** lateral view. Scale bars: 10 mm (**A–C**, **E–I**, **K–P**); 100 mm (**D**, **J**).

##### Ednapsammia

Taxon classificationAnimaliaScleractiniaDendrophylliidae

Filander gen. nov.

0F751A85-EB9E-5FFC-B790-2F1A775CC88D

http://zoobank.org/35B7C13E-13A9-4F89-9291-C9EC65A432F8

###### Diagnosis.

Colony dendroid with sympodial branching, all achieved by extra-tentacular budding (frequently from theca of a parent corallite at an acute angle). Thin epitheca present. Septa normally arranged and granular. Columella absent. Endothecal dissepiments present.

###### Type species.

*Ednapsammiacolumnapriva* Filander, 2020, by original designation.

###### Etymology.

The genus name *Ednapsammia* is to honour the late Dr Edna Molewa, who was instrumental for the declaration of the offshore Phakisa Marine Protected Areas in South Africa. Gender: feminine.

###### Remarks.

*Ednapsammia* gen. nov. closely resembles *Dendrophyllia* in having a dendroid colony formed from sympodially arranged branches and well-developed costae. However, it can be differentiated in lacking a Pourtalès plan and columella. *Ednapsammia* gen. nov. is also morphologically similar to *Atlantia* López & Capel, 2020, a genus that has dendroid to phaceloid colonies and normally arranged septa, but differs in the presence of a thin epitheca, porosity being prevalent near calicular margin, and lacking a columella.

##### Ednapsammia columnapriva

Taxon classificationAnimaliaScleractiniaDendrophylliidae

Filander sp. nov.

2F1B5581-F512-5749-891D-3F0D1D6FE8BA

http://zoobank.org/35B7C13E-13A9-4F89-9291-C9EC65A432F8

Fig. 9C–G

###### Type locality.

Off Knysna, South Africa (RV ‘Algoa’ stn. DCS13: 35°07'11.34"S, 23°02'41.91"E); 333 m.

###### Material examined.

***Holotype***– SAMC_A090159 (1 specimen): Southern margin, 116 km from Gouritsmond/off Goukamma Estuary, 35°07'11.34"S, 23°02'41.91"E; 333 m. ***Paratypes***–DSCS–INV 226 (1 specimen), DSCS–INV 227 (1 specimen), DSCS–INV 229 (1 specimen), DSCS–INV 231 (1 specimen), DSCS–INV 232 (1 specimen), DSCS–INV 235 (1 specimen), DSCS–INV 238 (1 specimen), SAMC_A090149 (1 specimen): Southern margin, 116 km from Gouritsmond/off Goukamma Estuary, 35°07'11.34"S, 23°02'41.91"E; 333 m.

###### Imagery data.

SAM_H1441: Southern margin, 3 km from Mosselbaai/11 km off Hartenbos Estuary, 34°10'59.99"S, 22°10'00.00"E; 216 m.

###### Etymology.

The species name *columnapriva* (derived from Latin *columna* meaning pillar and *privus* meaning deprived of) alludes to the lack of columella.

###### Description.

Corallum dendroid formed by extra-tentacular budding from base and from theca of parent corallite. Holotype consists of 19 corallites, and 54.7 mm in H. Calice circular to elliptical (GCD:LCD = 1.0–1.1), calicular margin finely laciniate. Thin epitheca, predominantly porous near calicular margin. Costae thick, equal in width, granular, extending to base, and separated by deep intercostal striae. Corallum white.

Septa hexamerally arranged in three cycles, last cycle being incomplete, according to the formula: S_1_ > S_2_ > S_3_ (≤ 21 septa). S_1_ extend to fossa, sometimes almost meeting opposite septa. S_2_ ~ ^1^/_3_ smaller than S_1._ Third septal cycle incomplete, usually with only one S_3_ per half-system. S_3_^1^/_3_ the width of S_2_. Primary and secondary septa cycles (S_1–2_) with straight and vertical axial margins, whilst S_3_ bears slightly laciniate axial margins. Fossa deep, columella absent.

###### Distribution.

Regional: Southern margin of South Africa, extending from off Mosselbaai towards Gouritsmond; 216–333 m.

###### Remarks.

Examined specimens are easily distinguished from other colonial dendrophylliids by their septa being hexameral and arranged normally (no Pourtalès plan), in three incomplete cycles. Despite that the imaged specimen (SAM_H1441 [in part: 1 specimen]) was not traceable in the Iziko Museums collection. This record nonetheless confirms that such a form was historically collected off the southern margin of South Africa.

##### Enallopsammia

Taxon classificationAnimaliaScleractiniaDendrophylliidae

Michelotti, 1871

00CFE8A6-569A-5BFD-B3C0-458265F29434

###### Diagnosis.

Colonial. Arborescent colonies formed by extra-tentacular budding. Corallites often, but not always, unifacially arranged. Coenosteum dense, synapticulotheca porous only near calices and on distal branches. Septa arranged normally. Columella small.

###### Type species.

*Coenopsammiascillae* Seguenza, 1864, by monotypy

##### Enallopsammia pusilla

Taxon classificationAnimaliaScleractiniaDendrophylliidae

(Alcock, 1902)

9A6A88DA-7100-5CC4-A8E1-7AC351EED693

Fig. 9H

Dendrophyllia (Coenopsammia) pusilla Alcock, 1902a: 113. –Alcock 1902c: 44, pl. 5, figs 38, 38A.
Dendrophyllia (Coenopsammia) profunda . –Alcock 1902c: 43.
Coenopsammia profunda . –von Marenzeller 1904a: 313–314, pl. 18, fig. 24.
Enallopsammia marenzelleri . –Zibrowius 1973: 49–51, pl. 1, figs 1–7. –Zibrowius 1980: 204–205.
Enallopsammi a sp. cf. E.marenzelleri. –Cairns 1982: 57–58, pl. 18, figs 5–6. –Cairns 1995: 128–129, pl. 44, figs G, H.
Enallopsammia pusilla . –Cairns and Zibrowius 1997: 194, fig. 29F. –Cairns 2004a: 316, fig. 12F, G.

###### Type locality.

Off Sulu Archipelago, Philippines (HMS ‘Siboga’ stn. 95: 5°43.5'00"N, 119°40'00"E); 522 m (Alcock 1902a).

###### Type material.

The holotype and the paratypes are deposited at the ZMA (Cairns and Zibrowius 1997).

###### Material examined.

DSCS_INV 34 (1 specimen): Southern margin,140 km from Agulhas/144 km off Ratels Estuary, 36°02'29.58"S, 19°41'24.61"E; 445–463 m. DSCS_INV 40 (1 specimen): Southern margin,140 km from Agulhas/144 km off Ratels Estuary, 36°02'29.58"S, 19°41'24.61"E; 445–463 m. DSCS_INV 42 (1 specimen): Southern margin,140 km from Agulhas/144 km off Ratels Estuary, 36°02'29.58"S, 19°41'24.61"E; 445–463 m. DSCS_INV 223 (1 specimen): Southern margin, 116 km from Gouritsmond/off Goukamma Estuary, 35°07'11.34"S, 23°02'41.91"E; 333 m. DSCS_INV 225 (2 specimens): Southern margin, 116 km from Gouritsmond/off Goukamma Estuary, 35°07'11.34"S, 23°02'41.91"E; 333 m. DSCS–INV 238 (1 specimen): Southern margin, 116 km from Gouritsmond/off Goukamma Estuary, 35°07'11.34"S, 23°02'41.91"E; 333 m. SAMC_A090153 (1 specimen): 116 km from Knysna/off Goukamma Estuary, 35°07'11.34"S, 23°02'41.91"E; 333 m.

###### Description.

Irregularly shaped colonies formed by extra-tentacular budding, and firmly attached to substrate by an encrusting base. Buds projecting ≤ 3 mm above branch coenosteum. Corallites circular to slightly elliptical (GCD:LCD = 1.0–1.1), calicular margin serrated. Costae well developed on entire branch, slightly convex, and covered with small granules. Intercostal striae deep, thin, and porous. Corallum white.

Septa hexamerally arranged in three complete cycles according to the formula: S_1–2_ > S_3_ (24 septa in two size classes). S_1_ independent, narrow, extending closer to columella deep in fossa with smooth axial margins. S_2_ equal in width to S_1,_ and bearing smooth upper axial margins that become finely serrated deep in fossa. S_3_ slightly less wide than S_1–2,_ sometimes appearing rudimentary, and have dentate axial margins. S_3_ fuses to adjacent S_2_ near columella. Fossa deep, containing a spongy columella.

###### Distribution.

Regional: Southern margin of South Africa, extending from Agulhas towards Knysna; 333–463 m. Elsewhere: Philippines; Indonesia; South China Sea (Alcock 1902a; Cairns and Zibrowius 1997); New Zealand (Cairns 1995); Meteor Seamounts; Azores; Nicobar Islands (Zibrowius 1980; Cairns 1982); Bay of Bengal (von Marenzeller 1904a); Australia (Cairns 2004a); 371–805 m.

###### Remarks.

The examined specimens of *Enallopsammiapusilla* represent a new record for the region, the species differing from its congeners in having three complete cycles, corallites forming on all branch faces, and well-developed costae throughout the corallum (Zibrowius 1973).

##### Enallopsammia rostrata

Taxon classificationAnimaliaScleractiniaDendrophylliidae

(Pourtalès, 1878)

59A4D02B-75E8-551C-9349-FDF24693C20A

Fig. 9I, J

Amphihelia rostrata Pourtalès, 1878: 204, pl. 1, figs 4, 5. –Agassiz 1888: 152, fig. 473. –Gourret 1906: 122, pl. 12, fig. 11A, B.
Stereopsammia rostrata . –Pourtalès 1880: 97, 110–111.
Dendrophyllia (Coenopsammia) amphelioides Alcock, 1902a: 43–44, pl. 5.
Anisopsammia amphelioides . –Vaughan 1907: 156–157, pl. 47, figs 1, 2.
Coenopsammia amphelioides var. cucullata Vaughan, 1907: 157, pl. 48, figs 1–4.
Anisopsammia rostrata . –Gravier 1915: 3. –Gravier 1920: 102, pl. 12, figs 181–185.
Enallopsammia rostrata . –Squires 1959a: 40. –Laborel 1970: 156. –Zibrowius 1973: 44–45, pl. 2, figs 14–15. –Cairns 1978: 9. –Cairns 1979: 186–188, pl. 37, figs 2–3, 6. –Zibrowius 1980: 201–203, pl. 105, figs A–K, pl. 106, figs A–C. –Cairns 1982: 57, pl. 18, figs 1–4. –Cairns 1984: 27–28. –Zibrowius and Grygier 1985: 131, figs 48–50. –Zibrowius 1985: 314, 319, 322, 323. –Zibrowius and Gili 1990: 39–42, pl. 6, figs A–F, pl. 7, figs A–F. –Cairns 1991: 26, pl. 12, fig. B. –Dawson 1992: 45. –Cairns and Parker 1992: 52–53, pl. 18, figs E–I. –Cairns and Keller 1993: 281–282. –Cairns 1994: 92–93, pl. 39, figs D–F. –Cairns 1995: 127–128, pl. 44, figs C–F. –Cairns and Zibrowius 1997: 195. –Cairns 1999a: 134–135. –Cairns et al. 1999: 27. –Romano and Cairns 2000: 1049. –Cairns 2004a: 267, 316. –Le Goff–Vitry et al. 2004: 170, 176. –Cairns 2006: 48. –Kitahara 2007: 504, 505, 511, 513, 516, fig. 5G. –Pires 2007: 269. –Kitahara et al. 2010b. –Kitahara and Cairns 2021: 326–328, figs 173D–G, 174.
Enallopsammia amphelioides . –Zibrowius 1973: 45–46. –Cairns 1979: 187, pl. 40, figs 4, 5. –Zibrowius 1980: 203–204, pl. 106, figs D–I. –Grygier and Newman 1985: 6, fig. 2A–D.

###### Type locality.

Off the Straits of Florida (SSS ‘Blake’ stn. 2: 23°14'00"N, 82°25'00"W); 1472 m (Pourtalès 1878).

###### Type material.

Two syntypes are deposited at the MCZ (Cairns 1979; Zibrowius 1980).

###### Material examined.

SAMC_A073270 (1 specimen): Eastern margin, 39 km from Port St. Johns/13 km off Mkweni Estuary, 31°30'06.11"S, 29°55'12.00"E; 200 m. DSCS_INV 158 (1 specimen): Southern margin, 200 km from Gouritsmond/204 km off Goukou Estuary, 36°09'28.13"S, 21°59'53.81"E; 226–236 m. DSCS_INV 160 (1 specimen): Southern margin, 200 km from Gouritsmond/204 km off Goukou Estuary, 36°09'28.13"S, 21°59'53.81"E; 226–236 m. DSCS_INV 238 (1 specimen): Southern margin, 114 km from Knysna/off Bulolo Estuary, 35°06'11.27"S, 23°02'41.91"E; 333m. DSCS_INV 238 (1 specimen): 116 km from Knysna/off Goukamma Estuary, 35°07'11.34"S, 23°02'41.91"E; 333 m. DSCS_INV 224 (1 specimen): 116 km from Knysna/off Goukamma Estuary, 35°07'11.34"S, 23°02'41.91"E; 333 m. DEFF_SVMEC_INV 261 (1 specimen): Southern margin, 88 km from Oesterbaai/85 km off Tsitsikamma Estuary, 34°52'21.97"S, 24°12'51.01"E; 341–367 m.

###### Description.

Uniplanar colonies formed by extra-tentacular budding, and firmly attached to substrate by an encrusting base. Corallites confined to one face of corallum, arranged uniserially, and projecting ≤ 4.1 mm above branch. Corallites circular to slightly elliptical (GCD:LCD = 1.0–1.1); calicular margin serrate. Costae prominent at calicular margin, often forming a hood that partially covers the calice; becoming poorly-defined in direction to base, slightly convex, and covered with small granules. Intercostal striae deep, thin, and porous. Corallum white.

Septa hexamerally arranged in three complete cycles according to the formula: S_1_ > S_3_ > S_3_ (24 septa). S_1_ most exsert, narrow, and extending closer to columella deep in fossa with concave and slight serrate axial margins. S_2_ ¾ the width of S_1,_ and bear serrated axial margins. S_3_ slightly less wide than S_1–2,_ sometimes appearing rudimentary, and have dentate axial margins. S_3_ sometimes fusing to adjacent S_2_ near columella. Fossa deep, containing a rudimentary or trabecular columella.

###### Distribution.

Regional: Southern to eastern margin of South Africa, extending from Knysna towards Port St. Johns; 200–367 m. Elsewhere: Cosmopolitan, except for eastern Pacific and continental Antarctica; 110–2165 m.

###### Remarks.

The examined specimens extend the known distribution of *Enallopsammiarostrata* in the southwest Indian Ocean (Cairns and Keller 1993) further south from the Madagascar Plateau, thus representing a new record for South Africa. Species is easily distinguished by its uniplanar development with calices in only one side of the colony, (unlike the bushy and irregularly arranged calice of both *E.profunda* (Pourtalès, 1868) and *E.pusilla*), and costae being prominent at the calicular margin (instead of being prominent throughout corallum as in the case of both *E.profunda* and *E.pusilla*). Although previous specimens of *E.rostrata* have been reported to display septocostal rostrum (the enlargement of one CS_1_) (Cairns 1982; Cairns and Zibrowius 1997), this feature is absent in the South African examined representatives (all of which are small colonies).

##### Endopachys

Taxon classificationAnimaliaScleractiniaDendrophylliidae

Lonsdale, 1845

BDC7B9DD-A9DA-5A16-B81A-16B4C98603ED

###### Diagnosis.

Corallum solitary and free, resulting from transverse division or budding from corallum margin. Corallum straight. Shape of corallum variable, including cuneiform, compressed-cylindrical, and flabellate. Some species with six or twelve robust ridges or flanges aligned to C_1_ and C_2_. Epitheca absent. Base of corallum covered with spines. Towards calices, spines usually aligned into narrow costae. Pourtalès plan present. ≤ five cycles of septa. P_3_ or P_4_ usually present. Columella elongate, discrete, and spongy.

###### Type species.

*Endopachysalatum* Lonsdale, 1845, by subsequent designation (Wells 1975).

##### Endopachys grayi

Taxon classificationAnimaliaScleractiniaDendrophylliidae

Milne-Edwards & Haime, 1848

4F700005-0A61-522F-9217-E7875D312D89

Fig. 9K, L

Endopachys grayi Milne-Edwards & Haime, 1848b: 82–83, pl. 1, figs 2, 2A. –Semper 1872: 267. –van der Horst 1922: 68, 74. –van der Horst 1926: 51. –van der Horst 1927: 6–7, pl. 2, fig. 12. –Faustino 1927: 240–241. –Gardiner and Waugh 1939: 241. –Yabe and Eguchi 1942b: 139. –Squires 1961: 17. –Pillai 1972: 213. –Boshoff 1981: 42 (in part). –Cairns 1984: 27, pl. 5, fig. E. –Zibrowius and Grygier 1985: 137. –Veron 1986: 610. –Cairns 1989b: 34. –Cairns 1991a: 24–25, pl. 10, figs I–J, pl. 11, figs A, B. –Cairns and Keller 1993: 276. –Cairns 1994: 84–85, pl. 36, figs E, H pl. 37, fig. I. –Cairns 1995: 121–122, pl. 41, figs C–H. –Cairns and Zibrowius 1997: 185–186. –Cairns 1998: 362, 365. –Cairns 1999: 132, fig. 22F. –Cairns et al. 1999: 27. –Cairns 2001: 25, pl. 7, fig. G. –Cairns 2004a. 276, 316. –Cairns 2006: 49. –Kitahara and Cairns 2021: 329–330, 332, figs 173H–J, 175, 176A–B.
Endopachys weberi Alcock, 1902a: 109–110.
Endopachys oahense Vaughan, 1907: 147–148, pl. 44, fig. 3.
Endopachys japonicum Yabe & Eguchi, 1932e: 388, 399. –Yabe and Eguchi 1932b: 443. –Yabe and Eguchi 1932a: 14–17, pl. 2, figs 1–6. –Eguchi 1934a: 268. –Yabe and Eguchi 1942b: 139. –Eguchi 1965: 293. –Eguchi and Miyawaki 1975: 59.
Endopachys vaughani Durham, 1947: 39–40, pl. 11, figs 6–8, 10, 11.
Endopachys sp. –van der Horst 1922: 68, pl. 8,fig. 4.

###### Type locality.

Unknown (Cairns 1994; Cairns and Zibrowius 1997).

###### Type material.

Presumably lost (Cairns 1994).

###### Material examined.

ORI_EId1(2 specimens): Eastern margin, other locality data unknown. SAMC_A073064 (2 specimens): Eastern margin, 4 km from Cape Vidal/off Groot Brak Estuary, 28°07'30.00"S, 32°36'24.11"E; 75–80 m. SAMC_A073069 (1 specimen): Eastern margin, 48 km from Cape Vidal/21 km off Mgobezeleni Estuary, 27°42'53.99"S, 32°40'54.11"E; 160 m. SAMC_A073213 (1 specimen): Eastern margin, 29 km from Durban/14 km off Mbokodweni Estuary, 30°06'24.12"S, 31°00'47.88"E; 160–170 m. SAMC_A073266 (5 specimens): Southern margin, 2 km from Stilbaai/1 km off Goukou Estuary, 34°22'55.26"S, 21°25'25.49"E; 88 m. SAMC_A090114 (1 specimen): Eastern margin, 16 km from Port St. Johns/13 km off Bulolo Estuary, 31°45'00.00"S, 29°26'59.99"E; 70 m. SAMC_A090115 (9 specimens): Eastern margin, 29 km from Durban/22 km off Mdloti Estuary, 29°46'00.00"S, 31°16'59.99"E; 110–130 m. **SAM_H1420 (5 specimens)**: Eastern margin, 39 km from Mtunzini/8 km off Zinkwasi Estuary, 29°13'00.00"S, 31°30'00.00"E; 66–77 m. **SAM_H1427 (5 specimens)**: Eastern margin, 2 km from Durban/8 km off Umgeni Estuary, 29°52'00.00"S, 31°00'00.00"E; 99 m. SAM_H1476 (3 specimens): Eastern margin2 km from Durban/8 km off Umgeni Estuary, 29°52'00.00"S, 31°00'00.00"E; 99 m. SAM_H3120 (28 specimens): Eastern margin, 39 km from Mtunzini/8 km off Zinkwasi Estuary, 29°13'00.00"S, 31°30'00.00"E; 73 m. SAM_H3121 (2 specimens): Eastern margin, 39 km from Mtunzini/8 km off Zinkwasi Estuary, 29°13'00.00"S, 31°30'00.00"E; 73 m. **SAM_H3122 (3 specimens)**: Eastern margin, 6 km from Durban/9 km off Umgeni Estuary, 29°52'59.99"S, 31°03'05.00"E; 86 m. SAM_H3123 (2 specimens): Eastern margin, 9 km from Shaka’s Rock/2 km off Tongati Estuary, 29°34'00.00"S, 31°10'59.99"E; 66 m. **SAM_H4593 (2 specimens)**: Eastern margin, 26 km from Port St. Johns/off Bulolo Estuary, 29°34'47.99"S, 31°41'59.99"E; 138 m. DEFF_NANSEN–INV 32 (8 specimens): Eastern margin, 30 km from Shaka’s Rock/31 km off Tongati Estuary, 29°43'11.99"S, 31°25'47.99"E; 185 m. **USNM 91812 (3 specimens)**: Eastern margin, 29 km from Shaka’s Rock/24 km off Mdloti Estuary, 29°45'54.00"S, 31°18'11.88"E; 105 m. **USNM 91813 (1 specimen)**: Eastern margin, 44 km south of Ponta Do Ouro/28 km off Kosi Bay Estuary, 27°14'35.88"S, 32°48'47.87"E; 74 m.

###### Imagery data.

PF 10983 (4 specimens): Eastern margin, 19 km from Shaka’s Rock/3 km off Mdloti Estuary, 29°38'59.99"S, 31°07'59.99"E; 71–73 m.

###### Description.

Corallum (anthocyathus) variable in shape, including cuneiform, compressed-cylindrical, and flabellate. Corallum free, compressed and usually with a rounded base on GCD plane. Largest specimen examined (DEFF_NANSEN–INV 32) 37.2 × 19.7 mm in CD (excluding crests), and 25.8 mm in H. Thecal edges project outward to form slightly porous, and straight to slightly sinuous thecal crests. Upper thecal crest meets in acute angle, and often support one bud obliquely oriented (sometimes supporting ≤ four buds). Crest wider at lower half of corallum. Costae equal in width and flat to slightly convex, becoming progressively less developed towards base. Intercostal striae thin, narrow, and porous. Corallum white.

Septa hexamerally arranged in five complete cycles according to the formula: S_1–2_ > S_3_ > S_5_ > S_4_ (96 septa). S_1–2_ thick, most exsert, porous at upper distal margin, and extend towards columella with straight, vertical, and finely dentate axial margins. S_3_ less exsert and extend ¾ the distance of S_1–2_ with dentate axial margins. S_4_ variable in development: if not flanked by a pair of S_5_, both S_4_ in a half-system bend towards each other meeting before S_3_ in a characteristic Pourtalès plan, but, if flanked, S_4_ have highly laciniate axial margins. S_5_ dimorphic in development: those adjacent to S_1_ more exsert and wider than those adjacent to S_2_. Each Pourtalès plan terminates in a palus-like structure. All septal faces granular. Fossa deep, containing septum in the elongate and spongy columella.

###### Distribution.

Regional: Eastern margin of South Africa, extending from off Port St. Johns towards Kosi Bay Estuary (44 km south of Ponta Do Ouro: Mozambique); 66–170 m. Elsewhere: Mozambique; Tanzania (Cairns 1989b); Zanzibar; Mauritius (van der Horst 1926); Saya de Malha; Arabian Sea (Gardiner and Waugh 1939) Cairns and Keller 1993); Philippines and Indonesia (Cairns and Zibrowius 1997; Kitahara and Cairns 2021), Malaysia; Australia (Cairns 2004); New Caledonia (Zibrowius and Grygier 1985); Wallis and Futuna; Vanuatu (Cairns 1999a); Hawaii; United States; Japan (Cairns 1984; Zibrowius and Grygier 1985; Cairns 1994); 37–550 m.

###### Remarks.

*Endopachysgrayi* is easily recognised by its corallum shape and presence of lateral thecal crest. Overall, *Tropidocyathuslessoni* (Michelin, 1842) resembles *E.grayi*, but belongs to a different family (Turbinoliidae) and is easily distinguished by having a solid corallum (not porous as dendrophylliids). Within the genus, only two species, *E.grayi* and *Endopachysbulbosa* Cairns & Zibrowius, 1997, are known and they differ in: (i) distribution (*E.grayi* = Indo-Pacific vs. *E.bulbosa* = South Pacific), (ii) basal thickness (*E.grayi* = 3 mm vs. *E.bulbosa* = 4 mm), (iii) thecal face angle in relation to height (*E.grayi* = face angle being low initially and broadens with height vs. *E.bulbosa* = face angle being high initially and decreases with height), (iv) septal exsertness (*E.grayi* = S_1–2_ being ≤ 3.0 mm vs. *E.bulbosa* = S_1–2_ being ≤ 5.0 mm), (v) costae (*E.grayi* = poorly-defined vs. *E.bulbosa* = well-defined costae), and (vi) presence or absence of pali (*E.grayi* = pali present vs. *E.bulbosa* = pali absent) (Cairns and Zibrowius 1997). The DEFF_NANSEN–INV 32 specimen appears to be the largest *E.grayi* recorded to date. It is 37.2 × 19.7 mm in CD (excluding crests), 25.8 mm in H, and has a pair of S_6,_ totalling 98 septa.

##### Endopsammia

Taxon classificationAnimaliaScleractiniaDendrophylliidae

Milne-Edwards & Haime, 1848

7FA833E6-452D-565D-8B1A-F004C0B57A06

###### Diagnosis.

Corallum solitary, conical to subcylindrical, and firmly attached. Epitheca thin, covering most of the synapticulotheca. Underlying epitheca weakly costate, covered with low granules. Septa arranged in normal insertion pattern in ≤ four cycles. Axial margins of all septa coarsely dentate to laciniate. Columella spongy, non-discrete. Tabular endothecal dissepiments present in elongate coralla.

###### Type species.

*Endopsammiaphilippensis* Milne-Edwards & Haime, 1848, by monotypy.

##### Endopsammia philippensis

Taxon classificationAnimaliaScleractiniaDendrophylliidae

Milne-Edwards & Haime, 1848

AB4FEA8B-8121-5D17-A29F-A55A65F6FA78

Fig. 9M, N

Endopsammia philippensis Milne-Edwards & Haime, 1848b: 91, pl. 1, figs 5, 5A. –Faustino 1927: 243–244, pl. 77, figs 5, 6. –Pillai and Scheer 1976: 71–72. –Cairns and Zibrowius 1997: 188, fig. 28C–E. –Cairns 2001: 23, pls 5H, I . –Cairns 2004a: 316.
Balanophyllia regularis . –van der Horst 1922: 63. –van der Horst 1926: 50, pl. 3, figs 10, 11.
Endopsammia philippinensis . –Wells 1964: 118, pl. 2, figs 12, 13. –Cairns 1991: 26. –Cairns and Keller 1993: 221.
Certotrochus brunneus . –Boshoff 1981: 36.

###### Type locality.

Philippines, depth unknown (Milne-Edwards and Haime 1848b).

###### Type material.

The holotype is deposited at the NHMUK or MNHN (Cairns and Zibrowius 1997; Cairns 2001, 2004a).

###### Material examined.

ORI_DIIIb1 (2 specimens): Eastern margin, 33 km from Durban/31 km off Beachwood Mangroves, 29°55'00.00"S, 31°19'59.99"E; 442 m.

###### Imagery data.

SAM_H1576 (1 specimen): Eastern margin, 35 km from Port Edward/10 km off Mtentu Estuary, 31°18'00.00"S, 29°58'00.00"E; depth unknown.

###### Description.

Corallum conical to sub-cylindrical, relatively small, attached to substrate by a thin encrusting base. Calice circular and calicular margin lanceted. Largest specimen examined (ORI_DIIIb) 7.80 × 7.70 mm in CD, 8.20 mm in H. Epitheca thin and extending ¾ of lower corallum. Theca near calicular margin porous and non-costate. Corallum white.

Septa hexamerally arranged in four cycles, last cycle being incomplete, according to the formula: S_1_ ≥ S_2_ > S_3_ > S_4_ (≤ 30 septa). S_1_ slightly exsert and reaches columella. Higher cycle septa (S_2–4_) progressively less exsert (if at all). S_2_ equal or ¾ the width of S_1._ S_3_ being ^1^/_4_ the width of S_2_, sometimes rudimentary in some half-systems without S_4._ S_4,_ if present, rudimentary. All septa bear dentate to slightly laciniate axial margins. Septa faces bearing spines. Fossa shallow, containing a spongy or rudimentary columella.

###### Distribution.

Regional: Eastern margin of South Africa, from north of Port Edward extending towards Durban; 442 m. Elsewhere: Philippines (Milne-Edwards and Haime 1848b); Indonesia (Cairns and Zibrowius 1997); Indian Ocean islands (Maldives, Zanzibar, Seychelles, Chagos) (van der Horst 1926; Pillai and Scheer 1976); tropical Pacific Ocean; Loyalty Islands; Queensland; Papua New Guinea (Wells 1964; Cairns and Zibrowius 1997; Cairns 2004a); 0–73 m.

###### Remarks.

Among congeners (*Endopsammiaregularis* (Gardiner, 1899) and *E.pourtalesi* (Durham & Barnard, 1952)), *E.philippensis* closely resembles *E.regularis* in having septa hexamerally arranged in four incomplete cycles, in corallum being solitary (relatively squat), and bearing an epitheca that extends ¾ of the corallum height. However, they differ as *E.regularis* has exsert septa. Although *E.pourtalesi* also has septa arranged hexamerally in four incomplete cycles, this species is distinguished from *E.philippensis* by its quasi-colonial coralla with slender corallites, presence of endothecal dissepiments, and bearing non-exsert septa (Cairns 1991). *Endopsammiaphilippensis* has previously been documented in the southwest Indian Ocean (van der Horst 1922, 1026; Cairns and Keller 1993), therefore the examined Boshoff (1981) material (ORI_DIIIb1) represents a species range extension further south of Zanzibar, and a new record for South Africa.

##### Heteropsammia

Taxon classificationAnimaliaScleractiniaDendrophylliidae

Milne-Edwards & Haime, 1848

7E05EC24-3197-5C9A-9373-BCA35DCD2D34

###### Diagnosis.

Corallum solitary or colonial, latter condition achieved by intratentacular budding and resulting in ≤ 40 contiguous corallites. Adult corallum free and mobile, globular in shape. Coralla usually attached to small gastropod shells, these subsequently overgrown. Each specimen apparently in obligate symbiosis with a sipunculid worm, which lives in base of corallum. Epitheca absent. Synapticulotheca covered with finely serrate ridges, usually one to three ridges per corresponding septum (not considered to be conventional costae). Pourtalès plan present. Paliform lobes may be present. Columella spongy, not discrete. Endotheca absent.

###### Type species.

*Heteropsammiamichelinii* Milne-Edwards & Haime, 1848, by monotypy.

##### Heteropsammia cochlea

Taxon classificationAnimaliaScleractiniaDendrophylliidae

(Spengler, 1781)

06C1DFB8-DFE1-5435-8F2B-CAD4660CD1AB

Figs 9O, P , 10A, B

Madrepora cochlea Spengler, 1781: 240–248, figs A–D.
Psammoseris cylicioides Tenison-Woods, 1879: 10–11. –Tenison-Woods 1880: 297–299.
Heteropsammia michelini . –Kent 1893: 106, 177. –Wells 1964: 108, 120.
Heteropsammia cochlea . –van der Horst 1922: 66–67. –van der Horst 1926: 51. –Veron and Pichon 1980: 416–420, figs 727, 729. –Zibrowius and Grygier 1985: 129, figs 43–44. –Veron 1986: 576–577. –Hoeksema and Best 1991: 234–237, figs 24–28 (in part). –Cairns 1998: 406–408. –Cairns 1999a: 132–133. –Cairns et al. 1999: 27. –Veron 2000: 407. –Cairns 2001: 19–20, pl. 2, figs H–J, pl. 3, figs A–E –Cairns 2004a: 316. –Kitahara and Cairns 2021: 334, 336, figs 176C,178A–C.
Heterocyathus aequicostatus . –Boshoff 1981: 37.
Heteropsammia aphrodes . –Boshoff 1981: 42.
Heteropsammia cochleata . –Cairns 2009: 25.

###### Type locality.

Off Tranquebar, southeastern India, depth unknown (Spengler 1781).

###### Type material.

The type specimen is presumably lost (Cairns 2004a).

###### Material examined.

ORI_DIIIe1_3 (1 specimen), ORI_EIe1 (10 specimens): Eastern margin, locality data unknown. SAMC_A073006 (1 specimen): Locality data unknown; 17 m. SAMC_A073051 (6 specimens): Eastern margin, , 41 km south of Ponta Do Ouro/26 km off Kosi Bay Estuary, 27°13'00.11"S, 32°49'41.87"E; 72 m. SAMC_A073061 (8 specimens): Eastern margin, 25 km from Cape Vidal/23 km off St Lucia Estuary, 27°54'42.11"S, 32°36'42.11"E; 42–50 m. SAMC_A073065 (1 specimen): Eastern margin, 67 km from CAPE VIDA/6 km off Mgobezeleni Estuary, 27°32'48.12"S, 32°42'00.00"E; 50 m. SAMC_A073072 (3 specimens): Eastern margin, 36 km from Cape Vidal/33 km Mgobezeleni Estuary, 27°48'54.00"S, 32°38'24.00"E; 52 m. SAMC_A073084 (3 specimens): Locality data unknown. SAMC_A073086 (1 specimen): Eastern margin, 329 km from Port Edward/330 km off Mtentu Estuary, 32°55'18.12"S, 32°55'18.12"E; 49 m. SAMC_A073089 (1 specimen): Eastern margin, 67 km south of Ponta Do Ouro/14 km off Mgobezeleni Estuary, 27°26'12.11"S, 32°44'12.11"E; 55–60 m. SAMC_A073090 (2 specimens): Eastern margin, 69 km from Cape Vidal/5 km off Mgobezeleni Estuary, 27°31'36.12"S, 32°41'48.11"E; 40 m. SAMC_A073091 (2 specimens), SAMC_A073093 (1 specimen): Locality data unknown. SAMC_A073095 (3 specimens): Eastern margin, 66 km south of Ponta Do Ouro/15 km off Mgobezeleni Estuary, 27°25'59.87"S, 32°44'30.12"E; 55–100 m. SAMC_A073098 (1 specimen): Locality data unknown. SAMC_A073100 (5 specimens): Eastern margin, 42 km south of Ponta Do Ouro/27 km off Kosi Bay Estuary, 27°13'30.00"S, 32°49'30.00"E; 78 m. SAMC_A073112 (2 specimens): Eastern margin, 35 km from Cape Vidal/32 km off St Lucia Estuary, 27°49'41.87"S, 32°38'12.11"E; 54 m. SAMC_A073115 (5 specimens): Eastern margin, 35 km from Cape Vidal/32 km off St Lucia Estuary, 27°49'41.87"S, 32°38'12.11"E; 47–50 m. SAMC_A073118 (12 specimens): Eastern margin, 25 km off Cape Vidal/23 km off St Lucia Estuary, 27°54'42.11"S, 32°36'42.11"E; 42–50 m; SAMC_A073124 (1 specimen): Eastern margin, 66 km south of Ponta Do Ouro/15 km off Mgobezeleni Estuary, 27°25'54.11"S, 32°44'17.88"E; 46–66 m. SAMC_A073142 (1 specimen): Eastern margin, 19 km south of Ponta Do Ouro/12 km off Kosi-Kumpungwini (Sifungwe) Estuary, 27°01'05.87"S, 32°55'12.00"E; 78 m. SAMC_A073143 (1 specimen): Eastern margin, 38 km south of Ponta Do Ouro/24 km off Kosi Bay Estuary, 27°11'24.00"S, 32°51'00.00"E; 100 m. SAMC_A073156 (1 specimen): Eastern margin, 39 km from Cape Vidal/29 km off Mgobezeleni Estuary, 27°47'23.99"S, 32°38'53.87"E; 65–70 m. SAMC_A073170 (31 specimen): Eastern margin, 39 km from Cape Vidal/29 km off Mgobezeleni Estuary, 27°47'23.99"S, 32°38'53.87"E; 65–70 m. SAMC_A073192 (1 specimen): Eastern margin, 69 km from Cape Vidal/7 km off Mgobezeleni Estuary, 27°31'48.00"S, 32°42'47.99"E; 70 m. SAMC_A073193 (1 specimen): Eastern margin, 67 km from Cape Vidal/6 km off Mgobezeleni Estuary, 27°32'30.11"S, 32°42'00.00"E; 48–58 m. SAMC_A073205 (3 specimens): Eastern margin, 65 km from Cape Vidal/7 km off Mgobezeleni Estuary, 27°33'47.88"S, 32°42'15.12"E; 64 m. SAMC_A073214 (30 specimens): Eastern margin, 37 km from Cape Vidal/32 km off Mgobezeleni Estuary, 27°48'47.88"S, 32°38'53.87"E; 50 m. SAMC_A073218 (1 specimen): Eastern margin, 42 km south of Ponta Do Ouro/27 km off Kosi Bay Estuary, 27°13'30.00"S, 32°49'30.00"E; 74 m. SAMC_A090117 (1 specimen): Eastern margin, 66 km from Cape Vidal/7 km off Mgobezeleni Estuary, 27°33'11.88"S, 32°42'47.87"E; 85 m. SAMC_A090118 (2 specimens): Eastern margin, 59 km from Cape Vidal/10 km off Mgobezeleni Estuary, 27°37'00.00"S, 32°40'54.00"E; depth unknown. SAM_H814 (21 specimen): Eastern margin, 19 km from Shaka’s Rock/3 km off Mdloti Estuary, 29°38'59.99"S, 31°07'59.99"E; depth unknown. USNM 90242 (1 specimen, sub-sample of SAMC_A073086): Eastern margin, 329 km from Port Edward/330 km off Mtentu Estuary, 32°55'18.12"S, 32°55'18.12"E; 49 m. USNM 90426 (1 specimen): Eastern margin, , 41 km south of Ponta Do Ouro/26 km off Kosi Bay Estuary, 27°13'00.11"S, 32°49'41.87"E; 72 m. USNM 90434 (5 specimens, sub-samples of SAMC_A073205): Eastern margin, 65 km from Cape Vidal/7 km off Mgobezeleni Estuary, 27°33'47.88"S, 32°42'15.12"E; 64 m. USNM 90435 (1 specimen): Eastern margin, 67 km from Cape Vidal/6 km off Mgobezeleni Estuary, 27°32'42.00"S, 32°42'29.88"E; 63 m. USNM 90439 (11 specimen): Eastern margin, 64 km from Cape Vidal/7 km off Mgobezeleni Estuary, 27°34'12.00"S, 32°42'06.12"E; 62–64 m.

###### Description.

Corallum solitary, encapsulating a gastropod shell, with one large efferent pore projecting downward from base of corallum, and several smaller pores on lower theca. Calice elliptical (GCD:LCD = 1.3–1.5); calicular margin lanceted. Largest specimen examined (SAMC_A073170) 11.4 × 9.1 mm in CD, and 13.1 mm in H. Upper theca highly porous and usually composed of discontinuous interconnected granular rows. Lower theca granular. Corallum white.

Septa hexamerally arranged in four complete cycles according to the formula: S_1–2_ > S_4_ > S_3_ (48 septa). S_1–2_ highly exsert, equally wide, with straight axial margins that fuse to columellar elements. S_3_ least exsert septa and bear serrate axial margins. S_4_ fuse to neighbouring septa near calicular margin forming well-developed and porous triangular lancets. In each half-system, a pair of S_4_ fuses before flanked S_3_ and reach columella as one septum. S_4_ upper axial margins highly concave becoming almost vertical after fusing to adjacent S_4_ near columella. Fossa of moderate depth, containing a spongy columella.

###### Distribution.

Regional: Eastern margin of South Africa, extending from off Port Edward towards Kosi-Kumpungwini (Sifungwe) Estuary (19 km south of Ponta Do Ouro: Mozambique); 17–100 m. Elsewhere: Widespread throughout the tropical Indo-Pacific (Veron and Pichon 1980; Hoeksema and Best 1991; Cairns 1999a); 6–622 m.

###### Remarks.

*Heteropsammiacochlea* is a symbiotic facultative species, known to be zooxanthellate or azooxanthellate in shallow waters and those found in deeper waters being azooxanthellate (Hoeksema and Mathews 2015). The variation in corallum type has resulted in a long standing discussion regarding the number of species represented within *Heteropsammia*. As such, two valid species are recogniszed (*H.cochlea* and *H.eupsammides* (Gray, 1849)) on the basis of the number of calices (monostomous or polystomous) (Hoeksema and Best 1991; Hoeksema 1993). Whilst Cairns (2001) on the other hand, cautiously acknowledges three (*H.cochlea*, *H.eupsammides* (Gray, 1849), and *H.moretonensis* Wells, 1964) in his generic revision of dendrophylliids. Irrespective of this, two species are represented in South Africa, and the differences between the two are elaborated in the account of *H.eupsammides* below. Zibrowius and Grygier (1985) have reported *H.cochlea* from the Great Barrier Reef and Somalia to host endoparasites of the ascothoracid crustacean *Petrarcaokadai*, an observation not evident in the South African specimens examined. Additionally, specimens examined include zooxanthellate representatives (> 40 m) (Hoeksema and Best 2015) and should therefore not be considered in biodiversity assessments focusing on azooxanthellate forms.

##### Heteropsammia eupsammides

Taxon classificationAnimaliaScleractiniaDendrophylliidae

(Gray, 1849)

BDBE7BE9-9945-5526-AB49-CE5329ED5273

Fig. 10C, D.

Heterocyathus eupsammides Gray, 1849: 77, pl. 2, figs 5–7.
Heteropsammia geminata Verrill, 1870: 370–371, fig. 1. –van der Horst 1922: 67. –Faustino 1927: 239–240, pl. 76, figs 11–13.
Heteropsammia multilobata Moseley, 1881: 196–197, pl. 12, figs 1–3.
Heteropsammia michelini . –van der Horst 1926: 51, pl. 3, figs 14–20. –Gardiner and Waugh 1938: 241.
Heteropsammia cochlea . –Veron and Pichon 1980: 416–420.
Heteropsammia eupsammides . –Hoeksema and Best 1991: 237–240. –Kitahara and Cairns 2021: 336–338, figs 178D–I, 179.

###### Type locality.

China Sea (Verrill 1870).

###### Type material.

Unknown.

###### Material examined.

SAMC_A090129 (1 specimen): Eastern margin, 69 km from Cape Vidal/5 km off Mgobezeleni Estuary, 27°31'36.12"S, 32°41'48.11"E; 40 m.

###### Imagery data.

ZMK (in part: 2 specimens): Locality data unknown.

###### Description.

Corallum solitary, encapsulating a gastropod shell, with one large efferent pore projecting downward from base of corallum, and several smaller efferent pores on lower theca. Calice irregular to elliptical (GCD:LCD = 1.5), calicular margin lanceted. Only specimen examined (SAMC_A090129) 12.9 × 8.7 mm in CD and 8.9 mm in H. Upper theca highly porous and usually composed of discontinuous but interconnected granular rows. Lower theca becoming more granular. Corallum white.

Septa hexamerally arranged in six incomplete cycles according to the formula: S_1–2_ > S_3_ > S_5_ > S_4_ (107 septa). S_1–2_ highly exsert, equally wide, with lower axial margins fusing to columellar elements. Higher cycle septa (S_1–2_) progressively less exsert. S_3_^1^/_2_ the width of S_1–2_. S_4_ dimorphic in development: those in half-systems lacking S_6_ ~ ^1^/_5_ the width of S_3;_ but those in half-systems with S_6_^1^/_3_ the width S_3._ S_5_ also dimorphic, being double the width of S_4_ in half-systems lacking S_6_ and merging in front of S_4_ before meeting columella as one septum. However, in half-systems with S_6,_ those S_5_ neighbouring S_3_ are double the width of S_4,_ but those flanked by S_6_ are rudimentary. S_6_^1^/_3_ the width of S_4_ and fuse before flanked S_5_, reaching columella as one septum. All septa with straight and slightly serrate axial margins. Fossa of moderate depth, containing a spongy columella.

###### Distribution.

Regional: Eastern margins of South Africa, off Cape Vidal; 40 m. Elsewhere: China Sea (Verrill 1870); Indonesia (van der Horst 1922; Hoeksema and Best 1991); Philippines (Moseley 1881); New Caledonia (Kitahara and Cairns 2021); Australia (Veron and Pichon 1980); Maldives; Seychelles (van der Horst 1926); and Zanzibar (van der Horst 1926; Gardiner and Waugh 1938); 38–281 m.

###### Remarks.

*Heteropsammiaeupsammides* is one of the apozooxanthellate species, like *H.cochlea*, it exhibits a symbiotic relationship with sipunculan worms. These two species may be distinguished from one another by whether they are monostomous or polystomous (Cairns 2001: *Heteropsammiaeupsammides* is polystomous [i.e., has > 2 calices formed by intra-stomodeal budding] (Hoeksema and Best 1991). The examined specimen of *H.eupsammides* appears to be in its early stages of calyx separation and therefore can be mistaken with *H.cochlea*. However, can be distinguished by the number of cycles (six incomplete cycles in *H.eupsammides* as compared with four complete ones in *H.cochlea*) and a higher number of septa (*H.eupsammides* :107 vs. *H.cochlea* :48), which is a resuly of varying stomata (smaller in *H.eupsammides*). Apart from the number of septa cycles, the septa profile varies between the two species, whereby *H.eupsammides* bears S_3_ > S_5_ > S_4_, with straight to slightly serrated axial margins, and lacking porous triangular lancets (as compared with *H.cochlea* which has S_4_ > S_3_, with consistent straight axial margins, and bearing triangular lancets). This record represents a range southern range extension from Zanzibar.

**Figure 10.**
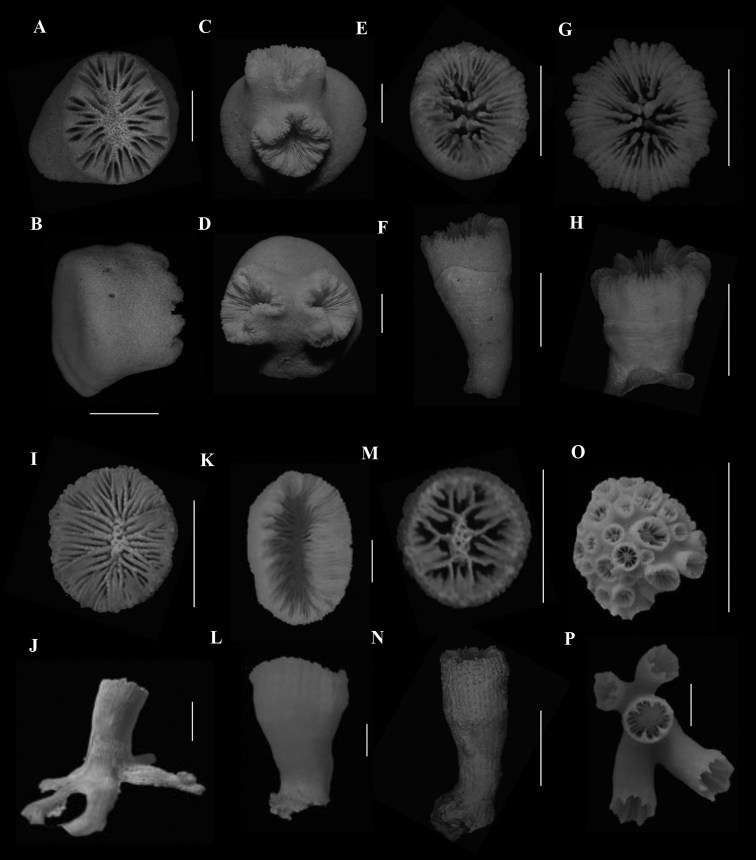
**A**, **B***Heteropsammiacochlea* (ORI_EIe1, locality data unknown) **A** calicular view **B** lateral view **C**, **D***Heteropsammiaeupsammides* (ZMK, locality data unknown) **C** calicular view **D** calicular view **E–H***Pourtalopsammiatogata***E**, **F** (SAM_H2829, off East London, 146–238 m) **E** calicular view **F** lateral view **G**, **H** (SAM_H2831, off Cintsa, 159 m) **G** calicular view **H** lateral view **I**, **J***Rhizopsammiaannae* (SAM_H1497, off the Agulhas, depth unknown) **I** calicular view **J** lateral view **K**, **L***Rhizopsammiacompacta* (SAMC_A073041, off Kosi Bay Estuary, 69 m) **K** calicular view **L** lateral view **M**, **N***Rhizopsammiaverrilli* (SAM_H1502, off Richards Bay, 165 m) **M** calicular view **N** lateral view **O***Tubastraeacoccinea* (ORI_EIb4, Isipingo; depth unknown) **P**Tubastraea sp. cf. diaphana (SAM_H5103, Cape Vidal, 59 m): Calicular view. Scale bars: 10 mm.

##### Pourtalopsammia

Taxon classificationAnimaliaScleractiniaDendrophylliidae

Cairns, 2001

93234958-35E2-5A4E-85D6-03BD1BB78E76

###### Diagnosis.

Corallum solitary, conical to subcylindrical (sometimes scolecoid), and attached. Epitheca well developed, covering basal synapticulotheca; coenosteum distal to epitheca covered with thin, hispid ridges. Septa arranged in normal insertion pattern (not Pourtalès plan); three or four cycles of septa; axial margins of S_1_ highly sinuous. Columella absent. Endothecal dissepiments absent.

###### Type species.

*Balanophylliatogata* van der Horst, 1927, by monotypy.

##### Pourtalopsammia togata

Taxon classificationAnimaliaScleractiniaDendrophylliidae

(van der Horst, 1927)

9EC0AF8B-4B28-5E99-8C00-B7518CC423CE

Fig. 10E–H

Balanophyllia togata van der Horst, 1927: 5–6.
Thecopsammia togata . –Wells 1935: 531.
Trochopsammia togata . –Cairns and Keller 1993: 275–276. –Cairns 2000: 22, pl. 4, figs F–I, pl. 5, fig. A.
Pourtalopsammia togata . –Cairns 2001: 22.

###### Type locality.

Off Buffalo River mouth, South Africa; 567 m (van der Horst 1927).

###### Type material.

The type material is presumably at the BMNH (Cairns 2001).

###### Material examined.

DEFF_AI2–INV 135 (1 specimen): Eastern margin, 37 km from Cintsa/21 km off Cwili Estuary, 32°49'59.99"S, 28°30'00.00"E; 228 m. DSCS–INV 124 (1 specimen): Southern margin, 192 km from Agulhas/198 km off De Mond-Heuningnes Estuary, 36°15'34.13"S, 21°11'46.61"E; 513 m. DSCS–INV 364 (1 specimen): Southern margin, 68 km from Cape St. Francis/70 km off Slang Estuary, 34°47'35.77"S, 24°38'35.69"E; 520 m. DSCS–INV 424 (1 specimen): Southern margin, 65 km from Cape St. Francis/78 km off Slang Estuary, 34°43'40.13"S, 25°08'53.47"E; 622 m. DSCS–INV 425 (1 specimen): Southern margin, 65 km from Cape St. Francis/78 km off Slang Estuary, 34°43'40.13"S, 25°08'53.47"E; 622 m. DSCS–INV 477 (2 specimens): Southern margin, 58 km from Port Alfred/35 km off Mgwalana Estuary, 33°39'10.19"S, 27°29'57.58"E; 304 m. DSCS–INV 516 (17 specimens): Southern margin, 58 km from Port Alfred/35 km off Mgwalana Estuary, 33°39'10.19"S, 27°29'57.58"E; 304 m. DSCS–INV 569 (8 specimens): Southern margin, 65 km from Cape St. Francis/70 km off Slang Estuary, 34°47'05.01"S, 24°45'42.30"E; 304 m. SAMC_A073015 (3 specimens): Southern margin, 32 km from Mazeppa Bay/19 km MenduEstuary, 32°25'00.11"S, 28°58'18.11"E; 330–340 m. SAMC_A073042 (1 specimen): Eastern margin, 53 km from Shaka’s Rock/46 km off Zinkwasi Estuary, 29°32'53.88"S, 31°47'12.11"E; 200 m. SAMC_A073105 (3 specimens): Eastern margin, 36 km from Cape Vidal/32 km off Mgobezeleni Estuary, 27°48'54.00"S, 32°38'24.00"E; 52 m. SAMC_A073157 (2 specimens): Eastern margin, 10 km from Port Edward/24 km off Bilanhlolo Estuary, 31°05'48.11"S, 30°18'47.88"E; 140 m. SAMC_A073158 (2 specimens): Eastern margin, 10 km from Port Edward/24 km off Bilanhlolo Estuary, 31°05'48.11"S, 30°18'47.88"E; 140 m. SAMC_A073159 (2 specimens): Southern margin, 37 km from Mazeppa Bay/15 km off Mendu Estuary, 32°20'35.87"S, 29°00'11.87"E; 100 m. SAMC_A073172 (1 specimen): Eastern margin, 36 km from Coffee Bay/20 km off Ntlonyane Estuary, 32°18'11.88"S, 29°06'11.88"E; 550 m. SAMC_A073180 (5 specimens): Southern margin, 33 km from Mazeppa Bay/24 km off Cwili Estuary, 32°45'47.88"S, 28°36'24.12"E; 240–250 m. SAMC_A073269 (1 specimen): Southern margin, 40 km from Port St. Johns/13 km off Mkweni Estuary, 31°30'09.00"S, 29°55'47.99"E; 300 m. SAMC_A090113 (1 specimen): Southern margin, 98 km from Gansbaai/103 km off Buffels Oos Estuary, 35°15'18.00"S, 18°39'18.00"E; 547 m. SAMC_A090152 (4 specimens): 116 km from Knysna/off Goukamma Estuary, 35°07'11.34"S, 23°02'41.91"E; 333 m. SAM_H1379 (2 specimens): Southern margin, 3 km from East London/1 km off Buffalo Estuary, 33°01'29.99"S, 27°55'00.00"E; 549 m. SAM_H1687 (1 specimen): Eastern margin, 17 km from St. Lucia Estuary/16 km off Mfolozi Estuary, 28°21'53.99"S, 32°34'36.00"E; 775–825 m. SAM_H2827 (2 specimens): Eastern margin, 14 km from Cape Vidal/21 km off St Lucia Estuary, 28°04'00.00"S, 32°40'47.99"E; 550 m. SAM_H2829 (1 specimen): Southern margin, 11 km from East London/5 km off Gouda Estuary, 33°05'03.24"S, 27°49'33.40"E; 146–238 m. SAM_H2830 (2 specimens): Eastern margin, 14 km from Mazeppa Bay/20 km off Great Kei Estuary, 32°34'00.00"S, 28°33'00.00"E; 174 m. SAM_H2831 (14 specimens): Southern margin, 28 km from CINSTA/3 km off Morgan Estuary, 32°42'31.81"S, 28°21'54.38"E; 159 m. SAM_H2842 (9 specimens): Eastern margin, 18 km from Cape Vidal/27 km off Mfolozi Estuary, 28°16'18.00"S, 32°38'48.00"E; 670 m. SAM_H3032 (1 specimen): Eastern margin, 36 km from Port Shepstone/29 km off Mhlabatshane Estuary, 30°43'11.99"S, 30°48'47.99"E; 780 m. SAM_H3033 (4 specimens): Eastern margin, 19 km from Margate/off Bilanhlolo Estuary, 30°56'59.99"S, 30°31'41.99"E; 850 m. SAM_H3109 (1 specimen): Eastern margin, 23 km from St. Lucia Estuary/21 km off Mfolozi Estuary, 28°31'41.99"S, 32°34'00.00"E; 680 m. SAM_H3135 (6 specimens): Southern margin, 40 km from Cintsa/29 km off Cwili Estuary, 32°55'00.00"S, 28°31'00.00"E; 630 m. SAM_H3136 (18 specimens): Eastern margin, 28 km from Coffee Bay/19 km off Bulungulu Estuary, 32°14'53.99"S, 29°10'23.99"E; 620–560 m. SAM_H3137 (9 specimens): Southern margin, 32 km off Mazeppa Bay/24 km off Kobole Estuary, 32°28'36.00"S, 28°58'48.00"E; 710–775 m. SAM_H3138 (11 specimen): Eastern margin, 30 km from Scottburgh/20 km off Fafa Estuary, 30°33'24.00"S, 30°48'35.99"E; 690 m. SAM_H3139 (2 specimens): Southern margin, 29 km from Mazeppa Bay/25 km off Kobole Estuary, 32°29'30.00"S, 28°57'06.00"E; 650–700 m. SAM_H3157 (2 specimens): Eastern margin, 30 km from Coffee Bay/21 km off Bulungulu Estuary, 32°15'24.00"S, 29°09'42.00"E; 600–650 m. SAM_H3209 (1 specimen): Eastern margin, 14 km from Cape Vidal/21 km off St Lucia Estuary, 28°04'00.00"S, 32°40'47.99"E; 550 m. SAM_H4244 (2 specimens): Eastern margin, 48 km south of Ponta Do Ouro/32 km off Kosi Bay Estuary, 27°16'48.00"S, 32°49'23.99"E; 400 m. SAM_H4245 (5 specimens): Eastern margin, 18 km south of Ponta Do Ouro/20 km off Kosi-Kumpungwini (Sifungwe) Estuary, 26°55'30.00"S, 33°02'48.12"E; 500 m. SAM_H4246 (4 specimens): Eastern margin, 15 km south of Ponta Do Ouro/17 km off Kosi-Kumpungwini (Sifungwe) Estuary, 26°55'30.00"S, 33°01'05.88"E; 370 m. SAM_H4247 (1 specimen): Eastern margin, 8 km south of Ponta Do Ouro/7 km off Kosi-Kumpungwini (Sifungwe) Estuary, 26°55'18.12"S, 32°55'05.88"E; 50 m. **SAM_H4592 (1 specimen)**: Southern margin, 32 km off Mazeppa Bay/24 km off Kobole Estuary, 32°28'36.00"S, 28°58'48.00"E; 710–775 m. **USNM 77237 (1 specimen)**: Eastern margin, 46 km from Port Dunford/45 km off Nyoni Estuary, 29°19'00.00"S, 32°00'00.00"E; 366 m. **USNM 91791 (4 specimens, sub-sample of SAM_H3136)**: Eastern margin, 28 km from Coffee Bay/19 km off Bulungulu Estuary, 32°14'53.99"S, 29°10'23.99"E; 620–560 m.

###### Description.

Corallum variable in shape, ranging from conical to subcylindrical, sometimes scolecoid, but always attached to substrate by a robust pedicel (PD:GCD = 0.3–0.6) that expands into a thin encrusting base. Calice circular to slightly elliptical (GCD:LCD = 1.0–1.1), with a slightly serrate calicular margin. Theca thick and uniformly hispid. Costae absent. Lower epitheca well developed, covering synapticulotheca, covered in granules. Corallum white.

Septa hexamerally arranged in four complete cycles according to the formula: S_1_ > S_2_ > S_4_ > S_3_ (48 septa). S_1_ most exsert. S_2_ slightly less exsert than S_1_, and being ¾ the width of S_1_. Both S_1_ and S_2_ bearing straight to slightly sinuous axial margins. Higher cycle septa (S_3–4_) becoming progressively less exsert and smaller. S_3_ is ½ the width and less sinuous than S_2_. S_4_ rudimentary, and bear the least sinuous but slightly dentate axial margins. Septal faces granular. Fossa deep, containing a rudimentary columella.

###### Distribution.

Regional: Southern to eastern margin of South Africa, extending from off Gansbaai towards Kosi-Kumpungwini (Sifungwe) Estuary (8 km south of Ponta Do Ouro: Mozambique); 50–775 m. Elsewhere: Only known from South Africa.

###### Remarks.

*Pourtalopsammiatogata* appears to be endemic to South Africa, and records presented herein extend its distribution further south towards Gansbaai. Furthermore, the genus is monotypic and closely resembles *Trochopsammia* Pourtalès, 1878 but differs in having a hispid theca (Cairns and Keller 1993; Cairns 2001).

##### Rhizopsammia

Taxon classificationAnimaliaScleractiniaDendrophylliidae

Verrill, 1870

807089FF-879D-5A8C-8EA6-77AF9B05CF82

###### Diagnosis.

Small reptoid colonies produced by extra-tentacular stoloniferous budding. Synapticulotheca of finely granular costae, often covered with epitheca. Pourtalès plan present; pali absent. Columella spongy, usually small. Endotheca absent.

###### Type species.

*Rhizopsammiapulchra* Verrill, 1870, by monotypy

##### Rhizopsammia annae

Taxon classificationAnimaliaScleractiniaDendrophylliidae

(van der Horst, 1933)

73969151-21F2-5C97-AA2A-BCDF78309D81

Fig. 10I, J

Balanophyllia annae van der Horst, 1933: 156–158, pl. 7, fig. 1–4. –van der Horst 1938, pl. 5, fig. 1. –Boshoff 1981: 40.
Rhizopsammia annae . –Zibrowuis and Gili 1990: 44. –Cairns and Keller 1993: 276, fig. 13C. –Cairns 2001: 27.

###### Type locality.

Oudekraal, Cape Peninsula, South Africa; depth unknown (van der Horst 1938).

###### Type material.

Unknown.

###### Material examined.

ORI_EIg3 (10 specimens), SAMC_A072994 (3 specimens): Locality data unknown. SAMC_A073016 (3 specimens): Eastern margin, 31 km from Richards Bay/46 km Mlalazi Estuary, 29°00'54.00"S, 32°15'35.99"E; 500 m. SAM_H1497 (1 specimen): Southern margin, 3 km from Agulhas/16 km off De Mond-Heuningnes Estuary, 34°49'52.54"S, 20°00'49.88"E; depth unknown. SAM_H1498 (1 specimen):) : Southern margin, 3 km from Agulhas/16 km off De Mond-Heuningnes Estuary, 34°49'52.54"S, 20°00'49.88"E; depth unknown. SAM_H3041 (6 specimens): Southern margin, 2 km from Mosselbaai/10 km off Hartenbos Estuary, 34°10'37.57"S, 22°09'19.14"E; 55 m. SAM_H3366 (6 specimens): Southern margin, 15 km from Port Elizabeth/16 km off Bakens River Estuary, 33°49'59.99"S, 25°40'00.00"E; depth unknown.

###### Imagery data.

UCTES_FAL 368 L (3 specimens): Southern margin, 19 km from Pringle Bay/16 km off Buffels Oos Estuary, 34°12'36.00"S, 18°46'41.99"E; 40 m. UCTES_SCD 268 F (5 specimens): Southern margin, 6 km from East London/4 km off Buffalo Estuary, 33°02'30.00"S, 27°56'30.00"E; 55 m.

###### Description.

Reptoid colonies formed by ceratoid to cylindrical, straight to slightly curved corallites attached through a pedicel that expands into a thin encrusting base. Corallites united by narrow basal stolons. Calice circular to slightly elliptical (GCD:LCD = 1.0–1.2), calicular margin lanceted. Largest specimen examined (SAM_H1497) 6.3 × 5.3 mm in CD, and 13.4 mm in H. Epitheca variable in development: in some corallites epitheca reaches calicular margin, but in other corallites it is restricted to base. Costae conspicuous below theca, being equal in width and covered with fine, pointed, and randomly arranged granules. Intercostal striae porous and thin. Corallum white.

Septa hexamerally arranged in four cycles according to the formula: S_1_ ≥ S_2_ > S_4_ > S_3_ (≤ 48 septa). S_1–2_ independent. S_1_ most exsert, extending to columella with dentate axial margins. S_2_ slightly less exsert, sometimes slightly less wide, but otherwise similar in profile as S_1_. Higher cycle septa (S_3–4_) progressively less exsert, if at all. S_3_ is ^1^/_3_ the width of S_2_, with straight to slightly sinuous axial margins. S_4_ ¾ wider than S_3_, merging towards flanked S_3_ and again in front of S_2_ before joining columella as one septum. S_4_ axial margins the most dentate (but with fewer teeth). Septa commonly closely packed, with all septal faces being granular. Fossa of moderate depth, containing a spongy columella.

###### Distribution.

Regional: Southern and eastern margin of South Africa, from Pringle Bay extending towards Richards Bay; 40–500 m. Elsewhere: Only known from South Africa.

###### Remarks.

As noted by previous authors (van der Horst 1922; Cairns 1994; Cairns and Zibrowius 1997), a damaged *Rhizopsammia* specimen may be confused with *Balanophyllia* as it is the intact stolons that distinguish *Rhizopsammia* from other dendrophylliids. Nonetheless, the examined specimens agree with van der Horst’s (1922) description of the species, apart from a couple of specimens (i.e., SAMC_A072994) that followed a divergent septa arrangement according to: S_1_ > S_3 >_ S_2_ > S_4._

##### Rhizopsammia compacta

Taxon classificationAnimaliaScleractiniaDendrophylliidae

Sheppard & Sheppard, 1991

24C62C33-651C-53AC-B9AE-2219D08B9122

Fig. 10K, L

Rhizopsammia compacta Sheppard & Sheppard, 1991: 153, fig. 179. –Cairns and Keller 1993: 277, fig. 13B, E.
Balanophyllia bairdiana . –Boshoff 1981: 40.
Balanophyllia ponderosa . –Boshoff 1981: 41.

###### Type locality.

Off Musandam, Gulf of Oman; 35 m (Cairns and Keller 1993).

###### Type material.

The holotype is lodged at the BMNH (GBIF 2020).

###### Material examined.

ORI_EIa2 (2 specimens): Eastern margin, 14 km from Durban/12 km off Mbokodweni Estuary, 29°58'00.00"S, 31°01'59.99"E; 49 m. ORI_EIa6 (40 specimens), ORICH_1 (2 specimens): Eastern margin, other locality data unknown. SAMC_A072979 (20 specimens): Southern margin, 21 km off Port Elizabeth/16 km off Bakens River Estuary, 33°58'59.99"S, 25°46'59.99"E; 90 m. SAMC_A073033 (1 specimen): Eastern margin, 28 km south of Ponta Do Ouro/17 km off Kosi Bay Estuary, 27°06'18.00"S, 32°52'00.12"E; 50 m. SAMC_A073041 (1 specimen): 24 km south of Ponta Do Ouro/16 off Kosi Bay Estuary, 27°04'00.00"S, 32°52'59.99"E; 69 m. SAMC_A073081 (6 specimens): Eastern margin, 27 km from Richards Bay/40 km off Mlalazi Estuary, 29°00'24.11"S, 32°12'00.00"E; 152 m. SAMC_A073083 (6 specimens): Eastern margin, 28 km from Richards Bay/40 km off Mlalazi Estuary, 29°00'54.00"S, 32°12'06.12"E; 215 m. SAMC_A073136 (1 specimen): Eastern margin, 39 km from Cape Vidal/29 km off Mgobezeleni Estuary, 27°47'23.99"S, 32°38'53.87"E; 65–70 m. SAMC_A073192 (3 specimens): Eastern margin, 69 km from Cape Vidal/7 km off Mgobezeleni Estuary, 27°31'48.00"S, 32°42'47.99"E; 70 m. **SAMC_A073199 (1 specimen, sub-sample of USNM 91800)**: Eastern margin, 17 km south of Ponta Do Ouro/11 km off Kosi-Kumpungwini (Sifungwe) Estuary, 27°00'17.99"S, 32°55'18.12"E; 67 m. SAMC_A073204 (1 specimen): Eastern margin, 67 km from Cape Vidal/7 km off Mgobezeleni Estuary, 27°32'48.12"S, 32°42'36.00"E; 68 m. SAMC_A073208 (1 specimen): Eastern margin, 16 km from St. Lucia Estuary/17 km off Mfolozi Estuary, 28°17'30.11"S, 32°32'35.88"E; 50 m. SAMC_A073221 (1 specimen): Eastern margin, 68 km from Cape Vidal/5 off Mgobezeleni Estuary, 27°31'59.99"S, 32°42'00.00"E; 77 m. SAMC_A090105 (2 specimens): Locality data unknown. SAMC_A090112 (1 specimen): Southern margin, 11 km from Pringle Bay/8 km off Buffels Oos Estuary, 34°16'29.99"S, 18°49'29.99"E; 14–17 m. SAM_H1373 (5 specimens): Western margin, 14 km from Cape Town/13 km off Diep Estuary, 33°57'31.20"S, 18°22'20.64"E; depth unknown. SAM_H1375 (1 specimen): Western margin, 14 km from Cape Town/13 km off Diep Estuary, 33°57'31.20"S, 18°22'20.64"E; 44 m. SAM_H1380 (2 specimens): Southern margin, 16 km from Cape Point/8 km off Elsies Estuary, 34°12'59.05"S, 18°27'57.32"E; 40 m. SAM_H1414 (1 specimen): Southern margin, 16 km from Cape Point/8 km off Elsies Estuary, 34°12'59.05"S, 18°27'57.32"E; depth unknown. SAM_H1686 (6 specimens): Southern margin, 16 km from Cape Point/8 km off Elsies Estuary, 34°12'59.05"S, 18°27'57.32"E; depth unknown. SAM_H 2844 (1 specimen): Southern margin, 6 km from Kenton On Sea/5 km off Boknes Estuary, 33°43'07.59"S, 26°37'37.95"E; 90 m. **SAM_H4578 (1 specimen)**: Eastern margin, 6 km south of Ponta Do Ouro/9 km off Kosi-Kumpungwini (Sifungwe) Estuary, 26°53'30.00"S, 32°55'36.00"E; 51 m. **SAM_H4579 (2 specimens)**: Eastern margin, 42 km south of Ponta Do Ouro/27 km off Kosi Bay Estuary, 27°13'30.00"S, 32°49'30.00"E; 74 m. **USNM 91807 (2 specimens)**: Eastern margin, 38 km south of Ponta Do Ouro/23 km off Kosi Bay Estuary, 27°11'17.99"S, 32°50'14.39"E; 78 m.

###### Description.

Colony irregularly shaped, composed of corallites interconnected by narrow stolons, or composed of small corallites budding from lower edge zone of larger corallites. Corallites ceratoid to cylindrical, bearing elliptical calices (GCD:LCD = 1.4–1.8), calicular margin lanceted. Largest colony examined (ORI_EIa6) consists of 20 corallites. Largest corallite (SAMC_A073041) 20.2 × 13.0 mm in CD, and 33.5 mm in H. If present, epitheca thin. Costae conspicuous throughout corallum, equal in width, covered with fine, pointed, and randomly arranged granules. Intercostal striae thin and deep. Corallum white.

Septa hexamerally arranged in five cycles according to the formula: S_1_ ≥ S_2_ > S_3_ > S_5_ > S_4_, with occasional pairs of S_6_ in half-systems (≤ 112 septa). S_1–2_ both independent, most exsert, reaching columella with straight and smooth axial margins that become dentate deep in fossa. S_3_ slightly less to as exsert as S_1–2_, but having only ½ the width of S_1–2_, but otherwise similar in profile. Higher cycle septa (S_4–5_) progressively less exsert, if at all. S_4_^1^/_3_ the width of S_3_, and have the least dentate lower axial margins. S_5_ ¾ wider than S_4_, merging towards S_4_ before joining columella as one septum. S_5_ axial margins dentate to laciniate. S_6_, if present, restricted to calicular margin. Septa commonly closely packed, with all septal faces being granular. Pali absent. Fossa of moderate depth, containing a spongy columella that fuses with axial margins of all septa except S_4_ (and S_6_ if present).

###### Distribution.

Regional: Western to eastern margin of South Africa, extending from off Cape Town towards Kosi-Kumpungwini (Sifungwe) Estuary (9 km south of Ponta Do Ouro: Mozambique); 40–215 m. Elsewhere: Gulf of Oman (Sheppard and Sheppard 1991; Cairns and Keller 1993); Mozambique (Cairns and Keller 1993); 35–110 m.

###### Remarks.

Our examined specimens agree with the redescription of Cairns and Keller (1993) of *Rhizopsammiacompacta*. Although the lack of stolons may cause confusion with *Balanophylliadiademata* based on van der Horst’s (1927) description (and associated image), *R.compacta* can be distinguished by consistently having five complete septal cycles (occasionally with a pair of S_6_ = 6:6:12:24:48:16) as compared with five incomplete cycles of *B.diademata* (6:6:12:24:34). *Rhizopsammiacompacta* may be distinguished from the other South African congeners by its relatively large adult corallum (20.0 mm CD), as compared with the medium-sized *R.verrilli* van der Horst, 1922 (8.0 mm) and the small *R.annae* (6.3 mm). The hexamerally arranged septa in six cycles (≤ 112 septa) may also assist with separating *R.compacta* from *R.verrilli* and *R.annae*, which both have septa hexamerally arranged in four cycles (48 septa).

##### Rhizopsammia verrilli

Taxon classificationAnimaliaScleractiniaDendrophylliidae

van der Horst, 1922

AFE239E9-53E5-5D1B-A3A6-C8914EEEB0A0

Fig. 10M, N

Rhizopsammia verrilli van der Horst, 1922: 64, pl. 8: figs 1, 2. –Wells 1983: 241–242, pl. 15: figs 1–4. –Cairns 1991: 25, pl. 22, figs C–E. –Cairns and Zibrowius 1997: 188–189, fig. 28F, G. –Cairns 1998: 408 . –Cairns 2004a: 318.
Balanophyllia scheeri Durham, 1962:46, 53–54, figs 2B, C, 4, 7 . –Durham 1966: 125.
Dendrophyllia gracilis . –Cairns 1991: 23.

###### Type locality.

Indonesia (HMS ‘Siboga’ stns. 220 and 282); 54–278 m (van der Horst 1922).

###### Type material.

Syntypes are deposited at the ZMA (Cairns 2004a).

###### Material examined.

SAMC_A073042 (8 specimens): Eastern margin, 53 km from Shaka’s Rock/46 km off Zinkwasi Estuary, 29°32'53.88"S, 31°47'12.11"E; 200 m. SAMC_A073157 (5 specimens): Eastern margin, 10 km from Port Edward/24 km off Bilanhlolo Estuary, 31°05'48.11"S, 30°18'47.88"E; 140 m. SAMC_A073197 (2 specimens): Eastern margin, 18 km from St. Lucia Estuary/15 km off Mfolozi Estuary, 28°31'48.00"S, 32°26'06.00"E; 160–180 m. SAMC_A073198 (1 specimen): Eastern margin, 10 km from Shaka’s Rock/12 km off Mhlali Estuary, 29°32'12.00"S, 31°19'47.99"E; 50 m. SAM_H1502 (1 specimen): Eastern margin, 14 km from Richards Bay/23 km off Mlalazi Estuary, 28°52'59.99"S, 32°01'00.00"E; 165 m.

###### Description.

Colony consists of corallites interconnected by narrow stolons, and composed of small corallites budding from upper margin zone of larger corallites. Corallites ceratoid to cylindrical. Largest examined corallite (SAMC_ A073198) 9.3 × 8.8 mm in CD, and 21.5 mm in H. Epitheca thin, if present. Costae granular, conspicuous throughout corallum, and equal in width. Intercostal striae thin, porous. Corallum white.

Septa hexamerally arranged in four cycles according to the formula: S_1_ > S_2_ ≥ S_4_ > S_3_ (48 septa). S_1–2_ independent, most exsert, and joining columella deeper in fossa with straight axial margins. S_2_ slightly less wide than S_1_. Higher cycle septa (S_3–4_) progressively less exsert. S_3_^1^/_3_ the width of S_2_, and bear laciniate axial margins. In complete half-systems a pair of S_4_ meets before S_3_ and joins columella as one septum. S_4_ axial margins dentate to laciniate. Septa appearing loosely packed, with all septal faces covered with granules. Pali absent. Fossa of moderate depth, containing a spongy columella.

###### Distribution.

Regional: Eastern margin of South Africa, extending from off Port Edward towards St Lucia; 50–200 m. Elsewhere: Philippines (Cairns and Zibrowius 1997); Indonesia (van der Horst 1922; Cairns and Zibrowius 1997); New Caledonia (Kitahara and Cairns 2021); Galápagos Islands (Cairns 1991); Cocos Island (Durham 1962); Mozambique? (MacNae and Kalk 1958); 2–700 m.

###### Remarks.

Based on a literature comparison, the South African *Rhizopsammiaverrilli* differs from the previously reported Indo-Pacific representatives by having four complete cycles instead of five, the last incomplete. Striking similarities between *R.verrilli* and *R.wettsteini* Scheel & Pillai, 1983 have been highlighted (Arrigoni et al 2014), however specimens of the latter have not been examined and thus a comparison is not detailed herein. Specimens reported here are new records for the South African region, but not for the southwest Indian Ocean, as this *R.verrilli* has been recorded off Mozambique by MacNae and Kalk (1958). This Mozambican record forms part of the Inhaca collection, presumably examined by Boshoff and reported in MacNae and Kalk’s (1958). Unfortunately, the subsequent Boshoff (1981) checklist historically raised concerns on the reliability of *R.verrilli* in the southwest Indian Ocean. Furthermore, Cairns and Keller (1993) did not list this species in their southwest Indian Ocean account. However, the specimens reported herein confirm the occurrence *R.verrilli* in the region. Nonetheless, the Inhaca collection is of concern and the examination of these specimens is of high priority for biodiversity assessments of the region.

##### Tubastraea

Taxon classificationAnimaliaScleractiniaDendrophylliidae

Lesson, 1829

30D67745-8C3B-5EB9-BBD5-E2140E387371

###### Diagnosis.

Colonies dendroid, bushy, or plocoid, all achieved by extra-tentacular budding. Costate, no epitheca. Septa arranged normally. Pali absent. Columella usually small and spongy.

###### Type species.

*Tubastraeacoccinea* Lesson, 1829, by monotypy.

##### Tubastraea coccinea

Taxon classificationAnimaliaScleractiniaDendrophylliidae

Lesson, 1829

86B597DA-A831-5A2B-8638-969E24FB6605

Fig. 10O

Tubastraea coccinea Lesson, 1829: 93. –Wells 1936: 132. –Cairns et al. 1991: 48 . –Cairns 1991a: 26–27, pl. 12, figs C–E. –Ogawa and Takahashi 1993: 98, pl. 1, figs 1–8, pl. 2, figs 1–4, pl. 5, figs 1–5. –Cairns and Keller 1993: 282–284. –Cairns 1994: 93–94, pl. 39, figs G–I. –Cairns and Zibrowius 1997: 197. –Cairns 1998: 409. –Cairns et al. 1999: 27. –Cairns 2000: 178–180, figs 212–215. –Romano and Cairns 2000: 1049. –Cairns 2001: 29, pl. 10, figs I–L. –Randall 2003: 136. –Cairns 2004a: 318. –Tachikawa 2005: 20, pl. 13, figs A–C. –Cairns 2006: 49. –Kitahara 2007: 504–505, 515, fig. 5K. –Pires 2007: 269.– Cairns 2009: 27. –Lam et al. 2008: 736, fig. 2A, B. –Kitahara et al. 2010a: 115. – Kitahara et al. 2010b: 9. –Kitahara and Cairns 2021: 351, 353–354, figs 188A–B, 189.
Lobopsammia aurea Quoy & Gaimard, 1833: 195, pl. 15, figs 7–11.
Dendrophyllia aurantiaca Dana, 1846: 388.
Coenopsammia coccinea . –Milne-Edwards and Haime 1848b: 107–108.
Coenopsammia ehrenbergiana Milne-Edwards & Haime, 1848b: 109, pl. 1, fig. 12.
Dendrophyllia ehrenbergiana . –van der Horst 1922: 55–56, 74, pl. 7.
Tubastraea tenuilamellosa . –Durham 1947: 38–39, pl. 11, figs 1, 2, 4, 9, pl. 12, figs 6, 7. –Durham and Barnard 1952: 105–106, pl. 12, fig. 50D. –Boschma 1953: 109–117, pl. 9, figs 1–4, pl. 10, figs 1, 3–5, pl. 11, figs 1, 3.
Tubastrea tenuilamellosa . –Boschma 1951: 44–46. –Durham 1962: 42, 44–46. –Olivares 1971: 75–77, pl. 2, figs A, B.
Tubastraea aurea . –Boschma 1953: 111–118 (in part: pl. 10, figs 2, 6, pl. 11, figs 4–6, pl. 12, figs 1–6). –Stephenson and Wells 1956: 59. –Squires 1959b: 427–428. –Pichon 1964: 191. –Eguchi 1965a: 295. –Utinomi 1965: 257–258. –Squires 1966: 169.
Dendrophyllia aurea . –Macnae and Kalk 1958: 123.
Dendrophyllia coccinea . –Eguchi 1965a: 296. –Utinomi 1965: 257. –Boshoff 1981: 41.
Tubastrea coccinea . –Latypov 1990: 66–67, pl. 27, fig. 1, pl. 32, fig. 3.

###### Type locality.

Bora-Bora, Society Islands; depth unknown (Lesson 1829).

###### Type material.

The holotype is deposited at the MNHNP (Wells 1936; Cairns 1994).

###### Material examined.

**ORI_EIb4 (1 specimen)**: Eastern margin, Isipingo; depth unknown.

###### Description.

Colony plocoid, formed by extra-tentacular budding at colony margin. Calices adjacent to each other, and project slightly above coenosteum. Corallites circular to slightly elliptical (GCD:LCD = 1.0–1.3), reaching ≤ 10.0 mm in CD. Costae prominent, equal in width, and granulated. Intercostal striae deep and porous. Corallum white.

Septa hexamerally arranged in four complete cycles according to the formula: S_1_ ≥ S_2_ > S_3_ > S_4_ (≤ 48 septa). S_1_ extend to columella with vertical axial margins. S_2_ equal or only slightly less wide than S_1_, also with straight axial margins. S_3_ ~ ¼ the width of S_2_, and bear dentate to laciniate axial margins. S_4_ rudimentary, but sometimes joining before S_3_ in a weak Pourtalès plan. S_4_ axial margins laciniate. All septa non-exsert. Fossa of moderate depth containing a columella ranging from rudimentary to a large spongy structure.

###### Distribution.

Regional: Eastern margin of South Africa, off Isipingo (Boshoff 1981); depth unknown. Elsewhere: Cosmopolitan in tropical shallow and warm temperate waters (Cairns and Zibrowius 1997); 0–110 m.

###### Remarks.

*Tubastraeacoccinea*, an Indo-Pacific species, has been noted to exhibit a highly invasive footprint in the Atlantic (Cairns 1994, 2000; Sammarco et al. 2010) during the last seven decades. Six Recent species (*T.coccinea*, *T.diaphana* (Dana, 1846), *T.faulkneri* Wells, 1982, *T.floreana* Wells, 1982, *T.micranthus* (Ehrenberg, 1834), *T.tagusensis* Wells, 1982) are recogniszed to date; three of which occur off South Africa (*T.coccinea*, *T.diaphana*, *T.micranthus*) and are distinguished from one another by colony size and shape (Cairns and Keller 1993; Cairns and Zibrowius 1997).

##### Tubastraea sp. cf. diaphana

Taxon classificationAnimaliaScleractiniaDendrophylliidae

(Dana, 1846)

2339ABBC-D306-5234-9318-F8059D340E6C

Figs 10P , 11A

Dendrophyllia diaphana Dana, 1846: 389, pl. 27, fig. 3. –Vaughan 1918: 144–145, pl. 60, figs 2, 3.
Dendrophyllia aequiserialis Quelch, 1886: 147.
Dendrophyllia micranthus var. fruticosa Nemenzo, 1960: 17–18, pl. 9, fig. 1.
Tubastraea diaphana . –Scheer and Pillai 1983: 174, pl. 41, figs 1–4. –Cairns and Keller 1993: 284, pl. 13, fig. H. –Cairns and Zibrowius 1997: 196–197. –Cairns 1998: 409–410 . –Cairns 2001: 29 . –Cairns 2004a: 318.
Dendrophyllia sibogae van der Horst,1922: 56–57, pl. 8, figs 18, 19.

###### Type locality.

Singapore, depth unknown (Dana 1846).

###### Type material.

The holotype is deposited at the NMNH (Cairns 1994).

###### Material examined.

SAM_H5103 (1 specimen): Eastern margin, 59 km from Cape Vidal/9 km off Mgobezeleni Estuary, 27°36'38.45"S, 32°40'02.99"E; 59 m.

###### Description.

Colonies phaceloid, forming small bushy clusters of corallites. Branching achieved by extra-tentacular budding fairly closely at broad base (BD = 1.6). Corallites circular to slightly elliptical (GCD:LCD = 1.0–1.1), and ≤ 27.4 mm in H. Theca thin and porous. Costae well developed, particularly C_1,_ which is the same-size as associated primary septa. Corallum white. Tissue pale orange in live specimen.

Septa hexamerally arranged in four cycles, last cycle being incomplete, according to the formula: S_1_ > S_2_ > S_3_–_4_ (48 septa). S_1_ exsert, with straight axial margins. S_2_ less than ¼ width of S_1_, and have dentate axial margins. S_3–4_ rudimentary, being wider deeper in fossa, and bearing dentate lower axial margins. Fossa of exceptional depth and spacious, containing a spongy columella.

###### Distribution.

Regional: Eastern margin of South Africa, off Aliwal shoal (Cairns and Keller 1993) and Cape Vidal; 9–59 m. Elsewhere: Zanzibar; Madagascar (Cairns and Keller 1993); Red Sea (Scheer and Pillai 1983); Australia (Cairns 2004a); Philippines (Cairns and Zibrowius 1997); Indonesia (van der Horst 1922); New Caledonia (Kitahara and Cairns 2021); Singapore (Dana 1946); 1–30 m

###### Remarks.

The examined specimen closely resembles *Tubastraeadiaphana* (Dana, 1846) in that its corallum is phaceloid, a species reported by Cairns and Keller (1993) to occur in the Natal region. However, differences are observed in the tertiary and quaternaries septa: both being restricted to the calicular margin, tissue being pale orange, and in bearing a spongy columella (Fig. 10O). The specimen examined herein was collected at 23 m deeper depth than the maximum depth of *Tubastraeadiaphana*.

##### Tubastraea micranthus

Taxon classificationAnimaliaScleractiniaDendrophylliidae

(Ehrenberg, 1834)

40F215D2-0DB8-570E-8815-5CAFEB6672A3

Fig. 11B

Oculina micranthus Ehrenberg, 1834: 304.
Dendrophyllia nigrescens Dana, 1846: 387. –Vaughan 1918: 143–144, pl. 60, figs 1, 1A. –Searles 1956: 24, pl. 39A. –Stephenson and Wells 1956: 55. –Wells 1964: 108.
Coenopsammia viridis Milne-Edwards & Haime, 1848b: 110.
Dendrophyllia micranthus . –van der Horst 1922: 49–51. –van der Horst 1926: 43–44, pl. 2, figs 6, 7. –Faustino 1927: 218–220, pl. 72, figs 1, 2. –Crossland 1952: 171–172. –Stephenson and Wells 1956: 55. –Nemenzo 1960: 16–17, pl. 8, fig. 2. –Scheer and Pillai 1974: 63, pl. 29, fig. 3. –Pillai and Scheer 1976: 16. –Betterton 1981: 242, figs 199–200. –Boshoff 1981: 41.
Dendrophyllia micranthus var. grandi Crossland, 1952: 173, pl. 55, fig. 1, pl. 56, fig. 1.
Tubastraea micranthus . –Macnae and Kalk 1958: 123. –Scheer and Pillai 1983: 175–176, pl. 41, figs 7–8. –Schuhmacher 1984: 94, figs 1A–B, 4. –Zibrowius and Grygier 1985: 130. –Cairns and Zibrowius 1997: 195–196. –Cairns 1998: 410. –Cairns et al. 1999: 28. –Cairns 2001: 29. –Paula and Creed 2004: 176, 181. –Cairns 2004a: 267, 318. –Tachikawa 2005: 20–21, pl. 13, figs G–K. –Sammarco et al. 2010: 131–140, figs 2A, 3A, 4A. –Cairns 2009: 28.
Tubastrea micrantha . –Wells 1964: 108. –Ogawa and Takahashi 1993: 99–100, pl. 4, figs 1–6, pl. 6, figs 5, 6.
Dendrophyllia cf. micrantha . –Best et al. 1980: 621.
Tubastraea micrantha . –Pichon 1978: 441. –Wells 1983. –Veron 1986: 583, fig. 3, 585, figs 3, 7. –Cairns and Keller 1993: 282. –Romano and Cairns 2000: 1049.
Dendrophyllia diaphana . –Boshoff 1981: 42.

###### Type locality.

Unknown.

###### Type material.

Unknown.

###### Material examined.

**ORI_EIb3 (1 specimen)**: Locality data unknown.

###### Description.

Colony dendroid, more or less uniplanar, branching formed by extra-tentacular budding. Coralla reaching ≤ 125.0 mm in H, and 30.0 mm in PD. Corallites projecting at 45° angle from axial branch, being circular to slightly elliptical (GCD:LCD = 1.1–1.5), and reaching ≤ 7 mm in CD. Calicular margin slightly serrated. Theca thin and porous, particularly near calicular margin. Costae well developed, ridged, and granular. Intercostal striae porous. Corallum white and more porous distally. Coenosarc dark green or brown in live specimens.

Septa hexamerally arranged in three complete cycles according to the formula: S_1_ > S_2_ >> S_3_. S_1_ non-exsert with straight axial margins. S_2_ ~ ¾ the width of S_1_, also having straight axial margins. S_3_ rudimentary, and bearing dentate to laciniate axial margins. Fossa of deep, especially that of axial corallites. Columella rudimentary.

###### Distribution.

Regional: Eastern margin of South Africa (Boshoff 1981); depth unknown. Elsewhere: Mozambique (Cairns and Keller 1993); Red Sea (Scheer and Pillai 1983); Comoro Islands (Schuhmacher 1084); Seychelles (Milne-Edwards and Haime 1848b); Madagascar (Pichon 1978); Mauritius; Maldives; Nicobar Islands (Scheer and Pillai 1974); Australia; (Cairns 2004a); wide-spread in the tropical Pacific (Cairns and Zibrowius 1997); 0–50 m.

**Figure 11.**
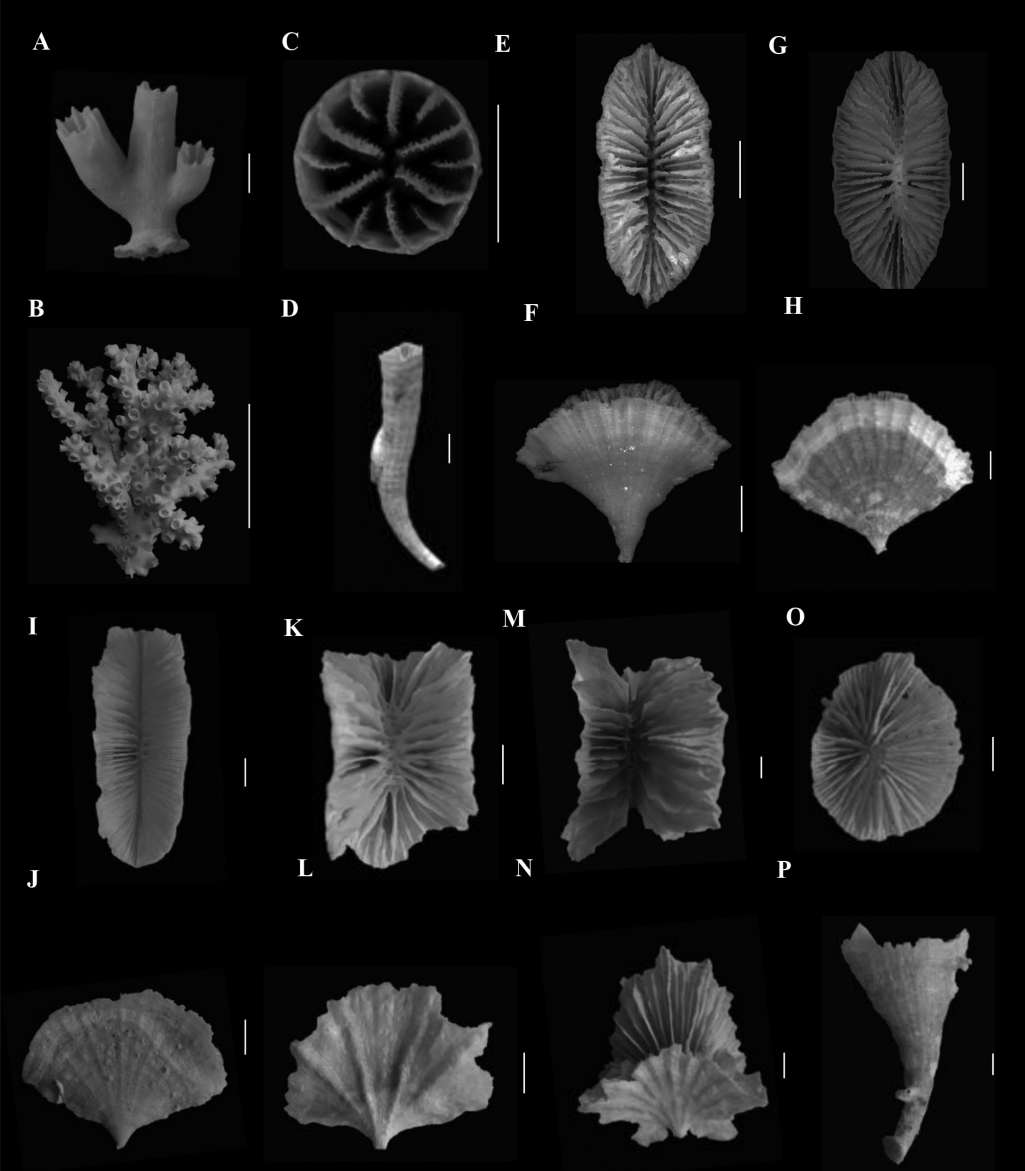
**A**Tubastraea sp. cf. diaphana (SAM_H5103, Cape Vidal, 59 m) **A** lateral view **B***Tubastraeamicranthus* (ORI_EIb3, locality data unknown) **B** full view **C**, **D**Flabellum (Flabellum) leptoconus (ORI_DIIa1, Port St. Johns, 409–440 m) **C** calicular view **D** lateral view **E**, **H**Flabellum (Flabellum) pavoninum**E**, **F** (SAMC_A073135, off Coffee Bay, 250–280 m) **E** calicular view **F** lateral view **G**, **H** (SAM_H4582, off Shaka’s Rock, 150 m) **G** calicular view **H** lateral view **I**, **J**Flabellum (Flabellum) politum (SAMC_ A090160, off Shaka’s Rock, 53–57 m) **I** calicular view **J** lateral view **K**, **L**Flabellum (Ulocyathus) alabastrum (SAMC_ A090102, off the Agulhas, 168 m) **K** calicular view **L** lateral view **M**, **N**Flabellum (Ulocyathus) lowekeyesi (SAM_H1695, off Kosi Bay Estuary, 720–780 m) **M** calicular view **N** lateral view **O**, **P***Javaniaantarctica* (SAMC_A090150, off Gouritsmond, 333 m) **O** calicular view **P** lateral view. Scale bars: 10 mm.

###### Remarks.

*Tubastraeamicranthus* superficially resembles *Dendrophylliaijimai* in having a more or less uniplanar colony, but can be distinguished in bearing three normally arranged septal cycles as compared with five incomplete cycles arranged in a Pourtalès plan in the latter. This arborescent and sympodial branching further makes *T.micranthus* unique to the other congeners (Cairns and Keller 1993).

#### Family Flabellidae Bourne, 1905

##### Flabellum

Taxon classificationAnimaliaScleractiniaFlabellidae

Lesson, 1831

B9D8AD6B-1E62-51EE-B2DE-9B6E1E6B3A34

###### Diagnosis.

Corallum solitary; attached or free. Corallum shaped in a variety of ways, including ceratoid, campanulate, bowl-shaped, discoidal, or compressed-flabellate. Transverse division lacking. Base and lower pedicel not reinforced with stereome. Wall epithecal, usually porcelaneous, without costae. Pali, dissepiments, and synapticulae absent. Columella rudimentary to absent, when present a simple fusion of lower axial margin of larger septa. Exclusively azooxanthellate.

##### Flabellum (Flabellum)

Taxon classificationAnimaliaScleractiniaFlabellidae

Lesson, 1831

25E12231-1D41-54F2-92B0-5A7B6EBED9DA

###### Diagnosis.

*Flabellum* having a smooth calicular margin.

###### Type species.

*Flabellumpavoninum* Lesson, 1831, by monotypy.

##### Flabellum (Flabellum) leptoconus

Taxon classificationAnimaliaScleractiniaFlabellidae

Cairns & Zibrowius, 2016

986D1DCE-3222-5C66-8535-A402E0CDE8E3

Fig. 11C, D

Flabellum sibogae Gardiner, 1904: 98. –Cairns and Keller 1993: 220, 221.
Adkinsella sp. –Boshoff 1981: 34.
Flabellum (Flabellum) leptoconus Cairns & Zibrowius, 2016: 161–162, fig. 1F, J.

###### Type locality.

South of Natal region, South Africa (USNM 62498: 31°23'00"S, 29°54'00"E); 409–440 m (Cairns and Zibrowius 2016).

###### Type material.

The holotype and three paratypes are deposited at the NMNH (Cairns and Zibrowius 2016), whilst one paratype is deposited at ORI.

###### Material examined.

**ORI_DIIa1 (1 specimen: paratype)**: Southern margin, 44 km from Port St. Johns/4 km from Mkweni Estuary, 31°22'59.99"S, 29°53'59.99"E; 409–440 m. DSCS INV–358 (2 specimens): Southern margin, 92 km from Oubosstrand/89 km off Tsitsikamma Estuary, 34°53'21.93"S, 24°06'56.47"E; 355 m. DSCS–INV 569 (2 specimens): Southern margin, 65 km from Cape St. Francis/70 km from Slang Estuary, 34°47'05.01"S, 24°45'42.30"E; 392 m. SAMC_A073153 (2 specimens): Eastern margin, 19 km from Coffee Bay/18 km off Mdumbi Estuary, 32°02'53.87"S, 29°19'41.87"E; 250–280 m. SAMC_A073179 (1 specimen): Southern margin, 34 km from Mazeppa Bay/17 km off Mendu Estuary, 32°23'35.88"S, 28°59'12.11"E; 295–350 m. SAMC_A073190 (4 specimens): Southern margin, 34 km from Mazeppa Bay/17 km off Mendu Estuary, 32°23'35.88"S, 28°59'12.11"E; 295–350 m. SAMC_A090154 (1 specimen): Southern margin, 58 km from Port Alfred/35 km off Mgwalana Estuary, 33°39'10.19"S, 27°29'57.58"E; 304 m. **SAM_H1239** (1 specimen): Southern margin, 26 km from Cape Point/20 km off Sand Estuary, 34°10'22.29"S, 18°39'51.94"E; 567 m. SAM_H2841 (1 specimen): Eastern margin, 20 km from Cape Vidal/23 km off St Lucia Estuary, 27°59'29.99"S, 32°40'47.99"E; 550 m. SAM_H3150 (1 specimen): Southern margin, 48 km from Port Alfred/19 km off Mgwalana Estuary, 33°32'59.99"S, 27°22'59.99"E; 80 m. SAM_H3180 (2 specimens): Eastern margin, 28 km from Coffee Bay/19 km off Bulungulu Estuary, 32°14'53.99"S, 29°10'23.99"E; 620–560 m. SAM_H3181(I specimen): Eastern margin, 30 km from Scottburgh/20 km off Fafa Estuary, 30°33'23.99"S, 30°48'35.99"E; 690 m. SAM_H3182 (24 specimens): Southern margin, 40 km from Cintsa/29 km from Cwili Estuary, 32°54'59.99"S, 28°30'59.99"E; 630 m. SAM_H3183 (7 specimens): Southern margin, 32 km off Mazeppa Bay/24 km off Kobole Estuary, 32°28'36.00"S, 28°58'48.00"E; 710–775 m. SAM_H3184 (4 specimens): Southern margin, 22 km from Gonubie/21 km off Gqunube Estuary, 33°06'00.00"S, 28°08'17.99"E; 700–650 m. **USNM 62498 (2 specimen: holotype)**: Southern margin, 44 km from Port St. Johns/4 km from Mkweni Estuary, 31°22'59.99"S, 29°53'59.99"E; 410 m.

###### Imagery data.

**USNM 91721 (4 specimens: paratypes)**: Eastern margin, 46 km from Port Dunford/45 km off Nyoni Estuary, 29°19'12.00"S, 32°00'00.00"E; 366 m. Galathea St. 196 (2 specimens). USNM 87645 (5 specimens): Eastern margin, 146 km from Gouritsmond/152 km off Goukou Estuary, 35°40'12.00"S, 21°58'48.00"E; 424 m.

###### Description.

Corallum elongated, sub-cylindrical, mostly attached through a slender pedicel, which expands into an encrusting base. Thecal edge diverging at an angle of 7–9°. Coralla rarely straight, most examined specimens bent near base. Calice calicular with thin and fragile calicular margin. Largest specimen examined (SAM_H2841) 6.3 × 6.2 mm in CD, and 38.0 mm in H. Theca fragile; epitheca bear longitudinal ridges corresponding to septa. Spaces between epithecal ridges usually have transverse and fine striations. Corallum light brown and sometimes white.

Septa hexamerally arranged in two cycles according to the formula: S_1_ > S_2_ (12 septa). S_1_ extend towards centre of fossa with straight to slightly sinuous axial margin. S_2_ half the width of S_1_, also with slightly sinuous axial margin. Lower septal faces bear a row of spines. Fossa deep and narrow.

###### Distribution.

Regional: Southern to eastern margin of South Africa, off Cape Point extending to Cape Vidal, 80–775 m. Elsewhere: Only known from South Africa (Cairns and Zibrowius 2016).

###### Remarks.

Flabellum (F.) leptoconus was well described by Cairns and Zibrowius (2016) and examined specimens add no taxonomic information, apart from the row of spines present on the septal faces deep in fossa.

##### Flabellum (Flabellum) pavoninum

Taxon classificationAnimaliaScleractiniaFlabellidae

Lesson, 1831

9FBF7D6D-785E-58AF-8AC6-18B9C3E61C7A

Fig. 11E–H

Flabellum pavoninum Lesson, 1831: 2. –Gardiner 1902: 123–125, pl. 4, figs 18–12. – Gardiner 1904: 98. –Gardiner and Waugh 1938: 174. –Zibrowius et al. 1975: 98–99, pl. 2, figs D–E. –Boshoff 1981: 35. –Cairns 1984: 20. –Zibrowius and Grygier 1985: 122.
Flabellum coalitum von Marenzeller, 1888: 48–49. –Cairns 1989a: 46, 50, fig. 24E, F, I–L.
Flabellum thourarsii . –Boshoff 1981: 34.
Flabellum sp. 6. –Cairns 1989a: 63, 67, fig. 2.
Flabellum (Flabellum) pavoninum . –Cairns 1989a: 46–50, figs 23G–L, fig. 24A–D. –Cairns and Keller 1993: 263–264. –Cairns 1994: 70–71, pl. 30, figs G–L, pl. 31, figs A–E. –Cairns and Zibrowius 1997: 150–151, figs A–D, G, H. –Cairns 1999a: 115–116, fig. 18G–I.

###### Type locality.

Off Sandwich Islands, Hawaiian Islands; no depth given (Cairns 1989b).

###### Type material.

Five syntypes are deposited at the MNHNP (Cairns 1989a).

###### Material examined.

ORI_DIIb1 (3 specimens): Locality data unknown; 27 m. **ORI_DIIb4 (2 specimens)**: Locality data unknown. DEFF_NANSEN–INV17 (2 specimens): Eastern margin, 15 km from Scottburgh/16 km off Mahlongwana Estuary, 30°18'00.00"S, 30°54'36.00"E; 226 m. DEFF_NANSEN–INV 51 (2 specimens): Eastern margin, 39 km from Shaka’s Rock/37 km from Mdlotane Estuary, 29°34'47.99"S, 31°37'47.99"E; 130–144 m. SAMC_A073053 (1 specimen): Eastern margin, 5 km from Cape Vidal/16 km off St Lucia Estuary, 28°08'24.00"S, 32°36'24.11"E; 165 m. SAMC_A073063 (2 specimens): Eastern margin, 18 km from Cape Vidal/19 km off Mfolozi Estuary, 28°17'30.11"S, 32°34'12.00"E; 100 m. SAMC_A073135 (2 specimens): Eastern margin, 19 km from Coffee Bay/18 km off Mdumbi Estuary, 32°02'53.87"S, 29°19'41.87"E; 250–280 m. SAMC_A073171 (1 specimen): Eastern margin, 48 km from Cape Vidal/21 km from Mgobezeleni Estuary, 27°42'53.99"S, 32°40'54.11"E; 160 m. SAMC_A073213 (1 specimen): Eastern margin, 29 km from Durban/14 km off Mbokodweni Estuary, 30°06'24.12"S, 31°00'47.88"E; 160–170 m. SAMC_A073225 (2 specimens): Eastern margin, 34 km from Shaka’s Rock/off Tongati Estuary, 29°45'59.99"S, 31°25'59.99"E; 110–130 m. SAMC_A090097 (5 specimens): Eastern margin, 34 km from Shaka’s Rock/ off Tongati Estuary, 29°45'59.99"S, 31°25'59.99"E; 110–130 m. SAMC_A090098 (4 specimens): Eastern margin, 46 km from Shaka’s Rock/42 km off Mdlotane Estuary, 29°34'59.99"S, 31°41'59.99"E; 138 m. SAM_H1432 (1 specimen): Eastern margin, 2 km from Durban/8 km off Umgeni Estuary, 29°51'59.99"S, 31°00'00.00"E; 101 m. SAM_H1477 (2 specimens): Eastern margin, 19 km from Shaka’s Rock/3 km off Mdloti Estuary, 29°38'59.99"S, 31°07'59.99"E; 49 m. SAM_H3845 (8 specimens): Eastern margin, 34 km from Shaka’s Rock/ off Tongati Estuary, 29°45'59.99"S, 31°25'59.99"E; 110–130 m. SAM_H4582 (1 specimen): Eastern margin, 31 km from Shaka’s Rock/33 km from Mhlali Estuary, 29°39'48.00"S, 31°30'05.99"E; 150 m. **USNM 82134 (4 specimens)**: Eastern margin, 27 km from Durban/22 km from Beachwood Mangroves, 29°48'00.00"S, 31°16'11.99"E; 232 m.

###### Imagery data.

**BMNH 1950.1.11.30 (2 specimens)**: Locality data unknown. **Mortensen 31 (5 specimens)**: Eastern margin, off Durban, 128 m. **ZH10 (1 specimen)**: Eastern margin, 68 km from Cape Vidal/8 km off Mgobezeleni Estuary, 27°32'34.79"S, 32°43'33.59"E; 150 m.

###### Description.

Corallum flabellate, unattached, but bearing a narrow pedicel circular in profile. Calice compressed (GCD:LCD = 1.26–2.50), with smooth calicular margin. Largest specimen examined (DEFF_NANSEN–INV17) 48.3 × 19.3 mm in CD, 34.4 mm in H, and 1.3 mm in PD. Thecal faces planar but diverging in an angle between 50–70°. Thecal edges bear low and convex crest. Angle of thecal edge (excluding crest) between 93–138°. Corallum white or sometimes reddish brown.

Septa arranged in six cycles, last being incomplete, according to the formula: S_1–3_ > S_4_ > S_5_ > S_6_ (≤ 141 septa). S_1–3_ equidistant, having moderately sinuous axial margin that join columella. Upper S_1–3_ distal margins notched. S_4_ ~ ¼ the size of S_1–3_, and have sinuous axial margin that sometimes joins columella. S_5_ ½ the width of S_4_ with sinuous to dentate axial margin. S_6,_ if present, restricted to calicular margin. Septal faces bear spines perpendicular to septal margin. Fossa deep and narrow, containing a rudimentary columella.

###### Distribution.

Regional: Eastern margin of South Africa, off Coffee Bay extending towards Cape Vidal; 49–280 m. Elsewhere: Tanzania (Gardiner and Waugh 1938); Kenya (Cairns 1989b); Madagascar (Gardiner and Waugh 1938, Zibrowius et al. 1975); Maldives (Gardiner and Waugh 1938); Australia (Cairns 2004a); Vanuatu; Wallis and Futuna Islands (Cairns 1999a); New Caledonia (Kitahara and Cairns 2021); Philippines; Indonesia (Cairns and Zibrowius 1997); Hawaiian Islands; South China Sea; Japan (Cairns 1989a, 1999); 73–665 m.

###### Remarks.

Flabellum (F.) pavoninum is reported in two forms that display different GCD:LCD and thecal edge ratios. The GCD:LCD ratio range from 1.9–2.1 (as reported in the *coalitum* form) to < 2.4 (typical form), and the thecal edge from 74° (*coalitum*) to 139° (typical) (Cairns 1994). Intraspecific variation is detailed by Cairns (1989a, 1989b), who suggested that the South African representative is potentially a different species. This suspicion was later clarified by Cairns and Keller (1993) who noted that characteristics of both, the Japanese and South African representatives overlapped, therefore confirming the Indian Ocean species to be indeed *F.pavoninum*. Nonetheless, *F.pavoninum* may be mistaken with Pacific *F.arcuatile* Cairns, 1999, but can be distinguished by its lateral thecal edge.

##### Flabellum (Flabellum) politum

Taxon classificationAnimaliaScleractiniaFlabellidae

Cairns, 1989a

ADC0CF65-5FF2-5F65-A621-FF7F09605F3C

Fig. 11I, J

Flabellum pavoninum paripavoninum . –Yabe and Eguchi 1942a: 91–93 (in part: ‘Soyo Maru’ Stn. 419, pl. 5, fig. 8A–C) . –Yabe and Eguchi 1942b: 129–130 (in part: pl. 11, fig. 9A–C).
Flabellum (Flabellum) politum Cairns, 1989a: 53–54, pl. 28, figs A–F. –Cairns 1989b: 67. –Cairns 1994: 73, pl. 32, figs A–C. –Cairns and Zibrowius 1997: 153–154. –Cairns 1998: 394. –Cairns et al. 1999: 31. –Cairns 2004a: 303. –Cairns 2009: 18. –Kitahara and Cairns 2021: 186–188, figs 91E–H, 93.
Flabellum sp. 1. –Cairns 1989b: 63, 77.

###### Type locality.

Samar Sea, Philippines (12°13'15"N, 124°05'03"E); 216 m (Cairns 1989a).

###### Type material.

The holotype and fourteen paratypes are deposited at the NMNH (Cairns 1989a, 1994).

###### Material examined.

DEFF_NANSEN– INV 34 (5 specimens): Eastern margin, 32 km from Shaka’s Rock/16 km off Nhlabane Estuary, 29°43'11.99"S, 31°25'47.99"E; 184 m. DEFF_NANSEN–INV 96 (2 specimens): Eastern margin, 32 km from Richards Bay/91 km off Bulungulu Estuary, 28°40'47.99"S, 32°23'59.99"E; 20 m. SAMC_A090160 (5 specimens): Eastern margin, 10 km from Shaka’s Rock/Mhlali Estuary, 29°30'00.00"S, 31°20'23.99"E; 53–54 m.

###### Description.

Corallum flabellate, unattached, and bearing a narrow circular pedicel. Calice compressed (GCD:LCD = 2.1–2.4), with a smooth calicular margin. Largest specimen examined (SAMC_A090160) 38.9 × 16.4 mm in CD, 30.8 mm in H, and 1.3 mm in PD. Thecal faces planar and diverging in an angle between 50–70°. Angle of thecal edge (excluding crest) between 124–150°, thecal crest rounded. Corallum white with reddish brown stripes corresponding to septa, usually porcelaneous.

Septa arranged in six complete cycles according to the formula: S_1–3_ > S_4_ > S_5_ > S_6_, sometimes pairs of S_7_ present (≤ 194 septa). S_1–3_ equidistant, and joining columella with moderately sinuous axial margins. S_1–3_ upper distal edge notched. S_4_ ~ ¼ the size of S_1–3_, sometimes joining columella with sinuous axial margin. S_5_ ½ the size of S_4._ S_6_ rudimentary. S_7_, if present, also rudimentary. Septa faces with spines arranged perpendicular to septal margin. Fossa deep, narrow, with a rudimentary columella.

###### Distribution.

Regional: Eastern margin of South Africa, off Shaka’s Rock extending towards Richards Bay; 20–185 m. Elsewhere: Philippines; Indonesia (Cairns and Zibrowius 1997); South China Sea; Japan (Cairns 1989a); New Caledonia (Kitahara and Cairns 2021); Australia (Cairns 2004a); 40–717 m.

###### Remarks.

These specimens represent new records of Flabellum (F.) politum in the southwestern Indian Ocean and illustrate that it may be found in shallower waters. It is also worth noting that adult *F.politum* can be easily mistaken for juvenile Flabellum (F.) pavoninum “*coalitum*” form (Cairns and Zibrowius 1997), but may be distinguished by its smooth and porcelaneous thecal face. A full comparison of *F.politum* and other species can be found in Cairns (1989b).

##### Flabellum (Ulocyathus)

Taxon classificationAnimaliaScleractiniaFlabellidae

Sar, 1851

BB20709B-3EFF-5B1F-BB99-9AC5B73FB4C8

###### Diagnosis.

*Flabellum* having a jagged or lacerate calicular edge.

###### Type species.

*Ulocyathusarcticus* Sars, 1851 (= *Flabellummacandrewi* Gray, 1849), monotypy.

##### Flabellum (Ulocyathus) alabastrum

Taxon classificationAnimaliaScleractiniaFlabellidae

Moseley, 1873

C614C031-575F-5FE8-B173-DD7EA1187EC4

Fig. 11K, L.

Flabellum alabastrum Moseley in Thompson, 1873: 403, fig. 2. –Moseley 1881: 169, pl. 7, figs 1, 1A, B, 2, 2A, B, pl. 16, fig. 11. –Zibrowius 1980: 148, pl. 77 A–J. –Cairns 1981: 6. –Zibrowius 1985: 318. –Zibrowius and Gili 1990: 38. pl. 2S, T.
Flabellum minus Duncan 1878: 243, pl. 45, figs 10–13.
Flabellum (Ulocyathus) alabastrum . –Cairns 1989: 54.

###### Type locality.

Azores (HMS ‘Challenger’ stn. 73: 37°26'00"N, 25°13'00"W); 1829 m (Zibrowius 1980).

###### Type material.

Syntypes are deposited at the BMNH (Zibrowius 1980).

###### Material examined.

SAMC_A090102 (1 specimen): Southern margin, 125 km from Agulhas/134 km off Ratels Estuary, 35°56'37.38"S, 20°02'27.30"E; 168 m.

###### Description.

Corallum unattached with a narrow and circular pedicel. Calice compressed (GCD:LCD = 1.4), with a highly jagged calicular margin. Largest specimen examined 28.3 × 19.8 mm in CD, 22.1 mm in H, and 2.2 mm in PD. Thecal faces planar and diverging at an angle of 110°. Thecal edges crested and diverging in an angle of 150° (excluding crests). Thecal crest sinuous. C_1–3_ well developed and extending from calicular margin towards base. Thin and faint growth ridges, corresponding to higher cycle septa (S_4–5_) present. Corallum predominantly white, but pedicel and intercostal striae reddish brown.

Septa arranged in five cycles, last being incomplete, according to the formula: S_1–3_ > S_4_ > S_5_ (≤ 80 septa). S_1–3_ equidistant and join columella with moderately sinuous axial margins. S_4_ ~ ½ the size of S_1–3_, and bear sinuous axial margin. S_5_ rudimentary. Septal faces bear small spines sparsely arranged along the septal margins. Fossa moderately deep, narrow, and containing a rudimentary columella composed by fusion of lower axial margin of larger septa.

###### Distribution.

Regional: Western to southern margin of South Africa, off Alexander Bay extending towards Algulhas; 168–1089 m. Elsewhere: Walvis Ridge (Zibrowius and Gili 1990); Hebrides; Azores; Gulf of Guinea, Georgia towards Davis Strait (Cairns 1981); Bahamas (Zibrowius 1980); 357–2000 m.

###### Remarks.

Flabellum (U.) alabastrum is one of the four flabellid species with a constricted corallum and may be mistaken with *F.lowekeyesi* Squires & Ralph, 1965. However, the two species differ in having a shorter, crested lateral edges (LEL: H = 0.3–0.5 vs. 0.9 for *F.alabastrum*), and a taller corallum (GCD: H = 1.0–1.3 vs. 1.5 for *F.alabastrum*)(Cairns 1989a, 1995). Flabellum (U.) alabastrum is historically only known from the Atlantic, thus the current South African record extends the species distribution to the Indian Ocean (east of Cape Point). Furthermore, the examined specimens confirm that the *F.alabastrum* is found 189 m shallower than previously known.

##### Flabellum (Ulocyathus) lowekeyesi

Taxon classificationAnimaliaScleractiniaFlabellidae

Squires & Ralph, 1965

6F85525C-C4BC-53F5-A08D-16EA68A64CF7

Fig. 11M, N

Flabellum lowekeyesi Squires & Ralph, 1965: 259–261, figs 1, 2. –Squires and Keyes 1967: 27, pl. 6, figs 1, 2.
Flabellum (Ulocyathus) lowekeyesi . –Cairns 1989a: 54. –Cairns and Keller 1993: 262, fig. 10 D, E. –Cairns 1995: 100–101, pl. 32, figs G–I. –Cairns 2004a: 304. –Kitahara and Cairns 2021: 200–202, figs 100I–L, 101.

###### Type locality.

Off Cape Brett, New Zealand (stn. 29: 26 miles off Cape Brett); 732 m (Squires and Ralph 1965).

###### Type material.

The holotype is deposited at the MoNZ and one paratype at the NMNH (Cairns 1995).

###### Material examined.

SAM_H1695 (1 specimen): Eastern margin, 49 km south of Ponta Do Ouro/37 km off Kosi Bay Estuary, 27°17'30.00"S, 32°54'59.99"E; 720–780 m. SAM_H1696 (1 specimen): Eastern margin, 44 km south of Ponta Do Ouro/32 km off Kosi Bay Estuary, 27°14'47.99"S, 32°54'35.99"E; 700 m. SAM_H2814 (2 specimens): Eastern margin, 36 km from St. Lucia/34 km off Mfolozi Estuary, 28°37'47.99"S, 32°38'29.99"E; 1200–1000 m. SAM_H3095 (1 specimen): Eastern margin, 17 km from St. Lucia Estuary/16 km off Mfolozi Estuary, 28°21'53.99"S, 32°34'36.00"E; 775–825 m.

###### Description.

Corallum unattached and bearing a narrow and circular pedicel. Calice compressed (GCD:LCD = 1.3), with highly lacerated calicular margin. Largest specimen examined (SAM_H2814) 27.6 × 20.9 mm in CD, 30.1 mm in H, and 2.8 mm in PD. Thecal faces straight and diverging in an angle between 55–73°. Thecal edge short and continuously crested. Angle of thecal edges (excluding crest) between 160–190°. C_1–3_ well developed and extending from calicular margin towards base. Theca bear chevron-shaped growth lines, and fine granulation. Corallum predominantly white, but pedicel, intercostal striae, and axial septal faces reddish brown.

Septa hexamerally arranged in a variable number of cycles. Examined specimens have five or six cycles, last being incomplete, according to the formula: S_1–3_ > S_4_ > S_5_ > S_6_ (88–110 septa). S_1–3_ equidistant and joining columella deep in fossa with straight and thickened axial margins. S_4_ ~ ^1^/_3_ the size of S_1–3_, and also have straight axial margin. S_5_ rudimentary in specimens with fifth cycle incomplete, but ½ the width of S_4_ in specimens having sixth cycle incomplete. S_6_ rudimentary. Septal faces finely granular. Fossa deep, narrow, with a trabecular columella following the shape of the curved corallum.

###### Distribution.

Regional: Eastern margin of South Africa, off St. Lucia extending towards Kosi Bay Estuary (49 km south of Ponta Do Ouro: Mozambique); 700–1200 m. Elsewhere: Off Mozambique; Mascarene Plateau; Madagascar (Cairns and Keller 1993); New Caledonia (Kitahara and Cairns 2021); Tasmania; New Zealand (Squires and Ralph 1965; Cairns 1995, 2004a); 278–1100 m.

###### Remarks.

Specimens represent at range extension, from Mozambique to South African territory. Flabellum (U.) lowekeyesi closely resembles *F.messum* Alcock, 1902a but differs in its greater thecal edge angle, tendency of S_4_ axial margin to fuse to adjacent septa, larger white coralla, and smaller pedicel (Cairns 1989a, 1995).

##### Javania

Taxon classificationAnimaliaScleractiniaFlabellidae

Duncan, 1876

C905AE62-22E8-5091-8BC9-ECC18079F14D

###### Diagnosis.

Corallum solitary, sub-cylindrical to turbinate, and attached by a pedicel that is strongly reinforced with numerous layers of dense stereome (tectura). Three to five cycles of highly exsert septa present, resulting in a lacerate calicular edge. Pali absent. Columella rudimentary or absent.

###### Type species.

*Javaniainsignis* Duncan, 1876, by monotypy.

##### Javania antarctica

Taxon classificationAnimaliaScleractiniaFlabellidae

(Gravier, 1914)

58518126-4579-56C2-9C14-1029D5A76CC4

Fig. 11O, P

Desmophyllum antarcticum Gravier, 1914: 236–238. –Cairns 1914b: 122–125, pl. 1, figs 1–3 (in part).
Javania antarctica . –Cairns 1982: 48, 50, pl. 15, figs 1–4. –Cairns et al. 1999: 31. –Cairns 2004b: 8. –Cairns 2009: 20. –Cairns and Polonio 2013: 79, figs 4G, H, J, 18. –Kitahara and Cairns 2021: 204–205, figs 102E–G, 103.

###### Type locality.

Off Anvers Island, Antarctica (RV ‘Pourquoi-Pas’ (?) stn. 8: 64°50'00"S, 63°30'00"W); 53 m (Cairns 1982).

###### Type material.

The syntype is deposited at the MNHNP (Cairns 1982).

###### Material examined.

SAMC_A090150 (3 specimens): Southern margin, 116 km from Gouritsmond/km off Goukamma Estuary, 35°07'11.27"S, 23°02'41.75"E; 333 m.

###### Description.

Corallum trochoid to ceratoid, straight to slightly curved, and attached through a slender but reinforced pedicel, which expands into an encrusting base. Calice elliptical (GCD:LCD = 1.4), with a thin and serrated calicular margin, which is usually broken. Largest specimen examined 27.9 × 19.4 mm in CD, and 46.2 mm in H. Theca thin, glistening, and porcelaneous. Faint costae correspond to all septa, and extend from calicular margin to base. Transversely arranged, chevron-shaped growth lines more distinctive at major septa. Corallum white.

Septa hexamerally arranged in five cycles according to the formula: S_1–2_ > S_3_ > S_4_ > S_5_ (96 septa). Septa of all examined specimens damaged at calicular margin, therefore unable to observe exsertness. S_1–2_ almost joining opposite septa with straight to slightly sinuous, thickened axial margins. S_3_ slightly less wide and less sinuous than S_1–2._ S_4_ ~ ¼ smaller than S_3,_ with similarly or slightly more sinuous axial margin. S_5_ rudimentary, but bearing the most sinuous axial margin. Septal faces finely granulated with pointed spines. Fossa deep.

###### Distribution.

Regional: Southern margin of South Africa, Gouritsmond; 333m. Elsewhere: Antarctica (Cairns 1982); Argentina (Cairns and Polonio 2013); New Caledonia (Kitahara and Cairns 2021) 53–1626 m.

###### Remarks.

Among all congeners, *Javaniaantarctica* most closely resembles *J. lamprotichum* (Moseley, 1880) but differs in corallum pigmentation, shape, and height. *Javaniaantarctica* is also similar to *J.insignis* in having five cycles of septa, but can be distinguished by its delicate theca, thin calicular margin, and less exsert septa. Specimens examined herein represent a new record for South Africa.

##### Javania insignis

Taxon classificationAnimaliaScleractiniaFlabellidae

Duncan, 1876

5F5DAD83-156A-5C60-BB53-20A41A38FC4E

Fig. 12A, B

Javania insignis Duncan, 1876: 435, pl. 39, figs 11–13. –von Marenzeller 1907: 23, pl. 2, fig. 6. –Yabe and Eguchi 1932e: 388. –Zibrowius 1974c: 8–9, pl. 1, figs 1–6. –Scheer and Pillai 1983: 165–166, pl. 37, figs 9–12. –Cairns 1984: 23, pl. 4, figs F–H. –Cairns 1989a: 77–78, pl. 40, figs D–E, G–H, J–K. –Cairns 1994: 80, pl. 34, figs I–K. –Cairns and Keller 1993: 272. –Tachikawa 2005: 10, pl. 4, figs C–D. –Cairns and Zibrowius 1997: 163–164. –Cairns et al. 1999: 31. –Cairns 2004a: 304. –Cairns 2004b: 8. –Cairns 2009: 20. –Kitahara and Cairns 2021: 214–215, figs 103, 108D–F.
Flabellum weberi Alcock, 1902a: 107. 94.
Desmophyllum cf. insigne . –Yabe and Eguchi 1942b: 115, pl. 9, figs 5, 6.
Desmophyllum insignis . –Eguchi 1965a: 290. –Eguchi 1968: C41–C42, pl. C9, figs 4–9. –Song 1982: 136, pl. 2, figs 5, 6.
Flabellum rubrum . –Boshoff 1981: 35.

###### Type locality.

Off Owase, Japan (34°13'00'N, 136°13'00'E); 88 m (Cairns 1994).

###### Type material.

The holotype is deposited at the NHMUK (Cairns 1994).

###### Material examined.

DEFF_CCS2D17–INV116 (1 specimen): Western margin, 53 km from Saldanha/80 km off Berg River V Estuary, 33°06'35.77"S, 17°23'01.26"E; 410 m. ORI_DIIb5 (1 specimen): Southern margin, other locality data unknown. SAMC_A073082 (2 specimens): Eastern margin, 31 km from Richards Bay/46 km off Mlalazi Estuary, 29°00'54.00"S, 32°15'35.99"E; 500 m. SAMC_A073083 (1 specimen): Eastern margin, 28 km from Richards Bay/40 km off Mlalazi Estuary, 29°00'54.00"S, 32°12'06.12"E; 215 m. SAMC_A073096 (3 specimens): Locality data unknown. SAMC_A73218 (1 specimen): Eastern margin, 42 km south of Ponta Do Ouro/27 km from Kosi Bay Estuary, 27°13'30.00"S, 32°49'30.00"E; 74 m. SAMC_A90116 (1 specimen): Eastern margin, 33 km from Port Dunford/37 km from Mlalazi Estuary, 29°08'59.99"S, 32°05'24.00"E; 85 m.

###### Description.

Corallum ceratoid, robust, expanding into a flared calice, and attached through a slender reinforced pedicel, which expands into an encrusting base. Calice elliptical (GCD:LCD = 1.40–1.70), with a thick and jagged calicular margin. Largest specimen examined (SAMC_A90116) 25.6 × 15.9 mm in CD, and 25.5 mm in H. Theca robust and smooth. C_1–3_ restricted to upper ^1^/_3_ of coralla. Corallum white.

Septa hexamerally arranged in five cycles according to the formula: S_1_ > S_2_ > S_3_ > S_4_ > S_5_ (96 septa). S_1_ most exsert, with straight and vertical axial margin. S_2_ smaller, slightly less exsert than S_1_, and have slightly sinuous axial margin. S_3_ as exsert as S_2_ but only ½ the width of S_2._ S_3_ axial edge as sinuous as that from S_2._ S_4_ non-exsert, ¼ the size of S_3_, and bear moderately sinuous axial margin. S_5_ rudimentary, but have the most sinuous axial margin. All septal faces finely granulated with blunt spines. Fossa deep.

###### Distribution.

Regional: Eastern margin of South Africa, off Richards Bay extending towards Kosi Bay Estuary (42 km south of Ponta Do Ouro: Mozambique); 74–500 m. Elsewhere: Mozambique; Madagascar (Cairns and Keller 1993); Red Sea (von Marenzeller 1907); Reunion (Zibrowius and Grygier 1985); Hawaiian Islands (Cairns 1984); Japan (Cairns 1989b); Philippines; Indonesia (Cairns and Zibrowius 1997); New Caledonia (Kitahara and Cairns 2021); 46–1050 m.

###### Remarks.

*Javaniainsignis* was first collected off the South African margin through the UCT Ecological Survey, but identified as *Flabellumrubrum* (ORI_ DIIb5) by Boshoff (1981). Subsequently, Cairns and Keller (1993) reported on the occurrence of this species within the region and their account, therefore, represents the first reliable record of *J.insignis* within South African territory. *Javaniainsignis* is compared with *J. antarctica* in the species account above.

**Figure 12.**
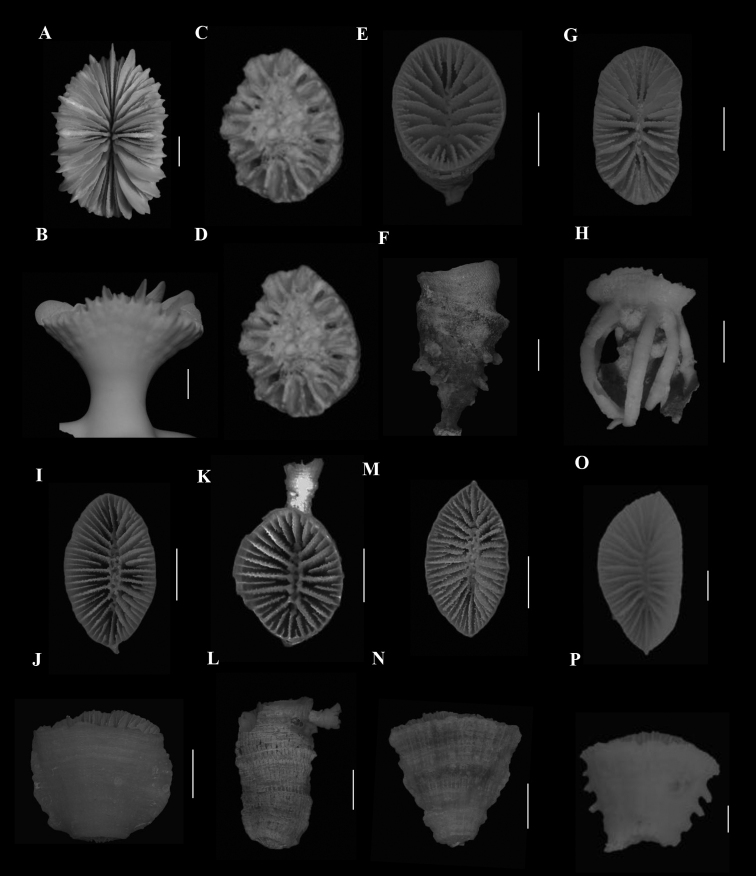
**A**, **B***Javaniainsignis* (SAMC_A090116, off Port Dunford, 85 m) **A** calicular view **B** lateral view **C**, **D***Placotrochidesscaphula* (USNM 91772, off Scottburgh, 1360 m) **C** calicular view **D** calicular view **E**, **H***Rhizotrochustypus***E**, **F** (SAMC_A090094, off Coffee Bay, 160 m) **E** calicular view **F** lateral view **G**, **H** (SCD39B, off Coffee Bay, 160 m) **G** calicular view **H** lateral view **I**, **J***Truncatoflabellumformosum* (SAM_H1389, off Shaka’s Rock, 183 m) **I** calicular view **J** lateral view **K**, **L***Truncatoflabellumgardineri* (SAM_H3847, off Shaka’s Rock, 138 m) **K** calicular view **L** lateral view **M**, **N***Truncatoflabelluminconstans* (SAM_H1241, off Kei Mouth, 66 m) **M** calicular view **N** lateral view **O**, **P***Truncatoflabellummultispinosum* (SAMC_A087450, locality data unknown) **O** calicular view **P** lateral view. Scale bars: 10 mm.

##### Placotrochides

Taxon classificationAnimaliaScleractiniaFlabellidae

Alcock, 1902

5E60C6A3-CF0B-5365-9028-BC99080B6E63

###### Diagnosis.

Corallum solitary and compressed-cylindrical. Transverse division present, resulting in an anthocyathus with a basal scar almost as large as calicular diameter. Thecal spines absent. Three to four cycles of non-exsert septa. Calicular edge smooth. Columella well developed, trabecular.

###### Type species.

*Placotrochidesscaphula* Alcock, 1902c, by subsequent designation (Wells 1936).

##### Placotrochides scaphula

Taxon classificationAnimaliaScleractiniaFlabellidae

Alcock, 1902

560D7877-98B2-5598-9812-887588039A58

Fig. 12C, D

Placotrochides scaphula Alcock, 1902c: 34, pl. 4, figs 32, 32A. –Wells 1936:124. –Zibrowius 1974d: 23. –Zibrowius 1980: 159. –Cairns 1989b: 78–79, pl. 40–41, figs 1 and A–E. –Cairns and Parker 1992: 48–49, figs 15H–I. –Cairns and Keller 1993: 272–273, pl. 12, figs D, G. –Cairns 1994: 79–80, pl. 34, figs F–H. –Cairns and Zibrowius 1997: 174. –Cairns and Kithara 2012: pl. 20, figs N–O. –Cairns 2016: 40, fig. 11C. –Li et al. 2017: 149. –Kitahara and Cairns 2021: 222, figs 110, 111A–C.

###### Type locality.

Off southeastern Celebes, Flores Sea, 462 m (Cairns 1989b).

###### Type material.

The holotype is deposited at the ZMA (Cairns 1989b).

###### Material examined.

**USNM 91772 (3 specimens)**: Eastern margin, 28 km from Scottburgh/21 km off Mkomazi Estuary, 30°11'59.99"S, 32°01'00.00"E; 1360 m.

###### Imagery data.

MN_SM246 (4 specimens): Eastern margin, 39 km from Port St. Johns/32 km off Hluleka Estuary; 1660–1640 m.

###### Description.

Corallum compressed, cylindrical and robust. Thecal faces parallel. Calice elliptical (GCD:LCD = 1.2–1.3), with a smooth calicular margin. Largest specimen examined 6.4 × 5.0 mm in CD, and 3 mm in H. Costae wide and flat. Intercostal striae narrow. Reported to bear chevron-shape growth lines peaking at each costae and a large basal scar. However, examined specimens are eroded and these features are not visible. Corallum white.

Septa hexamerally arranged in four cycles according to the formula: S_1–2_ > S_3_ > S_4_ (42 septa). S_1–2_ have vertical and slightly sinuous axial margin. S_3_ dimorphic in development: when flanked by a pair of S_4_, S_3_ being ¼ smaller than S_1–2_, and bear smooth axial margin that fuse to columella. However, unflanked S_3_ being ¾ smaller than S_1–2_, and bear finely serrated axial margin. S_4_ rudimentary. All septa are non-exsert and widely spaced. Fossa deep and elongated, containing a trabecular columella.

###### Distribution.

Regional: Eastern margin of South Africa, off Scottburgh extending towards Port St. Johns; 930–1660 m. Elsewhere: Madagascar (Cairns and Keller 1993); Australia (Cairns and Parker 1992); Japan (Cairns 1994); Philippines; Indonesia (Alcock 1902a, 1902b; Cairns and Zibrowius 1997); New Caledonia (Kitahara and Cairns 2021); 462–1628 m.

###### Remarks.

Apart from *Placotrochidesscaphula*, there are four other *Placotrochides* known to date (*P.cylindrica* Cairns, 2004, *P.frustum* Cairns, 1979, *P.minuta* Cairns, 2004, and *P.yapensis* Li, Yu-Rong & Xu, 2017) , of which *P.scaphula* most resembles *P.minuta* but can be distinguished by its larger corallum and higher number of septa at the same calicular diameter (Cairns 2004a). The large corallum size (< 12 mm) and septa arrangement are features *P.scaphula* shares with *P.yapensis*. These two species can be separated by the GCD:LCD ratio (1.4–2.1 in *P.scaphula* vs. 1.1–1.2 in *P.yapensis*) and also by the number of septa (≤ 42 in *P.scaphula* vs. 48 in *P.yapensis)*. *Placotrochidesscaphula* is well described by Cairns (1989b, 1994), and there are no new South African records subsequent to Cairns and Keller (1993), apart from the imagery records represented herein (MN_SM246).

##### Rhizotrochus

Taxon classificationAnimaliaScleractiniaFlabellidae

Milne-Edwards & Haime, 1848a

A705817E-0881-5F32-A516-4782F5849BE6

###### Diagnosis.

Corallum ceratoid to turbinate or compressed. Transverse division absent. Pedicel small and not reinforced by stereome; however, two to twenty slender hollow rootlets anchor corallum base. Thecal spines absent. Three to six cycles of non-exsert septa, the lower septal cycle being usually highly concave near calicular edge. Pali absent. Columella rudimentary.

###### Type species.

*Rhizotrochustypus* Milne-Edwards & Haime, 1848, by monotypy.

##### Rhizotrochus typus

Taxon classificationAnimaliaScleractiniaFlabellidae

Milne-Edwards & Haime, 1848

9E10AF89-FD8E-51ED-B366-2CAEBE945B64

Fig. 12E–H

Rhizotrochus typus Milne-Edwards & Haime, 1848a 282, pl. 8, fig. 16. –Pourtalès 1871: 13. –Studer 1881: 28. –Moseley 1881: 131. –von Marenzeller 1907: 23, pl. 2, fig. 5. –Yabe and Sugiyama 1936: 346–348, figs 3, 3A. –Cairns 1989a: 79–81, pl. 41, figs F, J. –Cairns 1994 81, pl. 35, figs A–C, pl. 40, figs H, I. –Cairns and Zibrowius 1997: 161, figs 22D, E. –Cairns 1999a: 127, fig. 22A. –Cairns et al. 1999: 31. –Tachikawa 2005: 10–11, pl. 4, figs E–H. –Cairns 2009: 21. –Kitahara and Cairns 2021: 234–236, figs 120F–G, 121.
Flabellum rubrum . –Gardiner 1902a: 125–152, pl. 4, fig. 34. –Gardiner 1902b: 464–471.
Flabellum harmeri . –Boshoff 1981: 35.
Monomyces sp. –Boshoff 1981: 35.

###### Type locality.

Singapore, depth unknown (Cairns 1994).

###### Type material.

Two syntypes are deposited at the MNHNP (Cairns 1994).

###### Material examined.

ORI_DIIb3 (in part: 1 specimen): Locality data unknown, 27 m. ORI_DIIc1 (4 specimens), SAMC_A072990 (1 specimen): Locality data unknown. SAMC_A073048 (1 specimen): Locality data unknown; SAMC_A073194 (2 specimens): Southern margin, 5 km from Gonubie/3 km off Gqunube Estuary, 32°57'11.87"S, 28°02'48.12"E; 30 m. SAMC_A073247 (3 specimens): Eastern margin, 34 km from Coffee Bay/7 km off Ntlonyane Estuary, 32°15'11.99"S, 28°57'41.99"E; 160 m. SAM_A90094 (2 specimens): Eastern margin, 34 km from Coffee Bay/16 km from Ntlonyane Estuary, 32°16'59.99"S, 29°03'59.99"E; 160 m. UCTES_SCD39B (3 specimens): Eastern margin, 34 km from Coffee Bay/16 km from Ntlonyane Estuary, 32°16'59.99"S, 29°03'59.99"E; 160 m

###### Imagery data.

BMNH 1939.7.20.816–833 (1 specimen), BMNH 1950.1.10.34 (1 specimen), BMNH 1950.10.97–102 (1 specimen), BMNH 1950.1.11.63 (1 specimen): Cape of Good Hope, other locality data unknown.

###### Description.

Corallum conical, robust, and attached to substrate by a slender and non-reinforced pedicel (< 3 mm in D). Calice irregularly elliptical (GCD:LCD = 1.2–1.5), with thin, smooth, and flared calicular margin. Largest specimen examined (SAMC_A072990) 14.4 × 9.8 mm in CD, and 21.1 mm in H. Theca thin and usually encrusted, lower theca bears numerous (> 5) hollow rootlets that improve corallum anchoring (rootlets of examined specimens broken). Costae equally wide but poorly developed. Intercostal striae shallow. Transversal chevron-shaped growth lines present from calicular margin towards pedicel. Corallum predominantly white, becoming light-beige from lower theca towards base.

Septa hexamerally arranged in six cycles, last being incomplete, according to the formula: S_1–2_ > S_3_ > S_4_ > S_5_ > S_6_. However, specimens examined also have fifth cycle incomplete (totalling 78–89 septa). S_1–2_ equal in width and have straight and vertical axial margin, which become slightly sinuous deeper in the fossa. Higher cycle septa (S_3–5_) progressively smaller, and bearing slightly sinuous axial margins. S_6_ if present rudimentary, but, if absent, then S_5_ rudimentary. Septal faces finely granulated. Fossa deep, with rudimentary or absent columella.

###### Distribution.

Regional: Southern and eastern margin of South Africa, off Gonubie extending Coffee Bay; 30–160 m. Elsewhere: Andaman Islands; Bay of Bengal (Alcock 1893); Red Sea (von Marenzeller 1907); Japan (Cairns 1989b, 1994); South China Sea; Philippines; Indonesia (Cairns and Zibrowius 1997); Vanuatu (Cairns 1999a); New Caledonia (Kitahara and Cairns 2021); 20–1048 m.

###### Remarks.

Although *Rhizotrochustypus* was collected off the South African margin (Cape of Good Hope) in the early 20^th^ century, it was incorrectly identified by Gardiner (1902a, 1902b) as *Flabellumrubrum*. Subsequently, Boshoff (1981) misidentified *R.typus* as two other flabellids (*F.harmeri* [ORI_DIIb3] and *Monomyces* sp. [ORI_DIIc1]). *Rhizotrochus* has long been synonymiszed with *Monomyces*, however historical accounts have outlined the difference in the number and placement/pattern of their rootlets, in which *Rhizotrochus* has > 2 rootlets that are not fused to pedicel as compare with *Monomyces* Ehrenberg, 1834, which displays < 2 rootlets fused to the pedicel (Cairns 1989a). Despite the examined specimens having broken rootlets (thus not showing whether they join pedicel or not), we herein provide the first reliable record for the species within South African localities by use of the number of rootlets (> 5) observed.

##### Truncatoflabellum

Taxon classificationAnimaliaScleractiniaFlabellidae

Cairns, 1989

72DAF2A2-5859-5EE1-8393-54076650E843

###### Diagnosis.

Corallum solitary and highly compressed. Asexual reproduction by transverse division, resulting in a free anthocyathus budded from a basal anthocaulus. Calicular edge smooth to highly serrate. Thecal edge spines or crests common. Pali absent. Columella rudimentary.

###### Type species.

*Euphylliaspheniscus* Dana, 1846, by original designation.

##### Truncatoflabellum formosum

Taxon classificationAnimaliaScleractiniaFlabellidae

Cairns, 1989

2A96AF46-E7D2-53B1-BA04-113517E92A32

Fig. 12I, J

Flabellum rubrum . –Faustino 1927: 53 (in part: ‘Albatross’ Stns 5265 and 5658). –Yabe and Eguchi 1942a: 96–98 (in part: pl. 8, fig. 14).
Truncatoflabellum formosum Cairns, 1989a: 69–70, pl. 35, figs J, K, pl. 36, figs A, B (in part: not ‘Albatross’ stns. 5137, 5484, 5162, and 5483). –Cairns and Keller 1993: 265, figs 10I, 11A. –Cairns 1994: 77, pl. 33, figs G–H. –Cairns and Zibrowius 1997: 169–170. –Cairns 1998: 396. –Cairns et al. 1999: 31. –Cairns 2004a: 266, 309. –Cairns 2016: 35, fig. 10B. –Kitahara and Cairns 2021: 244–246, figs 126E–H, 127.
Truncatoflabellum sp. nov. –Cairns 1989a: 73, pl. 38, figs G–H.

###### Type locality.

Off Mindanao, Philippines (RV ‘Albatross’ stn. 5249: 7°06'06"N, 125°40'08"E); 42 m (Cairns 1989a).

###### Type material.

The holotype is deposited at the BMNH (Cairns 1989a).

###### Material examined.

SAMC_A073104 (1 specimen): Eastern margin, 18 km from Cape Vidal/20 km off Mfolozi Brak Estuary, 28°17'23.99"S, 32°34'36.12"E; 198 m. SAMC_A073167 (11 specimen): Eastern margin, 18 km from St. Lucia Estuary/15 km off Mfolozi Estuary, 28°31'48.00"S, 32°26'06.00"E; 160–180 m. SAMC_A073176 (1 specimen): Eastern margin, 35 km from Durban/26 km off Mbokodweni Estuary, 30°07'59.99"S, 31°08'59.99"E; 150 m. SAMC_A073181 (4 specimens): Eastern margin, 11 km from Port St. Johns/10 km off Bulolo Estuary, 31°43'54.12"S, 29°32'12.11"E; 190 m. SAMC_A073212 (1 specimen): Eastern margin, 5 km from Cape Vidal/16 km off St Lucia Estuary, 28°07'05.88"S, 32°36'35.99"E; 145 m. SAMC_A073266 (1 specimen): Southern margin, 2 km from Stilbaai/1 km off Goukou Estuary, 34°22'55.26"S, 21°25'25.49"E; 88 m. SAMC_A090100 (3 specimens): Eastern margin, 34 km from Shaka’s Rock/ off Tongati Estuary, 29°45'59.99"S, 31°25'59.99"E; 110–130 m. SAM_H784 (48 specimens): Eastern margin, 19 km from Shaka’s Rock/3 km off Mdloti Estuary, 29°38'59.99"S, 31°07'59.99"E; 71–73 m. SAM_H835 (1 specimen): Southern margin, 6 km from Kenton On Sea/5 km off Boknes Estuary, 33°43'07.59"S, 26°37'37.95"E; 90 m. SAM_H1241 (6 specimens): Eastern margin, 2 km from Kei Mouth/27 km off Groot Berg Estuary, 32°40'33.99"S, 28°22'50.99"E; 66 m. SAM_H1389 (1 specimen): Eastern margin, 19 km from Shaka’s Rock/3 km off Mdloti Estuary, 29°38'59.99"S, 31°07'59.99"E; 183 m. SAM_H1399 (3 specimens): Eastern margin, 39 km from Mtunzini/8 km from Zinkwasi Estuary, 29°12'59.99"S, 31°30'00.00"E; 73 m. SAM_H1437 (45 specimens): Eastern margin, 2 km from Durban/8 km off Umgeni Estuary, 29°52'00.00"S, 31°00'00.00"E; 99 m. SAM_H1442 (1 specimen): Eastern margin, 6 km from Durban/9 km from Umgeni Estuary, 29°52'59.99"S, 31°03'04.99"E; 86 m. SAM_H1452 (1 specimen): Southern margin, 113 km from Gonubie/112 km off Gqunube Estuary, 33°43'00.00"S, 28°46'59.99"E; 90 m. SAM_H1456 (1 specimen): Eastern margin, 9 km from Shaka’s Rock/2 km off Tongati Estuary, 29°34'00.00"S, 31°10'59.99"E; 66 m. SAM_H1698 (1 specimen): Eastern margin, 18 km from Cape Vidal/27 km off Mfolozi Estuary, 28°16'18.00"S, 32°38'48.00"E; 670 m. SAM_H3096 (10 specimens): Eastern margin, 2 km from Durban/8 km off Umgeni Estuary, 29°51'59.99"S, 31°00'00.00"E; 101 m. SAM_H3097 (1 specimen): Southern margin, 14 km from Cape Padrone/26 km from Boknes Estuary, 33°45'59.99"S, 26°18'59.99"E; 115 m. SAM_H3846 (1 specimen): Eastern margin, 34 km from Shaka’s Rock/ off Tongati Estuary, 29°45'59.99"S, 31°25'59.99"E; 110–130 m. USNM 91758 (1 specimen): Eastern margin, 5 km from Cape Vidal/16 km off St Lucia Estuary, 28°07'05.88"S, 32°36'35.99"E; 145 m.

###### Description.

Corallum (anthocyathus) medium to large, compressed conical, with a small open base. Calice elliptical (GCD:LCD = 1.2–2.5), with smooth and slightly arched calicular margin. Basal scar 4.0 × 3.0 mm in diameter, with 12 complete septa originating from scar. Largest specimen examined (SAM_H3096) 25.0 × 10.0 mm in CD, and 10 mm in H. Theca glistening, may be encrusted or smooth, with longitudinal striae, and transverse corrugations. Thecal faces carinate, with angle of 45°. Anthocyathi have one to three pairs of thecal spines (SAMC_A090100). Thecal spines cylindrical in profile. Corallum white, with theca sometimes reddish brown.

Septa arranged in three size classes according to the formula: 20:20:38–40 (≤ 80 septa). Primary septa arched and concave at calicular margin, but have vertical and slightly sinuous axial margin that extend toward columella. Secondary septa ½ the width of primaries, being finely dentate near calicular margin and bearing moderately sinuous axial margin. Tertiary rudimentary. All septa non-exsert, but primaries may be slightly exsert in juvenile specimens. Septal faces bear blunt granules obliquely aligned in rows along septal margin. Fossa moderately deep with a rudimentary columella formed by lower axial margin of primary septa.

###### Distribution.

Regional: Southern to eastern margin of South Africa, off Stilbaai extending towards Cape Vidal; 55–670 m. Elsewhere: Mozambique (Cairns and Keller 1993); Australia (Cairns 2004a); Philippines (Cairns 1989a); Indonesia (Cairns and Zibrowius 1997); New Caledonia (Kitahara and Cairns 2021); Japan (Cairns 1989a, 1999); 42–993 m.

###### Remarks.

*Truncatoflabellumformosum* superficially resembles *T.multispinosum* Cairns in Cairns and Keller 1993 in having a medium to large corallum, numerous septa, not rounded thecal margin, and having thecal spines. *T.formosum* can be differentiated in having less (≤ 2) carinate thecal spines. The species was first reported in South African territory by Cairns and Keller (1993), off the eastern margin of South Africa, and the material examined here extends regional distribution towards Still Bay (off the southern margin).

##### Truncatoflabellum gardineri

Taxon classificationAnimaliaScleractiniaFlabellidae

Cairns in Cairns & Keller, 1993

8248C288-DB16-5EFE-887C-3757B5D9CAA4

Fig. 12M, N

Truncatoflabellum gardineri Cairns in Cairns & Keller, 1993: 266, pl.11, figs B–D. –Cairns 1994: 78–79. Pl. 34, figs A, B. –Cairns 2016: 37, fig. 10D.

###### Type locality.

Off Durban area, South Africa (RV ‘Anton Bruun’ stn. 390S: 29°35'00"S, 31°42'00"E); 138 m (Cairns and Keller 1993).

###### Type material.

The holotype and most paratypes are deposited at NMNH, whilst 3 paratypes are at the SAM (Cairns and Keller 1993; Cairns 1994).

###### Material examined.

SAM_H3847 (1 specimen): Eastern margin, 46 km from Shaka’s Rock/42 km off Mdlotane Estuary, 29°34'59.99"S, 31°42'00.00"E; 138 m. **SAM_H4577 (3 specimens: paratypes)**: 26 km from Port St. Johns/off Bulolo Estuary, 29°34'47.99"S, 31°41'59.99"E; 138 m. **USNM 91736 (1 specimen: holotype)**: Eastern margin, 26 km from Port St. Johns/off Bulolo Estuary, 29°34'47.99"S, 31°41'59.99"E; 138 m.

###### Description.

Corallum (anthocyathus) small, elongated, and with a smooth calicular margin. New specimen examined (SAM_H3847) has a slightly damaged calicular margin: 7.1 × 6.9 mm in CD (estimated), and 16.6 mm in H. Basal scar 3.6 × 2.5 mm in diameter, with 12 complete septa originating from scar. Theca with longitudinal striae, and transverse chevron-shaped growth lines. Thecal faces carinate and diverging in an angle between 14 and 18°. Corallum white, with beige theca.

Septa hexamerally arranged in four complete cycles according to the formula: S_1_ ≥ S_2_ > S_3_ > S_4_ (48 septa). However, newly examined specimen has fourth cycle incomplete (total of 42 septa). S_1_ as wide to only slightly wider than S_2_, both having vertical and slightly sinuous axial margin that extend to columella. S_3_ ½ width of S_2,_ and bearing moderately sinuous axial margin. S_4_ ~ ^1^/_3_ the width of S_3_, usually rudimentary, and having dentate axial margin. Septal faces bear blunt granules obliquely aligned in rows along septal margin. Fossa moderately deep with a rudimentary columella formed by the fusion of lower S_1–2_ axial margins.

###### Distribution.

Regional: Eastern margin of South Africa, off Shaka’s Rock; 138 m. Elsewhere: Japan; (Cairns 1994); 100–144 m.

###### Remarks.

The original author of *Truncatoflabellumgardineri* provided a detailed description of the species and, therefore, the description above is mainly based on the single new South African specimen (SAM_H3847). Although this specimen is slightly damaged around the calicular margin, characteristic features are observable, providing information on ontogenetic variability. Unlike the type specimens, the newly examined specimen has slightly larger S_1_ as compare with having equal-sized S_1–2_. Furthermore, the newly examined specimen has the fourth cycle incomplete rather than four or five complete ones (Cairns 2016). Apart from these observations, the specimen fits the known characteristics of the species.

##### Truncatoflabellum inconstans

Taxon classificationAnimaliaScleractiniaFlabellidae

(von Marenzeller, 1904a)

CCB42CE8-F94C-51DC-BAFB-09F0694D7666

Fig. 12M, N

Flabellum inconstans von Marenzeller, 1904a: 277–280, pl. 27, figs 11 A–L. –Boshoff 1981: 34.
Flabellum harmeri . –Boshoff 1981: 35.
Truncatoflabellum inconstans . –Cairns 1989b: 61. –Cairns and Keller 1993: 220. –Cairns 2016: 34, fig. 9D.

###### Type locality.

Off St. Francis Bay, South Africa (SS ‘Valdivia’ stn. 100: 34°08'09"S, 24°59'30"E); 100 m (von Marenzeller 1904a).

###### Type material.

The syntype are deposited at the ZMB (GBIF 2020).

###### Material examined.

**ORI_DIIb2 (1 specimen)**: Locality data unknown. ORI_ DIIb3 (in part: 1 specimen): Locality data unknown, 27 m. SAMC–A072990 (1 specimen), SAMC–A072991 (3 specimens): Locality data unknown. **SAMC–A090103 (1 specimen)**: Southern margin, 15 km from Arniston/21 km from De Mond-Heuningnes Estuary, 34°46'59.99"S, 20°19'00.00"E; 80 m. SAM_H1241 (in part: 1 specimen): Southern margin, 2 km from Kei Mouth/27 km off Groot Berg Estuary, 32°40'33.99"S, 28°22'50.99"E; 66 m. SAM_H1381 (1 specimen): Eastern margin, 2 km from Durban/8 km off Umgeni Estuary, 29°51'59.99"S, 31°00'00.00"E; 101 m. SAM_H1382 (2 specimens): Southern margin, 48 km from Cape Padrone/47 km off Bakens River Estuary, 34°04'59.99"S, 26°05'59.99"E; 37 m. SAM_H1386 (1 specimen): Southern margin, 28 km from Gonubie/27 km off Buffalo Estuary, 33°09'29.99"S, 28°03'06.00"E; 86 m. SAM_H1392 (2 specimens): Southern margin, 6 km from Kenton On Sea/5 km off Boknes Estuary, 33°43'07.59"S, 26°37'37.95"E; 90 m. SAM_H1417 (1 specimen): Southern margin, 29 km from Kenton On Sea/off Boesmans Estuary, 33°53'39.99"S, 26°51'00.00"E; 121 m. SAM_H1425 (1 specimen): Southern margin, 7 km from East London/5 km off Buffalo Estuary, 33°02'59.99"S, 27°57'00.00"E; depth unknown. SAM_H1434 (4 specimens): Locality data unknown. SAM_H1435 (1 specimen): Southern margin, 6 km from Kidds Beach/5 km off Ncera Estuary, 33°11'59.99"S, 27°40'59.99"E; 79 m. SAM_H1444 (2 specimens): Southern margin, 14 km from Cape Padrone/26 km off Boknes Estuary, 33°46'00.00"S, 26°19'00.00"E; 104 m. SAM_H1446 (1 specimen): Southern margin, 6 km from Kidds Beach/5 km off Ncera Estuary, 33°11'59.99"S, 27°40'59.99"E; 79 m. SAM_H1470 (2 specimens): Eastern margin, 27 km from Mtunzini/25 km off Matigulu Estuary, 29°10'36.00"S, 31°51'00.00"E; 115 m. SAM_H1511 (2 specimens): Southern margin, 25 km from Port Elizabeth/23 km off Bakens River Estuary, 33°49'36.36"S, 25°47'42.94"E; 49–183 m. SAM_H3071 (34 specimens): Southern margin, 6 km from Kenton On Sea/5 km off Boknes Estuary, 33°43'07.59"S, 26°37'37.95"E; 90 m. SAM_H3130 (1 specimen): Southern margin, 46 km from Port Alfred/12 km off Mgwalana Estuary, 33°29'24.00"S, 27°21'11.99"E; 80 m. SAM_H3155 (1 specimen): Southern margin, 47 km from Port Alfred/14 km off Mgwalana Estuary, 33°30'18.00"S, 27°22'05.99"E; 80 m. DEFF_NANSEN–INV 19 (2 specimens): Eastern margin, 20 km from Durban/19 km off Beachwood Mangroves; 29°52'56.39"S, 31°12'15.59"E; 218 m.

###### Imagery data.

BMNH 1939.7.20.816–833 (in part: 1 specimen): Southern margin, 7 km from East London/5 km off Buffalo Estuary, 33°02'59.99"S, 27°57'00.00"E; 62 m. BMNH–1950.1.10.97–102 (in part: 1 specimen): Locality data unknown. DTE 100 (syntype: 1 specimen): Southern margin, 12 km from Jeffreys Bay/21 km off Gamtoos Estuary, 34°08'53.99"S, 24°59'17.99"E; 100 m. Mortensen St 30 (2 specimens): Eastern margin, off Durban; 94 m. Mortensen St 31 (14 specimens): Eastern margin, off Durban; 128 m.

###### Description.

Corallum (anthocyathus) large, robust, and having a small open base. Calice elliptical to sub-cylindrical (GCD:LCD = 1.47–2.60), with a thin and smooth calicular margin. Basal scar 5.3–6.6 × 3.4–4.0 mm in diameter, with 24 complete septa originating from scar. Largest anthocyathus examined (SAM_H1386) 25.6 × 18.1 mm in CD and 37.4 mm in H. Theca bears fine longitudinal striae, and chevron-shaped growth lines. C_1–3_ distinct and extend from calicular margin to base. Thecal edge smooth, rounded, or acute. Thecal edge diverges in an angle between 45 and 55°, and thecal faces between 20 and 25°. Corallum white.

Septa hexamerally arranged in six cycles, last being incomplete, according to the formula: S_1–3_ > S_4_ > S_5_ > S_6_ (102 septa). However, smaller coralla (< 25 mm GCD) display ≤ 78 septa. S_1–3_ greater in width than S_4,_ and having vertical, slightly sinuous, and thickened axial margins. S_4_ ~ ½ the width of S_1–3,_ and bear sinuous axial margin, which sometimes appear to be dentate. S_5_^1^/_3_ the width of S_4,_ and have dentate axial margin. However, if S_6_ is absent, S_5_ rudimentary. S_6_ rudimentary. Septal faces covered with granules obliquely aligned in rows along septal margin. Fossa deep containing a rudimentary columella formed by S_1–3_ lower axial margin.

###### Distribution.

Regional: Southern to eastern margin of South Africa, off Jeffreys Bay extending towards Durban; 29–183 m. Elsewhere: Only known from South Africa.

###### Remarks.

The South African endemic *Truncatoflabelluminconstans* differs from the other South African congeners by lacking thecal spines, in thecal edge and thecal face inclinations, GCD:LCD ratio, H:GCD ratio, number of septa, and distinction of ribbed C_1–3_ (Cairns 2016). However, *T.inconstans* may be mistaken with *T.stabile* (von Marenzeller, 1904a); *T.inconstans* differs in having a thecal edge angle inclination range of 40–50° (as opposed to the 60–90° range of *T.stabile*), and a H:GCD between 1.0–1.5 (compare with 0.7–1.1 of *T.stabile*) (key in Cairns 2016: 9).

##### Truncatoflabellum multispinosum

Taxon classificationAnimaliaScleractiniaFlabellidae

Cairns in Cairns & Keller, 1993

811CA57A-58F7-53EC-92A7-0E0B6D8D7B0A

Fig. 12O–P.

Truncatoflabellum multispinosum Cairns in Cairns & Keller, 1993: 268 and 272, figs 11H, 12A–C. –Cairns 1999b: 32. –Cairns 2016: 32, fig. 8D. –Tenjing et al. 2019: 92. –Kitahara and Cairns 2021: 251, 253, figs 128, 131A–D.

###### Type locality.

Off Quissico, south-eastern Mozambique (RV ‘Vityaz’ stn. 2634: 25°05'00"S, 34°50'00"E); 90–92 m (Cairns and Keller 1993).

###### Type material.

The holotype and most paratypes are deposited at NMNH, whilst one paratype is deposited at the SAM (Cairns and Keller 1993).

###### Material examined.

SAMC_A087450 (2 specimens): Locality unknown. **SAM_H4580 (1 specimen: paratype)**: Eastern margin, 256 km south of Ponta Do Ouro/1 km off Elsies Estuary, 25°07'00.00"S, 34°34'00.00"E; 112 m. **USNM 91746 (1 paratype)**: Eastern margin, 73 km from Cape Vidal/17 km off Mgobezeleni Estuary, 27°31'21.60"S, 32°49'10.80"E; 75 m.

###### Description.

Corallum medium-sized (GCD ~25.0 mm) and sub-cylindrical. Calice elliptical (GCD:LCD = 2.05–2.80), with a thin and slightly serrated calicular margin. Basal scar 8.9 × 4.9 mm in diameter, with 24 complete septa observable on scar. Largest anthocyathus examined (SAMC_A087450) 22.3 × 11.9 mm in CD, and 18.4 mm in H. Five pairs of slender and rounded thecal spines occur on thecal edges. Thecal faces diverge in an angle ~ 55°, and thecal edges ~ 30°. Corallum white, with theca being faintly greenish brown.

Septa decamerally arranged in five cycles, last being incomplete, according to the formula: S_1–2_ > S_3_ > S_4_ > S_5_ (84 septa). S_1–2_ slightly larger than S_3,_ but all having vertical and slightly sinuous axial margins. S_4_ ½ the width of S_1–3_ and bear sinuous and slightly dentate axial margin. S_5_ rudimentary. Septal faces covered with granules obliquely aligned in rows along axial septal margin. Fossa deep with a trabecular columella formed by the fusion of lower axial margins of S_1–3._

###### Distribution.

Regional: Eastern margin of South Africa, off Cape Vidal extending towards Elsies Estuary (256 km south of Ponta Do Ouro: Mozambique); 67–75 m. Elsewhere: Zanzibar; Mozambique; Madagascar (Cairns and Keller 1993); New Caledonia (Cairns 2016); Kitahara and Cairns 2021 62–350 m.

###### Remarks.

*Truncatoflabellummultispinosum* is well described by Cairns and Keller (1993), who outline the variation in symmetry and number of septa in relation to corallum size. The two newly examined specimens represent a medium-sized anthocyathus (above description is based on this specimen). *Truncatoflabellummultispinosum* and *T.vanuatu* (Wells, 1984) are the only two Recent species reported to have ≤ five pairs of thecal spines. However, *T.multispinosum* differs by having a larger corallum, greater thecal edge angle range, and a higher number of septa (Cairns and Keller 1993; Cairns 2016).

##### Truncatoflabellum pusillum

Taxon classificationAnimaliaScleractiniaFlabellidae

Cairns, 1989a

0CAB31B7-87E4-51D0-9F87-96A4E9CC732E

Fig. 13A, B

Truncatoflabellum pusillum Cairns, 1989a: 71–72, pl. 37, figs A–E. –Cairns and Keller 1993: 265, pl. 11, fig. E. –Cairns and Zibrowius 1997: 170. –Cairns 1999a: 120, figs 19G, H. –Cairns et al. 1999: 32. –Randall 2003: 136. –Cairns 2009: 19. –Cairns 2016: 16, fig. 3B. –Kitahara and Cairns 2021: 256–257, 259, figs 134, 135A–L.

###### Type locality.

Sibuyana Sea, Philippines (RV ‘Albatross’ stn. 5178: 12°43'N, 122°06'15"E); 143 m (Cairns 1989a).

###### Type material.

Types are at the NMNH (Cairns1989a).

###### Material examined.

SAMC_A073189 (1 specimen): Eastern margin, 9 km from Shaka’s Rock/12 km off Mhlali Estuary, 29°32'12.11"S, 31°19'36.11"E; 50 m. SAMC_A073200 (1 specimen): Eastern margin, 19 km from Shaka’s Rock/20 km off Mhlali Estuary, 29°33'11.88"S, 31°25'23.88"E; 60–64 m. SAM_H2838 (57 specimens): Eastern margin, 19 km from Shaka’s Rock/3 km off Mdloti Estuary, 29°38'59.99"S, 31°07'59.99"E; 71–73 m. SAM_H2839 (24 specimens): Southern margin, 48 km from Cape Padrone/47 km off Bakens River Estuary, 34°04'59.99"S, 26°05'59.99"E; 37 m.

###### Description.

Corallum small (GCD < 13.0 mm). Calice may be short or long, but always highly compressed (GCD:LCD = 1.1–1.3). Calicular margin thin and slightly serrated. Basal scar 4.0 × 2.8 mm in diameter, with 24 septa originating from scar. Largest anthocyathus examined (SAM_H2839) 15.2 × 7.6 mm in CD, and 13.4 mm in H. Theca porcelaneous, bearing thin transverse chevron-shaped lines. Thecal spines sometimes present (≤ 3), usually of various size and shape, but consistently facing downwards. Thecal faces diverge in an angle between 18 and 20°, and thecal edge between 14 and 18°. Corallum white, with faint greenish brown stripes on theca.

Septa hexamerally arranged in five cycles, last being incomplete, according to the formula: S_1_ ≥ S_2_ > S_3_ > S_4_ > S_5_ (≤ 60 septa). S_1_ ca. the same size or only slightly larger than S_2_. S_1–2_ axial margins vertical and sinuous. S_3_ ¾ the width of S_1–2_ and bear sinuous to dentate axial margin. S_4_ small, ~ ^1^/_3_ the width of S_3_, and have dentate axial margin. S_5_ rudimentary. Septal faces covered with granules. Fossa of moderate depth, containing a rudimentary columella formed by the fusion of S_1–3_ lower axial margins.

###### Distribution.

Regional: Southern and eastern margin of South Africa, off Cape Padrone extending towards Shaka’s Rock; 37–73 m. Elsewhere: Philippines (Cairns 1989a; Cairns and Zibrowius 1997); Indonesia (Cairns and Zibrowius 1997); New Caledonia (Cairns 2016 ; Kitahara and Cairns 2021); Mozambique (Cairns and Keller 1993); 85–460 m.

###### Remarks.

Although *Truncatoflabellumpusillum* was previously known from the southwest Indian Ocean, the new records reported herein represent a further southwards range extension, from Mozambique into South Africa. The South African specimens vary from the existing descriptions (Cairns 1989a; Cairns and Zibrowius 1997; Cairns 1999a) in having ≤ 60 septa instead of 48, a larger CD (12.0 mm as compare with 8.0 mm), and a basal scar of 4 × 3 mm (rather than 2.4–3.2 × 1.7–1.8 mm). The South African *T.pusillum* resembles *T.dens* in septal symmetry and CD size, but differs in having thecal spines (rather than crests), and thecal edge diverging in an angle of 14–18° instead of a bimodal edge angle.

**Figure 13.**
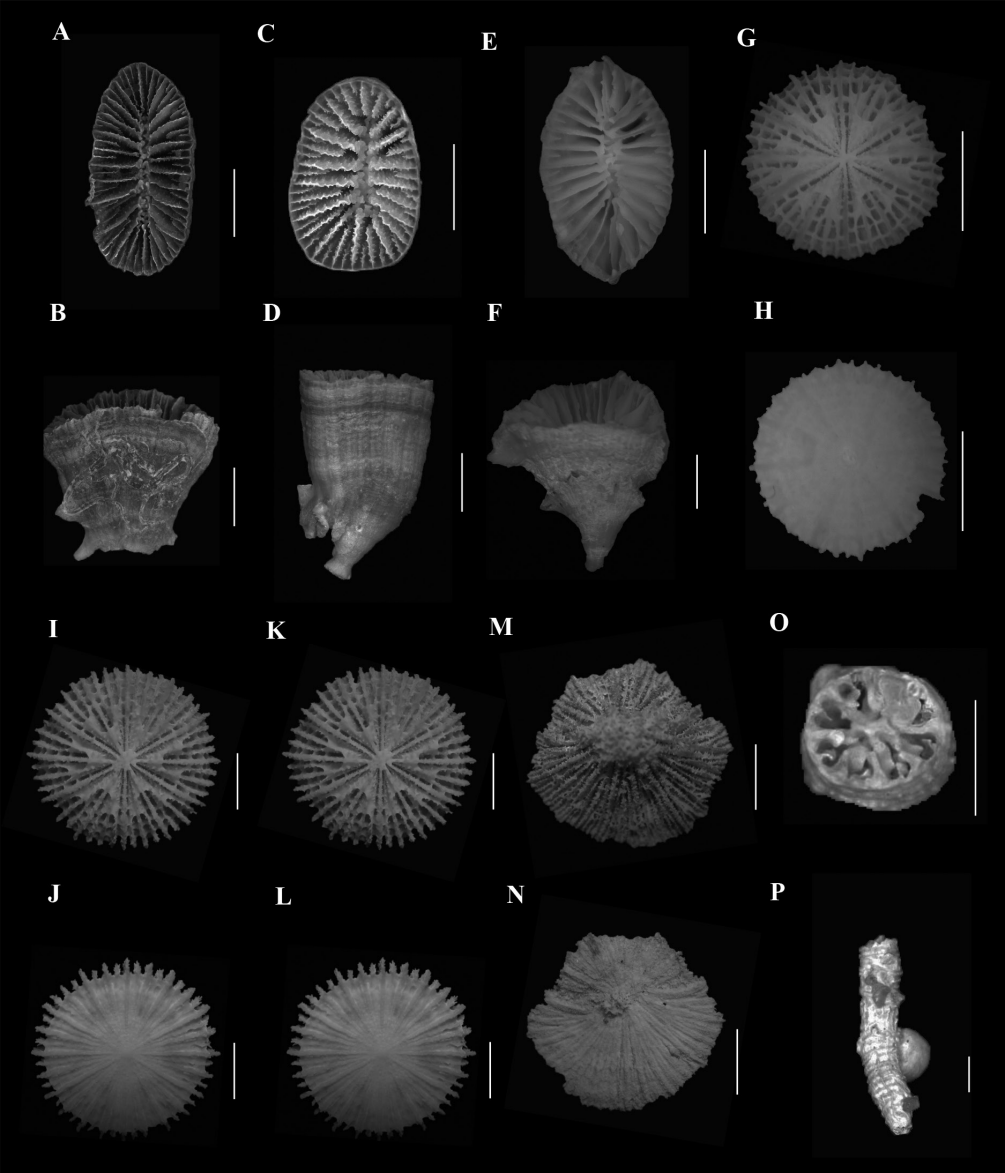
**A**, **B***Truncatoflabellumpusillum* (SAM_H2839, off Cape Padrone, 37 m) **A** calicular view **B** lateral view **C**, **D***Truncatoflabellumzuluense* (SAMC_A090096, off Cape Vidal, 85 m) **C** calicular view **D** lateral view **E**, **F***Truncatoflabellum* sp (SAMC_A073161, off Port St. Johns, 140–145 m) **E** calicular view **F** lateral view **G**, **H**Fungiacyathus (Bathyactis) hydra (USNM 86869, off Alexandra Bay, 882 m) **G** calicular view **H** basal view **I**, **L**Fungiacyathus (Bathyactis) sibogae (SAM_H1697, off Mgobezeleni Estuary, 1050 m) **I** calicular view **J** basal view **K** calicular view **L** basal view **M**, **N**Fungiacyathus (Fungiacyathus) sp. (SAM_H1431, off Durban, 99 m) **M** calicular view **N** basal view **O**, **P***Guyniaannulata* (USNM 77201, off Port St. Johns, 138 m) **O** calicular view **P** lateral view. Scale bars: 10 mm (**A–N**); 2 mm (**O**, **P**).

##### Truncatoflabellum zuluense

Taxon classificationAnimaliaScleractiniaFlabellidae

Cairns in Cairns & Keller, 1993

DE9F69FE-C0D0-5AFC-A59E-308A001423BE

Fig. 13C, D

Truncatoflabellum zuluense Cairns in Cairns & Keller, 1993: 267–268, figs 11F, G. –Cairns 2016: 16, fig. 3A.
Flabellum inconstans . –Boshoff 1981: 34.

###### Type locality.

Off Zululand, South Africa (RV ‘Meiring Naude’ stn. ZK21: 27°47'00"S, 32°39'10"E); 62–84 m (Cairns and Keller 1993).

###### Type material.

The holotype and most paratypes are deposited at NMNH, whilst one paratype being deposited at SAM (Cairns and Keller 1993).

###### Material examined.

SAMC_A073113 (1 specimen): Eastern margin, 47 km from Cape Vidal/21 km from Mgobezeleni Estuary, 27°43'14.88"S, 32°40'36.11"E; 110 m. SAMC_A073182 (1 specimen): Southern margin, 26 km from East London/24 km off Buffalo Estuary, 33°11'48.11"S, 28°03'11.88"E; 90 m. SAMC_A090095 (1 specimen): Eastern margin, 16 km from Scottburgh/12 km off Mkomazi Estuary, 30°15'00.00"S, 30°54'18.00"E; 100 m. SAMC_A090096 (1 specimen): Eastern margin, 66 km from Cape Vidal/7 km off Mgobezeleni Estuary, 27°33'11.99"S, 32°43'00.00"E; 85 m. SAM_H1398 (12 specimens): Southern margin, 32 km from Cintsa/11 km off Cwili Estuary, 32°45'45.00"S, 28°26'15.00"E; 66 m. SAM_H3156 (12 specimens): Southern margin, 18 km from Gonubie/off Gqunube Estuary, 33°04'35.99"S, 28°06'35.99"E; 90 m. SAM_H4581 (1 specimen: paratype): Eastern margin, 25 km south of Ponta Do Ouro/17 km from Kosi Bay Estuary, 27°04'47.99"S, 32°53'30.00"E; 65 m. **USNM 91747 (Holotype)**: Eastern margin, 39 km from Cape Vidal/29 km off Mgobezeleni Estuary, 27°47'21.59"S, 32°39'03.60"E; 62–84 m.

###### Description.

Corallum small to medium-sized (GCD ~ 10.0–18.0 mm), with thecal edge diverging in an angle between 28 and 38°. Calice compressed (GCD:LCD = 1.1–1.8), with a slightly serrated calicular margin. Basal scar 6.8 × 4.8 mm in diameter, with 24 septa originating from scar. Largest specimen examined (SAMC_A073182) 19.0 × 10.6 mm in CD, and 18.7 mm in H. Theca smooth, sometimes bearing a pair of basal spines. Thecal faces diverge in an angle ~ 18–23° and thecal edge ~ 35–48°. Anthocyathi and anthocauli remain attached, but fracture zone is demarcated by a thin line. Corallum white, with costae a darker tint of reddish or greenish brown.

Septa hexamerally arranged in five cycles, the last cycle incomplete, according to the formula: S_1–2_ > S_3_ >> S_4_ > S_5_ (≤ 80 septa). S1–2 bear vertical and sinuous axial margins. S_3_ ~ ¾ the width of S_1–2,_ also with sinuous axial margin. S_4_ small, ~ ^1^/_3_ the width of S_3_, and bearing dentate axial margin. S_5_ rudimentary. Septal faces granular. Fossa deep, with a rudimentary columella formed by the fusion of S_1-2_ lower axial margins.

###### Distribution.

Regional: Southern and eastern margin of South Africa, from off Gonubie extending towards Cape Vidal; 62–110 m. Elsewhere: Only known from South Africa.

###### Remarks.

*Truncatoflabellumzuluense* is known to occur in the KwaZulu-Natal region and is distinctive in the anthocaulus often remains attached to the anthocyathus throughout development. Amongst the 38 Recent species of the genus, *T.zuluense* and *T.dens* (Alcock, 1902a) are the only two species known to usually maintain such an attachment long into ontogenesis. However, the two species may be distinguished by thecal face (14–18° in *T.dens* vs. 18–22° in *T.zuluense*) and thecal edge angles (bimodal in *T.dens* vs. 35 to 48° in *T.zuluense*), and GCD:LCD ratio (1.7–2.3 in *T.dens* vs. 1.4–2.0 in *T.zuluense*). Among South African congeners, *T.zuluense* may be mistaken with *T.gardineri*, whereby the resemblance and morphological differences between them are detailed by Cairns and Keller (1993) in their account of the species and keyed by Cairns (2016).

##### Truncatoflabellum

Taxon classificationAnimaliaScleractiniaFlabellidae

sp.

91C6CABA-6BFE-5959-A563-563D040EF6A3

Fig. 13E, F

###### Material examined.

SAMC_A073161 (1 specimen): Eastern margin, 25 km from Port St. Johns/16 km off Cwili Estuary, 31°49'59.99"S, 29°40'00.00"E; 140–145 m.

###### Description.

Corallum small, with thecal edges diverging at 30°. Calice flared and elliptical (GCD:LCD = 1.7), with a slightly serrated calicular margin. Specimen examined 12.7 × 7.6 mm in CD, 1.3 mm in PD, and 14.2 mm in H. Theca smooth. Thecal faces diverge in an angle of ~ 60°, and thecal edge of ~ 23°. Specimen has anthocyathus still attached to anthocaulus, but a fracture line indicates that they were about to transversely divide. Corallum white.

Septa thick and hexamerally arranged in four cycles, the last being incomplete, according to the formula: S_1–3_ > S_4_ (45 septa). S_1–3_ axial edges straight, but become slightly sinuous deeper in fossa. S_4_ rudimentary and bearing sinuous axial margin. Septal faces smooth, occasionally with sparsely arranged granules. Fossa of moderate depth, with a rudimentary columella formed by the fusion of the S_1–3_ lower axial margins.

###### Distribution.

Regional: Eastern margin of South Africa, off Port St. Johns; 140–145 m.

###### Remarks.

The examined specimen is unique in its calice being flared, lateral margin diverging at an angle of 23°, and having distinctly thick septa. More specimens are needed to confirm the possibility of it being an undescribed species.

#### Family Fungiacyathidae Chevalier, 1987

##### Fungiacyathus

Taxon classificationAnimaliaScleractiniaFungiacyathidae

Sars, 1872

C5276339-4857-5E38-8961-194AD1850706

###### Diagnosis.

Corallum solitary, cupolate, free. Septotheca horizontal. Costae either thin serrate ridges or rounded and granular. Four or five cycles of septa. Septal faces carinate. Septa usually linked to their adjacent septa by synapticular plates. Pali may be present. Columella spongy.

###### Type species.

*Fungiacyathusfragilis* Sars, 1872, by monotypy.

##### Fungiacyathus (Bathyactis)

Taxon classificationAnimaliaScleractiniaFungiacyathidae

Moseley, 1881

86B3FF44-C373-5A28-BC04-F7453B44F631

###### Diagnosis.

*Fungiacyathus* with four septal cycles (48 septa).

###### Type species.

*Fungiasymmetrica* Pourtalès, 1871, by monotypy.

##### Fungiacyathus (Bathyactis) hydra

Taxon classificationAnimaliaScleractiniaFungiacyathidae

Zibrowius & Gili, 1990

904E6DC9-BDA9-5152-AB3D-B944F8C29958

Fig. 13G, H

Fungiacyathus hydra Zibrowius & Gili, 1990: 22–25, pl. 1, figs A–N.

###### Type locality.

Off Walvis Ridge (*Benguela VI* Expedition stn. BB12: 25°34'00"S, 06°07'00"E); 882–886 m (Zibrowius and Gill 1990).

###### Type material.

The holotype and paratypes are deposited at the NMNH (Zibrowius and Gili 1990).

###### Material examined.

**USNM 86869 (3 paratypes)**: Western margin, off Alexandra Bay; 882 m.

###### Description.

(based on Zibrowius and Gill 1990) Corallum small (GCD ~ 9.0–12.0 mm), fragile, discoidal, with a slightly concave, convex, or flat base. Calice circular and ~ 5.0 mm in H. Base bear distinct, granular to serrate, costa. C_1–2_ prominent near calicular margin, becoming less so towards centre of base. C_3_ and C_4_ unequal and less developed than C_1_ and C_2_. Intercostal spaces fairly deep and smooth. Corallum white.

Septa hexamerally arranged in four cycles according to the formula: S_1_ > S_2_ > S_3–4_ (48 septa). S_1_ independent but joining adjacent S_4_ by extended synapticular bars. S_2_ ¼ smaller than S_1_, whilst S_3–4_ are equal in width. S_2–4_ solidly fused, thus forming distinct delta junctions. Septal faces smooth, with no dentations. Columella absent or may be present as narrow tabular spines

###### Distribution.

Regional: Western margin of South Africa, off Kerbehuk; 882 m (Zibrowius and Gill 1990). Elsewhere: Walvis Ridge; 882–886 m (Zibrowius and Gill 1990).

###### Remarks.

The regional occurrence of Fungiacyathus (Bathyactis) hydra is based on Zibrowius and Gili (1990), and no additional specimens have since been reported. Fungiacyathus (B.) hydra resembles *F.symmetricus* (Pourtalès, 1871) in having a small-sized corallum, but can be distinguished by its less sinuous septa and poorly developed columella (Zibrowius and Gili 1990).

##### Fungiacyathus (Bathyactis) sibogae

Taxon classificationAnimaliaScleractiniaFungiacyathidae

(Alcock, 1902a)

81B8323F-F73A-59F4-AE35-9960DBCEF8BB

Fig. 13I–L

Bathyactis sibogae Alcock, 1902a: 108.
Bathyactis symmetrica . –von Marenzeller 1904a: 312–313, pl. 18, fig. 25. –Gardiner and Waugh 1939: 231.
Bathyactis stabilis Gardiner & Waugh, 1939: 231–232, figs 1, 2.
Fungiacyathus (Bathyactis) sibogae . –Cairns 1989b: 10–11, pl. 3, figs D–K, pl. 4, figs A–C. –Cairns and Keller 1993: 218, 230. –Cairns and Zibrowius 1997: 70.

###### Type locality.

Ceram Sea, Indonesia (HMS ‘Siboga’ stn. 175: 2°37.7'00"S, 130°33.4'00"E); 1914 m (Alcock, 1902a).

###### Type material.

The paralectotype is deposited at the ZMA (Cairns and Zibrowius 1997).

###### Material examined.

None.

###### Imagery data.

SAM_H1403 (1 specimen): Eastern margin, off Durban harbour, 805 m. SAM_H1688 (in part: 2 specimens): eastern margin, 51 km south of Ponta Do Ouro/40 km off Mgobezeleni Estuary, 27°18'42.00"S, 32°57'47.99"E; 840 m. SAM_H1689 (in part: 1 specimen): eastern margin, 44 km south of Ponta Do Ouro/35 km off Kosi Bay Estuary, 27°14'47.99"S, 32°57'59.99"E; 900 m. SAM_H1697 (2 specimens): eastern margin, 58 km south of Ponta Do Ouro/46 km off Mgobezeleni Estuary, 27°21'18.00"S, 33°03'53.99"E; 1050 m.

###### Description.

Corallum discoidal, with a flat to slightly convex base, reaching ≤ 14.5 mm in CD. Costae ridged, serrate, and narrow. C_1_–_2_ ridged from epicentre of base to calicular margin, and C_3_–_4_ ridged only near calicular margin. Costae correspond with septa in width, with C_1_–_2_ equal in width and C_3_–_4_ becoming progressively narrower. Intercostal spaces moderately deep and granulated. Corallum white.

Septa hexamerally arranged in four cycles according to the formula: S_1_ > S_2_ > S_3_ > S_4_ (48 septa). S_1_ highly exsert and extend to columella. S_1_ appear to be independent, but are connected to adjacent S_4_ by five or six synapticular bars, which gradually increase in size near calicular margin. S_2_ slightly less exsert and less wide than S_1_. S_2_ bear 4–6 trabecular spines, of which the third and fourth are the tallest and often curved towards columella. S_3_ equally exsert and slightly wider than S_2_, bearing 4–7 trabecular spines, of which the fourth to sixth are elongated and inclined towards columella. S_4_ least exsert and smaller septa, but have nine or ten trabecular spines. S_2_–_4_ fuse in a typical fungiacyathid fashion, forming porous and rudimentary canopies. All septa, except S_4_, have septal lobe occurring peripheral to spines. Septal faces planar, covered with serrated ridges, which are covered by broad and well-spaced teeth, thus giving a dentated appearance. Marginal shelf absent. Columella papillose.

###### Distribution.

Regional: Eastern margin of South Africa, off Durban towards Kosi Bay Estuary (35 km south of Ponta Do Ouro: Mozambique); 805–1050 m. Elsewhere: Mozambique (Cairns and Keller 1993); Tanzania (von Marenzeller 1904a); Kenya; Madagascar; Gulf of Oman (Gardiner and Waugh 1939); Indonesia (Alcock 1902a; Cairns 1989b; Cairns and Zibrowius 1997); 463–1914 m.

###### Remarks.

Fungiacyathus (B.) sibogae differs from *F.hydra* in its septal faces being dentate, and its papillose columella. Although the above records are based on images of specimens that we were unable to track in the South African Museum collection, the diagnostic features are visible and as such specimens fit the description provided in Cairns (1989b).

##### Fungiacyathus (Fungiacyathus)

Taxon classificationAnimaliaScleractiniaFungiacyathidae

(Sars, 1872)

B11EAA83-D07A-5909-AF46-719D6A242404

###### Diagnosis.

*Fungiacyathus* with five septal cycles (96 septa).

###### Type species.

*Fungiacyathusfragilis* Sars, 1872, by monotypy.

##### Fungiacyathus (Fungiacythus) stephanus

Taxon classificationAnimaliaScleractiniaFungiacyathidae

(Alcock, 1893)

2E6EEC60-1EEC-532C-8B5D-ED63BA7E72E6

Bathyactis stephanus Alcock, 1893: 149, pl. 5. figs 12, 12A.
Bathyactis stephana . –Alcock 1898: 11, 28–29, pl. 3.
Bathyactis sibogae Alcock, 1902a (in part). –Alcock 1902c: 38 (in part) .
Bathyactis symmetrica . –Gardiner and Waugh 1939: 230–231 (in part).
Bathyactis stephana . –Gardiner and Waugh 1939: 232.
Fungiacyathus (Fungiacythus) stephanus . –Cairns 1989a: 7–9, pl 1, figs A–K, pl. 2, figs A–N. –Cairns and Keller 1993: 218, 230. –Cairns 1995: 31–32, pl 1, figs A–C. –Cairns and Zibrowius 1997: 68–69. –Cairns 1998. 369. –Cairns 1999. –Cairns 54–56. –Cairns 2004a: 271. –Kitahara and Cairns 2021: 162–164, figs 79E–F, 80.

###### Type locality.

Off Krishna Delta, Bay of Bengal; (HMS ‘Investigator’ stn. 133: 15°43'30"N, 81°19'30"E); 1240 m (Cairns 1989a).

###### Type material.

The holotype is presumed to be deposited at the IM (Cairns 1989b).

###### Material examined.

None.

###### Distribution.

Regional: Eastern margin of South Africa, off Natal (Gardiner and Waugh 1939); depth unknown. Elsewhere: Mozambique (Cairns and Keller 1993); Gulf of Aden (Gardiner and Waugh 1939); Japan (Cairns 1994a); Bay of Bengal (Alcock 1893); Philippines; Indonesia (Cairns 1989b; Cairns and Zibrowius 1997); Malaysia; Wallis and Futuna; Vanuatu (Cairns 1999a); Australia; New Zealand (Cairns 1995, 1998); 245–2000 m.

###### Remarks.

Fungiacyathus (F.) stephanus was first reported from South Africa as *Bathyactisstephana* (Gardiner and Waugh 1939). Subsequently, Cairns and Keller (1993) also reported on records from the region. Of the five other Recent species (*F.fragilis* Sars, 1872, *F.paliferus* (Alcock, 1902a), *F.pusilluspusillus* (Pourtalès, 1868), *F.pusilluspacificus* Cairns, 1995, *F.multicarinatus* Cairns, 1998, *F.sandoi* Cairns, 1999), *F.stephanus* resembles *F.fragilis* but differs in having smaller P_2_, its S_1–2_ bearing higher septal lobes, and the concave-based forms having a marginal shelf.

##### Fungiacyathus (Fungiacyathus)

Taxon classificationAnimaliaScleractiniaFungiacyathidae

sp.

4A5671D8-145E-5AC1-8D42-594736703647

Fig. 13M, N

Fungiacyathus sp. –Zibrowius and Grygier 1985: 119–120, figs 6–9.

###### Material examined.

None.

###### Imagery data.

**SAM_H1431** (1 specimen with excavations): Eastern margin, 5 km from Durban/7 km off Umgeni Estuary, (RV ‘Pieter Faure’: 29°52'00.00"S, 31°03'00.00"E); 99 m.

###### Distribution.

Regional: Eastern margin of South Africa, Durban; 99 m (Zibrowius and Grygier 1985).

###### Remarks.

Two species of fungiacyathids (*F.stephanus* and *F.paliferus*) with five cycles of septa have been reported within the south-western Indian Ocean (Cairns and Keller 1993). *Fungiacyathusstephanus* differs from *F.paliferus* in its fragile corallum, sinuous septal margin, and fairly high septal lobes (Cairns 1989a). Nonetheless, due to most of the diagnostic features being damaged in this specimen, we have retained this entry at genus level.

#### Family Guyniidae Hickson, 1910

##### Guynia

Taxon classificationAnimaliaScleractiniaGuyniidae

Duncan, 1872

FCDBD388-2AE1-57C6-8FDD-8515E676EC34

###### Diagnosis.

Solitary, ceratoid to scolecoid, free or fixed laterally. Chain of individuals sometimes produced by extra-tentacular budding. Wall epithecal. A row of mural “pores” present in every interseptal space. Pali absent. Columella composed of one twisted ribbon.

###### Type species.

*Guyniaannulata* Duncan, 1872, by monotypy.

##### Guynia annulata

Taxon classificationAnimaliaScleractiniaGuyniidae

Duncan, 1872

05D1BA20-A053-5D21-B995-1C54D93367C6

Fig. 13O, P

Guynia annulata Duncan, 1872: 32, pl. 1, figs 1–8. –Duncan 1873: 335–336, pl. 47, figs 9–16. –Pourtalès 1874: 44, pl. 9, figs 3–4. –Pourtalès 1878: 209–Pourtalès 1880: 97, 112.– Hickson 1910: 5. –Gardiner and Waugh 1938: 172. –Zibrowius 1969: 327–328. –Wells 1972: 6, figs 11–14. –Wells and Lang 1973: 58. –Wells 1973: 59–63, figs 1–3. –Bourcier and Zibrowius 1973: 827. –Zibrowius and Saldanha 1976: 101–102. –Zibrowius and Grieshaber 1977: 381. –Cairns 1977b: 5. –Cairns 1978: 11. –Cairns 1979: 164–165, pl. 32, figs 1–3. –Zibrowius 1980: 161–162, pl. 83, figs A–Q. –Gili 1982: 131, 137–138, fig. 62H. –Cairns 1984: 23, pl. 5, figs A, B. –Cairns et al. 1986: 187–188, pl. 56. –Cairns 1989a: 42–43, pl. 21, fig. f, pl. 42, figs A–E. –Cairns et al. 1991: 48. –Cairns and Parker 1992: 42–43, pl. 14, figs G, H. –Cairns and Keller 1993: 273, figs 12H, I. –Cairns and Zibrowius 1997: 150. –Cairns 1998: 392. –Cairns 1999a: 113–114. –Cairns 2000: 148–149, figs 170, 173. –Stolarski 2000: 13–33, figs 1A, 2, 3A–C, E, F, 4G. –Romano and Cairns 2000: 1048, 1052, 1054. –Stolarski 2000: 13–38, figs 1, 2, 3A–C, 4D. –Randall 2003: 136. –Cairns 2004a: 266, 302. –Le Goff–Vitry et al. 2004: 170. –Zibrowius and Taviani 2005: 811. –Cairns 2006: 48. –Cairns et al. 2009: 345. –Kitahara and Cairns 2021: 355–356, 358, figs 190, 191A–C.
Pyrophyllia inflata Hickson, 1910: 1–7.
Guynia n. sp. sensu  Goreau and Wells 1967: 449.

###### Type locality.

Adventure Bank, Mediterranean; 168 m (Duncan, 1872).

###### Type material.

Eighteen syntypes are deposited at the BMNH (Cairns 1979).

###### Material examined.

**USNM 77201 (1 specimen)**: Eastern margin, 26 km from Port St. Johns/off Bulolo Estuary, 29°34'47.99"S, 31°41'59.99"E; 138 m.

###### Description.

Corallum solitary, small, vermiform to scolecoid, and usually attached by its side, but occasionally free. Calice cylindrical (GCD:LCD = 1.0–1.1), calicular margin smooth. Specimen examined (USNM 77201) 1.0 × 0.9 mm in CD and 5.2 mm in H. Epitheca ringed by imbricate transversal ridges, which meet with vertical costae giving a grid-like pattern along coralla. Corallum light brown.

Septa octamerally arranged in two cycles according to the formula: S_1_ > S_2_ (16 septa). S_1_ with narrow upper margin, which gradually widen deep in fossa with sinuous axial margin. S_2_ slightly smaller and bearing a less sinuous axial margin compare with S_1_. Septal faces smooth. Fossa shallow containing a single ribbon as columella.

###### Distribution.

Regional: Eastern margin of South Africa, off Port St. Johns, at 138 m. Elsewhere: Mozambique (Cairns and Keller 1993); Gulf of Oman (Hickson 1910); New Caledonia (Cairns 1989; Kitahara and Cairns 2021); Australia (Cairns and Parker 1992; Cairns 2004a); New Zealand; Philippines; Indonesia; Japan (Cairns and Zibrowius 1997); Wallis and Futuna region; Vanuatu (Cairns 1999a); Mediterranean (Duncan 1872; Zibrowius 1980); Gulf of Mexico; western Caribbean; Bermuda (Cairns 1979); Hawaiian Islands (Cairns 1984a); 28–653 m.

###### Remarks.

*Guyniaannulata* is the only extant representative of the family Guyniidae. The only other species assigned to the genus is a fossil collected from the Eocene and Oligocene, for which Cairns (1989a/b) considers the generic placement doubtful. Apart from the genus being monotypic, *G.annulata* has the smallest calicular diameter of all known scleractinian corals, and has a widespread tropical and sub-tropical distribution (Cairns and Parker 1992; Cairns and Keller 1993; Cairns and Zibrowius 1997). No new records of this cryptic species are reported herein and the description above is based on Cairns and Keller’s (1993) specimen collected off KwaZulu-Natal.

#### Family Micrabaciidae Vaughan, 1905

##### Letepsammia

Taxon classificationAnimaliaScleractiniaMicrabaciidae

Yabe & Eguchi, 1932c

E23DA941-BFF5-5EF2-9F2C-5D3CCF03E2A3

###### Diagnosis.

Corallum solitary, discoidal, and free. Synapticulothecate. Marginal shelf usually present. Costae thin and ridged. Intercostal spaces broader than costae and penetrated by large pores. Septa also highly porous, with complex dentition. Septa alternate in position with costae. Septa arranged in typical micrabaciid pattern, having multiple S_3_ bifurcations. Number of septa a function of calicular diameter, but 120 is the common adult number. Columella spongy.

###### Type species.

*Stephanophylliaformosissima* Moseley, 1876, by original designation.

##### Letepsammia formosissima

Taxon classificationAnimaliaScleractiniaMicrabaciidae

(Moseley, 1876)

6E5A1134-4E84-5EF4-8B48-D5499A57CC65

Fig. 14A, B

Stephanophyllia formosissima Moseley, 1876: 561–562. –Moseley 1877: 4. –Mosely 1881: 201–201, pl. 4, fig. 11, pl. 13, figs 6–7, pl. 16, figs 8, 9. –Vaughan 1907: 17, 23–24, 27, 28, 35, 38, 43–44, 146–147, 419, 426, pl. 44, figs 2, 2A. –Boschma 1923: 144–145, pl. 10, fig. 31. –Yabe and Eguchi 1932d: 61–63, pl. 8, figs 7, 8. –Eguchi 1934: 368. –Eguchi 1938: table 2. –Yabe and Eguchi 1942b: 107, 138, 139. –Crossland 1952: 92. –Wells 1958: 263, pl. 1, figs 1, 2. –Squires 1961:19. –Ralph and Squires 1962: 4, 16. –Uitnomi 1965: 249. –Zibrowius and Grygier 1985: 120. –Owens 1994: 586.
Stephanophyllia (Letepsammia) formosissima. –Yabe and Eguchi 1932b: 443.
Leptopenus discus . –Dennant 1906: 162.
Letepsammia formosissima . –Owens 1986b: 486–487. –Cairns 1984: 6–7. –Zibrowius and Grygier 1985: 120. –Cairns 1989b: 15–18, pl. 6, fig. J, pl. 7, figs G–I, pl. 8, figs A–D. –Cairns and Parker 1992, 8–9, pl. 1, figs F, H. –Cairns and Keller 1993: 230–231. fig. 3D. –Cairns 1994: 40–41, pl. 5, figs C, F. –Cairns and Zibrowius 1997: 73–75. –Cairns 1998: 371. –Cairns 1999: 59. –Cairns et al. 1999: 34. –Plusquellee et al. 1999: 998. –Riemann-Zurneck and Iken 2003: 383. –Cairns 2004a: 264, 271. –Cairns 2006: 47. –Cairns 2009: 2. –Janiszewska et al. 2011: 10. –Quattrini et al. 2020: 1538, fig. 2. –Seilitz et al. 2020: 6, fig. 1A–C . –Kitahara and Carins 2021: 55–56, figs 12I–L, 13.

###### Type locality.

Philippines and Indonesia (HMS ‘Challenger’ stns. 192 and 209: 5°49'12"S, 132°14'24"E, 10°14'00"S, 123°54'00"E, respectively); 174–236 m (Moseley 1876).

###### Type material.

Five syntypes are deposited at the BMNH (Cairns 1989a).

###### Material examined.

DEFF_NANSEN–INV 18: Eastern margin, 20 km from Durban/19 km off Beachwood Mangroves, 29°52'56.39"S, 31°12'15.59"E; 224 m. SAM_H1395 (1 specimen): Eastern margin, off Umdloti River mouth; 183 m. SAM_H1426 (1 specimen): Southern margin, off Great Fish River mouth; 183 m. SAM_H1429 (7 specimens): Southern margin, 6 km from Kidds Beach/5 km off Ncera Estuary, 33°11'59.99"S, 27°40'59.99"E; 79 m. SAM_H1452 (8 specimens): Locality data unknown. SAM_H1473 (2 specimens): Eastern margin, off Durban Harbor; 99 m. **USNM 91505 (1 specimen)**: Eastern margin, 26 km from Cape Vidal/25 km off St Lucia Estuary, 27°54'18.00"S, 32°37'59.87"E; 105 m.

###### Description.

Corallum discoidal (GCD:H = 3.5–3.7) with a flat to convex porous base, giving a low density to corallum. Largest specimen examined (SAM_H1426) 25.3 mm in CD and 7.0 mm in H. Calice circular, with serrated calicular margin. Costae ridged and thin, with closely packed granules resulting in serrated edges. Costal bifurcations correspond to septal pattern. Intercostal spaces porous, broader towards calicular margin and thinner towards epicentre of base. Synapticular bars connect each costa to neighbouring septa near calicular margin, and near epicentre of base synapticular bars connect adjacent costae. Marginal shelf low, reaching a maximum of 3 mm in width. Corallum white.

Up to 120 septa arranged in a typical micrabaciid fashion. S_1–2_ non-bifurcate and straight, with subsequent S_3_ leading to multiple bifurcations. S_1_ extend from calicular margin to columella with vertical axial margins. S_2_ also straight, extending from calicular margin to columella, but joined by S_3_ near columella. S_2–3_ fusion forms a delta bearing ≤ two spines. S_3_ bifurcates repeatedly. The first bifurcation produces two S_3_^i^ on either side of S_2_. The resultant edges of S_3_^i^ adjacent to S_2_ bifurcates three more times in which the first bifurcation produces one S_3_^ii^, second one S_3_^iii^, and the last two S_3_^iv^. The S_3_^i^ adjacent to S_1_ bifurcates four times, in which the first gives three S_3_^iii^ and two S_3_^iv^. Axial edge of S_1–2_ and sometimes S_3_ join the spongy columella.

###### Distribution.

Regional: Eastern margin of South Africa, off Great Fish River mouth extending towards Cape Vidal; 79–183 m. Elsewhere: Tanzania (Gardiner and Waugh 1939); Mozambique; Madagascar (Cairns and Keller 1993); Philippines; Indonesia (Cairns and Zibrowius 1997); Malaysia; Wallis and Futuna; Vanuatu; Australia; New Zealand (Cairns 1995, 1998;1999); Japan (Cairns 1994); 270–610 m.

###### Remarks.

*Letepsammiaformosissima* is one of the two species in the genus known from South African waters. It differs from the other reported species (*L.franki* Owens, 1994) in its coarser septal dentition. *Letepsammiaformosissima* was first reported in the region by van der Horst (1927) off Durban (towards St Lucia). Cairns (1989) subsequently alluded that three of van der Horst’s (1927) specimens were an undescribed species (USNM 82091). Later, Cairns and Keller (1993) also documented specimens resembling this undescribed species, which were later described as *L.franki* (Owens 1994).

**Figure 14.**
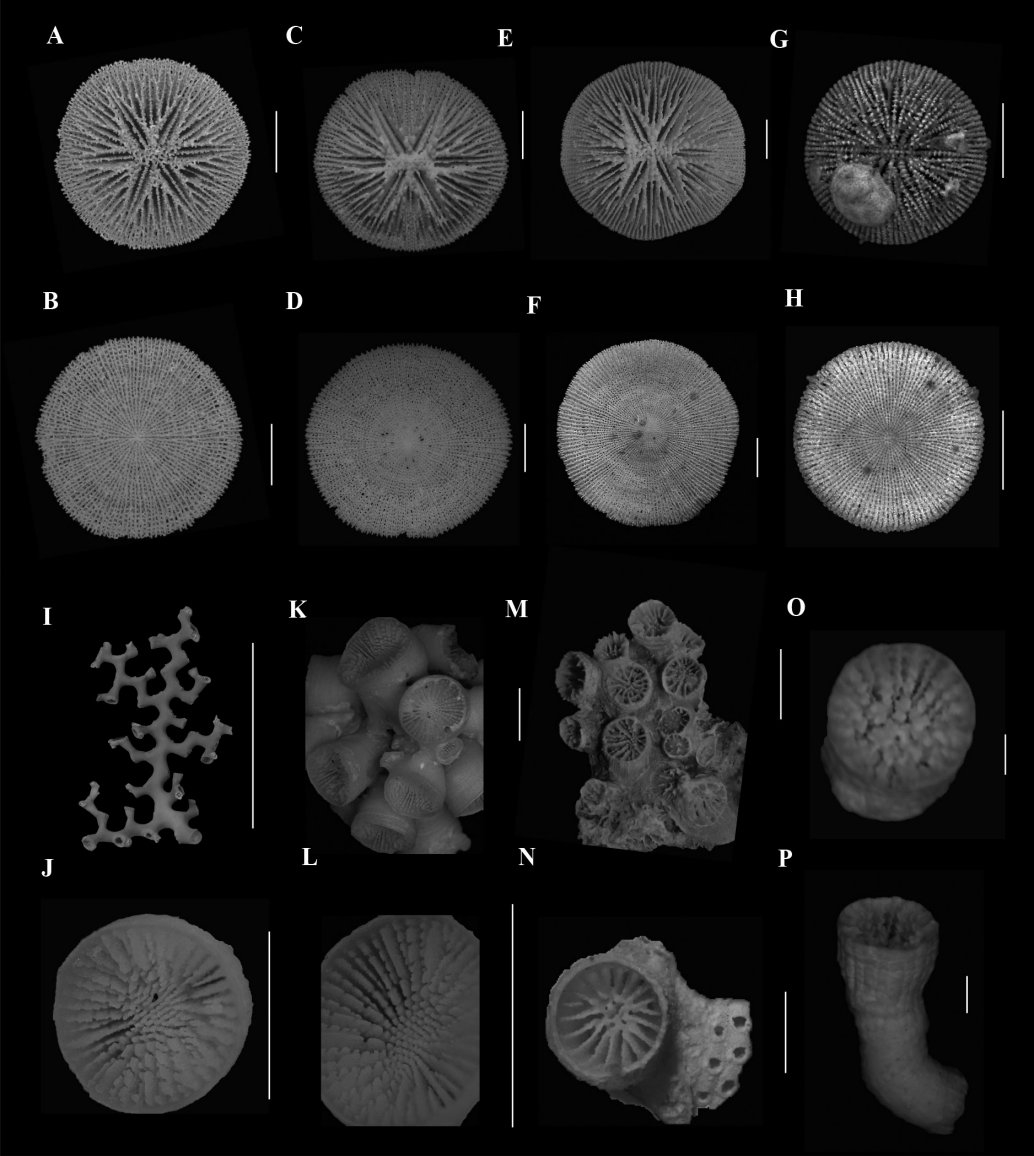
**A**, **B.***Letepsammiaformosissima* (SAM_H1429, off Kidds Beach, 79 m) **A** calicular view **B** basal view **C**, **D***Letepsammiafranki* (SAMC_A090070, off Durban, 95 m) **C** calicular view **D** basal view **E**, **F***Rhombopsammianiphada* (SAM_H1390, off Port Alfred, 90 m) **E** calicular view **F** basal view **G**, **H***Stephanophylliafungulus* (SAMC_A073106, Cape Vidal, 140 m) **G** calicular view **H** basal view **I***Madreporaoculata* (SAM_ H3038, off St Lucia, 825 m) full view **J**, **L**Culicia sp. cf. australiensis (SAMC_ A073032, off Shaka’s Rock, 50 m) **J** calicular view **K** full view **L** septa details **M**, **N***Culiciaexcavata.***M** (UCTES_DBN85H, off Ispongo, depth unknown): full view **N** (BMNH 1840.09.30.19, Cape of Good Hope, depth unknown): Calicular view **O**, **P***Stenocyathusvermiformis* (SAM_H3213, off Cintsa, 630 m) **O** calicular view **P** lateral view Scale bars: 10 mm (**A–H**, **J–N**); 100 mm (**I**); 2 mm (**O**, **P**).

##### Letepsammia franki

Taxon classificationAnimaliaScleractiniaMicrabaciidae

Owens, 1994

485D7B4E-3F1E-52DE-9AE3-4293B242B3CE

Fig. 14C, D

**Figure 15.**
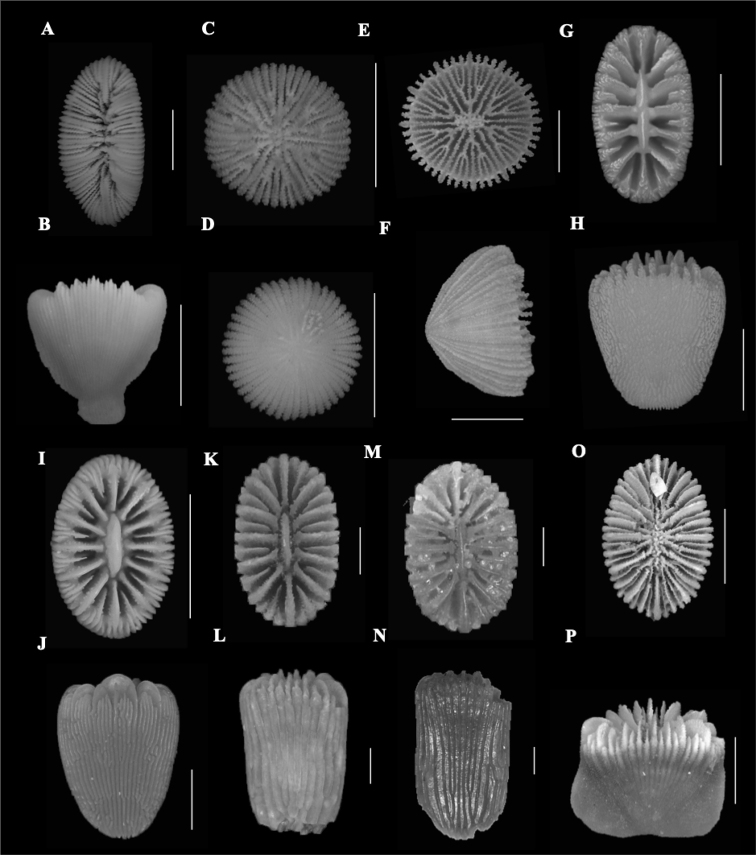
**A**, **B***Cyathotrochuspileus* (SAMC_A087424, off Durban, 270 m) **A** calicular view **B** lateral view **C**, **D***Deltocyathoidesorientalis* (BIVa2, locality unknown, 80) **C** calicular view **D** basal view **E**, **F***Deltocyathoidessentus* (USNM 91551, off Shaka’s Rock, 300 m) **G**, **H**Sphenotrochus (Eusthenotrochus) gilchristi (SAMC_A090086, off Cape Point, 24 m) **G** calicular view **H** lateral view **I**, **J**Sphenotrochus (Sphenotrochus) aurantiacus (SAM_ H1416, off the Agulhas, 366 m) **I** calicular view **J** lateral view **K**, **L**Sphenotrochus (Sphenotrochus) evexicostatus (SAMC_A090085, locality data unknown, 43 m) **K** calicular view **L** lateral view **M**, **N**Sphenotrochus (Sphenotrochus) imbricaticostatus (USNM 91715_Holotype, off Kosi-Kumpungwini (Sifungwe) Estuary, 44 m) **K** calicular view **L** lateral view **O**, **P***Tropidocyathuslessonii* (SAMC_ A073218, off Kosi Bay Estuary, 74 m) **O** calicular view **P** lateral view. Scale bars: 10 mm (**A–H**, **J–M**, **O–P**); 2 mm (**I**, **N**).

Stephanophyllia formosissima . –van der Horst 1927: 7. –Gardiner and Waugh 1939: 234. –Boshoff 1981: 24.
Letepsammia formosissima . –Cairns and Keller 1993: 218.
Letepsammia franki Owens 1994: 586–589, figs 1, 2. –Cairns 1999a: 59. –Seilitz et al. 2020: 2, fig. 2. –Kitahara and Cairns 2021: 57– 58, 60, figs 14, 15A–C.

###### Type locality.

Off Durban area, South Africa (RV ‘Anton Bruun’ stn. 390S: 29°35'00"S, 31°42'00"E); 138 m (Owens 1994).

###### Type material.

The holotype is deposited at the NMNH (Owens 1994).

###### Material examined.

ORI_BIVa1 (2 specimens): Eastern margin, Locality data unknown. SAMC_A073050 (14 specimens): Eastern margin, 26 km from Cape Vidal/25 km off St Lucia Estuary, 27°54'18.00"S, 32°37'59.87"E; 105 m. SAMC_A073151 (7 specimens): Eastern margin, 29 km from Durban/14 km off Mbokodweni Estuary, 30°06'24.12"S, 31°00'47.88"E; 160–170 m. SAMC_A073152 (7 specimens): Southern margin, 12 km from Gonubie/12 km off Gqunube Estuary, 33°01'48.00"S, 28°04'23.87"E; 85 m. SAMC_A073164 (1 specimen): Eastern margin, 28 km from Durban/14 km off Mbokodweni Estuary, 30°06'00.00"S, 31°01'36.00"E; 245–250 m. SAMC_A073175 (1 specimen): Eastern margin, 28 km from Coffee Bay/16 km off Hluleka Estuary, 31°55'58.79"S, 29°25'12.00"E; 300 m. SAMC_A073177 (1 specimen): Eastern margin, 19 km from Coffee Bay/18 km off Mdumbi Estuary, 32°02'53.87"S, 29°19'41.87"E; 250–280 m. SAMC_A073178 (4 specimens): Eastern margin, 28 km from Durban/14 km off Mbokodweni Estuary, 30°06'00.00"S, 31°01'36.00"E; 245–250 m. SAMC_A073185 (1 specimen): Eastern margin, 19 km from Durban/14 km off Mbokodweni Estuary, 30°00'36.00"S, 31°03'47.99"E; 140 m. SAMC_A087428 (1 specimen): Locality data unknown, SAMC_A090070 (1 specimen): Eastern margin, 20 km from Durban/16 km off Beachwood Mangrove, 29°50'12.12"S, 31°12'17.99"E; 95 m. SAMC_A090148 (1 specimen): Eastern margin, 30 km from Durban/31 km off Tongati Estuary, 29°43'11.99"S, 31°25'47.99"E; 185 m. SAM_H1364 (1 damaged specimen): Eastern margin, 2 km from Durban/8 km off Umgeni Estuary, 29°51'59.99"S, 31°00'00.00"E; 99 m. SAM_H1429 (1 specimen): Southern margin, 6 km from Kidds Beach/5 km off Ncera Estuary, 33°11'59.99"S, 27°40'59.99"E; 79 m. SAM_H1453 (1 specimen): Southern margin, 6 km from Kidds Beach/5 km off Ncera Estuary, 33°11'59.99"S, 27°40'59.99"E; 79 m. SAM_H3126 (2 specimens): Southern margin, 15 km from Port Alfred/11 km off Riet Estuary, 33°39'18.00"S, 27°01'05.99"E; 90 m. SAM_H3127 (3 specimens): Southern margin, 31 km from Port Alfred/20 km off Kleinemond (Oos) Estuary, 33°39'24.00"S, 27°11'42.00"E; 86 m. **USNM 75640 (7 specimens)**: Eastern margin, 26 km from Port St. Johns/off Bulolo Estuary, 29°34'47.99"S, 31°41'59.99"E; 138 m.

###### Imagery data.

BMNH 1939.07.20.401 (1 specimen): Locality data unknown. UCTES_NAD10B (6 specimens): Eastern margin, 29 km from Durban/22 km off Mdloti Estuary, 29°46'00.00"S, 31°16'59.99"E; 110–130 m. Mortensen-Java (4 specimens): Locality data unknown.

###### Description.

Corallum discoidal (GCD:H = 3.2–4.1), with a flat to convex and porous base. Closely packed septa, with coarse dentition giving the corallum a beaded appearance. Largest specimen examined (SAMC_A073164) 33.5 mm in CD and 8.0 mm in H. Calice circular, with serrated calicular margin. Costae ridged and thin, with closely packed low profile granules resulting in serrated costae. Costal bifurcations correspond to septal pattern. Intercostal spaces porous, thin at calicular margin and broaden towards epicentre of base. Synapticular bars connect each costa to neighbouring septa near calicular margin, and near epicentre of base synapticular bars connect adjacent costae. Marginal shelf low and narrow, reaching a maximum of 3 mm. Corallum white.

Septa arranged in a typical micrabaciid fashion reaching ≤ 120 septa. S_1–2_ non-bifurcate and straight, with subsequent S_3_ leading to multiple bifurcations. S_1_ imperforate and extend from columella to calicular margin. S_1_ with vertical and slightly dentated axial margin. S_2_ also straight and extending to columella, but joined by S_3_ near columella. S_2–3_ fusions form porous delta bearing numerous spines. S_3_ bifurcates repeatedly. First bifurcation produces two S_3_^i^ on either side of S_2_. Resultant edges of S_3_^i^ adjacent to S_2_ bifurcates three more times in which the first bifurcation gives S_3_^ii^, second one S_3_^iii^, and the last gives two S_3_^iv^. S_3_^i^ adjacent to S_1_ bifurcates four times, in which the first gives three S_3_^iii^ and two S_3_^iv^. Axial edge of S_1–2_ and sometimes S_3_, join the spongy columella.

###### Distribution.

Regional: Southern to eastern margin of South Africa, from off Port Alfred extending towards Cape Vidal; 79–300 m. Elsewhere: Off Pemba, Tanzania (Gardiner and Waugh 1939); 50–650 m (Owens 1994).

###### Remarks.

*Letepsammiafranki* was first reported in the region as *Stephanophylliaformosissima*. This misidentification, together with Boshoff’s (1981) (ORI_BIVa1), was noted by Cairns (1989a) and Cairns and Keller (1993). However, the undescribed species was named a year later by Owens (1994), who emphasiszed the difference between *L.franki* and *L.formosissima* to be the closely packed and dentate septa, which gave *L.franki* a more compact and beaded appearance. Another notable difference between the two species is that S_1_ are imperforate in *L.franki* and perforate in *L.formosissima*.

##### Rhombopsammia

Taxon classificationAnimaliaScleractiniaMicrabaciidae

Owens, 1986

2EF4D32F-C4B4-5FF7-AC31-F5386F2BF5F5

###### Diagnosis.

Corallum solitary, discoidal, and free. Synapticulothecate. Broad marginal shelf present. Costae ridged, thin and dentate. Intercostal spaces broader than costae and penetrated by large pores. Septa imperforate, with complex dentitions. Septa alternate in position with costae. Septa arranged in typical micrabaciid pattern, having multiple bifurcations of S_3_; number of septa a function of calicular diameter, varying between 99 and 144 in adult stage. Columella spongy.

###### Type species.

*Rhombopsammiasquiresi* Owens, 1986a, by original designation.

##### Rhombopsammia niphada

Taxon classificationAnimaliaScleractiniaMicrabaciidae

Owens, 1986

C16FA295-FB81-5BE7-8DC5-74F88E06A020

Fig. 14E, F

Rhombopsammia niphada Owens, 1986a: 252–255, figs 2B, 3A–D. –Cairns 1989a: 19–20, text-fig. 2, pl. 9, figs D–I, pl. 10, figs A, B. –Cairns 1994: 41, pl. 15, figs I–K, pl. 16, figs A, B. –Owens 1994: 588. –Cairns and Zibrowius 1997: 75–76. –Cairns 1998: 371. –Cairns et al. 1999: 34. –Plusquellec et al. 1999: 998. –Cairns 2004a: 271. –Cairns 2009: 2. –Kitahara et al. 2010b. –Janiszewska et al. 2011. –Quattrini et al. 2020: 1538, fig. 1. –Seilitz et al. 2020: 11. –Kitahara and Cairns 2021: 64–65, 67, figs 15D, H, 18, 19A–D.

###### Type locality.

Off Kyushu (USS ‘Albatross’ stn. 4911: 31°38'00"N, 129°19'00"E); 715 m (Owens 1986a; Cairns 1989a).

###### Type material.

Types are deposited at the NMNH (Cairns 1989a).

###### Material examined.

SAM_H1390 (1 specimen): Southern margin, 28 km from Port Alfred/3 km off Old Woman’s Estuary, 33°30'00.00"S, 27°08'59.99"E; 90 m. SAM_H1453 (3 specimens): Southern margin, 6 km from Kidds Beach/5 km off Ncera Estuary, 33°11'59.99"S, 27°40'59.99"E; 79 m. SAM_H3126 (2 specimens): Southern margin, 15 km from Port Alfred/11 km off Riet Estuary, 33°39'18.00"S, 27°01'05.99"E; 90 m. SAM_H3128 (1 specimen): 15 km from Port Alfred/11 km off Riet Estuary, 33°39'18.00"S, 27°01'36.00"E; 90 m.

###### Description.

Corallum discoidal (GCD:H = 3.1–3.9), with a flat to slightly concave or convex base. Septa imperforate, solid, and dentate. Largest specimen examined (SAM_H1453) 22.7 mm in CD and 5.8 mm in H. Calice circular, with finely serrated edges. Costae ridged and thin, with closely packed low profile granules resulting in serrated costae. Costal bifurcation alternates with septal pattern. Intercostal spaces porous, broader than costae, decreasing towards epicentre of base. Synapticular bars connect two costae to common adjacent septum, resulting in pores that also decrease in size towards epicentre of base. Septa gradually slope towards calicular margin, producing a narrow marginal shelf, which reaches a maximum of 2 mm. Corallum white.

Septa arranged in typical micrabaciid fashion, with ≤ 144 septa. S_1–2_ non-bifurcate and straight, with subsequent S_3_ leading to multiple bifurcations. S_1_ extend to columella with complex and uniform dentitions along septal margin, and bearing vertical vepreculae on lateral faces. S_2_ also straight and extending to columella, but joined by S_3_ near columella. Resulting S_2–3_ fusion forming a delta with numerous spines. S_3_ bifurcates repeatedly. First bifurcation produces two S_3_^i^ on either side of S_2_. Resultant edges of S_3_^i^ adjacent to S_2_ bifurcates three more times in which the first branching gives S_3_^ii^, second one S_3_^iii^, and last gives two S_3_^iv^. S_3_^i^ adjacent to S_1_ bifurcates six times, in which the first gives two S_3_^iii,^ three S_3_^iv,^ and two S_3_^iv^. All septa are predominantly solid, with S_3_ being perforated at base and at bifurcations. Axial edge of S_1–2_ and sometimes S_3_, join the spongy columella.

###### Distribution.

Regional: Southern margin of South Africa, off Port Alfred extending towards Kidds Beach; 79–90 m. Elsewhere: Philippines; Indonesia (Cairns 1989a; Cairns and Zibrowius 1997); Japan (Owens 1986a); 424–852 m.

###### Remarks.

The material examined herein represents a new record of *Rhombopsammianiphada* for South Africa, but adds no taxonomic information to what is already known about the species’ morphology. *Rhombopsammianiphada* may be mistaken for *Letepsammiaformosissima*, which is also found in South Africa, but differs in having predominantly solid septa, and in S_1_ septal faces bearing vepreculae, as compare with porous septa and smooth S_1_ septal faces in *L.formosissima* (Cairns 1989a; Cairns and Zibrowius 1997).

##### Stephanophyllia

Taxon classificationAnimaliaScleractiniaMicrabaciidae

Michelin, 1841

417F57D2-029E-54E4-904B-B2A6D2E1BC34

###### Diagnosis.

Corallum solitary, discoidal, and free. Synapticulothecate. A small marginal shelf may be present. Costae granular. Intercostal spaces broader than costae and penetrated by large pores. Septa imperforate, totalling 96 that alternate in position with costae. Septa arranged in typical micrabaciid pattern, having multiple bifurcations of the S_3_. Septa and costae interconnected by elongate, bar-shaped synapticulae (fulturae). Columella lamellar to papillose.

###### Type species.

*Fungiaelegans* Bronn, 1837, by original designation.

##### Stephanophyllia fungulus

Taxon classificationAnimaliaScleractiniaMicrabaciidae

Alcock, 1902

67117A06-A636-5E28-A4AD-A41B3DCBBA6F

Fig. 14G, H

Stephanophyllia complicata . –Alcock 1902a: 40 (in part: 1 of 3 specimens from ‘Siboga’ 59). –Moseley 1876.
Stephanophyllia fungulus Alcock, 1902b: 122–123. –Alcock 1902c: 40, pl. 5, fig. 35A, B. –Gardiner and Waugh 1939: 234. –Pillia and Scheer 1976: 14. –Cairns 1989b: 21–23, pl. 10, figs A–K, pl. 11, figs A–B. –Cairns and Keller 1993: 231. –Cairns 1994: 41–42, pl. 16, figs A–D, F, G. –Kitahara and Cairns 2021: 70, 72, figs 21, 22A–H.
Micrabacia fungulus . –Vaughan and Wells 1943: 312, pl. 20, fig. 1A, B.

###### Type locality.

Sulu Archipelago (HMS ‘Siboga’ stn. 100: 6°11'00"N, 120°37.5'00"E); 450 m (Alcock 1902b; Cairns 1989).

###### Type material.

Five syntypes are deposited at the ZMA (Cairns 1989a; Cairns and Zibrowius 1997).

###### Material examined.

SAMC_A073050 (1 specimen): Eastern margin, 26 km from Cape Vidal/25 km off St Lucia Estuary, 27°54'18.00"S, 32°37'59.87"E; 105 m. SAMC_A073106 (1 specimen): Eastern margin, 66 km from Cape Vidal/7 km off Mgobezeleni Estuary, 27°33'11.88"S, 32°43'00.12"E; 140 m. SAMC_A073139 (1 specimen): Eastern margin, 35 km from Cape Vidal/32 km off St Lucia Estuary, 27°49'41.87"S, 32°38'12.11"E; 54 m.

###### Description.

Corallum discoidal (GCD:H = 2.6–2.9), robust, and bearing a flat to slightly concave or convex base. Largest specimen examined (SAMC_A073106) 12.9 mm in CD and 4.5 mm in H. Calice circular, with finely serrated calicular margin. Costae equidistant with rounded edges. Costal bifurcation alternates with septal pattern. Intercostal spaces porous, narrow near base epicentre but broadens towards calicular edge. Synapticular bars connect two costae to common septum between them. No marginal shelf. Corallum white to creamy.

Septa arranged in typical micrabaciid fashion and total ≤ 96 septa. S_1–2_ non-bifurcate and straight, with subsequent S_3_ leading to multiple bifurcations. S_1_ extend to columella. S_2_ also extend to columella, but joined by S_3_ near columella. At the fusing point between S_2–3_ a delta with numerous spines is formed. S_3_ bifurcates repeatedly. First bifurcation produces two S_3_^i^ on either side of S_2_. Resultant edges of S_3_^i^ adjacent to S_2_ bifurcates three more times in which first branching gives S_3_^ii^, second one S_3_^iii^, and last gives two S_3_^iv^. S_3_^i^ adjacent to S_1_ bifurcates twice, in which first gives one S_3_^ii^, and two S_3_^iii^. Septa perforate at near base and at points of bifurcation. All septal margins straight. Septal faces bear granules shaped as small triangular spines. Columella lenticular and aligned with two S_1_.

###### Distribution.

Regional: Eastern margin of South Africa, off Cape Vidal; 54–150 m. Elsewhere: Off south-eastern Mozambique; Chagos Archipelago (Gardiner and Waugh 1939); Maldives (Pillar and Scheer 1976); Philippines; Indonesia (Cairns 1989a; Cairns and Zibrowius 1997); 15–635 m.

###### Remarks.

*Stephanophylliafungulus* is one of the four known species in the genus and was first reported in the region by Cairns (1989a), who detailed the morphological differences among them.

#### Family Oculinidae Gray,1847

##### Madrepora

Taxon classificationAnimaliaScleractiniaOculinidae

Linnaeus, 1758

78FDDA56-18E8-5BFB-94E5-931C3E81C8FB

###### Diagnosis.

Colonial, extra-tentacular sympodial budding forming dendroid colonies. Coenosteum dense. Costae and pali sometimes absent. Columella papillose or absent.

###### Type species.

*Madreporaoculata* Linnaeus, 1758, by subsequent designation (Verrill 1901).

##### Madrepora oculata

Taxon classificationAnimaliaScleractiniaOculinidae

Linnaeus, 1758

D19C6999-E9C3-5972-A559-0AE1576E1FB6

Fig. 14I

Madrepora oculata Linnaeus, 1758: 798. –Esper 1791: 108, pl. 12, figs 1–3. –von Marenzeller 1904b: 79. –Durham and Barnard 1952: 11. –Squires 1959a: 5. –Eguchi 1968: C29, pl. C8, figs 1–9. –Best 1970: 298, fig. 2. –Bourcier and Zibrowius 1973: 826, figs 6, 7. –Zibrowius 1974a: 761–766, pl. 2, figs 3–5. –Zibrowius 1980: 36–40, pl. 13, figs A–P. –Zibrowius and Grieshaber 1977: 377. –Cairns 1979: 39–42, pl. 3, fig. 2, pl. 4, fig. 5, pl. 5, figs 1–3. –Zibrowius 1979: 21. –Zibrowius 1980: 36–40, pl. 13, figs A–P. –Cairns 1982: 15, pl. 3, figs 4–6. –Cairns 1984: 10, pl. 1, fig. H. –Cairns 1991a: 9–10, pl. 2, fig. J, pl. 3, figs A–D. –Cairns and Keller 1993: 233. –Cairns 1994: 18–19, pl. 3, figs F–H. –Cairns 1995: 41, pl. 5, figs C–F, pl. 6, figs A, B. –Cairns and Zibrowius 1997: 79–80. –Cairns 1998: 372–374, fig. 1F–L. –Cairns 1999: 61, fig. 2E, F. –Cairns et al. 1999: 35. –Cairns 2004a: 274–275. –Le Goff–Vitry et al. 2004: 171, 173. –Kitahara 2006: 58, fig. 2A, B. –Kitahara 2007: 500–501, fig. 2G. –Cairns 2009: 4. –Kitahara et al. 2010b. –Miller et al. 2010, 3–5, 7, 9–10, 12. –Kitahara and Cairns 2021: 447–449, figs 241G–I, 243.
Amphihelia oculata . –Milne-Edwards and Haime 1857: 119. –Duncan 1873: 326, pl. 45, figs 1–3. –Alcock 1902c: 35. –von Marenzeller 1904a: 308–310, pl. 14, fig. 1. –Gravier 1920: 89, pl. 10, fig. 158–164.
Amphihelia ramea . –Duncan 1873: 326, pl. 44, figs 1–3, pl. 45, figs 4–6, pl. 46, figs 1–19. –Jordon 1895: 26. –Alcock 1902c: 35.
Lophohelia candida . –Moseley 1881: 179–180, pl. 9, figs 6–13.
Lophohelia tenuis . –Moseley 1881: 180–181, pl. 8, figs 11–14. –Bourne 1903: 26.
Cyathohelia formosa Alcock, 1898: 26–27, figs 2, 2A.
Lophohelia investigatoris Alcock, 1898: 24–25.
Desmophyllum sp. –Alcock 1902c: 28.
Madrepora kauaiensis Vaughan, 1907: 83–81, pl. 8, figs 1–2. –Gardiner and Waugh 1939: 227. –Crossland 1952: 121. –Wells 1964: 109. –Veron 1986: 599. –Cairns 2006: 47.
Amphihelia sp. –Gardiner 1913: 689.
Madrepora tenuis . –Faustino 1927: 107–108, pl. 14, figs 2, 5.
Madrepora alcocki Faustino, 1927: 106.
Madrepora formosa . –Zibrowius 1974b: 568–570, figs 6–9.
Lophelia exigua . –Boshoff 1981: 37.
Madrepora sp. –Veron 1986: 599.

###### Type locality.

Tyrrhenian Sea and Sicily, Mediterranean; depth unknown (Cairns and Zibrowius 1997).

###### Type material.

Types are lost (Zibrowius 1980).

###### Material examined.

ORI_DIIIf1 (1 fragment): Locality data unknown. SAM_H3038 (5 fragments): Eastern margin, 17 km from St. Lucia Estuary/16 km off Mfolozi Estuary, 28°21'53.99"S, 32°34'36.00"E; 775–825 m. SAM_H3039 (10 fragments): Eastern margin, 36 km off Port Shepstone/49 km off Mtentu Estuary, 30°43'11.99"S, 30°48'47.99"E; 900 m. SAM_H3040 (1 fragments): Southern margin, 36 km from Port Shepstone/29 km off Mhlabatshane Estuary, 30°43' 11.99" S, 30°48'47.99"E; 780 m.

###### Description.

Corallum colonial, with delicate distal branches. Colonies usually bushy but distal branches uniplanar, with sympodially arranged corallites. Corallites circular, reaching ≤ 3.0 mm in CD, with slightly serrated calicular margin. Costae thin and prominent near calicular edge, usually corresponding to higher cycle septa. Coenosteum finely granular, longitudinally striated. Corallum light beige to white.

Septa thin and hexamerally arranged in three complete cycles according to the formula: S_1_ > S_2_ > S_3_ (24 septa). S_1_ highly exsert, extending towards columella deep in fossa with dentate and sometimes laciniate axial margin. S_2_ less exsert, half the width of S_1_, and have dentate axial edges. S_3_ rudimentary and discontinuous. Fossa deep, containing a poorly developed papillose columella.

###### Distribution.

Regional: Southern and Eastern margins of South Africa, off Still Bay (Boshoff 1981) extending towards St Lucia; 780–900 m. Elsewhere: Cosmopolitan, apart from Antarctica (Alcock 1898; von Marenzeller 1904a; Gardiner and Waugh 1938; Zibrowius 1980; Cairns 1979, 1982, 1984; Cairns and Keller 1993; Cairns 2004a); 15–1950 m.

###### Remarks.

*Madreporaoculata* is one of the most well-studied framework building azooxanthellate coral species, and has been reported worldwide (apart from Antarctica). The first record from South Africa was identified as *Lopheliaexigua* Pourtalès, 1871 (Boshoff 1981) (ORI_DIIIf1), but two localities were listed, making it unclear where the sample was collected. Nonetheless, both of the stations fall within the southern margin of South Africa. One of the examined specimens (SAM_H3038) is attached to hexactinellid sponge spicules

#### Family Rhizangiidae d’Orbigny, 1851

##### Culicia

Taxon classificationAnimaliaScleractiniaRhizangiidae

Dana, 1846

80ADA16A-4DBA-51F8-B746-A3397E721C3B

###### Diagnosis.

Corallum colonial and consisting of low cylindrical corallites linked together by stolons. Corallites epithecate. S_1_ weakly dentate or lobate. Higher cycle septa finely dentate. Pali absent. Columella rudimentary.

###### Type species.

*Culiciastellata* Dana, 1846, by subsequent designation (Wells 1936).

##### Culicia sp. cf. australiensis

Taxon classificationAnimaliaScleractiniaRhizangiidae

Hoffmeister, 1933

06F0A0DF-EE1D-581E-9CC9-452AF645EDAD

Fig. 14J–L

Culicia australiensis Hoffmeister, 1933: 12, pl. 3, figs 3, 4. –Wells 1958: 263, pl. 1, figs 3, 4. –Squires 1960: 200, fig. 8. –Cairns and Parker 1992: 12–13, figs 2A, D, G. –Cairns 1998: 371–372. –Cairns 2004a: 273.
Culicia sp. Veron, 1986: 600.

###### Type locality.

Off Marsden Point, South Australia, 31 m (Cairns 2004a).

###### Type material.

Syntypes are deposited at the AM (Cairns 2004a).

###### Material examined.

SAMC_A073032 (2 colonies): Eastern margin, 9 km from Shaka’s Rock/12 km off Mhlali Estuary, 29°32'06.00"S, 31°19'47.99"E; 50 m.

###### Imagery data.

Mortensen Stn. 23 (1 colony): Eastern margin, off Durban; 64 m. Mortensen Stn. 30 (1 colony): Eastern margin, off Durban; 94 m. SAM_H1236 (1 colony): Eastern margin, O’Neil Peak; 101 m.

###### Description.

Colony reptoid, composed of elongated individual corallites joined by stolons. Corallites cylindrical, with circular to elliptical calices (GCD:LCD = 1.0–1.1), and ≤ 6.8 mm in H. Calicular margin smooth. Epitheca smooth and thin.

Septa hexamerally arranged in four cycles according to the formula: S_1–2_ > S_3_ > S_4_ (48 septa). S_1–2_ equal in width and bearing variable axial margin: sometimes smooth and in other cases dentate, both types extend to columella. S_3_ equal to or ^1^/_3_ smaller than S_1–2_, and bear a dentate axial margin. S_4_ rudimentary, also having dentate axial margin. All septa non-exsert and closely packed. Fossa moderately deep containing a papillose columella composed of granulated rods.

###### Distribution.

Regional: Eastern margin of South Africa, from off O’Neil Peak extending towards Durban; 50–101 m. Elsewhere: Australia (Hoffmeister 1933; Cairns and Parker 1992; Cairns 1998, 2004a); 3–378 m.

###### Remarks.

Three other taxa (*C.tenellatenella* Dana, 1846, *C.tenellanatalensis* (Duncan, 1876), and *C.excavata* (Milne-Edwards & Haime, 1849)) are historically known to occur in the south-west Indian Ocean, all of which have three septal cycles, not four. Specimens examined most closely resemble *C.australiensis* in all characters (including a hexamerally arranged septa with four cycles), but differ in having a dentated, not smooth S_1_ axial margin. However, *Culicia* requires revision and, therefore, the examined specimens are tentatively reported as C. sp. cf. australiensis until the time that the taxonomy of this genus is reviewed.

##### Culicia excavata

Taxon classificationAnimaliaScleractiniaRhizangiidae

(Milne-Edwards & Haime, 1849)

358E2D94-B6D7-5384-B3FE-7A322F69B6F0

Fig. 14M, N

Angia excavata Milne-Edwards & Haime, 1849: 177.
Culicia tenella . –Boshoff 1981: 25.

###### Type locality.

Cape of Good Hope, South Africa.

###### Type material.

The holotype is deposited at the BMNH.

###### Material examined.

None.

###### Imagery data.

BMNH 1840.09.30. 19 (Type: 1 colony): Cape of Good Hope. UCTES_DBN 85 H (1 colony): Eastern margin, off Isipingo, depth unknown.

###### Description.

Corallum colonial, reptoid, with short, cylindrical, and small individual corallites connected by stolons. Calice circular. Calicular margin smooth.

Septa hexamerally arranged in three cycles according to the formula: S_1–2_ ≥ S_3_ (24 septa). S_1–2_ equal in size, convex, and extend to an under-developed columella (if present) with slightly dentated axial margins. S_3_ rudimentary and curved towards S_2_ with dentate axial margins. Septa loosely packed. Pali absent. Columella rudimentary or absent. Fossa shallow.

###### Distribution.

Regional: Eastern margin of South Africa, off Durban; intertidal–138 m. Elsewhere: Mozambique; depth unknown (Boshoff 1981).

###### Remarks.

Subsequent to the original description of *Culiciaexcavata*, this species was misidentified and reported as *C.tenella* by Boshoff (1981). After close examination of the Boshoff’s specimen (collected off Mozambique: ORI_CIc1, 3 specimens), we confirm these specimens to be *C.excavata*. The occurrence of Boshoff’s East London record is not clear and should not be considered in biodiversity assessments. Nonetheless, more specimens of the cryptic *C.excavata* are required and the revision of the genus is urgently needed.

##### Culicia

Taxon classificationAnimaliaScleractiniaRhizangiidae

sp. cf. tenellanatalensis (Duncan, 1876)

1711AA74-071D-525B-B07D-94CDB7839807

Cylicia tenella . –Milne-Edwards and Haime 1857: 608.
Cylicia tenella var. natalensis Duncan, 1876: 439–440, pl. 40, fig. 3.
Culicia tenella . –Gardiner and Waugh 1939: 230.
Culicia sp. cf. C.natalensis. –Cairns and Keller 1993: 232–232, fig. 3G.
Culicia tenella natalensis . –Cairns 2004a: 274.

###### Type locality.

Cape of Good Hope, South Africa (Cairns 2004a).

###### Type material.

The holotype is deposited at the NHMUK (Cairns 2004a).

###### Material examined.

None.

###### Distribution.

Regional: Eastern margin of South Africa, off KwaZulu-Natal; depth unknown (Duncan 1876). Elsewhere: Tanzania (Gardiner and Waugh 1938); Kenya (Cairns and Keller 1993); 34 m.

###### Remarks.

This entry is based on Duncan’s (1876) subspecies *Culiciatenellanatalensis*. In his account, Duncan (1876) highlighted the association between *C.natalensis* and a brachiopod. Although we herein doubt the validity of Duncan’s South African subspecies, more specimens are required in order to compare *C.natalensis* to the Australian *C.tenella* and *C.hoffmeisteri* Squires, 1966. The latter is an Australian species that Cairns and Keller (1993) reported to have a close resemblance to their *C.natalensis* examined specimens from Kenya.

#### Family Stenocyathidae Stolarski, 2000

##### Stenocyathus

Taxon classificationAnimaliaScleractiniaStenocyathidae

Pourtalès, 1871

ADBA8CEC-A5F2-5932-8417-AA8FC388D749

###### Diagnosis.

Corallum solitary, ceratoid to cylindrical, free or attached. Wall epithecal with rows of thecal spots (pores) flanking each S_3_. Pali, if present, opposite S_2_. Columella composed of one or two twisted, crispate ribbons.

###### Type species.

*Coenocyathusvermiformis* Pourtalès, 1868, by monotypy.

##### Stenocyathus vermiformis

Taxon classificationAnimaliaScleractiniaStenocyathidae

(Pourtalès, 1868)

AB400137-1CF5-50FE-814C-878D6EE4CDC7

Fig. 14O, P

Coenocyathus vermiformis Pourtalès, 1868: 133–134.
Stenocyathus vermiformis . –Pourtalès 1871: 10, pl. 1, figs 1, 2, pl. 3, figs 11–13. –Pourtalès 1878: 202. –Pourtalès 1880: 96, 101 (in part), pl. 1, figs 15, 16. –Duncan 1883: 368. –Agassiz 1888: 148, fig. 483. –von Marenzeller 1904a: 298–300, pl. 18, fig. 16. –Gravier 1915: 2. –Gravier 1920: 30–32, pl. 3, figs 35–37, pl. 13, figs 193–197. –Gardiner and Waugh 1938: 172. –Wells 1947: 167, pl. 10, figs 1–5. –Wells 1958: 262. –Squires 1959a: 23. –Zibrowius 1969: 328. –Laborel 1970: 153. –Zibrowius 1971: 244. –Cairns 1977b: 5. –Cairns 1978: 11. –Cairns 1979: 168–170, pl. 32, figs 8–10, pl. 33, figs 1, 2. –Zibrowius 1980: 163–165, pl. 84, figs A–Q. –Cairns 1982: 52, pl. 16, figs 8–11. –Cairns 1984: 23, 25, pl. 5, fig. C. –Cairns et al. 1991: 48. –Cairns and Parker 1992: 43, fig. 14B, C. –Cairns and Keller 1993: 273, fig. 12E, F. –Cairns 1994: 69–70, pl. 22, fig. G, pl. 29, figs C, F. –Cairns 1995: 94–95, pl. 30, figs C–G. –Cairns et al. 1999: 33. –Cairns 2000: 151–153, fig. 178. –Cairns 2004a: 302. –Cairns 2006: 48. –Kitahara 2007: 504, 505, 511, 512, fig. 5F. –Cairns 2009: 22. –Kitahara et al. 2010b. –Kitahara and Cairns 2021: 559–561, figs 308D–F, 309.
Caryophyllia simplex Duncan, 1878: 237, pl. 43, figs 32–34.
Caryophyllia carpenteri Duncan, 1878: 237, pl. 43, figs 28–31.

###### Type locality.

Off Florida Keys, United States; 274–329 m (Cairns 1979).

###### Type material.

Syntypes are deposited at the MCZ (Cairns 1979).

###### Material examined.

SAM_H1699 (2 specimens): Eastern margin, 20 km from Cape Vidal/23 km off St Lucia Estuary, 27°59'30.00"S, 32°40'47.99"E; 550 m. SAM_H3212 (1 specimen): Southern margin, 46 km from Port Alfred/12 km off Mgwalana Estuary, 33°29'24.00"S, 27°21'11.99"E; 80 m. SAM_H3213 (2 specimens): Southern margin, 40 km from Cintsa/29 km off Cwili Estuary, 32°54'59.99"S, 28°30'59.99"E; 630 m. SAM_H3214 (1 specimen): Southern margin, 32 km off Mazeppa Bay/24 km off Kobole Estuary, 32°28'36.00"S, 28°58'48.00"E; 710–775 m.

###### Description.

Corallum solitary, small, vermiform, usually free, but occasionally attached. Calice cylindrical to sub-cylindrical (GCD:LCD = 1.0–1.4), calicular margin smooth. Largest specimen examined (SAM_H1699) 4.5 × 4.4 mm in CD and 20.0 mm in H. Theca thin, marked with transverse ridges extending from calicular edge to base, and white spots aligned in 24 longitudinal rows. Costae absent. Corallum white with light brown theca.

Septa hexamerally arranged in three cycles according to the formula: S_1_ > S_3_ ≥ S_2_ (24 septa). S_1_ extend ¾ distance to columella. S_2_ half the width of S_1_, and bear a pali. S_3_ slightly wider or equal to S_2_. All septa have sinuous axial edges, and granules arranged perpendicular to septal margin. Pali also granular and sinuous. Columella fascicular in a shallow to moderately deep fossa.

###### Distribution.

Regional: Southern to eastern margins of South Africa, from off Cintsa extending to Cape Vidal; 80–775 m. Elsewhere: Walters Shoal; St Paul and Amsterdam Islands (von Marenzeller 1904a; Zibrowius 1974a; Cairns and Keller 1993); New Zealand (Cairns 1995); Australia (Cairns 2004a); New Caledonia (Kitahara and Cairns 2021); Mediterranean Sea (Zibrowius 1980); from off the United States (Pourtalès 1868; Cairns 1979) to Brazil (Kitahara 2007); and Antarctica (Cairns 1982); 80–1500 m.

###### Remarks.

Specimens of *S.vermiformis* examined herein represent new records for the region, thus extending the previously known distribution from south of Madagascar towards South Africa. Furthermore, this is the only species of the family representative in the region and is distinctive in its small vermiform corallum, three septal cycles, and presence of thecal spots aligned in rows. Molecular evidence suggests that *S.vermiformis* groups with Caryophylliidae (Kitahara et al. 2010b) and thereby supports the hypothesis that thecal pores originated independently in different scleractinian lineages (Stolarski 2000). Nonetheless, additional work on the placement of the other species representatives of the family needs to be undertaken.

#### Family Turbinoliidae Milne-Edwards & Haime, 1848

##### Cyathotrochus

Taxon classificationAnimaliaScleractiniaTurbinoliidae

Bourne, 1905

607B1776-0123-55C2-B367-9B0C055F68F6

###### Diagnosis.

Corallum cuneiform, with rounded base and calice elliptical in cross section. GCD ≤ 25.0 mm. Costae highly ridged, independent in origin, and serrate in ornamentation. Intercostal region equal to costae in width, not pitted, and quite deep. Septa highly exsert and hexamerally arranged in four or five cycles (48–96 septa). Lamellar pali in three crowns before all but last septal cycle (P_1–3_ or P_1–4_), higher cycle pali arranged in chevrons. Columella papillose to sub-lamellar.

###### Type species.

*Cyathotrochusherdmani* Bourne, 1905 by monotypy.

##### Cyathotrochus pileus

Taxon classificationAnimaliaScleractiniaTurbinoliidae

(Alcock, 1902)

59B914D5-64B3-5D2D-B48E-ABF0FE32B28D

Fig. 15A, B

Endopachys australiae Tenison-Woods, 1878: 333, pl. 6, fig. 1A–C.
Tropidocyathus bougainvillea Milne-Edwards & Haime, 1857: 57.
Trochocyathus pileus Alcock, 1902a: 96–97. –Alcock 1902c: 15–16, pl. 2, figs 11, 11A. –Faustino 1927: 8, 34, 39, 81. –Gardiner and Waugh 1938: 187. –Yabe and Eguchi 1942b: 106, 123.
Tropidocyathus pileus . –Cairns 1989a: 34–35, pl. 17, figs A–H. –Cairns 1994: 68, pl. 29, figs D, E. –Cairns 1995: 91, pl. 28, figs A–C. –Cairns and Zibrowius 1997: 147–148, fig. 19H, I.
Cyathotrochus pileus . –Cairns 1997: 16, pl. 1, figs F–G, pl. 4, fig. F. –Cairns 1998: 392.– Cairns 1999: 110–111. –Cairns et al. 1999: 40. –Cairns 2004a: 292, figs 6D, E. –Kitahara et al. 2010b: 9. –Kitahara and Cairns 2021: 95–96, 98, figs 36H, 37, 38A–C.

###### Type locality.

Sulu Archipelago, Philippines (HMS ‘Siboga’ stn. 95: 5°43'00"N, 119°40'00"E); 522 m (Cairns 1994).

###### Type material.

Four syntypes are deposited at the ZMA (Cairns 1994).

###### Material examined.

SAMC_A073181 (2 specimens): Eastern margin, 11 km from Port St. Johns/10 km off Bulolo Estuary, 31°43'54.12"S, 29°32'12.11"E; 190 m. SAMC_A087424 (1 specimen): Eastern margin, 19 km from Durban/18 km off Beachwood Mangroves, 29°53'24.00"S, 31°11'12.11"E; 270 m.

###### Description.

Corallum cuneiform, laterally compressed, with a rounded base. Thecal edge crests absent. Calice elliptical (GCD:LCD = 1.7–1.9), calicular margin lanceted. Largest specimen examined (SAMC_A073181) 21.2 × 12.3 mm in CD, and 21.5 mm in H. Costae ridged, serrated, highly granular, and equal in width. Intercostal striae narrow, deep, and extend to base. Corallum predominantly pale cream, but freshly collected specimens pale orange with white septa and calicular margin.

Septa hexamerally arranged in five incomplete cycles according to the formula: S_1_ ≥ S_2_ > S_4_ > S_3_ or S_1_ ≥ S_2_ > S_3_ > S_5_ > S_4_. S_1_ highly exsert and each bearing a small palus. S_2_ slightly less exsert, and slightly smaller or equal in size to S_1_. P_2_ similar to P_1_ but rising higher than it in fossa. In half-systems without S_5_, S_3_ smaller and less exsert than S_2_ and bear the widest pali. S_4_ dimorphic in development: those adjacent to S_1_ are wider than those adjacent to S_2._ S_4_ fuses to S_1_ and S_2_ at calicular margin forming triangular apexes. In half-systems with S_5_, S_3_ small and bear a wide palus. S_4_ adjacent to S_2_ slightly wider than S_3_ and also bear a wide palus. S_4_ adjacent to S_1_ lack pali. S_5_ dimorphic in development: those adjacent S_1_ wider but as exsert as ones neighbouring S_2._ All axial edge of septa and pali slightly sinuous, with faces being uniformly covered by pointed and sharp granules. Columella papillose and aligned with GCD, but sometimes difficult to view due to the highly compressed corallum.

###### Distribution.

Regional: Eastern margin of South Africa, off Port St. Johns extending towards Durban; 190–270 m. Elsewhere: Tanzania (Gardiner and Waugh 1938); Japan; South China Sea (Cairns 1994); Philippines; Indonesia (Cairns and Zibrowius 1997); Australia (Cairns 1998); Vanuatu (Cairns 1999a); New Caledonia (Kitahara and Cairns 2021); 123–1110 m.

###### Remarks.

Although two authors have reported *Cyathotrochuspileus* before (Alcock, 1902a), their priority is discounted based on varying nomenclature reasons. *Endopachysaustraliae*Tenison-Woods 1878 account of species was not used in the literature subsequent to its original description. Whilst *Tropidocyathusbougainvillea* Milne-Edwards & Haime, 1857 type material is untraceable and the authors did not illustrate their specimens (Cairns 1989a). Thus, according to article 23.9.1 of the ICZN (1999), *Endopachysaustraliae* is considered to be a nomen oblitum and *C.pileus* to be a nomen protectum. There are two extant species belonging to *Cyathotrochus* (*C.pileus* and *C.nascornatus* (Gardiner and Waugh 1938)), which may be distinguished based on the irregularity of coralla (as a result of asexual reproduction) taken by *C.nascornatus* (Cairns 1989a). Nonetheless, examined specimens represent new records of *Cyathotrochuspileus* from South Africa and, therefore, extend its known distribution from south of Tanzania. Among the Turbinoliidae of the region, *C.pileus* resembles *Tropidocyathuslessonii*, but can be distinguished by the lack of thecal edge crest, ridged costae, and triangular apexes at the calicular margin.

##### Deltocyathoides

Taxon classificationAnimaliaScleractiniaTurbinoliidae

Yabe & Eguchi, 1932

EC980B48-3AA0-5462-A9C4-97ED18352BCB

###### Diagnosis.

Corallum bowl-shaped, with rounded base, and calice circular in cross section; transverse division absent. Costae ridged and serrate; intercostal regions deep, narrow, and not pitted. Higher cycle costae (C_3–4_) originate by bi– or trifurcation. Septa hexamerally arranged in four complete cycles. Sub-lamellar to styliform pali before all but last cycles of septa. Columella papillose.

###### Type species.

*Deltocyathoidesjaponicus* Yabe & Eguchi, 1932a (junior synonym of *Deltocyathusorientalis* Duncan, 1876, which is the type of *Paradeltocyathus* by original designation) (Cairns 1997).

##### Deltocyathoides orientalis

Taxon classificationAnimaliaScleractiniaTurbinoliidae

(Duncan, 1876)

5D4BCC15-7421-5228-BC0C-36FFA4268BCD

Fig. 15C, D

Deltocyathus orientalis Duncan, 1876: 431, pl. 38, figs 4–7.
Deltocyathus lens Alcock, 1902a: 99. –Alcock 1902c: 19–20, pl. 2, figs 16, 16A. –Zou 1988: 77–78, pl. 5, figs 6, 6A.
Deltocyathoides japonicus Yabe & Eguchi, 1932a: 389, fig. 3.
Deltocyathus minutus Gardiner & Waugh, 1938: 1980, fig. 5.
Stephanophyllia fungulus . –Boshoff 1981: 24.
Peponocyathus orientalis . –Wells 1984: 214. –Veron 1986: 608.
Peponocyathus australiensis . –Cairns 1989a: 29, 30–32, pl. 14, figs D–J, pl. 15, figs A–D. –Cairns and Parker 1992: 39–40, pl. 13, figs C, D. –Cairns and Keller 1993, 259–261. –Cairns 1994: 64–65, pl. 28, figs C–F, pl. 41, fig. I.
Deltocyathoides orientalis . –Cairns and Zibrowius 1997: 144–145. –Cairns 1997: 17, pl. 1, fig. H, pl. 7, fig. F. –Cairns 1998: 392. –Cairns 1999: 111. –Cairns et al. 1999: 40. –Cairns 2004a: 292.

###### Type locality.

South-eastern Honshu, Japan (34°12'00"N, 136°20'00"E); 95 m (Cairns 1989a).

###### Type material.

The holotype is presumed to be lost (Zibrowius 1980).

###### Material examined.

ORI_BVIa2 (1 specimen): Locality data unknown, 80 m. SAMC_A073139 (1 specimen): Eastern margin, 35 km from Cape Vidal/32 km off St Lucia Estuary, 27°49'41.87"S, 32°38'12.11"E; 54 m. **USNM 91711 (2 specimens)**: Eastern margin, 26 km from Port St. Johns/off Bulolo Estuary, 29°34'47.99"S, 31°41'59.99"E; 138 m.

###### Description.

Corallum bowl-shaped with a pointed base. Calice circular (GCD:LCD = 1.0–1.2), with a slightly serrated calicular margin. Largest specimen examined (SAMC_A073139) 6.7 mm in CD, and 3.7 mm in H. Costae ridged, granular, with only C_1_ extending from calicular margin to base epicentre. C_3_ fuses to their adjacent C_4_ (~ ^1^/_2_ or ^1^/_3_ from base epicentre), and closer to base the fused costa joins C_2_ to form a single costa that reaches base epicentre. Intercostal striae wide and deep near calicular margin, but progressively getting narrower and shallower near base epicentre. Corallum white.

Septa hexamerally arranged in four cycles according to the formula: S_1_ > S_2_ ≥ S_4_ > S_3_ (48 septa). S_1_ independent, most exsert, and separated from columella by a small palus, which is difficult to distinguish from columellar elements. S_2_ less exsert and ¾ width of S_1_, each bearing a thick palus that joins neighbouring P_3–4_ closer to columella. S_3_ ~ ½ the width of S_2_, less exsert, and bearing a thin palus (P_3_). S_4_ dimorphic in development: those adjacent to S_1_ are wider than S_3,_ but S_4_ adjacent to S_2_ are equal in width to S_3._ Small spines perpendicular to septal and palar margins give them a coarse appearance. Fossa absent. Columella papillose.

###### Distribution.

Regional: Eastern margin of South Africa; extending off Shaka’s Rock towards Cape Vidal; 54–138 m. Elsewhere: Japan (Yabe and Eguchi 1932a); Philippines; Indonesia (Cairns and Zibrowius 1997); Wallis and Futuna (Cairns 1999a); Australia (Cairns and Parker 1992; Cairns 2004); New Zealand (Cairns 1994); Tanzania; Red Sea (Gardiner and Waugh 1938; Cairns and Keller 1993); Florida; and Brazil (Cairns 1979); 44–635 m.

###### Remarks.

This widespread turbinoliid was first reported in the region by Boshoff (1981), who identified it as *Stephanophylliafungulus*. More than one decade later Cairns and Keller (1993) noted the occurrence of *Deltocyathoidesorientalis* in Natal, but as *Peponocyathusaustraliensis* (Cairns and Keller 1993). Subsequent authors (Cairns and Zibrowius 1997; Cairns 1999a, 2004a), then agreed with Cairns (1995) that the genus *Deltocyathoides* should be applied to previously identified *Peponocyathus* that do not undergo transverse division. *Deltocyathoidesorientalis* differs from other turbinoliids in the region in its bowl-shaped corallum, independent S_1_, fused P_2–4_ (which gives septa a V-shaped appearance), septa and pali with spines perpendicular to their margins, and costal morphology.

##### Deltocyathoides sentus

Taxon classificationAnimaliaScleractiniaTurbinoliidae

Kitahara & Cairns, 2021

58917694-6B55-5F83-B9B4-83659D9DAA3F

Fig. 15E, F

Deltocyathus lens . –Gardiner and Waugh 1938: 198.
Deltocyathus italicus . –Zibrowius 1974a: 756–757.
Deltocyathus sp. A. –Cairns and Keller 1993: 245–246, fig. 5G, H.
Deltocyathus sentus Kitahara & Cairns, 2021: 101–102, figs 40, 41G–I.

###### Material examined.

USNM 91551 (1 specimen): Eastern margin, 33 km from SHAKA’S ROCK/34 km off Tongati Estuary, 29°44'17.99"S, 31°27'36.00"E; 300 m.

###### Description.

Corallum unattached, shaped as a bowl with a pointed epicentre base. Calice circular, with serrate calicular margin. Specimen examined 10.0 × 9.8 mm in CD, and 6.3 mm in H. Costae granular, rounded, wider near calicular margin, and progressively smaller in width toward base. Only C_1–2_ extend towards pointed base. Intercostal spaces deep, also being wider near calicular margin, and progressively getting narrower towards base. Corallum white.

Septa hexamerally arranged in four cycles according to the formula: S_1_ > S_2_ > S_4_ > S_3_ (total of 48 septa). S_1_ most exsert, septa independent, bearing the largest and highest pali, which are separated from septa by a deep notch. Higher cycle septa (S_2–4_) becoming progressively less exsert. S_2_ slightly smaller than S_1_, also with a deep notch before a smaller palus. S_3_ ¾ the width of S_2_, with small pali. S_4_ ¼ wider than S_3_, with a pali slightly smaller than P_2_. P_4_ extend towards columella where it forms delta with adjacent P_2_. Sharp granules present on septal and palar faces. Moderately deep fossa, containing a papillose columella composed of a group of intertwined rods.

###### Distribution.

Regional: Eastern margin of South Africa, off Shaka’s Rock; 300 m (Cairns and Keller 1993). Elsewhere: Zanzibar (Gardiner and Waugh 1938); Madagascar (Zibrowius 1974a); New Caledonia (Kitahara and Cairns 2021); 217–1400 m.

###### Remarks.

This taxon is based on the conical *Deltocyathus* species reported by Cairns and Keller (1993), who also suggested that it may be similar to the species mentioned by Zibrowius (1974a) from Madagascar. Cairns and Keller (1993) additionally emphasiszed the differences in the rounded and granular costae, relatively small P_3_, and moderately deep fossa; features with are in agreement with *Deltocyathoidessentus* Kitahara & Cairns, 2021 recently reported from New Caledonia. In addition; *D.sentus* can be distinguished from the only other congener (*D.orientalis*) by having a pointed epicentre, palar faces bearing lateral ridges, S_3_ being less wider that S_4_. The specimen reported herein therefore confirms that *D.sentus* can be found in shallow waters than previously reported (Kitahara and Cairns 2021).

##### Sphenotrochus

Taxon classificationAnimaliaScleractiniaTurbinoliidae

Milne-Edwards & Haime, 1848

5A50D434-5BA5-5CA2-9CA4-BC211B8A3E33

###### Diagnosis.

Corallum cuneiform with a rounded base; transverse division lacking. Theca imperforate; costae smooth, corresponding to septa. Costae sometimes degenerate into discontinuous fragments on basal or all of thecal face. Three or four cycles of septa. Pali absent; columella lamellar.

###### Type species.

*Turbinoliacrispa* Lamarck, 1816, by subsequent designation (Milne-Edwards and Haime 1850b).

##### Sphenotrochus (Eusthenotrochus)

Taxon classificationAnimaliaScleractiniaTurbinoliidae

Wells, 1935

5CF1A13C-05C0-5EEB-B013-35A4CB598822

###### Diagnosis.

*Sphenotrochus* in which the costae are each composed of two or more irregular rows of short narrow ridges.

##### Sphenotrochus (Eusthenotrochus) gilchristi

Taxon classificationAnimaliaScleractiniaTurbinoliidae

Gardiner, 1904

1521F5C6-4707-594B-9796-320A21271747

Fig. 15G, H

Sphenotrochus gilchristi Gardiner, 1904: 98–99, pl. 1, figs A–G. –Boshoff 1981: 39. –Zibrowius and Gili 1990: 44.
Eusthenotrochus moseri Wells, 1935: 530.
Sphenotrochus (Eusthenotrochus) moseri . –Wells 1935: 530–532, pl. 18, figs 5, 6.
Sphenotrochus dentosus Boshoff 1981: 39.
Sphenotrochus sp. (*incertae sedis*). –Boshoff 1981: 39.
Sphenotrochus (Eusthenotrochus) gilchristi . –Cairns and Keller 1993: 259, fig. 7A, B.

###### Type locality.

Near Kowie, South Africa (33°45'20.0"S 26°44'20.0"E); 79–81m (Gardiner 1904).

###### Type material.

Two syntypes are deposited at the BMNH.

###### Material examined.

ORI_DIIIi2 (2 specimens), ORI**_**DIIIi3 (3 specimens), SAMC_A073226 (1 specimen), SAMC_A073075 (1 specimen): Locality data unknown. SAMC_A073232 (1 specimen): Southern margin, 21 km from Cape Point/14 km off Sand Estuary, 34°10'59.99"S, 18°34'59.99"E; 42 m. SAMC_A073236 (4 specimens): Southern margin, 22 km from Cape Point/4 km off Elsies Estuary, 34°09'59.99"S, 18°27'29.99"E; 24 m. SAMC_A090086 (14 specimens): Southern margin, 22 km from Cape Point/4 km off Elsies Estuary, 34°09'59.99"S, 18°27'29.99"E; 24 m. SAMC_A090087 (3 specimens): Southern margin, 18 km from Cape Point/16 km off Buffels Wes Estuary, 34°13'00.00"S, 18°34'59.99"E; 44 m. SAMC_A090088 (6 specimens): Southern margin, 80 km from Gouritsmond/91 km off Blinde Estuary, 34°59'00.00"S, 22°18'00.00"E; 106 m. SAM_H1376 (1 specimen): Southern margin, 28 km from Port Alfred/3 km off Old Womans’ Estuary, 33°30'00.00"S, 27°08'59.99"E; 183 m. SAM_H1405 (1 specimen): Western margin, 51 km from Cape Point/48 km off Krom Estuary, 34°28'59.87"S, 17°58'00.11"E; depth unknown. SAM_H1413 (1 specimen): Southern margin, 11 km from Kenton On Sea/11 km off Boesmans Estuary, 33°45'19.99"S, 26°44'19.99"E; 79–80 m. SAM_H1480 (1 specimen): Southern margin, 35 km from Port Alfred/5 km off Mpekweni Estuary, 33°27'59.99"S, 27°12'59.99"E; 97 m. SAM_H3204 (2 specimens): Southern margin, 6 km from Kenton On Sea/5 km off Boknes Estuary, 33°43'07.59"S, 26°37'37.95"E; 90 m. SAM_H3205 (7 specimens): Southern margin, 37 km from Port Elizabeth/32 km off Bakens River Estuary, 34°05'29.99"S, 25°55'14.99"E; 123 m. SAM_H3206 (3 specimens): Southern margin, 7 km from East London/5 km off Buffalo Estuary, 33°02'59.99"S, 27°57'00.00"E; 59 m. SAM_H3207 (2 specimens): Southern margin, 47 km from Kidds Beach/13 km off Bira Estuary, 33°29'03.99"S, 27°22'59.99"E; 80 m. SAM_H3208 (3 specimens): Southern margin, 31 km from Gonubie/off Gqunube Estuary, 33°09'59.99"S, 28°12'00.00"E; 90 m. SAM_H4243 (1 specimen): Eastern margin, 16 km from Scottburgh/12 km off Mkomazi Estuary, 30°15'00.00"S, 30°54'18.00"E; 100 m. **SAM_H4590 (1 specimen)**: Southern margin, 16 km from Cape Point/12 km off Elsies Estuary, 34°13'08.99"S, 18°31'59.99"E; 37 m.

###### Imagery data.

BMNH 1970.1.26.7 (2 syntypes imaged): Eastern margin, north of Durban; 79–81m; DTE (sub-genus type): Southern margin, 113 km from Gouritsmond/125 km off Blinde Estuary, 35°15'59.99"S, 22°26'06.99"E; 155 m.

###### Description.

Corallum cuneiform, appearing swollen around theca in lateral view. Base rounded giving a V-shaped appearance in side view. Calice elliptical (GCD:LCD = 1.4–2.8), with serrated calicular margin. Largest specimen examined (SAM_H4243) 8.6 × 3.4 mm in CD, and 10.5 mm in H. Costae smooth, well defined, each composed of two or more discontinuous ridges per septa. S_3_ associated costae have three or four longer continuous ridges, which separate 5.0 mm above base. Intercostal striae vary with costae size and arrangement, appearing deep and narrow. Corallum white and sometimes orange.

Septa hexamerally arranged in three cycles according to the formula: S_1–2_ > S_3_ (24 septa). S_1–2_ have straight axial margins. S_3_ dimorphic in size: sometimes ^2^/_3_ the size of S_1–2_ and bearing slightly sinuous axial margin or rudimentary. All septa thick and equally exsert. Septal thickening deposits connect septa to a solid lamellar columella deeper in fossa.

###### Distribution.

Regional: Western to eastern margin of South Africa, from off Cape Point extending towards Scottburgh; 24–155 m. Elsewhere: Only known from South Africa; 24–165 m.

###### Remarks.

Sphenotrochus (E.) gilchristi was first reported in the region by Gardiner (1904), and was subsequently placed in a subgenus by Wells (1935) who reported it as Sphenotrochus (E.) moseri. Thereafter, Cairns and Keller (1993) reported the species in the region and extended the known regional distribution further west.

##### Sphenotrochus (Sphenotrochus)

Taxon classificationAnimaliaScleractiniaTurbinoliidae

Milne-Edwards & Haime, 1848

CBE2203A-7036-510C-8A3D-85F4736A3C1F

###### Diagnosis.

*Sphenotrochus* in which each costae composed of a single, elongated ridge and are continuous from calice to base, or, at least, for upper part of thecal face.

##### Sphenotrochus (Sphenotrochus) aurantiacus

Taxon classificationAnimaliaScleractiniaTurbinoliidae

von Marenzeller, 1904

4FA888D5-EA77-5DE7-A59A-597D58C214EF

Fig. 15I, J

Sphenotrochus aurantiacus von Marenzeller, 1904a: 280–281, pl. 18, fig. 15. –Wells 1935: 531. –Boshoff 1981: 38–39. –Cairns 1989b: 38. –Cairns 1997: 25.
Sphenotrochus (Sphenotrochus) aurantiacus . –Cairns and Keller 1993: 254–255, fig. 7D, E, G, H.

###### Type locality.

Agulhas Bank, South Africa (SS ‘Valdivia’ stn. 104: 35°16'00"S 22°26'00"E); 115 m (von Marenzeller 1904a).

###### Type material.

Two syntypes are deposited at the ZMB (Cairns and Keller 1993).

###### Material examined.

DEFF_SVMEC–INV346: Southern margin, 96 km from PLETT/95 km off Storms Estuary, 34°52'01.38"S, 23°46'17.40"E; 213 m. SAMC_A072997 (1 specimen): Locality data unknown. SAMC_A073073 (1 specimen): Eastern margin, 29 km from Richards Bay/20 km off Nhlabane Estuary, 28°44'23.99"S, 32°23'12.11"E; 320–340 m. SAMC_A073145 (2 specimens): Eastern margin, 5 km from Cape Vidal/16 km off St Lucia Estuary, 28°08'24.00"S, 32°36'24.11"E; 165 m. SAMC_A073160 (1 specimen): Eastern margin, 27 km from Durban/12 km off Mbokodweni Estuary, 30°04'59.88"S, 31°00'24.11"E; 100 m. SAMC_A073162 (4 specimens): Eastern margin, 19 km from Coffee Bay/18 km off Mdumbi Estuary, 32°02'53.87"S, 29°19'41.87"E; 250–280 m. SAMC_A073168 (1 specimen): Southern margin, 37 km from Mazeppa Bay/18 km off Mendu Estuary, 32°22'48.00"S, 29°00'47.88"E; 450–500 m. SAMC_A073172 (1 specimen): Eastern margin, 36 km from Coffee Bay/20 km off Ntlonyane Estuary, 32°18'11.88"S, 29°06'11.88"E; 550 m. SAMC_A073176 (1 specimen): Eastern margin, 35 km from Durban/26 km off Mbokodweni Estuary, 30°07'59.99"S, 31°03'05.99"E; 150 m. SAMC_A073237 (1 specimen): Southern margin, False Bay; depth unknown. SAMC_A087450 (1 specimen): Locality data unknown. SAMC_A090082 (3 specimens): Locality data unknown; 106 m. SAM_H817 (1 specimen): Southern margin, 2 km from Mosselbaai/11 km off Hartenbos Estuary, 34°11'10.12"S, 22°09'40.59"E; 229 m. SAM_H1372 (1 specimen): Eastern margin, 27 km from Mtunzini/25 km off Matigulu Estuary, 29°10'36.00"S, 31°51'00.00"E; 115 m. SAM_H1385 (1 specimen): Eastern margin, 2 km from Durban/8 km off Umgeni Estuary, 29°51'59.99"S, 31°00'00.00"E; 101 m. SAM_H1401 (1 specimen): Southern margin, 13 km from East London/10 km off Ngqenga Estuary, 33°06'44.99"S, 27°55'44.99"E; 79 m. SAM_H1404 (2 specimens): Southern margin, 2 km from Mosselbaai/11 km off Hartenbos Estuary, 34°11'10.12"S, 22°09'40.59"E; 165–183 m. SAM_H1409 (3 specimens): Southern margin, 35 km from Port Alfred/5 km off Mpekweni Estuary, 33°27'59.99"S, 27°12'59.99"E; 97 m. SAM_H1410 (1 specimen): Southern margin, 14 km from Cape Padrone/26 km off Boknes Estuary, 33°45'59.99"S, 26°18'59.99"E; 90 m. SAM_H1411 (9 specimens): Southern margin, 7 km from East London/5 km off Buffalo Estuary, 33°02'59.99"S, 27°57'00.00"E; 59 m. SAM_H1416 (1 specimen): Southern margin, 241 km from Agulhas/247 km off De Mond-Heuningnes Estuary, 36°39'59.99"S, 21°25'59.99"E; 200 m. SAM_H1422 (1 specimen): Southern margin, 6 km from Kidds Beach/5 km off Ncera Estuary, 33°11'59.99"S, 27°40'59.99"E; 79 m. SAM_H1424 (1 specimen): Southern margin, off East London; 95 m; SAM_H3197 (1 specimen): Southern margin, 846 km from Port St. Johns/842 km off Mkweni Estuary, 36°39'59.99" S, 21°25'59.99"E; 366 m. SAM_H3202 (2 specimens): Southern margin, 18 km from Gonubie/off Gqunube Estuary, 29°38'59.99"S, 31°07'59.99"E; 90 m. SAM_H3203 (in part: 2 specimens): Southern margin, 48 km from Kidds Beach/14 km off Bira Estuary, 33°29'24.00"S, 27°22'59.99"E; 80 m. **SAM_H4589 (1 specimen)**: Eastern margin, 46 km from Port Dunford/45 km off Nyoni Estuary, 29°19'00.00"S, 32°00'00.00"E; 366 m.

###### Imagery data.

FAL700 (1 specimen): Southern margin, 24 km from Cape Point/20 km off Sand Estuary, 34°11'06.00"S, 18°38'59.99"E; 44 m.

###### Description.

Corallum cuneiform, appearing swollen. Base rounded or irregular in shape depending on the substrate. Calice elliptical (GCD:LCD = 1.79–3.7), with calicular margin slightly serrated. Largest specimen examined (SAM_H1372) 7.7 × 2.8 mm in CD, and 13.8 mm in H. Costae smooth, well defined, each composed of a single longitudinal ridge near calicular margin, which overlaps with adjacent costae towards base. Costae sometimes discontinuous at base. Costae arrangement and size correspond to septa. Corallum white and sometimes orange.

Septa hexamerally arranged in four cycles according to the formula: S_1_ > S_2_ > S_3_ > S_4_ (48 septa). S_1–2_ highly exsert and bear slightly sinuous axial margins. S_3_ less exsert, ^1^/_3_ smaller than S_1–2,_ and have slightly dentate axial margins. S_4_ rudimentary. All septal faces bear fine, regularly arranged granules. Columella solid, lamellar, and aligned with S_1_. Columellar lamellae rise above septa.

###### Distribution.

Regional: Western to eastern margin of South Africa, off Cape Vidal and extending towards St Lucia; 59–500 m. Elsewhere: No other geographical records are known.

###### Remarks.

Sphenotrochus (S.) aurantiacus is distinctive from the other two Recent species reported in the region by its: i) solid lamellar columella that rises far above the upper septal margin, ii) in having septa arranged in four cycles (48 septa), and iii) a costa:septa ratio of 1:1.

##### Sphenotrochus (Sphenotrochus) evexicostatus

Taxon classificationAnimaliaScleractiniaTurbinoliidae

Cairns in Cairns & Keller, 1993

FA80A198-FC64-5C0E-B638-E14A713BB3AA

Fig. 15K, L

Sphenotrochus intermedius . –Macnae and Kalk 1958: 123.
Sphenotrochus sp. –Pichon 1974: 176, fig. 5.
Sphenotrochus aurantiacus . –Boshoff 1981: 38–39 (in part).
Sphenotrochus (Sphenotrochus) evexicostatus Cairns in Cairns & Keller, 1993: 255, 258, fig. 9A–H.

###### Type locality.

Off south–eastern Mozambique (RV ‘Anton Bruun’ stn. 372B: 24°48'00"S, 34°59'00"E); 42 m (Cairns and Keller 1993).

###### Type material.

The holotype and five paratypes are deposited at the NMNH, whilst one paratype is deposited at the SAM (Cairns and Keller 1993).

###### Material examined.

ORI_DIIIi1 (3 specimens): Locality data unknown. SAMC_A090080 (3 specimens): Southern margin, 22 km from Cape Point/4 km off Elsies Estuary, 34°09'59.99"S, 18°27'29.99"E; 24 m. SAMC_A090083 (1 specimen): Southern margin, 18 km from Cape Point/16 km off Buffels Wes Estuary, 34°13'00.00"S, 18°35'00.0"E; 44 m. SAMC_A090085 (in part: 1 specimen): Locality data unknown; 43 m. SAM_H806 (8 specimens): Southern margin, 37 km from Port Elizabeth/32 km off Bakens River Estuary, 34°05'29.99"S, 25°55'14.99"E; 20 m. SAM_H3200 (2 specimens): Eastern margin, 19 km from Shaka’s Rock/3 km off Mdloti Estuary, 29°38'59.99"S, 31°07'59.99"E; 49 m. SAM_H3201 (1 specimen): Eastern margin, 19 km from Shaka’s Rock/3 km off Mdloti Estuary, 29°38'59.99"S, 31°07'59.99"E; 71–73 m. SAM_H3202 (10 specimens): Southern margin, 18 km from Gonubie/off Gqunube Estuary, 29°38'59.99"S, 31°07'59.99"E; 90 m. **SAM_H4584 (1 paratype)**:Eastern margin, 31 km south of Ponta Do Ouro/32 km off Kosi-Kumpungwini (Sifungwe) Estuary, 24°48'00"S, 34°59.00"E; 42 m. **USNM 77188 (12 paratypes)**: Eastern margin, 24 km from Shaka’s Rock/5 km off Mdlotane Estuary, 29°18'59.99"S, 31°21'00.00"E; 38 m.

###### Description.

Corallum cuneiform. Thecal faces diverge at 12°, producing a triangular profile in lateral view. Base rounded. Calice elliptical (GCD:LCD = 2.4–3.1), with serrated calicular margin. Largest specimen examined (SAM_H806) 6.7 × 2.3 mm in CD, and 8.8 mm in H, showing an episode of rejuvenescence. Costae rounded, equal in width, and do not overlap with adjacent costae. Costae slanting inwards towards base, being continuous until ~ 1.5 mm above base where they form discontinuous ridges. All costae smooth, except C_1_ and adjacent C_4,_ which are slightly granulated. Pairs of C_4_ occur in each half-system adjacent to S_1_ (32 costae), but S_4_ do not correspond to C_4._ Intercostal striae wider than costae. Corallum white but sometimes light-yellow to brown.

Septa hexamerally arranged in three cycles according to the formula: S_1–2_ > S_3_ (24 septa). S_1–2_ moderately exsert, equal in width, and join columella deep in fossa with slightly sinuous axial margin. S_3_ less exsert, ^1^/_3_ the width of S_1–2_, and bear slightly dentated axial margin. Columella solid and lamellar. Lamella rises almost to the height of septal upper margins. Columellar elements and septal faces bear low-profile granules.

###### Distribution.

Regional: Southern to eastern margin of South Africa, from off Cape Point extending towards Durban; 24–90 m. Elsewhere: Mozambique (Cairns and Keller 1993); Madagascar (?) Pichon 1974); 12–73 m.

###### Remarks.

Examined specimens of Sphenotrochus (S.) evexicostatus add no taxonomic knowledge to the existing diagnosis. The species is well described by Cairns and Keller (1993), who noted that it may co-exist with *S.imbricaticostatus*, which has similar septa, costae, and columella. However, *S.evexicostatus* can be distinguished in having: round, ridged, non-imbricate, equidistant costae; wide intercostal striae that are > 35% width of costae; and S_3_ ~ ^1^/_3_ the width of S_1–2_. The ornamentation of costae of *S.evexicostatus*, which is continuous from calice and become disconnected ~ 1.5 mm from base, is also a distinctive feature.

##### Sphenotrochus (Sphenotrochus) imbricaticostatus

Taxon classificationAnimaliaScleractiniaTurbinoliidae

Cairns in Cairns & Keller, 1993

E0227A41-4775-56ED-A3DC-120A7D4764F5

Fig. 15M, N

Sphenotrochus aurantiacus . –Boshoff 1981: 38–39 (in part).
Sphenotrochus (Sphenotrochus) imbricaticostatus Cairns in Cairns & Keller, 1993: 258–259, fig. 9A–H.

###### Type locality.

Off Kosi Bay, South Africa (RV ‘Meiring Naude’ stn. ZB27: 27°03'00"S 32°53'00"E); 44 m (Cairns and Keller 1993).

###### Type material.

The holotype and eight paratypes are deposited at the NMNH, whilst one paratype is deposited at the SAM (Cairns and Keller 1993).

###### Material examined.

SAMC_A090084 (1 specimen): Southern margin, 22 km from Cape Point/4 km off Elsies Estuary, 34°09'59.99"S, 18°27'29.99"E; 24 m. SAMC_A090085 (in part: 12 specimen): Locality data unknown; 43 m. SAM_H3203 (in part: 2 specimens): Southern margin, 48 km from Kidds Beach/14 km off Bira Estuary, 33°29'24.00"S, 27°22'59.99"E; 80 m. **SAM_H4586 (2 paratypes)**: Eastern margin, 31 km south of Ponta Do Ouro/32 km off Kosi-Kumpungwini (Sifungwe) Estuary, 24°48'00"S, 34°59.00"E; 42 m. **USNM–91715 (holotype)**: Eastern margin, 23 km south of Ponta Do Ouro/15 km off Kosi-Kumpungwini (Sifungwe) Estuary, 27°03'32.40"S, 32°52'59.99"E; 44 m. **USNM–91717 (2 paratypes)**: Eastern margin, 38 km from Shaka’s Rock/16 km off Zinkwasi Estuary, 29°21'00.00"S, 31°34'59.99"E; 57 m.

###### Description.

Corallum cuneiform, with highly compressed faces in lower corallum. Upper corallum highly flared, producing a rectangular-like side view. Base slightly rounded. Calice elliptical (GCD:LCD = 1.4–2.8). Largest specimen examined (USNM–91717) 3.7 × 2.5 in CD, and 6.7 mm in H. Costae smooth, flat, unequally in width, and ridged. In some specimens 12–14 costae overreach base and meet counterparts from opposite side. Pairs of C_4_ occur in each half-system adjacent to two S_1_ (32 costae), but S_4_ does not correspond to C_4_. Intercostal striae narrow, being slightly wider than costae. Corallum light brown.

Septa hexamerally arranged in three cycles according to the formula: S_1–2_ > S_3_ (24 septa). S_1–2_ moderately exsert, equal in width, and extending ^4^/_5_ distance to columella with thickened and vertical axial margins. S_3_ equally exsert, but ¾ the width of S_1–2_. S_3_ axial margin narrow, straight to sinuous. Columella composed of a sharp-edged lamella that rises as high as septa. Columella and all septal faces covered by slender spines, giving them a rough appearance.

###### Distribution.

Regional: Southern and Eastern margin of South Africa, off Cape Point extending towards Kosi-Kumpungwini (Sifungwe) Estuary (23 km south of Ponta Do Ouro: Mozambique); 24–80 m. Elsewhere: south-eastern Mozambique (Cairns and Keller 1993); 37–347 m.

###### Remarks.

Sphenotrochus (S.) imbricaticostatus differs from the two other South African species in the subgenus (*S.evexicostatus* and *S.aurantiacus*) by its compressed rectangular cuneiform corallum and wide, flat, imbricate costae, which alternates in width and imbricate with edges of adjacent costae (Cairns and Keller 1993).

##### Tropidocyathus

Taxon classificationAnimaliaScleractiniaTurbinoliidae

Milne-Edwards & Haime, 1848

B92F40F4-857D-54DF-95C6-A7991737F2B5

###### Diagnosis.

Corallum cuneiform, with rounded base and calice elliptical in cross section. Costae low, flat, and covered with small granules. Thecal edge costae expanded into alate edge crests and also uniformly granulated. Intercostal regions shallow, narrow, and not pitted. Higher cycle costae originate by trifurcation. Septa highly exsert and hexamerally arranged in four complete cycles. Lamellar pali in three crowns before all but last septal cycle. Each pair of P_3_ and single P_2_ in a system forming a chevron arrangement, but not fused. Columella papillose.

###### Type species.

*Flabellumlessoni* Michelin, 1842, by monotypy.

##### Tropidocyathus lessonii

Taxon classificationAnimaliaScleractiniaTurbinoliidae

(Michelin, 1842)

920C351F-7FC1-527E-9905-BC36F149381B

Fig. 15O–P

Flabellum lessonii Michelin, 1842: 119.
Tropidocyathus lessoni . –Milne-Edwards and Haime 1857: 57. –Gardiner and Waugh 1938: 194.–Cairns 1989a: 33–34, pl. 16D–L. –Cairns 1994: 67, pl. 29A, B. –Cairns and Keller 1993: 253, fig. 7C.
Trochocyathus (Tropidocyathus) lessoni . –Alcock 1902a: 17, pl. 2, figs 14, 14A.
Trochocyathus (Tropidocyathus) cf. lessoni . –Yabe and Eguchi 1942b: 124.
Trochocyathus (Tropidocyathus) wellsi Yabe & Eguchi, 1942b: 153, pl. 10, fig. 22A, B.
Tropidocyathus lessonii. –Cairns and Zibrowius 1997: 146–147. –Cairns 1997: 15–16, figs 1E, 4E, 7D. – Cairns 1998: 390–392. –Cairns 1999a: 110, fig. 17C. –Kitahara and Cairns 2021: 147–148, figs 68A–C, I–L, 7.

###### Type locality.

Unknown.

###### Type material.

The holotype is deposited at the MNHNP (Cairns 1989a).

###### Material examined.

SAMC_A073064 (1 specimen): Eastern margin, 5 km from Cape Vidal/16 km off St Lucia Estuary, 28°07'30.00"S, 32°36'24.11"E; 75–80 m. SAMC_A073095 (3 specimens): Eastern margin, 66 km south of Ponta Do Ouro/15 km off Mgobezeleni Estuary, 27°25'59.87"S, 32°44'30.12"E; 55–100 m. SAMC_A073106 (1 specimen): Eastern margin, 66 km from Cape Vidal/7 km off Mgobezeleni Estuary, 27°33'11.88"S, 32°43'00.12"E; 140 m. SAMC_A073131 (4 specimens): Eastern margin, 39 km from Cape Vidal/29 km off Mgobezeleni Estuary, 27°47'23.99"S, 32°38'53.87"E; 65–70 m. SAMC_A073135 (1 specimen): Eastern margin, 19 km from Coffee Bay/18 km off Mdumbi Estuary, 32°02'53.87"S, 29°19'41.87"E; 250–280 m. SAMC_A073149 (1 specimen): Eastern margin, 19 km south of Ponta Do Ouro/12 km off Kosi-Kumpungwini (Sifungwe) Estuary, 27°01'05.87"S, 32°55'12.00"E; 78 m. SAMC_A073191 (1 specimen): Eastern margin, 41 km south of Ponta Do Ouro/26 km off Kosi Bay Estuary, 27°12'53.99"S, 32°49'41.87"E; 66–71m. SAMC_A073209 (1 specimen): Eastern margin, 37 km south of Ponta Do Ouro/23 km off Kosi Bay Estuary, 27°11'05.99"N, 32°50'53.88"E; 100 m. SAMC_A073214 (1 specimen): Eastern margin, 37 km from Cape Vidal/32 km off Mgobezeleni Estuary, 27°48'47.88"S, 32°38'53.87"E; 50 m. SAMC_A073218 (1 specimen): Eastern margin, 42 km south of Ponta Do Ouro/27 km off Kosi Bay Estuary, 27°13'30.00"S, 32°49'30.00"E; 74 m. SAMC_A090079 (1 specimen): Eastern margin, 56 km south of Ponta Do Ouro/25 km off Mgobezeleni Estuary, 27°20'35.87"S, 32°46'41.88"E; 60 m. **SAM_H3101 (2 specimens)**: Eastern margin, 19 km from Shaka’s Rock/3 km off Mdloti Estuary, 29°38'59.99"S, 31°07'59.99"E; 71–73 m. **SAM_H3102 (1 specimen)**: Eastern margin, 6 km from Durban/9 km off Umgeni Estuary, 29°52'59.99"S, 31°03'04.99"E; 86 m. **SAM_H4583 (1 specimen)**: Eastern margin, 41 km south of Ponta Do Ouro/26 km off Kosi Bay Estuary, 27°13'05.99"S, 32°49'30.00"E; 60 m. **SAM_H4588 (2 specimens)**: Eastern margin, 42 km south of Ponta Do Ouro/27 km off Kosi Bay Estuary, 27°13'35.99"S, 32°49'17.99"E; 75 m.

###### Description.

Corallum cuneiform, with thecal edge crests extending laterally. Base rounded. Calice elliptical (GCD:LCD = 1.4–1.6), calicular margin slightly serrated. Largest specimen examined (SAMC_A073064) 16.8 × 10.8 mm in CD, and 16.4 mm in H. Costae broad, flat, variable in size. C_1–3_ wider than C_4_, and bearing 3–4 granules across width. C_4_ bear two granules across a costal width granules. Intercostal striae narrow, shallow, and fainting towards base and thecal crests. Thecal crests also lack costae, which are replaced by uniform granules. Corallum predominantly orange with white calicular margin and septa.

Septa hexamerally arranged in four cycles according to the formula: S_1_ > S_2_ > S_4_ > S_3_ (48 septa). S_1_ highly exsert, and extends to columella deep in fossa with straight axial edges. P1 small and separated from its septum by a small notch. S_2_ equally exsert, slightly less wide than S_1_, bearing a broad and larger palus. S_3_ less exsert than S_1–2_, ¾ the width of S_1–2_, and have a slightly sinuous axial margin. Each S_3_ bears a large palus, which joins P_2_ producing a V-shape appearance. S_4_ less exsert than S_3_, but slightly wider. S_4_ axial edges straight. All palar and septal faces bear sharp spines. Columella papillose, elongated, aligned to principal S_1_, sometimes fused as a lamella in larger specimens. Fossa moderately deep.

###### Distribution.

Regional: Eastern margin of South Africa, off Coffee Bay and extending towards Kosi-Kumpungwini (Sifungwe) Estuary (19 km south of Ponta Do Ouro: Mozambique); 50–280 m. Elsewhere: Tanzania (Gardiner and Waugh 1938); Mozambique; Kenya; north-eastern Somalia (Cairns 1989b); Vanuatu; and Wallis and Futuna Islands (Cairns 1999a); Philippines; Indonesia; China (Yabe and Eguchi 1942b; Cairns and Zibrowius 1997); New Caledonia (Kitahara and Cairns 2021); Australia (Cairns 1998); 50–421 m.

###### Remarks.

*Tropidocyathuslessonii* is distinctive from other South African Turbinoliidae in its thecal crests and colouration. However, it may be mistaken with *Endopachysgrayi*, which belongs to another family distinguished by its porous corallum (Dendrophylliidae). *Tropidocyathuslessonii* differs from its only extant congener, *T.labidus* Cairns & Zibrowius, 1997, in its conspicuous thecal edge crests.

## Conclusions

The present study has added to the knowledge base of the azooxanthellate coral fauna by updating 31 incorrectly identified Boshoff (1981) specimens, thus addressing a research priority identified more than 30 years ago (Zibrowius and Gili 1990). Furthermore, we report 28 new records for South Africa, three new species and one new genus. To further advance this research, molecular characteristics of specimens collected through recent surveys are being investigated for more integrated taxonomy and interrogation of patterns in depth and distribution are needed to support improved understanding of biogeography. Overall, this monograph represents an important contribution in terms of South African marine biodiversity and the diversity and distribution of azooxanthellate corals in general.

## Supplementary materials

Supplementary material 1 Azooxanthellate Scleractinia (Cnidaria_Anthozoa) from South Africa Data type excel table Explanation note Associated data for the above-mentioned paper. https://binary.pensoft.net/file/604119This dataset is made available under the Open Database License (http://opendatacommons.org/licenses/odbl/1.0/). The Open Database License (ODbL) is a license agreement intended to allow users to freely share, modify, and use this Dataset while maintaining this same freedom for others, provided that the original source and author(s) are credited.Zoleka N. Filander, Marcelo V. Kitahara, Stephen D. Cairns, Kerry J. Sink, Amanda T. Lombard

Supplementary material 2 Accompanying maps Data type word document Explanation note **Figure S1.** Map showing distributional records of the South African azooxanthellate coral species with coordinate data. **Figure S2.** Map of South Africa showing coastal cities in text box and the circled numbers represent estuaries (explained in Figures S3–S5). **Figure S3.** Map showing a magnified section of the western margin, where text boxes represent coastal cities and the circled numbers represent estuaries. **Figure S4.** A: Map showing a magnified section of the southern margin, where text boxes represent coastal cities and the circled numbers represent estuaries. B: Map showing the continuation of a magnified section of the southern margin, where text boxes represent coastal cities and the circled numbers represent estuaries. **Figure S5.** Map showing a magnified section of the eastern margin, where yellow text boxes represent coastal cities and the circled numbers represent estuaries. https://binary.pensoft.net/file/604120This dataset is made available under the Open Database License (http://opendatacommons.org/licenses/odbl/1.0/). The Open Database License (ODbL) is a license agreement intended to allow users to freely share, modify, and use this Dataset while maintaining this same freedom for others, provided that the original source and author(s) are credited.Zoleka N. Filander, Marcelo V. Kitahara, Stephen D. Cairns, Kerry J. Sink, Amanda T. Lombard
