# ﻿Corrigendum: Filander ZN, Kitahara MV, Cairns SD, Sink KJ, Lombard AT (2021) Azooxanthellate Scleractinia (Cnidaria, Anthozoa) from South Africa. ZooKeys 1066: 1–198. https://doi.org/10.3897/zookeys.1066.69697

**DOI:** 10.3897/zookeys.1129.95715

**Published:** 2022-11-16

**Authors:** Zoleka N. Filander, Marcelo V. Kitahara, Stephen D. Cairns, Kerry J. Sink, Amanda T. Lombard

**Affiliations:** 1 Biodiversity and Coastal Research, Oceans and Coasts, Department of Forestry, Fisheries, and Environment, Cape Town, South Africa; 2 Zoology Department, Nelson Mandela University, Port Elizabeth, South Africa; 3 Universidade Federal de São Paulo, Departamento de Ciências do Mar, Santos, Brazil; 4 Centro de Biologia Marinha, Universidade de São Paulo, São Sebastião, Brazil; 5 Department of Invertebrate Zoology, Smithsonian Institution, Washington DC, USA; 6 South African National Biodiversity Institute, Cape Town, South Africa; 7 Institute for Coastal and Marine Research, Nelson Mandela University, Port Elizabeth, South Africa

## ﻿

This is a short communication to revise the plates presented in the Filander et al. (2021) monograph on the South African azooxanthellate coral fauna. The authors moreover take this opportunity to retract the addition of *Paracontrochuscapensis* (Gardiner, 1904) as a synonym of *Monohedotrochuscapensis* (Gardiner, 1905). Cairns and Parker (1992) based their new combination of *P.capensis* on *Duncaniacapensis* Gardiner, 1904. In other words, Gardiner’s (1904) species is *P.capensis* and should be considered to occur in South Africa. Below we present the updated species synonymy and the revised figures.

**Figure 3.**
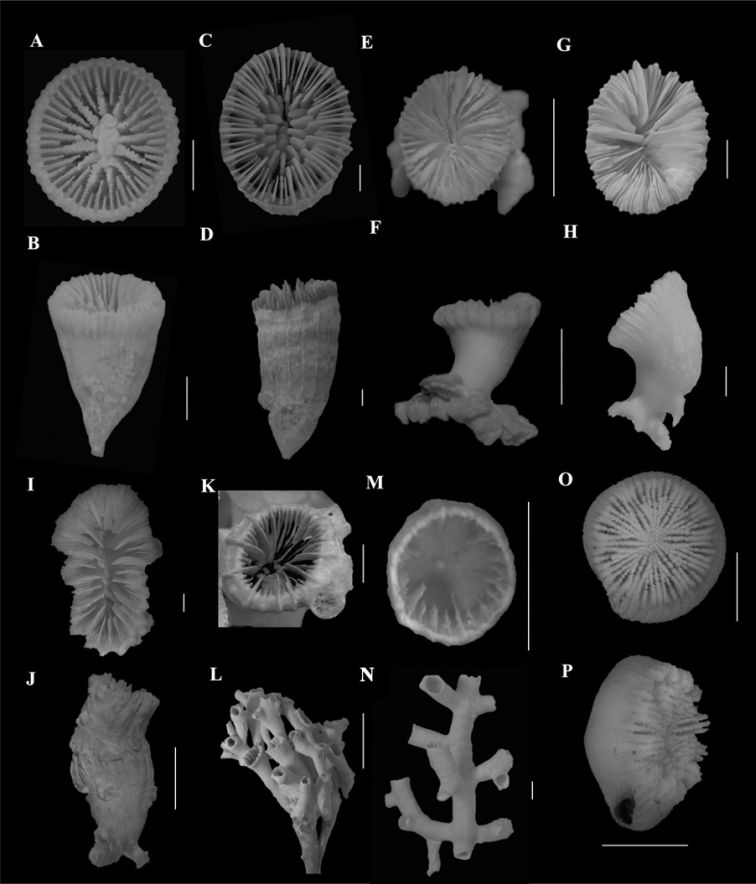
**A, B**Caryophyllia (Caryophyllia) stellula (SAM_H1485, off Agulhas, 200 m) **A** calicular view **B** lateral view **C, D**Caryophyllia (Caryophyllia) valdiviae (SAM_H3108, off Durban, depth unknown) **C** calicular view **D** lateral view **E, F***Crispatotrochuscornu* (UCT_NAD 17 F, off Isipingo, 49 m) **E** calicular view **F** lateral view **G, H, I, J***Desmophyllumdianthus***G, H** (SAMC_A077974, off Paternoster, 440 m) **G** calicular view **H** lateral view **I, J** (BMNH.1939.7.20.218, locality data unknown) **I** calicular view **J** lateral view **K, L***Desmophyllumpertusum* (SAM_H1605, off Melkbosstrand, depth unknown) **K** calicular view **L** lateral view **M, N***Goniocorelladumosa* (SAM_H3190, off Kidds Beach, 760 m) **M** calicular view **N** lateral view **O, P***Heterocyathusaequicostatus* (SAMC_A073186, off Durban, 150 m) **O** calicular view **P** lateral view. Scale bars: 10 mm (**A–I, K–P**); 100 mm (**J**).

**Figure 4.**
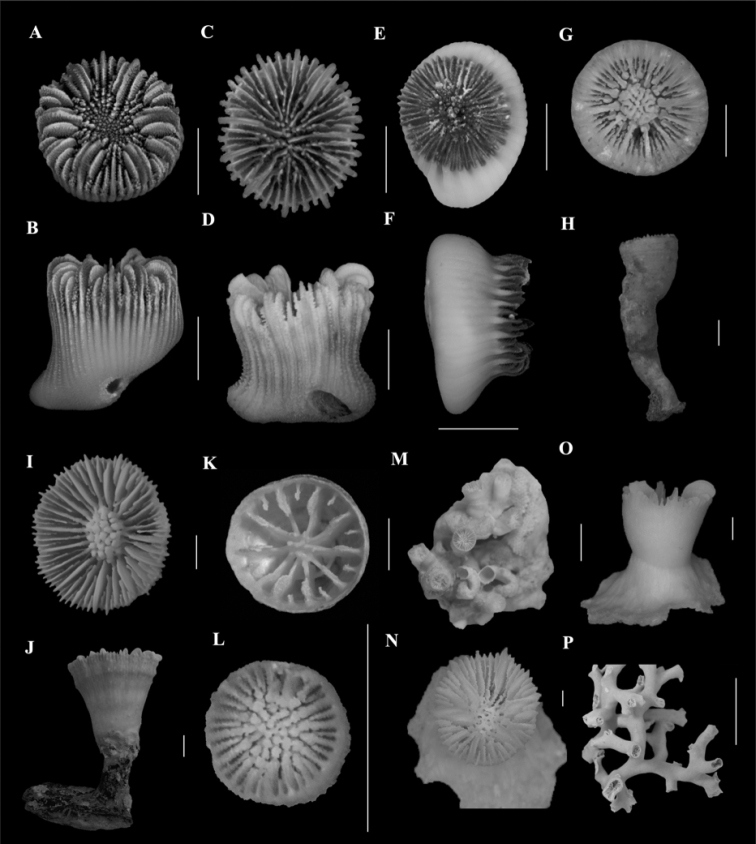
**A, B***Heterocyathusalternatus* (ORI_DIIIe1_1, locality data unknown) **A** calicular view **B** lateral view **C, D***Heterocyathusmonileseptatum* sp. nov. (SAM_H1431, off Durban Harbour, 99 m) **C** calicular view **D** lateral view **E, F***Heterocyathussulcatus* (SAMC_A073123, off Shaka’s Rock, 100–105 m) **E** calicular view **F** lateral view **G, H***Labyrinthocyathusdelicatus* (SAM_H2836, off East London, 146–238 m) **G** calicular view **H** lateral view **I, J, K***Monohedotrochuscapensis* comb. nov. **I, J** (SAMC_A088924, off Kidds Beach, 247–147 m) **I** calicular view **J** lateral view **K** (SAM_H3210, off Scottburgh, 690 m) calicular view **L, M***Polycyathus* sp. (USNM 91677, off Port Dunford, 69 m) **L** calicular view **M** full view **N, O***Rhizosmiliarobusta* (USNM 91689, off Kosi Bay Estuary, 74 m) **N** calicular view **O** lateral view **P***Solenosmiliavariabilis* (SAM_H3158, off Cintsa, 630 m) full view. Scale bars: 10 mm (**A–G, I–O**); 100 mm (**H, P**).

## Revised figures

**﻿Text revision**

***Monohedotrochus* Kitahara & Cairns, 2005**

**Diagnosis.** Corallum solitary, attached, straight, and elongate-conical to trochoid. Base monocyclic. Septotheca costate. Pedicel and base thick. Pali may be present, indistinguishable from columella. Columella papillose.

**Type species.***Monohedotrochuscapitolii* Kitahara & Cairns, 2005, by original designation.

***Monohedotrochuscapensis* (Gardiner, 1904), comb. nov.**

Fig. 4I–K

*Caryophylliacapensis*Gardiner 1904: 113–114, pl. 1, fig. 4A–D. – Boshoff 1981: 36. – Zibrowius and Gill 1990: 44.

*Desmophyllumcristagalli*. – Boshoff 1981: 37.

*Balanophylliacapensis*. – Boshoff 1981: 40 (in part).
