# Supplementary material for: Azooxanthellate Scleractinia (Cnidaria, Anthozoa) from South Africa
Source: Zookeys. 2021 Oct 28;1066:1–198. doi: 10.3897/zookeys.1066.69697 (PMC9633978; doi:10.3897/zookeys.1066.69697)
Supplement: Supplementary material 2 — Accompanying maps [file zookeys-1066-001-s002.docx]

###### Supplementary 2. Accompanying maps


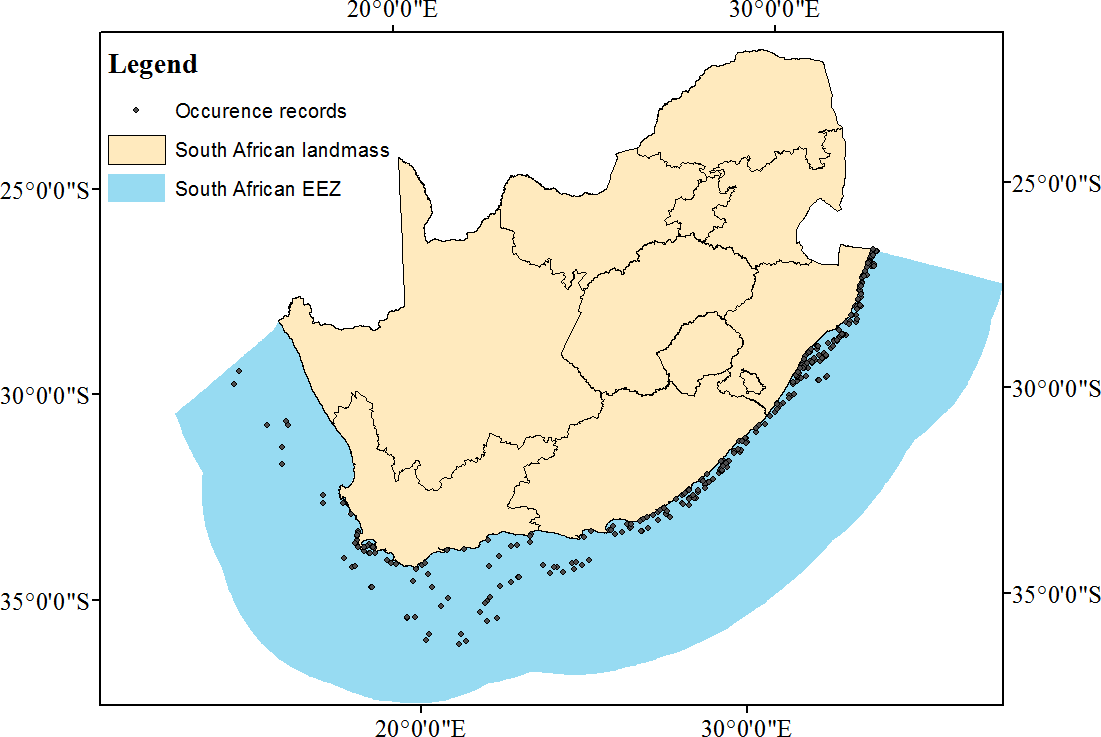


Figure 1: Map showing distributional records of the South African azooxanthellate coral species with coordinate data.


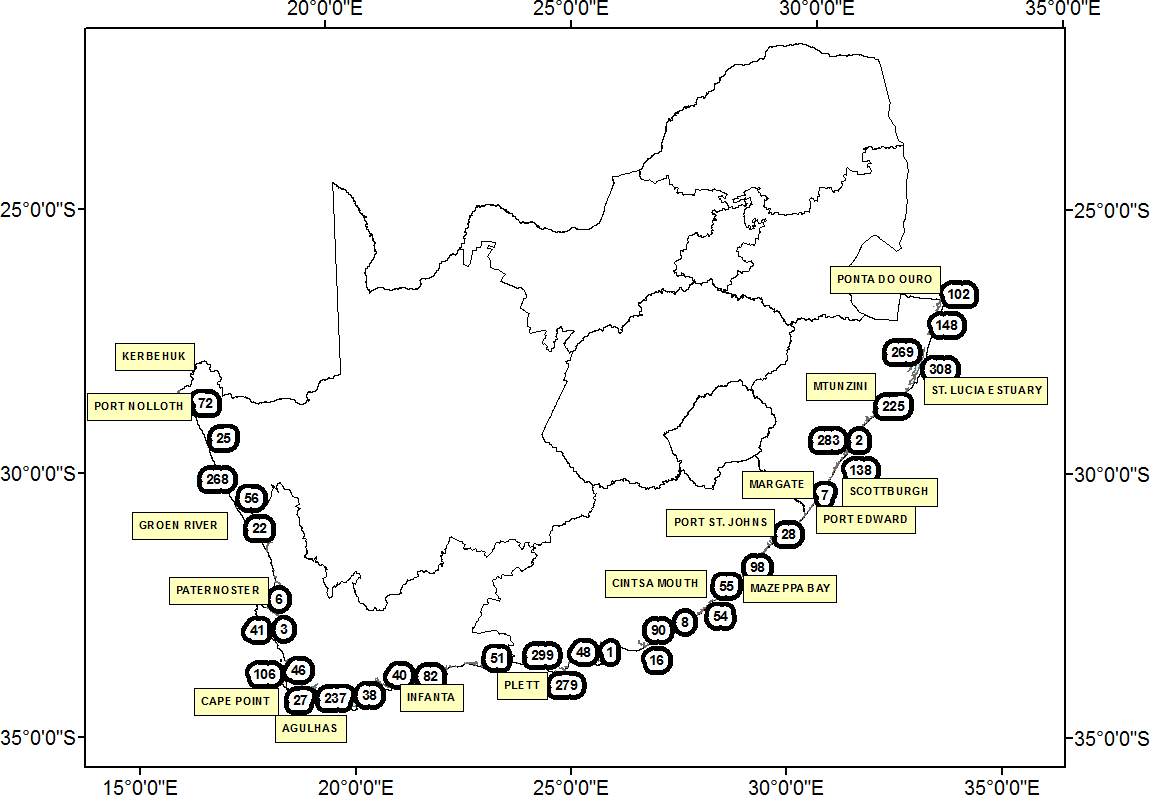


Figure 2: Map of South Africa showing coastal cities in text box and the circled numbers represent estuaries (explained in Figures 3-5).

## Legend


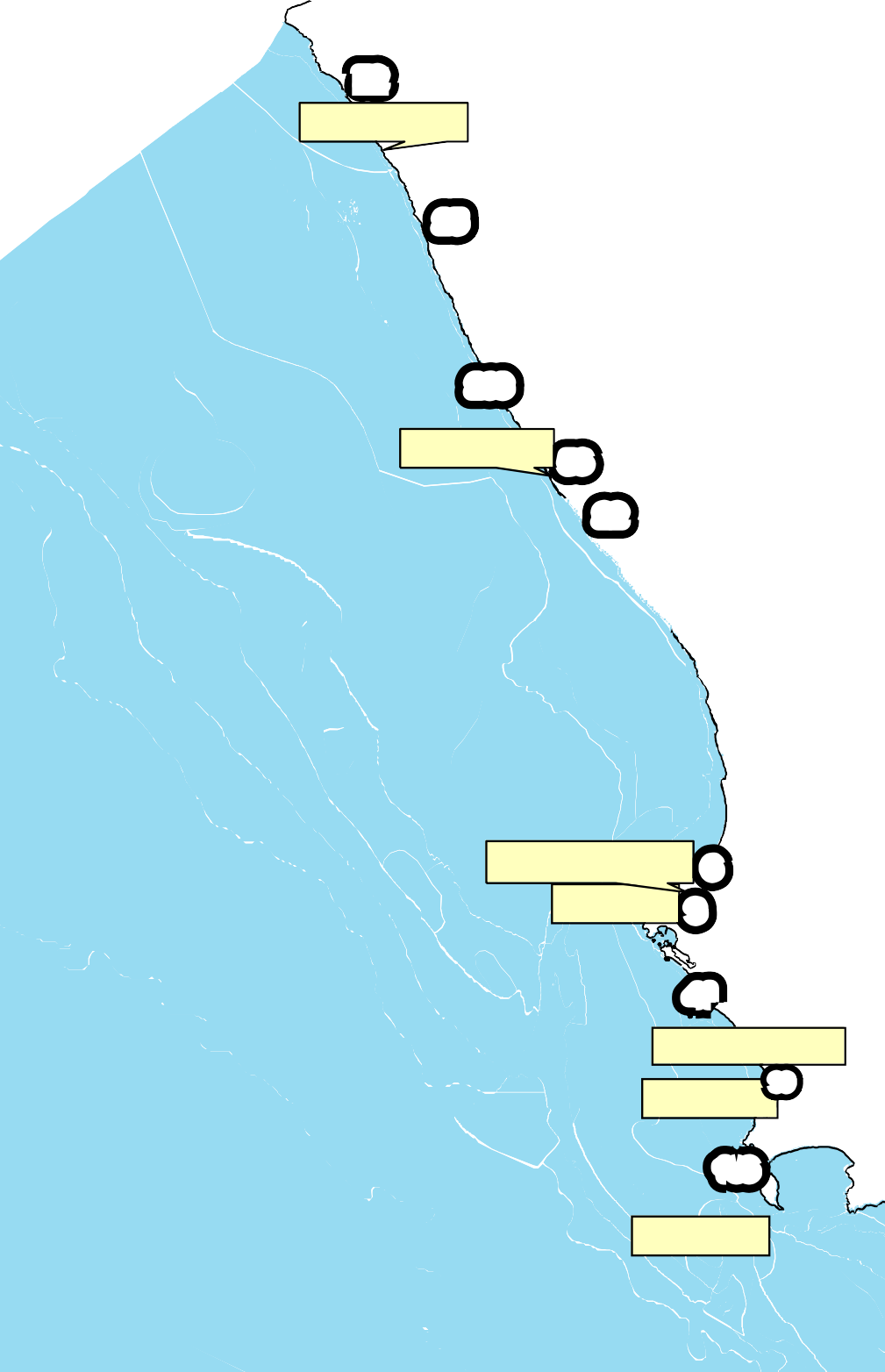


**72**

**PORT NOLLOTH**

3 = Berg River I Floodplain 6 = Berg River V Estuary 22 = Brak

25 = Buffels

39 = Diep

41 = Dwars (North)

**25**

**268**

**PATERNOSTER**

**6**

**SALDANHA 3**

**41**

**MELKBOSSTRAND**

**39**

**CAPE TOWN**

**106**

**KERBEHUK**

**Estuary** 56 = Groen

##### 72 = Holgat

106 = Krom

268 = Spoeg

**Others**

South African landmass South African EEZ


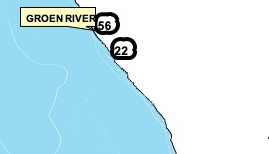


Figure 3: Map showing a magnified section of the western margin, where text boxes represent coastal cities and the circled numbers represent estuaries.

# Legend

## Estuary

### 12 = Blinde

20 = Bot River Lagoon 26 = Buffels Oos

27 = Buffels Wes

38 = De Mond-Heuningnes 40 = Duiwenhoks

46 = Elsies

51 = Goukamma

66 = Hartenbos

82 = Goukou

232 = Piesang

237 = Ratels

247 = Sand

263 = Slang

279 = Tsitsikamma

299 = Storms

**Others**

###### South African landmass South African EEZ

**OUBOSSTRAND**

**247**

**46**

**66**

**12 MOSSELBAAI**

**51**

**KNYSNA**

**PLETT**

**232 299**

**279**

**OESTERBAAI**

**263**

**CAPE ST. FRANCIS**

**27 26**

**PRINGLE 20 GANSBAAI**

**237**

**INFANTA 40**

**ARNISTON**

**38**

**82**

**STILBAAI**

**GOURITSMOND**


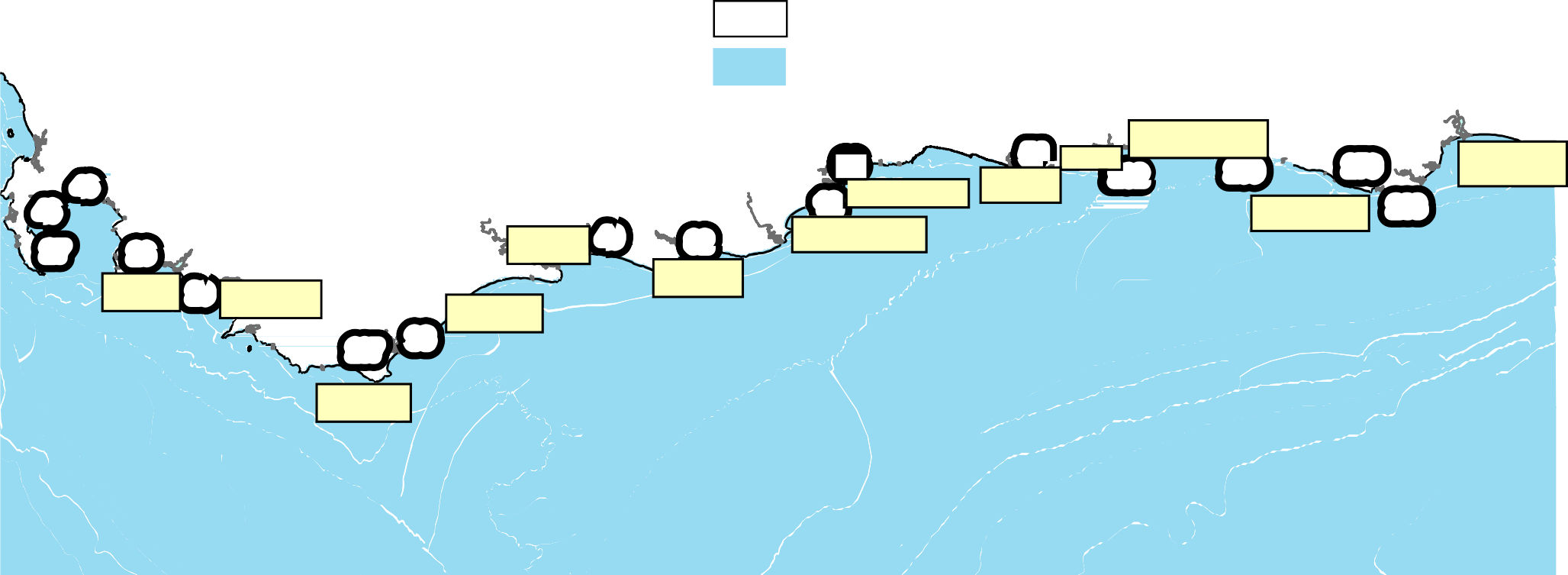


**AGULHAS**

Figure 4A: Map showing a magnified section of the southern margin, where text boxes represent coastal cities and the circled numbers represent estuaries.

#
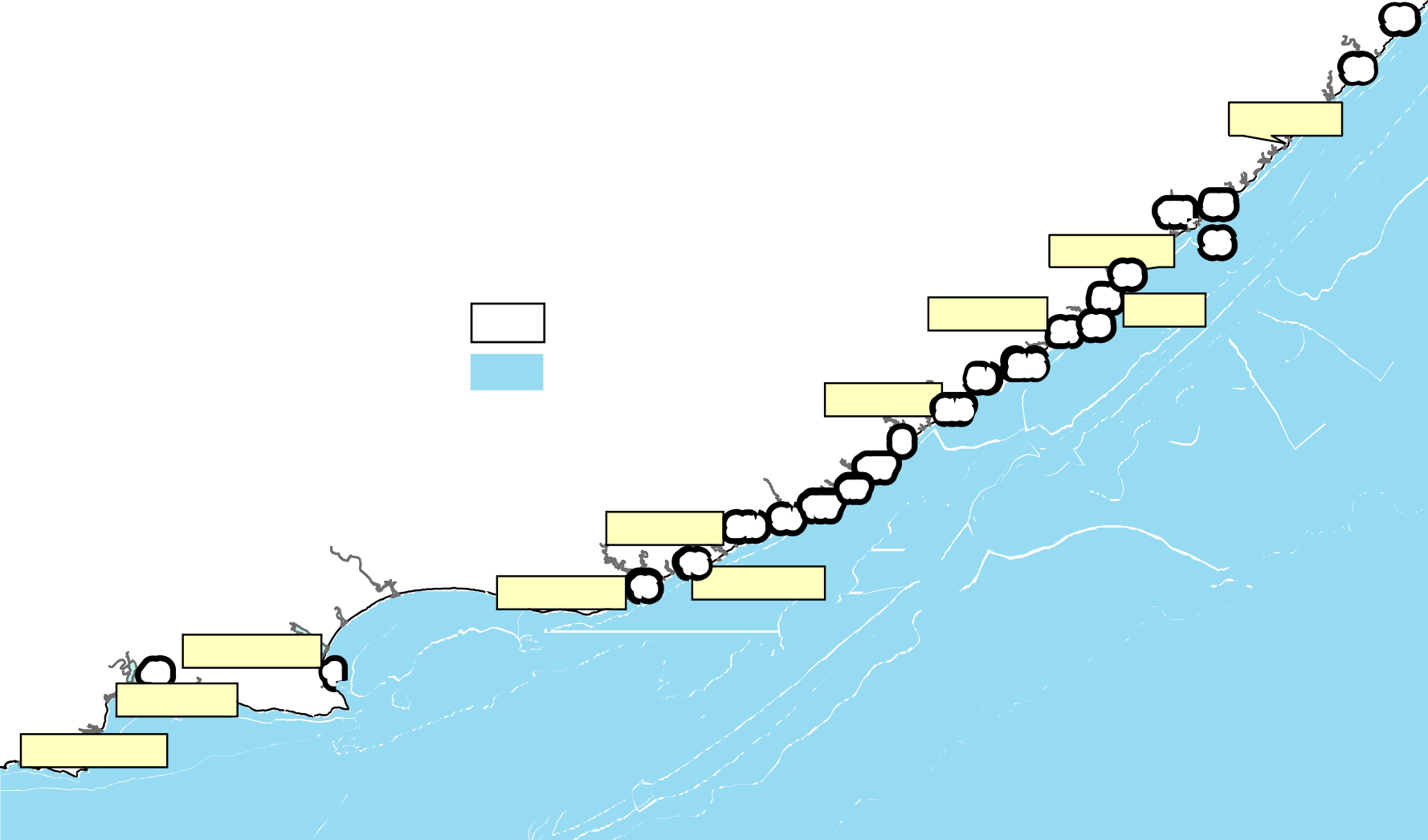
Legend

## Estuary

### 1 = Bakens River 8 = Bira

11 = Blind

14 = Boesmans

16 = Boknes

50 = Gouda

54 = Gqunube 55 = Great Kei

90 = Kleinemond (Oos) 98 = Kobole

206 = Ncera

212 = Ngqenga

226 = Old Womans’s 240 = Riet

**Others**

**171**

**CINTSA MOUTH**

**30**

**98**

**MAZEPPA BAY**

**55**

**36**

**145**

### 24 = Buffalo

30 = Bulura

145 = Mendu

149 = Mgwalana South African landmass

**54**

**EAST LONDON**

**24 11**

**GONUBIE**

### 36 = Cwili

48 = Gamtoos

171 = Morgan

177 = Mpekweni

###### South African EEZ

**KIDDS BEACH**

**8**

**50**

**206**

**212**

**PORT ALFRED**

**1164**

**240**

**90 226**

**149**

**177**

**CAPE PADRONE 16 KENTON ON SEA**

**PORT ELIZABETH**

**48 1**

**JEFFREYS BAY**

**CAPE ST. FRANCIS**

Figure 4B: Map showing the continuation of a magnified section of the southern margin, where text boxes represent coastal cities and the circled numbers represent estuaries

###### .


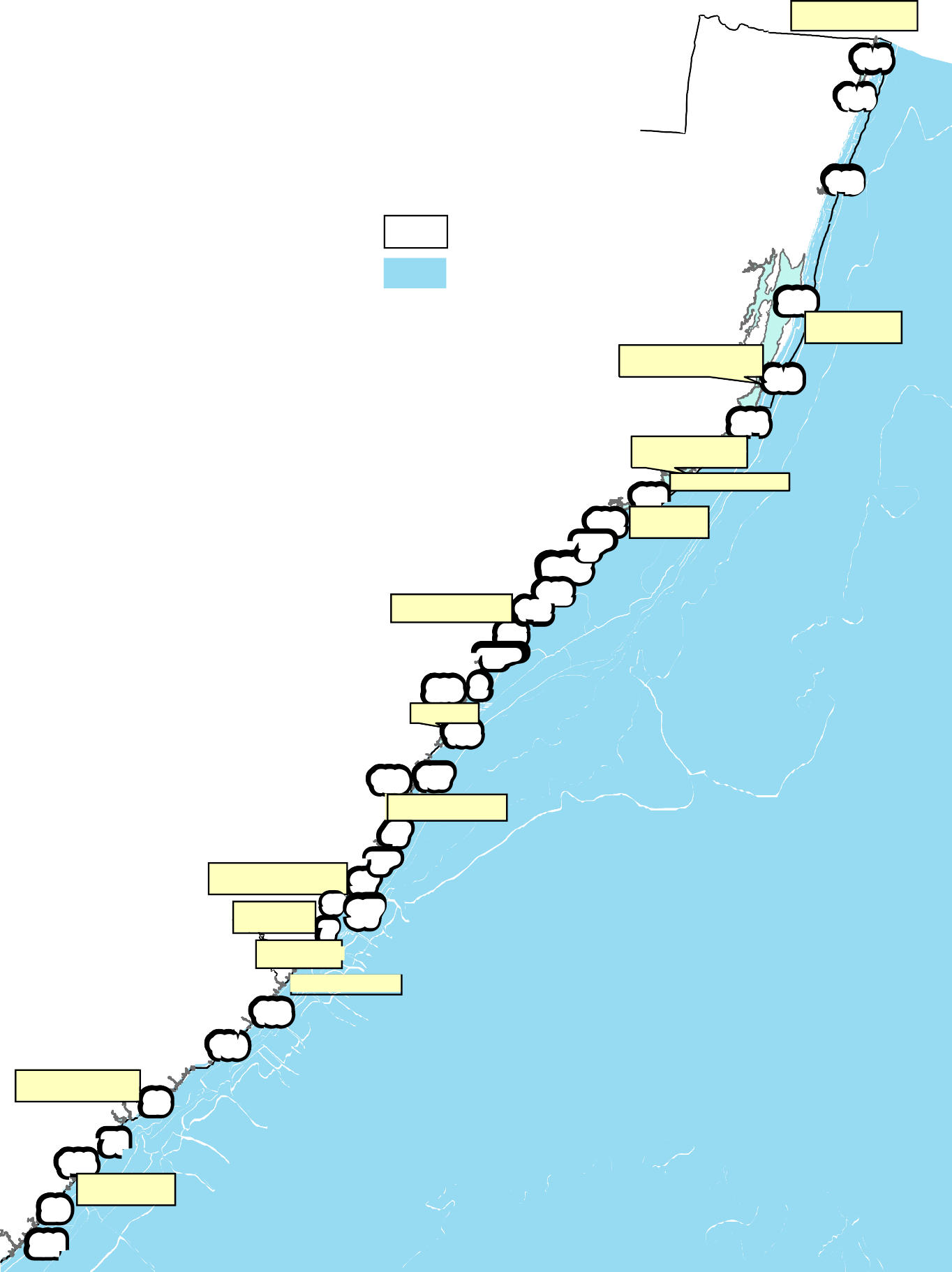
**Legend**

**Estuary**

#### 2 = Beachwood Mangroves 7 = Bilanhlolo

13 = Boboyi

28 = Bulolo

29 = Bulungulu

37 = Damba

47 = Fafa

71 = Hluleka

269 = St Lucia 293 = Zinkwasi

308 = Mfolozi

**Others**

South African landmass South African EEZ

**PONTA DO OURO**

**102**

**104**

**148**

**269**

#### 102 = Kosi - Kumpungwini (Sifungwe) 104 = Kosi Bay

129 = Mahlongwana

132 = Matigulu

138 = Mbokodweni

**ST. LUCIA ESTUARY**

**214**

**RICHARDS BAY**

**308**

**CAPE VIDAL**

#### 142 = Mdlotane **161**

**PORT DUNFORD**

143 = Mdloti

144 = Mdumbi

148 = Mgobezeleni

150 = Mhlabatshane **^SHAKAS^ ^ROCK^**

**293**

**142**

**151**

**132**

**225**

**MTUNZINI**

#### 151 = Mhlali

157 = Mkomazi

160 = Mkweni

161 = Mlalazi Estuary

188 = Mtentu **^129^**

**283 2**

**DURBAN**

**138**

**157**

**276**

**143**

#### 200 = Mzimkulu

214 = Nhlabane

221 = Ntlonyane

225 = Nyoni

**PORT SHEPSTONE**

**13**

**MARGATE**

**7**

**SCOTTBURGH**

**47**

**150**

**37**

**200**

#### 276 = Tongati

283 = Umgeni Estuary

**160**

**MARINA BEACH**

**PORT EDWARD**

**188**

**PORT ST. JOHNS**

**28**

**71**

**144**

**COFFEE BAY**

**29**

**221**

Figure 5: Map showing a magnified section of the eastern margin, where yellow text boxes represent coastal cities and the circled numbers represent estuaries.
